# Functional Categorization of Transcriptome in the Species *Symphysodon aequifasciatus* Pellegrin 1904 (Perciformes: Cichlidae) Exposed to Benzo[a]pyrene and Phenanthrene

**DOI:** 10.1371/journal.pone.0081083

**Published:** 2013-12-03

**Authors:** Renato de Souza Pinto Lemgruber, Nislanha Ana dos Anjos Marshall, Andrea Ghelfi, Daniel Barros Fagundes, Adalberto Luis Val

**Affiliations:** 1 Laboratory of Ecophysiology and Molecular Evolution (LEEM), Brazilian National Institute for Research in the Amazon (INPA), Manaus, Amazonas, Brazil; 2 Laboratory of Bioinformatics and modelling of middle Solimões (LBMS), Federal University of Amazon (UFAM), Coari, Amazonas, Brazil; Queen's University Belfast, United Kingdom

## Abstract

This study aims to evaluate the transcriptome alterations, through cDNA libraries, associated with the combined effects of two PAHs, benzo[a]pyrene (0.5 µg/L) and phenanthrene (50 µg/L), present in crude oil, on specimens of *Symphysodon aequifasciatus* (discus fish) after 48 h of exposure. The cDNA libraries were constructed according to the SOLiD™ SAGE™ protocol for sequencing in the SOLiD v.3 Plus sequencer. The results were analyzed by bioinformatics and differentially expressed genes were categorized using the gene ontology program. The functional categories (terms) found in the gene ontology and the gene network generated using STRING software were used to predict the adverse effects of benzo[a]pyrene and phenanthrene in the liver. In the present study, 27,127 genes (compared to *Danio rerio* database) were identified. Considering only those genes with a p-value less than or equal to 0.05 and greater than or equal to two-fold change in expression across libraries, we found 804 genes, 438 down-regulated (54%) and 366 up-regulated (46%), in the experimental group compared to the control. Out of this total, 327 genes were successfully categorized, 174 down-regulated and 153 up-regulated, using gene ontology. Using String, the gene network was composed by 199 nodes, 124 of them resulting in 274 interactions. The results showed that even an acute exposure of 48 h caused metabolic change in response to environmental contaminants, resulting in changes of cell integrity, in oxidation-reduction processes, in the immune response and disturbances of intracellular signaling of discus fish. Also the gene network has showed no central interplay cluster, exhibiting instead interconnected clusters interactions and connected sub-networks. These findings highlight that even an acute sublethal exposure of PAHs can cause metabolism changes that may affect survival of discus. Our findings using SOLiD coupled with SAGE-method resulted in a powerful and reliable means for gene expression analysis in discus, a non-model Amazonian fish.

## Introduction

The Amazon region has the highest diversity of freshwater fish in the world, with about 2,500 fish species known so far, and about 1,000 new species to be described [1], [2]. Despite its enormous importance, the Amazon fish fauna is threatened by a series of anthropogenic environmental disturbances such as overfishing [3], dam building for power generation [4] and water pollution by different sources of contaminants [5].

Discus fish (*Symphysodon aequifasciatus*) is an endemic species of the Amazon and one of the most commercially valuable species in the region, mainly as ornamental fish, for having bright colors and disc-shaped body [1], [6]. The reproductive and population parameters of this species were already studied in Piagaçu-Purus Sustainable Development Reserve (RDS-PP), lower Purus River (Brazilian Amazon) [7]. The species of this genus are unusual among fish species for parental care, in that both parents produce mucus secretions to feed their offspring after hatching [8], [9]. These features show the importance of studies concerning factors that could modify the biology of this species, such as the oil exploration and transportation down to Manaus. Discus occurs in these areas [6], [10].

The oil is extracted at the Urucu Oil Province and transported through pipelines and ships down the river to the Isaac Sabbá refinery (Reman) in Manaus to be refined [11]. Although oil and its byproducts are of great importance nowadays [12], the exploitation of this resource in the Amazon involves risks, such as the discharge of produced water and/or chronic or acute oil exposure, contaminating the environment, mainly, rivers and lakes. This type of contamination potentially causes irreversible damage to local biodiversity and social impacts [13]. Thus, several studies have tried to understand the possible biological responses of organisms of the Amazon to a possible environmental contamination by crude oil spill and oil derivatives [14], [15], [16], [17].

Among the components of oil, polycyclic aromatic hydrocarbons (PAHs) comprise a large number of organic compounds associated with toxicological effects on aquatic organisms [18], [19], [20]. As reviewed by [21], 16 PAHs are classified as priority for environmental studies by the United States Environmental Protection Agency (USEPA), including benzo[a]pyrene (BaP) and phenanthrene (Phe). Once absorbed (or uptaken) by organisms, PAHs may have its toxicity abolished, decreased or increased, by biotransformation processes, a series of reactions that transform the exogenous contaminants into more hydrophilic compounds and therefore more readily removable. However, these generated compounds that can bind covalently to cellular macromolecules such as proteins, DNA and RNA, causing cell damage, mortality, growth reduction, edema, cardiac dysfunctions, lesions, tumors, immune system damage and biochemical changes [18], [19], [22], which may compromise the health and survival of the individual.

Considering the potential risk of accidents due to petroleum mining activities and the importance of this fish species, this study aims to evaluate the sublethal effects of BaP and Phe after 48 h of exposure on the liver of the species *S. aequifasciatus*. Thus, the differentially expressed genes were identified and categorized using the Gene Ontology (GO) program [23], [24], and the gene network interactions were analyzed using the STRING (Search Tool for the Retrieval of Interacting Genes) software [25]. The identification and discussion of affected biological functions in liver of discus exposed to two PAHs indicate the risk of contaminants that can alter the normal biological function in this species and compromise its health and survival.

## Materials and Methods

### Fish sampling and exposure to PAHs

Twelve adult specimens of discus fish (*Symphysodon aequifasciatus*) were obtained from a local commercial dealer (“Prestige Aquário”, Manaus, AM, Brazil). They were acclimated in the laboratory using INPA's aerated groundwater for two weeks in 500 L tanks. During the acclimation period, animals were fed dry food pellets with 36% protein content (Purina) once a day. Forty-eight hours before the beginning of the experiments, feeding was suspended and fish were transferred to four 150 L-tanks, three fish per tank, where the experiment was performed. Fish remained without the addition of the contaminants for another 24 hours. Tanks 1 and 2 were set as control, where the fish were not exposed to any contaminant. Tanks 3 and 4 hold the fish exposed to combined sublethal levels of BaP and Phe.

The concentrations of BaP (SIGMA 40071) and Phe (SIGMA 442753) were 0.5 µg/L and 50 µg/L, respectively, both dissolved in DMSO (Dimethyl sulfoxide) (0.1 ml/L–0.01%). The same amount of DMSO (0.1 ml/L) was also added to the tanks 1 and 2 (control). The fish were carefully collected with an aquarium fish net after 48 h of exposure and euthanized by completely cervical spine dislocation. The liver of each fish was immediately excised using a sterile scissor and tweezers, stored separately in 2 mL-tube and immediately frozen in liquid nitrogen for proper conservation. An ichthiometer and a semi-analytical balance were used to determine the fish length and mass, respectively.

The physicochemical parameters of water were monitored at 0, 24 and 48 h. The pH values were measured using a pHmeter (Micronal model B374) and dissolved oxygen was measured with an oxygen meter (YSI, model 55/12 FT). The water temperature was maintained at 28°C.

### Total RNA extraction and SOLiD-SAGE cDNA library construction

Total RNA was extracted from *S. aequifasciatus* liver (20–30 mg) using 500 µL of TRIzol® (Invitrogen) according to the instructions of the manufacturer. RNA integrity was checked by 1% (m/v) agarose gel electrophoresis and the concentration and purity of RNA were determined by spectrophotometer readings at 260 and 280 nm, using NanoDrop®2000 (Thermo Scientific). RNA from six individuals (N = 6) was pooled in equal quantities to provide template for SOLiD libraries. Then, cDNA libraries were constructed according to the SOLiD™ SAGE™ (Serial Analysis of Gene Expression) protocols [26], [27], [28], using the full-scale emulsion PCR. The libraries were deposited in a 4-well slide for sequencing in the SOLiD v.3 Plus sequencer.

### Data analysis

The data generated by the SOLiD were analyzed using our own pipeline (Tamandua), for SAGE data analysis with next generation sequencers [29]. The genes were identified by comparison with the *Danio rerio* (zebrafish) database. Then, genes expression was normalized and was separated into two groups considering the value of their expressions, down-regulated (negative expression) and up-regulated (positive expression), in the experimental group compared to the control. Genes were considered differentially expressed when: 1) A greater-than-or-equal-to two-fold change in expression across libraries was observed; and 2) The p-value was less than or equal to 0.05. These differentially expressed genes were submitted to the gene ontology program (AmiGO version 1.8), against the database of annotated genes for *D. rerio*. The GO program has three general categories: Biological Process (BP), Cellular Component (CC), and Molecular Function (MF). For each category, there is a structure of terms or more specific levels in order to categorize the genes.

A paired student t-test was applied for each Gene Ontology (GO) term to evaluate differences between the measurements for the BaP and Phe-exposed and non-exposed group. For these tests, a p-value less than 0.05 were considered significant. Data were analyzed using R statistical environment version 3.0.1 [30].

We therefore used the STRING (Search Tool for the Retrieval of Interacting Genes) software (v.9.1) to look for known interactions among the genes. This is a large database of known and predicted protein interactions that cover more than 1,100 organisms [25]. All the down- and up-regulated genes (those successfully categorized in GO database) were submitted, together, using their gene symbols, against the STRING *D. rerio* database, selecting proteins interactions. This database includes direct (physical) and indirect (functional) associations that are derived from four sources: genomic context, high-throughput experiments, conserved co-expression and previous knowledge from literature. A confidence score for every protein–protein association was assigned to the network having that a higher score means that an association is supported by several types of evidence. Cluster networks were created using a value of 2 for the MCL clustering algorithm, which is included in the STRING website.

These two analyses were used to obtain insights into the BaP and Phe-induced changes of biological functions and the types of functions that were affected in discus by this combined exposure.

The high-throughput sequencing data have been deposited in the National Center for Biotechnology Gene Expression Omnibus (NCBI GEO) database (Accession number GSE51149).

### Ethics statement

All experimental procedure was approved by the Ethics Committee on Animal Use of the Brazilian National Institute for Research in the Amazon (CEUA-INPA) (Protocols 001/2011 and 050/2012).

## Results

No animal died during the experiment. The discus fish (*S. aequifasciatus*) used in both control and experimental groups showed no significant difference in the length (cm) and mass (grams) (N = 6 for all treatments, “t-student” test, p<0.05) (**Table 1**). The measured physicochemical parameters also showed no change between the control and the experimental group (p<0.05, t-test), indicating that the experiment was performed under similar conditions in the tanks (**Table 2**).

**Table 1:** Length (cm) and mass (g) of the fish in the control and experimental group.

Group	Length (cm)	Mass (g)
**Control**	14.42±0.90	106.67±20.66
**Experimental**	13.40±1.39	80.83±22.68

Results are presented as mean and standard deviation. (N = 6 to all treatments).

**Table 2:** Physicochemical parameters of water in tanks.

Group tank	pH	Dissolved oxygen (mg.L^−1^)	Temperature (°C)
**Control**	6.78±0.26	5.12±0.47	28
**Experimental**	6.72±0.24	5.37±0.62	28

Results are presented as mean and standard deviation.

**Figure 1:**
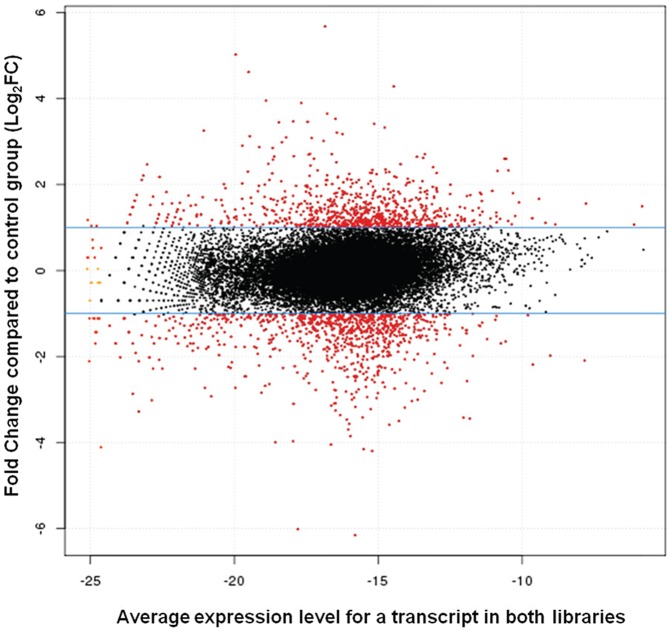
MA-plot of differentially expressed genes in benzo[a]pyrene and phenanthrene-exposed and non-exposed fish. The x-axis is an estimate of the relative abundance of the transcript (a measure of the average expression level for each transcript across the two libraries), and the y-axis is a measure of differential expression (Log_2_FC). The solid blue horizontal lines show where genes with two-fold differences in expression fell (Log_2_FC = 1), so all the genes with differential expression in this analysis show at least two-fold differences between treatments (red points).

We sequenced 95,278,865 tags in total, 32,311,242 tags in the control group and 62,967,623 tags in the experimental group. The comparison of control and experimental libraries resulted in a total of 1,140,779 unique tags. Out of this total, 27,127 genes were identified. Considering only those genes with a p-value less than or equal to 0.05 and a greater-than-or-equal-to two-fold change in expression across libraries, we found 804 genes, 438 down-regulated (54%) and 366 up-regulated genes (46%), in the experimental compared to the control group. Based on the normalized expression of genes, we generated a MA-plot graph, for comparison of gene expression in the experimental and control groups. The x-axis is an estimate of the relative abundance of each transcript across the two libraries, and the y-axis is a measure of differential expression (Log_2_FC). The solid blue horizontal lines show where genes with two-fold change in expression fell, so all the genes with differential expression in this analysis show two-fold, or greater, differences between treatments (red points) (Figure 1).

Out of the total of genes submitted to the gene ontology (compared to *D. rerio* database), 327 genes were successfully categorized, 174 down-regulated and 153 up-regulated genes. The down-regulated genes resulted in 399 terms, corresponding to 199 terms within the group related to biological process, 56 in cellular component and 144 in molecular function, while the up-regulated genes resulted in 413 terms, corresponding to 203 terms within the group related to biological process, 48 in cellular component and 162 in molecular function. Since multiple terms may be identified for the same gene, only the most relevant terms in the context of this study were selected. For each three broad categories (biological process, cellular component and molecular function), two pie chart graphs were generated, one containing the corresponding Top20 terms of down-regulated genes and the other containing the Top20 terms of up-regulated genes. In both cases, Top20 terms are those with highest percentage of representation within each of the three general categories. The other terms, in addition to Top20, with percentage of representation equal to or less than 1% of total, were grouped and are represented in the pie chart as well (Complete list in Tables S1 and S2).

**Figure 2:**
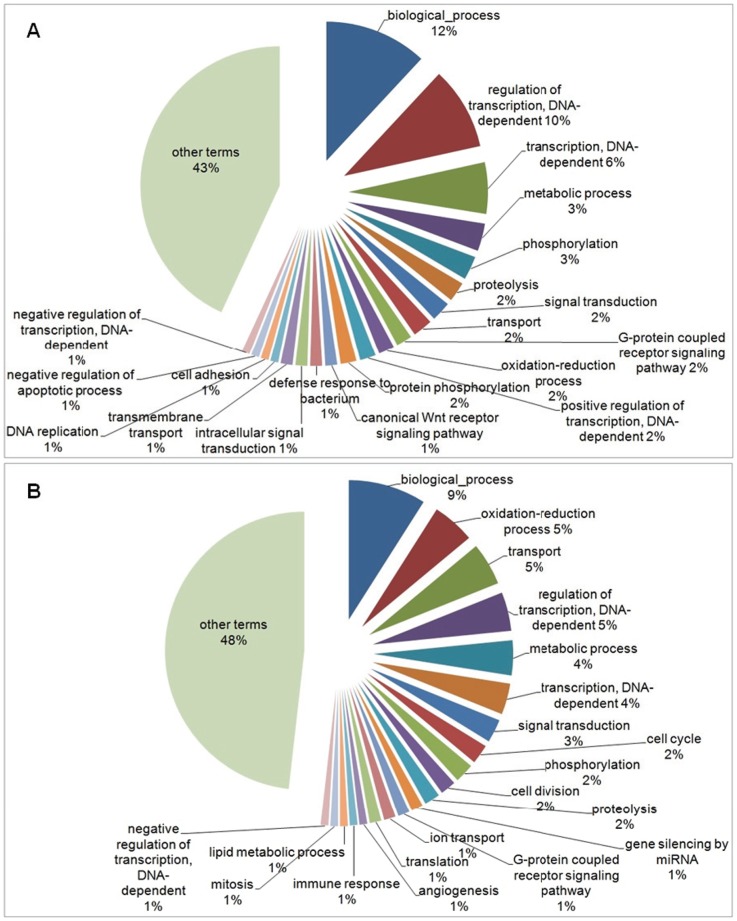
Pie-chart of Top20 GO terms for Biological Process category. Representation of Top20 GO terms results from the down- (a) and up-regulated genes (b), for Biological Process category, in the experimental group compared to control. The other terms, in addition to Top20, were represented together.

**Figure 3:**
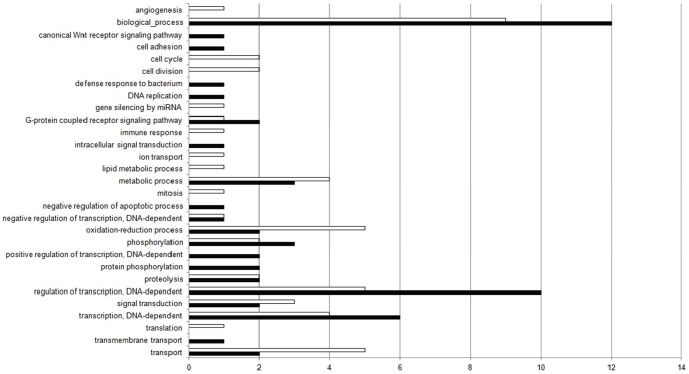
Comparison of Top20 GO terms found in the Biological Process category. Percentage of Top20 terms related to the down-regulated (black bars) and up-regulated genes (white bars), selected in the Biological Process category.

For the down-regulated genes, 109 terms were selected in the biological process category, 12% of which classified in the “biological process” term, followed by “regulation of transcription, DNA-dependent” (10%), “transcription, DNA-dependent” (6%), “metabolic process” (3%), phosphorylation (3%). The remaining Top20 terms were represented at proportions less than or equal to 2%. The other 88 terms, in addition to the Top20, totalized 43% (Figure 2A). For the up-regulated genes, 120 terms were selected in the biological process category, 9% of which classified in the “biological process” term, followed by “oxidation-reduction process” (5%), “transport” (5%) and “regulation of transcription, DNA-dependent” (5%). The other 100 terms, in addition to the Top20, totalized 48% (Figure 2B). Figure 3 shows the comparison between the percentage of Top20 terms related to the down-regulated (black bars) and up-regulated genes (white bars), selected in the Biological Process category.

**Figure 4:**
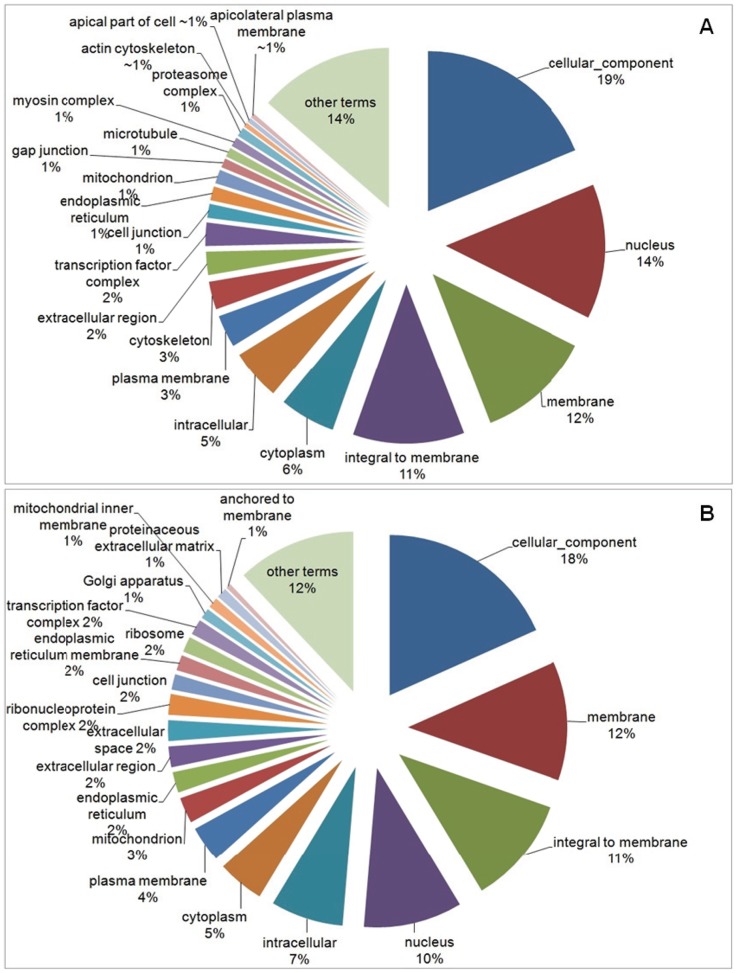
Pie-chart of Top20 GO terms for Cellular Component category. Representation of Top20 GO terms results from the down- (a) and up-regulated genes (b) for Cellular Component category, in the experimental group compared to control. The other terms, in addition to Top20, were represented together.

**Figure 5:**
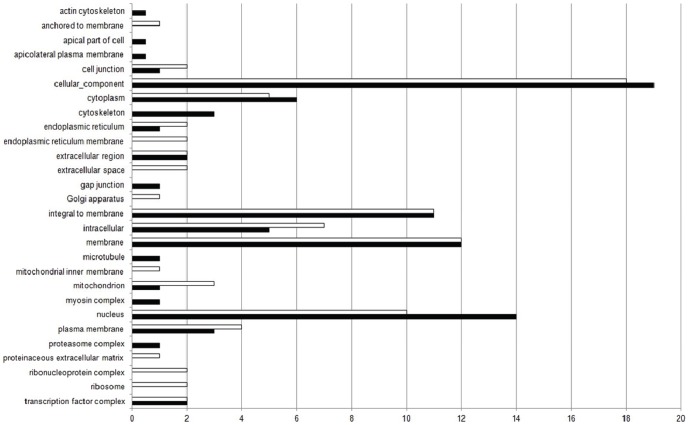
Comparison of Top20 GO terms found in the Cellular Component category. Percentage of Top20 terms related to the down-regulated (black bars) and up-regulated genes (white bars), selected in the Cellular Component category.

Among the 49 terms selected for the cellular component category, for the down-regulated genes, 19% were classified in “cellular component”, 14% in “nucleus”, 12% in “membrane”, 11% in “integral to membrane”, 6% in “cytoplasm” and 5% in “intracellular” term. The other 29 terms, in addition to the Top20, totalized 14% (Figure 4A). For the up-regulated genes, among 43 terms selected, 18% were classified in “cellular component” term, followed by “membrane” (12%), “integral to membrane” (11%), “nucleus” (10%), “intracellular” (7%) and “cytoplasm” (5%). The other 23 terms, in addition to the Top20, totalized 12% (Figure 4B). Figure 5 shows the comparison between the percentage of Top20 terms related to the down-regulated (black bars) and up-regulated genes (white bars), selected in the Cellular Component category.

**Figure 6:**
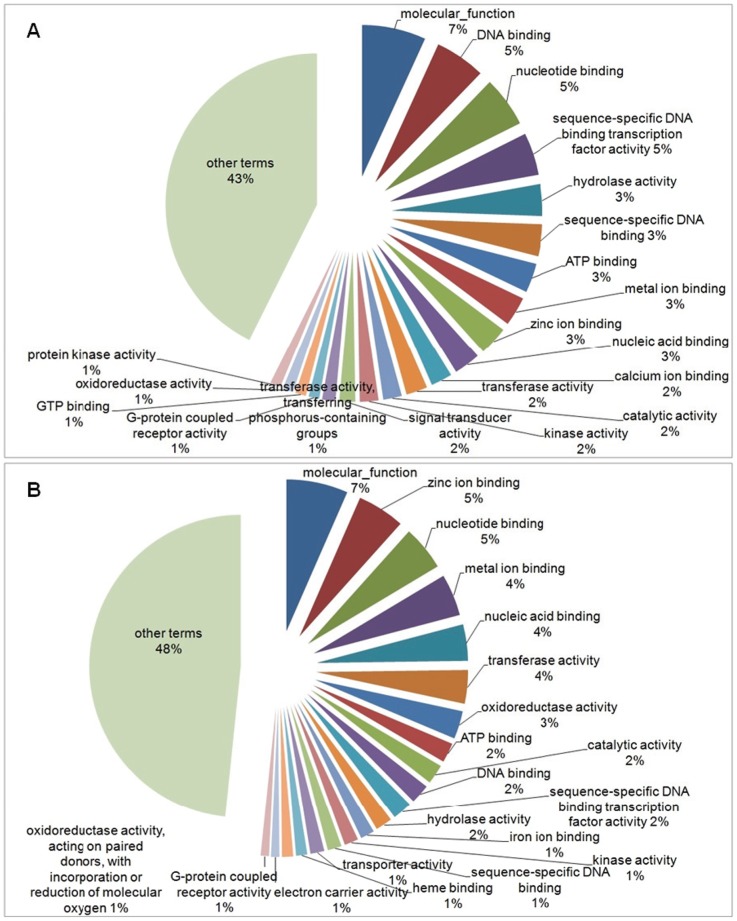
Pie-chart of Top20 GO terms found in the Molecular Function category. Representation of Top20 GO terms results from the down- (a) and up-regulated genes (b) for Molecular Function category, in the experimental group compared to control. The other terms, in addition to Top20, were represented together.

**Figure 7:**
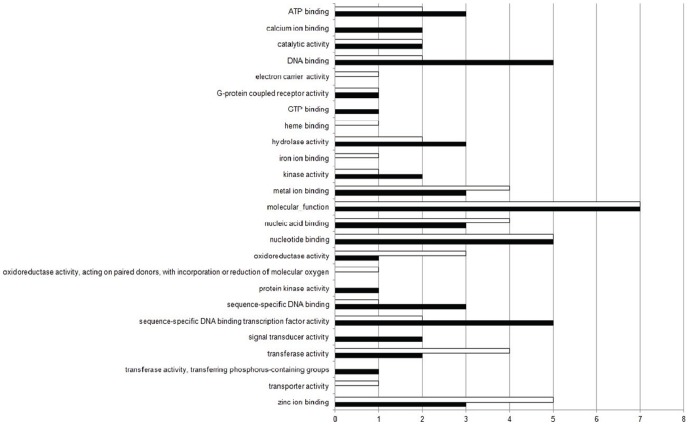
Comparison of Top20 GO terms found in the Molecular Function category. Percentage between the Top20 terms related to the down-regulated (black bars) and up-regulated genes (white bars), selected in the Molecular Function category.

Within the molecular function category, among the 140 terms selected for the down-regulated genes, the first four Top20 terms were: “molecular function” (7%), “DNA binding” (5%), “nucleotide binding” (5%) and “sequence-specific DNA binding transcription factor activity” (5%). The other 120 terms, in addition to the Top20, totalized 43% (Figure 6A). For the 160 terms selected for the up-regulated genes, 7% related to “molecular function”, followed by “zinc ion binding” (5%), “nucleotide binding” (5%), “metal ion binding” (4%) and “nucleic acid binding” (4%). The other 140 terms, in addition to the Top20, totalized 48% (Figure 6B). Figure 7 shows the comparison between the percentage of Top20 terms related to the down-regulated (black bars) and up-regulated genes (white bars), selected in the Molecular Function category.

Using the STRING software, a confidence gene network interaction was generated merging all genes expressed (successfully categorized in the GO) in the liver of discus fish. The proteins are represented with nodes and the interactions with continuous lines represent direct interactions (physical), while dashed lines represent indirect interactions (functional). Thicker lines indicate higher confidence score for protein-protein association. Nodes with no interactions have been hidden.

**Figure 8:**
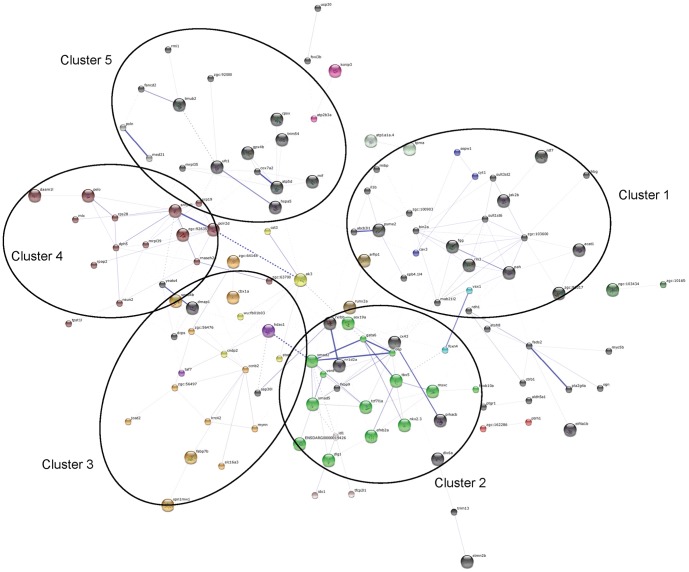
Gene network interactions using the STRING v.9.1 software. Interactions of genes (or proteins) expressed in the liver of discus fish 48 h exposed to benzo[a]pyrene and phenanthrene. Continuous lines represent direct interactions (physical), while dashed lines represent indirect ones (functional). Thicker lines represent stronger associations. Nodes with no interactions have been hidden.

199 nodes composed the final confidence gene network, 124 of them resulted in 274 interactions. Out of all interactions, seven were labeled as “highest confidence” (score>0.9), seven were labeled as “high confidence” (0.7–0.9), 18 were labeled as “medium confidence” (0.4–0.7) and 242 were labeled as “low confidence” (<0.4). The gene network shows five major clusters, namely Cluster 1, Cluster 2, Cluster 3, Cluster 4 and Cluster 5, with interconnected interactions and sparsely connected sub-networks (Figure 8, Tables S3 and S4). The genes grouped in each of the five clusters are highlighted in Figures S1–S5 and Table S5.

## Discussion

The oil exploitation in the Amazon is a reality. Considering the potential risk of accidents due to petroleum activity, including mining and transportation, it is important to investigate how species will respond to a possible oil contamination, from molecular to ecological perspective. Since a xenobiotic response in an organism begins at molecular or biochemical levels, they have high toxicological relevance. The adverse effects will increase over time if they are not compensated by cellular protective mechanisms [31], [32].

The molecular responses to contaminants exposure in Amazon fish fauna remain mostly unknown for a large number of species. This study aimed to evaluate the sublethal effects of BaP and Phe-exposure on transcriptional gene regulation in the discus fish, *S. aequifasciatus*, by its association with functional categories (terms) found in the gene ontology and gene network interactions generated by STRING software to predict the adverse effects in the liver. Furthermore, we were the first to use the SOLiD platform, a next-generation sequencing (NGS), to assess the transcriptome in this species.

Using the SOLiD v.3 Plus platform, about 95 millions of sequenced tags in the two libraries, control and experimental, were generated. By comparison with *D. rerio* database, we were able to identify 27,127 genes. Since *D. rerio* has 28,770 annotated genes [33], if we consider the same number of genes in these two species, this would represent approximately 94% of the genes identified for the species *S. aequifasciatus*. The data resulting from this study using SOLiD-SAGE methodology coupled with proper bioinformatic tools we could identify a large number of genes, even for a non-model species, which emphasizes the importance of this study, a pioneer for this neotropical fish A series of functional categories (according to GO) were identified and associated with responses to environmental stress. As pointed out by [23], [24], it is important to note that the genes can be annotated under more than one ontology term, since a gene, or its product, can have more than one molecular function, being involved in several biological processes, as well as acting at several places in the cell. Furthermore, the gene ontology annotation considers the whole organism, not an individual organ. Because of this, we selected only those terms that were relevant in context of this study. For example, for the gene *stmn2b*, the gene ontology search returns, in the biological process category, that the gene can be annotated to the terms: “nervous system development” and “regulation of microtubule polymerization or depolymerization”. Since we are working with liver, the term “nervous system development” does not make sense in the context of our study and was therefore excluded from our analysis (Table S2. The term “nervous system development” is not being shown in this table).

The exposure to xenobiotics compounds leads to activation of detoxification process, mainly in liver (central detoxification organ), which includes different types of enzymes classified in phase-I and phase-II detoxification process. The main biotransformation process is related with the cytochrome P450 heme proteins, a family of microsomal monooxygenases enzymes found mainly in the endoplasmic reticulum membrane [18], [34]. From our results, an increase in transcripts encoding “oxidation-reduction process” (BP) (p-value = 3.943e-13), “endoplasmic reticulum membrane” (CC) (p-value = 0.0009649), “electron carrier activity” (MF) (p-value = 0.001611), “heme binding” (MF) (p-value = 7.965e-05), “iron ion binding” (MF) (p-value = 9.934e-05), “oxidoreductase activity” (MF) (p-value = 1.054e-11) and “oxidoreductase activity, acting on paired donors, with incorporation or reduction of molecular oxygen” (MF) (p-value = 0.00245) were found (Figures 2B, 3, 4B, 5, 6B and 7). These functional categories together indicate an increase of xenobiotic-metabolizing process related to BaP and Phe-exposure. The main objective of biotransformation process is to facilitate the excretion of compounds by its transformation to more water-soluble compounds, in a series of reactions including oxidation, reduction and hydrolyzing. In general, the reaction cascade involves substrate binding to prosthetic heme ferric iron (Fe3^−^) of the monooxygenases, reduction of this iron by electron transfer and subsequently, binding of O_2_. The next steps involve the addition of a second electron, which originates peroxide, followed by cleavage of the O–O bond, hydroxylation of the substrate and, finally, the release of the product [18], [34]. Furthermore, functional categories, in up-regulated genes, associated with terms “translation” (BP) (p-value = 1.004e-08), “endoplasmic reticulum” (CC) (p-value = 8.985e-07), “endoplasmic reticulum membrane” (p-value = 0.0009649), “Golgi apparatus” (CC) (p-value = 5.14e-07), “ribonucleoprotein complex” (CC) (p-value = 8.986e-08), “ribosome” (CC) (p-value = 2.687e-09) (Figures 2B, 3, 4B and 5), indicate an increase of translational process, which is rather associated with higher capacity of protein synthesis. Indeed, xenobiotic compounds induce firstly an increase of transcriptional (not observed here) and subsequently translational processes of the genes coding for the xenobiotic-metabolizing enzymes. Although, an increase of translational processes was observed, it cannot be fully associated with xenobiotic-metabolizing enzymes, since other processes may also take place within the cell.

We observed an enrichment of functional categories, associated with down-regulated genes, such as “cell adhesion” (BP) (p-value<2.2e-16), “actin cytoskeleton” (CC) (p-value = 6.085e-09), “cytoskeleton” (CC) (p-value<2.2e-16), “gap junction” (CC) (p-value = 0.0002338), “microtubule” (CC) (p-value = 0.004577) (Figures 2A, 3, 4A and 5). These terms may reflect an altered cytoskeletal organization, disruption of cellular shape and liver tissue integrity. The cytoskeleton is a complex of diverse filamentous structures. The three major types are: actin filaments, intermediate filaments and microtubules. The role of cytoskeleton is not only as structural component, but also participates in many other processes, such as: intracellular transport, secretion, organization of cytoplasm, and generation of mechanical forces within the cell. The cytoskeleton and its structure are important to maintain the shape and integrity of a cell and a tissue, and for a cell-matrix and cell-cell adhesion and communication as well [35], [36]. Like our study, cytoskeleton disorganization by contamination-exposure was observed in hepatic cells of zebrafish (*D. rerio*) exposed to mercury [37]. Larvae of *D. rerio* exposed to Bisphenol-A also resulted in a deregulation of actin cytoskeleton and tight junction [38]. Also microcystins-exposure (a type of toxin produced by cyanobacteria) in primary cultured rat hepatocytes, induced a disruption and collapse of cytoskeleton [39]. These contaminants led to cytoskeleton alterations, resulting in abnormal cellular integrity, like found in our work. Deleterious effects on the integrity of actin cytoskeleton were also observed in haemocytes of copper- and cadmium-exposed mussel (*Mytilus galloprovincialis*). Other terms found, in down-regulated genes, may also be related to cytoskeleton alterations: “GTP binding” (MF) (p-value<2.2e-16) and “calcium ion binding” (MF) (p-value<2.2e-16) (Figures 6A and 7), since both GTP and Ca^2+^, despite their importance as intracellular signaling molecules, are related to proteins that control the filament formation – resulting in microtubule (de-) polymerization – and cross-linking of the actin network [35], [36]. In addition, the “canonical Wnt receptor signaling pathway” (BP) (p-value = 5.066e-06) term (or Wnt/β-catenin signaling pathway) (Figures 2A and 3), associated with down-regulated genes, may also be related with observed alterations in cytoskeleton, as discussed above. Since β-catenin, a component of Wnt complex, is also an important component of cadherin complexes, involved in cell-cell junction, and is needed to form links between cadherins and intracellular actin cytoskeleton [40].

Environmental contaminants can also alter normal process within the cell, inducing adverse effects such as DNA adduct [41], necrosis [42], edema and cardiac malformations [17], [38], and disruption of ionic regulatory mechanism [16], [43]. Tissue damage and stress can activate signal pathways to cell turnover, apoptosis and also may leads to cancer [44], [45]. We, interestingly, observed an overrepresentation of “cell cycle” (BP) (p-value = 0.002141), “cell division” (BP) (p-value = 0.002466), “mitosis” (BP) (p-value = 0.04194), “angiogenesis” (BP) (p-value = 0.0002882) terms and underrepresentation of “negative regulation of apoptotic process” (BP) (p-value = 2.694e-10) term (Figures 2 and 3). Considering the great capacity of liver regeneration [46], [47], these terms suggest, despite the adverse effects of BaP and Phe-exposure, an activation of signaling pathway leading to cell turnover, resulting in hepatic tissue remodeling or regeneration affected by xenobiotic compounds. Cell turnover was also detected in the epithelial intestine cell of the species *Epinephelus coioides* after one week and also after four weeks of exposure to BaP [48]. Furthermore, these authors, as well as our study, did not found an increase of apoptotic process between the treatments and the controls. Epithelial intestine cell turnover associated with mucus production was also detected in carp (*Cyprinus carpio*) induced orally by endosulfan, a type of insecticide. Although, the authors suggested that it seems to be associated with activation of adaptive mechanisms to protect the epithelium from pesticide [49]. Tilapia (*Oreochromis mossambicus*) epithelial gill cells exposed to hyperosmotic salinity stress also presented cell turnover and proliferation [50]. In addition, down-regulated genes associated with “canonical Wnt receptor signaling pathway” term may also be related to antagonize the known oncogenic actions of Wnt/β-catenin signaling network [40].

The “canonical Wnt receptor signaling pathway” (BP) (p-value = 5.066e-06) term also may be related to underrepresentation of “intracellular signal transduction” (BP) (p-value = 1.39e-15), “GTP binding” (MF) (p-value<2.2e-16) and “signal transducer activity” (MF) (p-value<2.2e-16) terms (Figures 6A and 7). The Wnt family is related with a broad range of processes within the cell, by transcriptional activation of target genes related to cell fate determination, cell proliferation, sense alterations in fuel availability and cellular stress, cancer, cellular metabolism and many other functions that still remain to be addressed to understand this pathway. Indeed, the canonical Wnt-signaling pathway (or β-Catenin-dependent Wnt signaling) has been subjected of intense investigation over the last years [40], [51]. To elucidate all the mechanisms implicated in the Wnt pathway is beyond the scope of this work. We suggest future studies to understand the regulation of gene expression associated with this important and complex signaling pathway in response to contaminants in fish.

The stress can suppress immune function in fish. In this study, we observed an overrepresentation of “defense response to bacterium” (BP) (p-value = 0.006356) term (Figures 2A and 3) associated with down-regulated genes, which indicate a decreased resistance to infection. Immune suppression was also observed by [52] in *Pomacentrus moluccensis* associated with heat-responsive genes. The immune response in trout (*Salmo clarkii*) was also affected in a lake contaminated by different types of contaminants [53]. A suppression of immune function and a depletion of resistance against bacterium after 48 h-exposure to BaP were also detected in Japanese medaka (*Oryzias latipes*) [54]. But, curiously, we also noted an increase of transcripts associated with “immune response” (BP) (p-value = 4.333e-05) term (Figures 2B and 3). These two terms together suggest an alteration of immune response associated with BaP and Phe-exposure and that the spent-energy involved in the contaminants metabolism provide a challenge to the immune system. However, further investigation is needed to understand the role of contaminants in the immune functions in discus fish. Indeed, although the effects of PAHs in fish commonly suppress immune functions, it depends on the mode of exposure, the dose used and/or the species being analyzed [20].

Other interesting term associated with up-regulated genes was “gene silencing by miRNA” (BP) (p-value = 8.626e-05) (Figures 2B and 3). MicroRNAs (miRNAs) are endogenous and small non-coding RNAs that can play important regulatory roles in animals and plants and are involved in post-translational gene regulation by targeting mRNAs for cleavage or translational repression. Their roles included regulation of developmental process, cell differentiation and proliferation, apoptosis and cancer susceptibility [55]. Additional studies would shed light on the regulatory roles of microRNAs in discus fish exposed to contaminants, since its regulatory mechanisms are still unclear.

Finally, the first terms observed in the pie chart graphs, for the three broad categories, are: “biological process”, “cellular component” and “molecular function”, respectively for each category with the same name (Figures 2–7). According to the gene ontology definition, these terms are used for those gene products whose biological process, cellular component and molecular function, respectively, are not yet known in a more specific level. Since these genes associated with these “unknown” functional categories were expressed in this study, it suggests that these genes may also take place in important roles in BaP and Phe-metabolism in discus fish. However, their functions remain to be elucidated as well as their roles in contaminant exposure.

Analyses with STRING revealed no central interplay cluster, exhibiting network interactions with different clusters connected. The interaction between genes in cluster 4 reveals processes involved in the transcriptional and translational regulation in the cell and cell metabolism as well. The genes presented in this cluster are related, among others, with functions such as cell cycle and division (*pelo*) and rRNA export from nucleus (*rps28*), constituent of ribosome (*rps28* and *snrpd1*), metabolic process (*dph5*). These functions play an important role in the control of intracellular metabolism and protein folding. From cluster 2, we note the interactions between genes related with intracellular signal transduction and control of transcription. The down-regulated gene *tcf7l1a*, which is related to canonical Wnt receptor signaling pathway and *smad* genes, up-regulated in our study, are both related to intracellular signal transduction. These genes physically interact with *vent, mscx, gata6* and *oep* genes and indirectly with *tbx5*. All genes, but *mscx*, were down-regulated. These down-regulated genes agree with the fact that they are related with transcription factor activity and are involved in biological process of regulation of transcription and DNA binding, which trigger gene expression. Also, the interactions between *tbx5, gata6, oep* and *smad2* were labeled with a high score from previous knowledge by text mining in STRING database (Table S4). These observations are connected, as discussed above for gene ontology analyses, with an increase of translational processes (and not observed, in fact, of transcriptional) related with xenobiotic-metabolizing response.

Other observation is the high scored relationship between *cox7a2* (cytochrome c oxidase, subunit VIIa 2) and *atp5d* (ATP synthase, H^+^ transporting, mitochondrial F1 complex, delta subunit) genes in cluster 5. These genes participate in the energy system production and are located in the mitochondrial membrane, which function is ATP synthesis in the respiratory chain reaction [56]. This interaction shows the workflow control for energy production regarding the metabolic demand in liver. Also, it is observed the interaction of these both genes with the up-regulated *gpx4b* (glutathione peroxidase 4b) gene that is related with the response for oxidative stress produced within the cell, particularly in mitochondria. Actually, glutathione peroxidases are also largely used as a potential biomarker for environmental contamination [18].

As mentioned above, integrating STRING result, a global analysis from clusters 1 to 5 highlighted no central interplay cluster or gene. The genes interactions occur in intricate pathways integrating down- and up-regulated genes. Although the up-regulated *ak3* gene could be pointed out as a potential integrator center, in a first moment, its interaction with other genes was not high scored by STRING program. Adenylate kinase (ak) proteins catalyze the interconversion of ADP to ATP and AMP, regulating energetic and metabolic signaling pathways within the cell, securing efficient cell energy economy, signal communication and stress response [57]. Nevertheless, the absence of a key cluster or gene high scored may be linked with the challenge to point out one main biomarker in environmental contamination studies. As reviewed by [18], the gene expression response to contaminants in fish depends on many factors, such as the type and concentration of contaminant, the age and species, water conditions and the tissue analyzed. For example, *cyp1a* gene is largely used as a potential biomarker in oil contamination [18], [58], but its expression as biomarker is also susceptible. While our study this gene did not appear up-regulated, [17] identified *cyp1a* as a potential biomarker in *Astronotus ocellatus* using oil-derived contaminants. Meanwhile, although [59] observed that crude oil induced CYP1A expression in tambaqui (*Colossoma macropomum*), as expected, they also showed that the CYP1A activity is induced by humic substances (HS), which is naturally presented in Amazon rivers [60]. In addition, many others parameters interact with the biota and the environmental challenge-response result from a complex gene regulation process, not yet fully understood. This highlights the importance of further studies in this area and the importance of avoiding environmental pollution.

Taken together, the present data suggest that the response of liver of discus fish to BaP and Phe-exposure is an integrated gene network, instead of a cascade of a manager gene or cluster. In addition, this gene network represents the first comprehensive transcriptome map for the liver in discus fish exposed to BaP and Phe and provides an initial comparison base for further transcriptome studies.

Our findings using the SOLiD, a next-generation sequencing, coupled with SAGE-method resulted in a powerful and reliable means for gene expression analysis in discus fish, a non-model Amazonian organism, which lacks a reference genome. The results from this study also showed that the combined sublethal 48 h exposure of benzo[a]pyrene and phenanthrene in discus fish (*S. aequifasciatus*) induced an increase of oxidation-reduction and translational processes, disruption of cell integrity, disturbance of intracellular signaling and immune function alterations. However, despite the adverse effects, the results also indicated a counter-regulatory response leading to liver cell renewal.

## Supporting Information

Figure S1 **Cluster 1 from gene network interactions using the STRING v.9.1 software.** Detail of genes grouped in the cluster 1 (Figure 8) that were expressed in the liver of *Symphysodon aequifasciatus* exposed to benzo[a]pyrene and phenanthrene for 48 h.
(TIF)

Figure S2 **Cluster 2 from gene network interactions using the STRING v.9.1 software.** Detail of genes grouped in the cluster 2 (Figure 8) that were expressed in the liver of *Symphysodon aequifasciatus* exposed to benzo[a]pyrene and phenanthrene for 48 h.
(TIF)

Figure S3 **Cluster 3 from gene network interactions using the STRING v.9.1 software.** Detail of genes grouped in the cluster 3 (Figure 8) that were expressed in the liver of *Symphysodon aequifasciatus* exposed to benzo[a]pyrene and phenanthrene for 48 h.
(TIF)

Figure S4 **Cluster 4 from gene network interactions using the STRING v.9.1 software.** Detail of genes grouped in the cluster 4 (Figure 8) that were expressed in the liver of *Symphysodon aequifasciatus* exposed to benzo[a]pyrene and phenanthrene for 48 h.
(TIF)

Figure S5 **Cluster 5 from gene network interactions using the STRING v.9.1 software.** Detail of genes grouped in the cluster 5 (Figure 8) that were expressed in the liver of *Symphysodon aequifasciatus* exposed to benzo[a]pyrene and phenanthrene for 48 h.
(TIF)

Table S1 **Down-regulated genes in** ***Symphysodon aequifasciatus*** **exposed to benzo[a]pyrene and phenanthrene for 48 h.** Only genes for which information regarding gene function is currently available in the Gene Ontology (AmiGO v. 1.8) are reported here. Where multiple terms have been identified for a gene, only those most relevant in the context of this study are reported. Genes are ranked according to Fold Change compared to control group (Log_2_FC). gene_id, gene_name and symbol are according to GenBank (National Center for Biotechnology Information). (BP  =  Biological Process; CC  =  Cellular Component; MF  =  Molecular Function).
(DOC)

Table S2 **Up-regulated genes in** ***Symphysodon aequifasciatus*** **exposed to benzo[a]pyrene and phenanthrene for 48 h.** Only genes for which information regarding gene function is currently available in the Gene Ontology (AmiGO v. 1.8) are reported here. Where multiple terms have been identified for a gene, only those most relevant in the context of this study are reported. Genes are ranked according to Fold Change compared to control group (Log_2_FC). gene_id, gene_name and symbol are according to GenBank (National Center for Biotechnology Information). (BP  =  Biological Process; CC  =  Cellular Component; MF  =  Molecular Function).
(DOC)

Table S3 **The list of genes in** ***Symphysodon aequifasciatus*** **exposed to benzo[a]pyrene and phenanthrene for 48 h submitted to STRING software (v.9.1).**
(DOC)

Table S4 **Proteins interactions in** ***Symphysodon aequifasciatus*** **exposed to benzo[a]pyrene and phenanthrene for 48 h using the STRING software (v.9.1).**
(DOC)

Table S5 **The list of genes, in** ***Symphysodon aequifasciatus*** **exposed to benzo[a]pyrene and phenanthrene for 48 h, grouped in each of the five clusters using STRING software (v.9.1).**
(DOC)
