# Supplementary figures and images for: Functional Categorization of Transcriptome in the Species Symphysodon aequifasciatus Pellegrin 1904 (Perciformes: Cichlidae) Exposed to Benzo[a]pyrene and Phenanthrene
Source: PLoS One. 2013 Dec 3;8(12):e81083. doi: 10.1371/journal.pone.0081083 (PMC3849039; doi:10.1371/journal.pone.0081083)

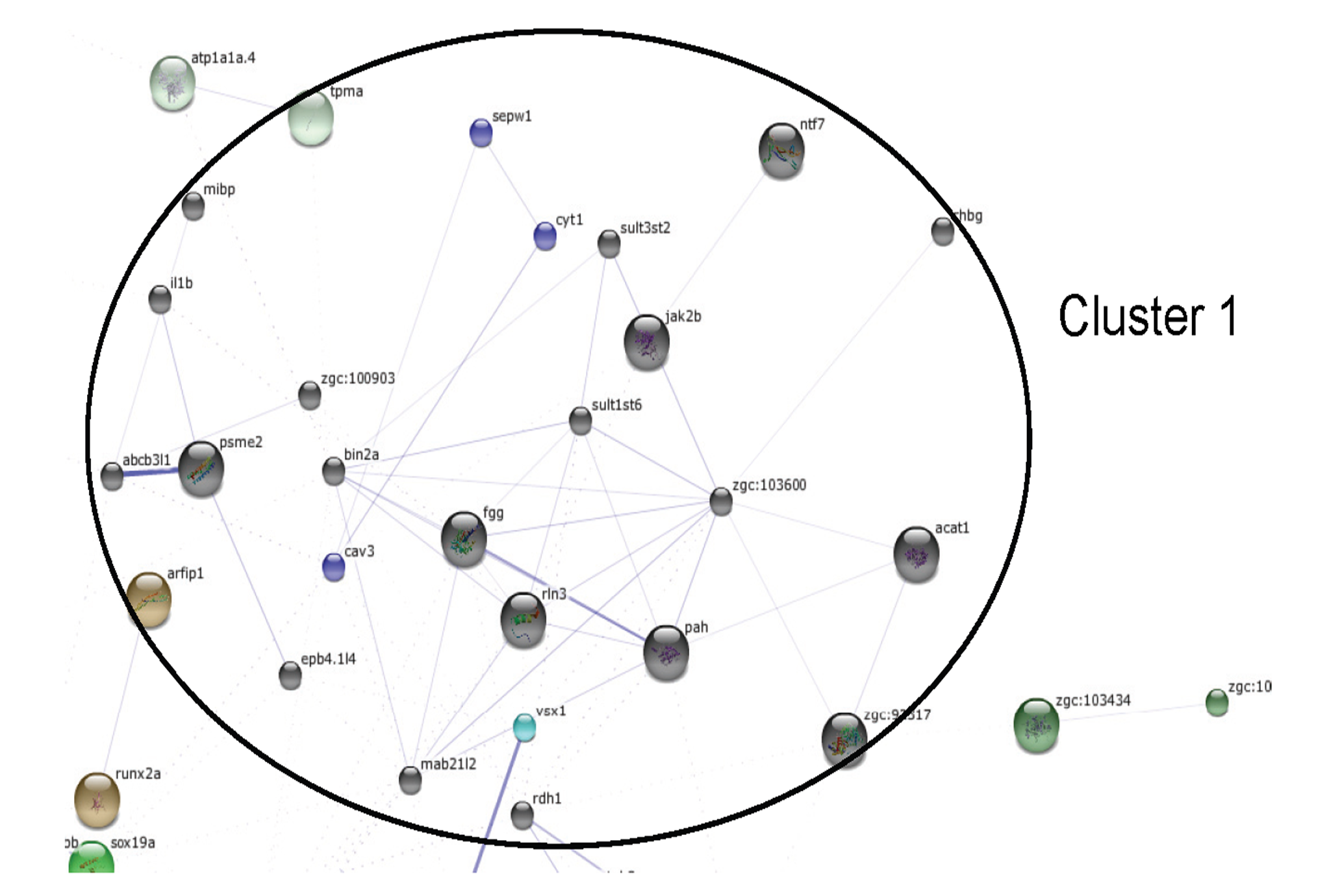

Supplement: Figure S1 — Cluster 1 from gene network interactions using the STRING v.9.1 software. Detail of genes grouped in the cluster 1 (Figure 8) that were expressed in the liver of Symphysodon aequifasciatus exposed to benzo[a]pyrene and phenanthrene for 48 h. (TIF) [file pone.0081083.s001.tif]

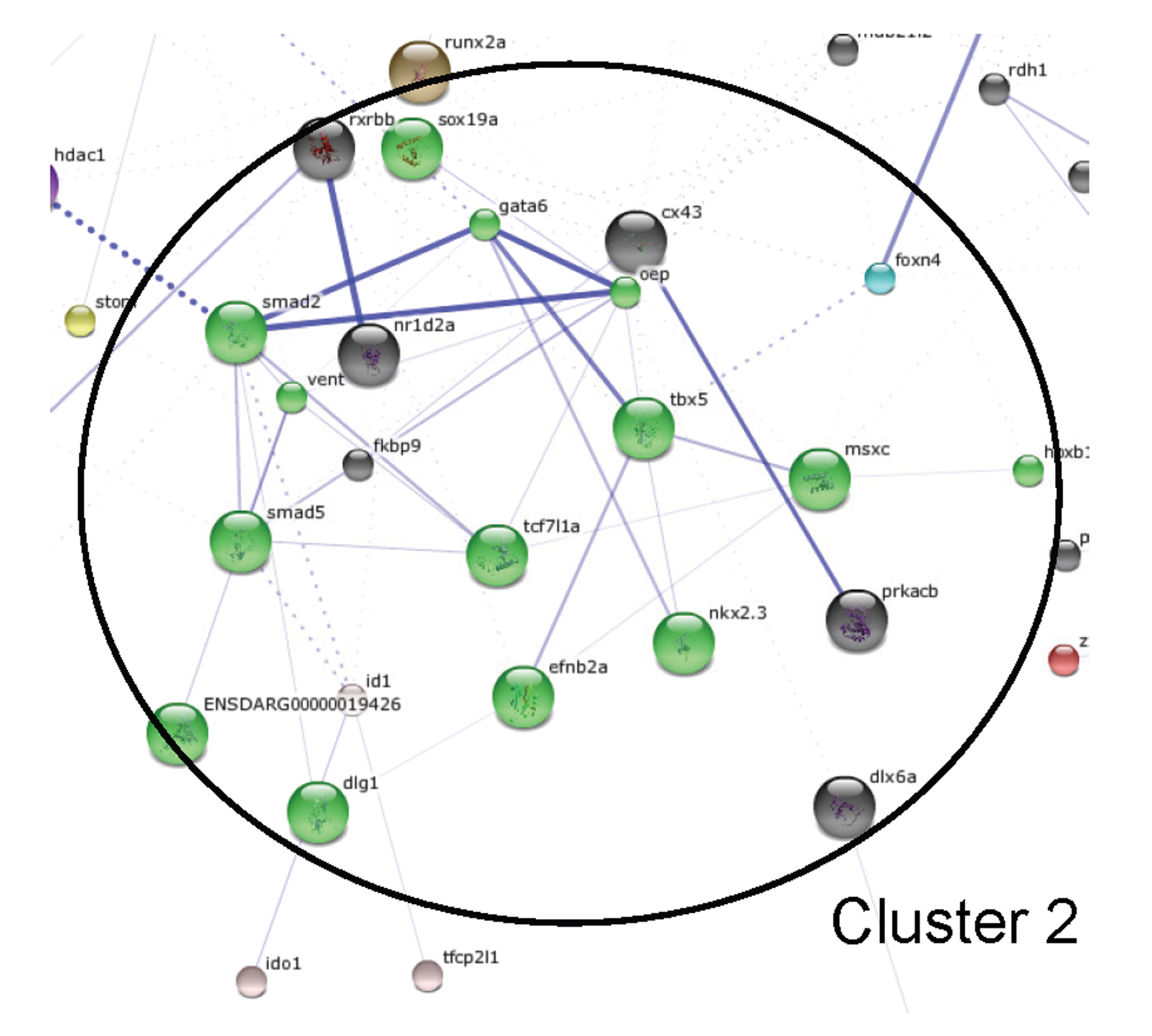

Supplement: Figure S2 — Cluster 2 from gene network interactions using the STRING v.9.1 software. Detail of genes grouped in the cluster 2 (Figure 8) that were expressed in the liver of Symphysodon aequifasciatus exposed to benzo[a]pyrene and phenanthrene for 48 h. (TIF) [file pone.0081083.s002.tif]

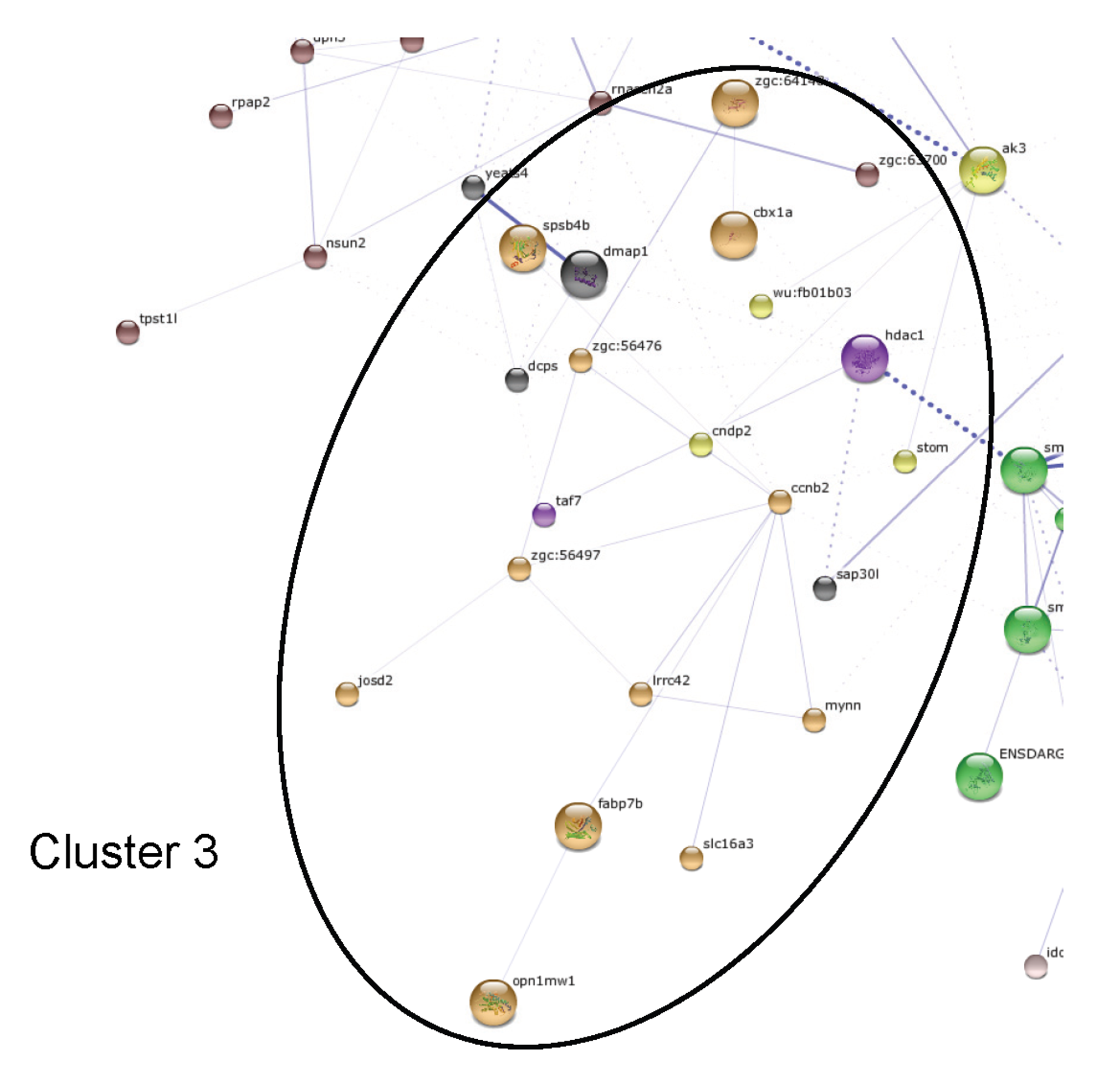

Supplement: Figure S3 — Cluster 3 from gene network interactions using the STRING v.9.1 software. Detail of genes grouped in the cluster 3 (Figure 8) that were expressed in the liver of Symphysodon aequifasciatus exposed to benzo[a]pyrene and phenanthrene for 48 h. (TIF) [file pone.0081083.s003.tif]

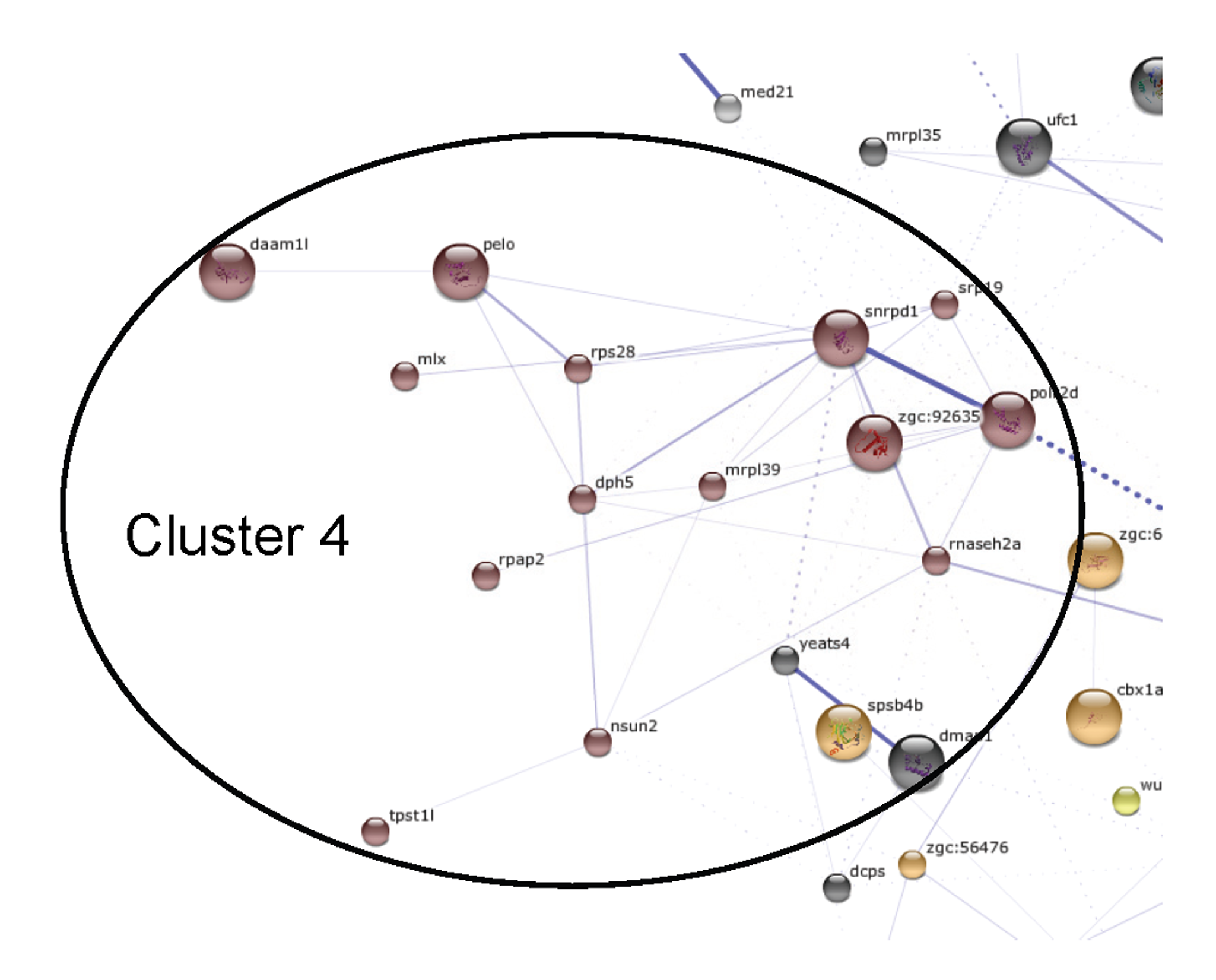

Supplement: Figure S4 — Cluster 4 from gene network interactions using the STRING v.9.1 software. Detail of genes grouped in the cluster 4 (Figure 8) that were expressed in the liver of Symphysodon aequifasciatus exposed to benzo[a]pyrene and phenanthrene for 48 h. (TIF) [file pone.0081083.s004.tif]

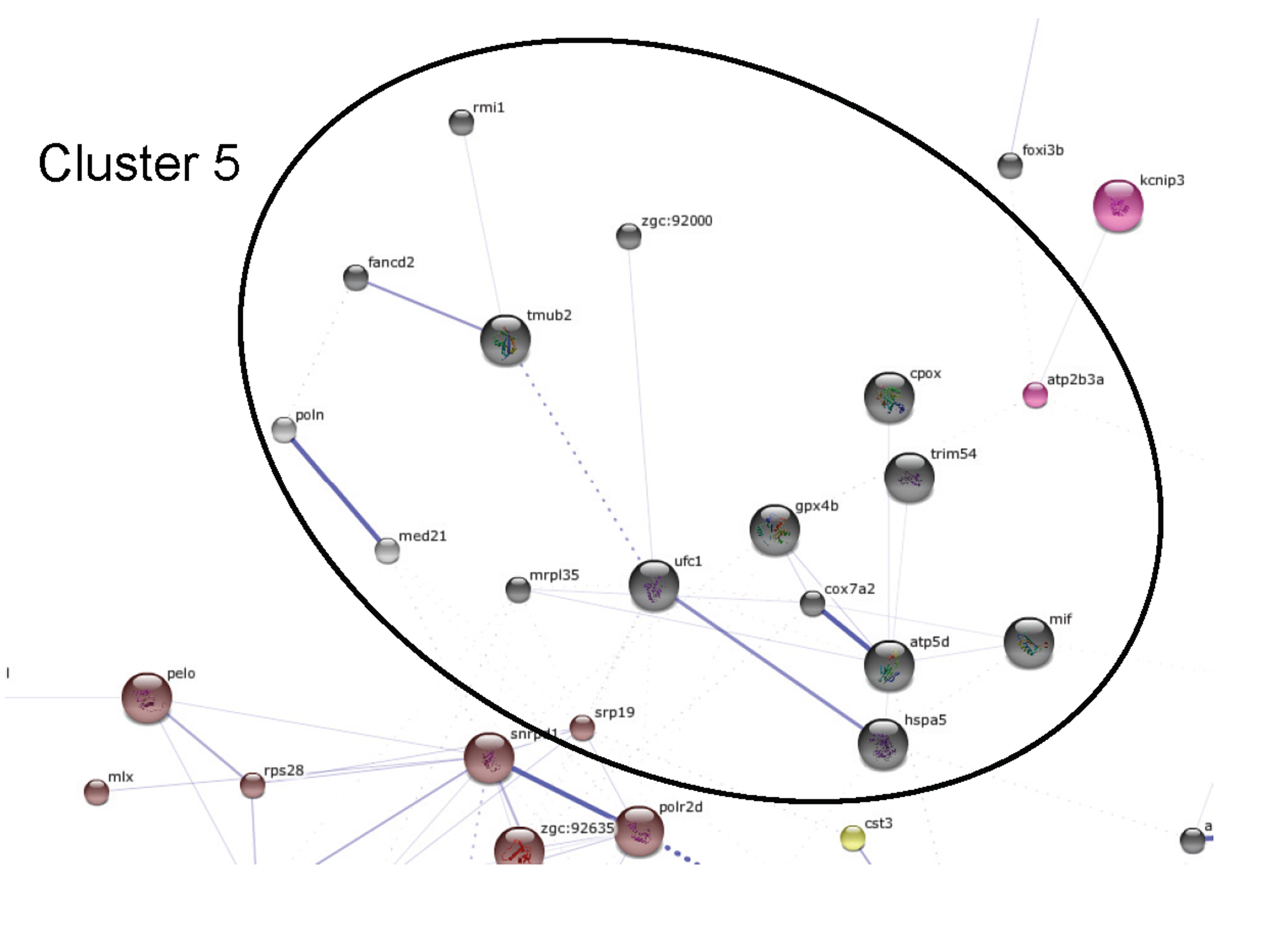

Supplement: Figure S5 — Cluster 5 from gene network interactions using the STRING v.9.1 software. Detail of genes grouped in the cluster 5 (Figure 8) that were expressed in the liver of Symphysodon aequifasciatus exposed to benzo[a]pyrene and phenanthrene for 48 h. (TIF) [file pone.0081083.s005.tif]
