# Supplementary material for: Functional Categorization of Transcriptome in the Species Symphysodon aequifasciatus Pellegrin 1904 (Perciformes: Cichlidae) Exposed to Benzo[a]pyrene and Phenanthrene
Source: PLoS One. 2013 Dec 3;8(12):e81083. doi: 10.1371/journal.pone.0081083 (PMC3849039; doi:10.1371/journal.pone.0081083)
Supplement: Table S1 — Down-regulated genes in Symphysodon aequifasciatus exposed to benzo[a]pyrene and phenanthrene for 48 h. Only genes for which information regarding gene function is currently available in the Gene Ontology (AmiGO v. 1.8) are reported here. Where multiple terms have been identified for a gene, only those most relevant in the context of this study are reported. Genes are ranked according to Fold Change compared to control group (Log2FC). gene_id, gene_name and symbol are according to GenBank (National Center for Biotechnology Information). (BP = Biological Process; CC = Cellular Component; MF = Molecular Function). (DOC) [file pone.0081083.s006.doc]

Table S1. Down-regulated genes in *Symphysodon aequifasciatus* exposed to benzo[a]pyrene and phenanthrene for 48h. Only genes for which information regarding gene function is currently available in the Gene Ontology (AmiGO v. 1.8) are reported here. Where multiple terms have been identified for a gene, only those most relevant in the context of this study are reported. Genes are ranked according to Fold Change compared to control group (Log_2_FC). gene_id, gene_name and symbol are according to GenBank (National Center for Biotechnology Information). (BP=Biological Process; CC=Cellular Component; MF=Molecular Function).

| gene_id | go_id | Gene Ontology term | Ontology | gene_name | symbol | Log_2_FC | PValue |
| --- | --- | --- | --- | --- | --- | --- | --- |
| 751630 | GO:0007264 | small GTPase mediated signal transduction | BP | zgc:153713 | zgc:153713 | -3.58968621 | 7.21848E-07 |
| 751630 | GO:0005622 | intracellular | CC | zgc:153713 | zgc:153713 | -3.58968621 | 7.21848E-07 |
| 751630 | GO:0016020 | membrane | CC | zgc:153713 | zgc:153713 | -3.58968621 | 7.21848E-07 |
| 751630 | GO:0005525 | GTP binding | MF | zgc:153713 | zgc:153713 | -3.58968621 | 7.21848E-07 |
| 751630 | GO:0000166 | nucleotide binding | MF | zgc:153713 | zgc:153713 | -3.58968621 | 7.21848E-07 |
| 560032 | GO:006007 | canonical Wnt receptor signaling pathway | BP | dishevelled, dsh homolog 1b (Drosophila) | dvl1b | -3.500498764 | 1.21097E-06 |
| 560032 | GO:0035556 | intracellular signal transduction | BP | dishevelled, dsh homolog 1b (Drosophila) | dvl1b | -3.500498764 | 1.21097E-06 |
| 560032 | GO:0045893 | positive regulation of transcription, DNA-dependent | BP | dishevelled, dsh homolog 1b (Drosophila) | dvl1b | -3.500498764 | 1.21097E-06 |
| 560032 | GO:0023051 | regulation of signaling | BP | dishevelled, dsh homolog 1b (Drosophila) | dvl1b | -3.500498764 | 1.21097E-06 |
| 560032 | GO:0005938 | cell cortex | CC | dishevelled, dsh homolog 1b (Drosophila) | dvl1b | -3.500498764 | 1.21097E-06 |
| 560032 | GO:0030136 | clathrin-coated vesicle | CC | dishevelled, dsh homolog 1b (Drosophila) | dvl1b | -3.500498764 | 1.21097E-06 |
| 560032 | GO:0005622 | intracellular | CC | dishevelled, dsh homolog 1b (Drosophila) | dvl1b | -3.500498764 | 1.21097E-06 |
| 560032 | GO:0005874 | microtubule | CC | dishevelled, dsh homolog 1b (Drosophila) | dvl1b | -3.500498764 | 1.21097E-06 |
| 560032 | GO:0005109 | frizzled binding | MF | dishevelled, dsh homolog 1b (Drosophila) | dvl1b | -3.500498764 | 1.21097E-06 |
| 560032 | GO:0019901 | protein kinase binding | MF | dishevelled, dsh homolog 1b (Drosophila) | dvl1b | -3.500498764 | 1.21097E-06 |
| 560032 | GO:0048365 | Rac GTPase binding | MF | dishevelled, dsh homolog 1b (Drosophila) | dvl1b | -3.500498764 | 1.21097E-06 |
| 560032 | GO:0004871 | signal transducer activity | MF | dishevelled, dsh homolog 1b (Drosophila) | dvl1b | -3.500498764 | 1.21097E-06 |
| 567402 | GO:0016459 | myosin complex | CC | myosin VB | myo5b | -3.42455941 | 1.81531E-06 |
| 567402 | GO:0005524 | ATP binding | MF | myosin VB | myo5b | -3.42455941 | 1.81531E-06 |
| 567402 | GO:0003774 | motor activity | MF | myosin VB | myo5b | -3.42455941 | 1.81531E-06 |
| 567402 | GO:0000166 | nucleotide binding | MF | myosin VB | myo5b | -3.42455941 | 1.81531E-06 |
| 436713 | GO:0006511 | ubiquitin-dependent protein catabolic process | BP | transcription elongation factor B (SIII), polypeptide 1b | tceb1b | -3.258033713 | 5.8626E-06 |
| 436713 | GO:0005575 | cellular_component | CC | transcription elongation factor B (SIII), polypeptide 1b | tceb1b | -3.258033713 | 5.8626E-06 |
| 562569 | GO:0035556 | intracellular signal transduction | BP | phospholipase C, delta 3b | plcd3b | -3.187504357 | 7.25548E-06 |
| 562569 | GO:0006629 | lipid metabolic process | BP | phospholipase C, delta 3b | plcd3b | -3.187504357 | 7.25548E-06 |
| 562569 | GO:0007165 | signal transduction | BP | phospholipase C, delta 3b | plcd3b | -3.187504357 | 7.25548E-06 |
| 562569 | GO:0005509 | calcium ion binding | MF | phospholipase C, delta 3b | plcd3b | -3.187504357 | 7.25548E-06 |
| 562569 | GO:0004435 | phosphatidylinositol phospholipase C activity | MF | phospholipase C, delta 3b | plcd3b | -3.187504357 | 7.25548E-06 |
| 562569 | GO:0005543 | phospholipid binding | MF | phospholipase C, delta 3b | plcd3b | -3.187504357 | 7.25548E-06 |
| 562569 | GO:0008081 | phosphoric diester hydrolase activity | MF | phospholipase C, delta 3b | plcd3b | -3.187504357 | 7.25548E-06 |
| 562569 | GO:0004871 | signal transducer activity | MF | phospholipase C, delta 3b | plcd3b | -3.187504357 | 7.25548E-06 |
| 100191016 | GO:0008150 | biological_process | BP | zgc:174690 | zgc:174690 | -3.087934919 | 1.51694E-05 |
| 100191016 | GO:0005622 | intracellular | CC | zgc:174690 | zgc:174690 | -3.087934919 | 1.51694E-05 |
| 100191016 | GO:0003676 | nucleic acid binding | MF | zgc:174690 | zgc:174690 | -3.087934919 | 1.51694E-05 |
| 100191016 | GO:0008270 | zinc ion binding | MF | zgc:174690 | zgc:174690 | -3.087934919 | 1.51694E-05 |
| 30493 | GO:0045892 | negative regulation of transcription, DNA-dependent | BP | inhibitor of DNA binding 1 | id1 | -2.966965718 | 2.61433E-05 |
| 30493 | GO:0005737 | cytoplasm | CC | inhibitor of DNA binding 1 | id1 | -2.966965718 | 2.61433E-05 |
| 30493 | GO:0005634 | nucleus | CC | inhibitor of DNA binding 1 | id1 | -2.966965718 | 2.61433E-05 |
| 30493 | GO:0046983 | protein dimerization activity | MF | inhibitor of DNA binding 1 | id1 | -2.966965718 | 2.61433E-05 |
| 100037349 | GO:0006633 | fatty acid biosynthetic process | BP | malonyl-CoA decarboxylase | mlycd | -2.949973532 | 3.19565E-05 |
| 100037349 | GO:0005575 | cellular_component | CC | malonyl-CoA decarboxylase | mlycd | -2.949973532 | 3.19565E-05 |
| 100037349 | GO:0050080 | malonyl-CoA decarboxylase activity | MF | malonyl-CoA decarboxylase | mlycd | -2.949973532 | 3.19565E-05 |
| 100318165 | GO:0016021 | integral to membrane | CC | epithelial membrane protein 1 | emp1 | -2.776197535 | 8.06508E-05 |
| 100318165 | GO:0016020 | membrane | CC | epithelial membrane protein 1 | emp1 | -2.776197535 | 8.06508E-05 |
| 406569 | GO:0043484 | regulation of RNA splicing | BP | RNA binding protein, fox-1 homolog (C. elegans) 2 | rbfox2 | -2.748115052 | 8.13252E-05 |
| 406569 | GO:0003676 | nucleic acid binding | MF | RNA binding protein, fox-1 homolog (C. elegans) 2 | rbfox2 | -2.748115052 | 8.13252E-05 |
| 406569 | GO:0000166 | nucleotide binding | MF | RNA binding protein, fox-1 homolog (C. elegans) 2 | rbfox2 | -2.748115052 | 8.13252E-05 |
| 569245 | GO:0055114 | oxidation-reduction process | BP | cytochrome P450, family 2, subfamily AD, polypeptide 3 | cyp2ad3 | -2.712201513 | 0.000101308 |
| 569245 | GO:0009055 | electron carrier activity | MF | cytochrome P450, family 2, subfamily AD, polypeptide 3 | cyp2ad3 | -2.712201513 | 0.000101308 |
| 569245 | GO:0020037 | heme binding | MF | cytochrome P450, family 2, subfamily AD, polypeptide 3 | cyp2ad3 | -2.712201513 | 0.000101308 |
| 569245 | GO:0005506 | iron ion binding | MF | cytochrome P450, family 2, subfamily AD, polypeptide 3 | cyp2ad3 | -2.712201513 | 0.000101308 |
| 569245 | GO:0046872 | metal ion binding | MF | cytochrome P450, family 2, subfamily AD, polypeptide 3 | cyp2ad3 | -2.712201513 | 0.000101308 |
| 569245 | GO:0004497 | monooxygenase activity | MF | cytochrome P450, family 2, subfamily AD, polypeptide 3 | cyp2ad3 | -2.712201513 | 0.000101308 |
| 569245 | GO:0016491 | oxidoreductase activity | MF | cytochrome P450, family 2, subfamily AD, polypeptide 3 | cyp2ad3 | -2.712201513 | 0.000101308 |
| 569245 | GO:0016705 | oxidoreductase activity, acting on paired donors, with incorporation or reduction of molecular oxygen | MF | cytochrome P450, family 2, subfamily AD, polypeptide 3 | cyp2ad3 | -2.712201513 | 0.000101308 |
| 569245 | GO:0016712 | oxidoreductase activity, acting on paired donors, with incorporation or reduction of molecular oxygen, reduced flavin or flavoprotein as one donor, and incorporation of one atom of oxygen | MF | cytochrome P450, family 2, subfamily AD, polypeptide 3 | cyp2ad3 | -2.712201513 | 0.000101308 |
| 325772 | GO:0032313 | regulation of Rab GTPase activity | BP | si:ch211-87l2.1 | si:ch211-87l2.1 | -2.67661556 | 0.000227437 |
| 325772 | GO:0005622 | intracellular | CC | si:ch211-87l2.1 | si:ch211-87l2.1 | -2.67661556 | 0.000227437 |
| 325772 | GO:0005097 | Rab GTPase activator activity | MF | si:ch211-87l2.1 | si:ch211-87l2.1 | -2.67661556 | 0.000227437 |
| 794293 | GO:0006355 | regulation of transcription, DNA-dependent | BP | zgc:171784 | zgc:171784 | -2.617099157 | 0.000162254 |
| 794293 | GO:0005634 | nucleus | CC | zgc:171784 | zgc:171784 | -2.617099157 | 0.000162254 |
| 794293 | GO:0003700 | sequence-specific DNA binding transcription factor activity | MF | zgc:171784 | zgc:171784 | -2.617099157 | 0.000162254 |
| 561775 | GO:0007049 | cell cycle | BP | sperm antigen with calponin homology and coiled-coil domains 1-like a | specc1la | -2.613545148 | 0.000163649 |
| 561775 | GO:0051301 | cell division | BP | sperm antigen with calponin homology and coiled-coil domains 1-like a | specc1la | -2.613545148 | 0.000163649 |
| 561775 | GO:0030054 | cell junction | CC | sperm antigen with calponin homology and coiled-coil domains 1-like a | specc1la | -2.613545148 | 0.000163649 |
| 561775 | GO:0005737 | cytoplasm | CC | sperm antigen with calponin homology and coiled-coil domains 1-like a | specc1la | -2.613545148 | 0.000163649 |
| 561775 | GO:0005856 | cytoskeleton | CC | sperm antigen with calponin homology and coiled-coil domains 1-like a | specc1la | -2.613545148 | 0.000163649 |
| 561775 | GO:0005921 | gap junction | CC | sperm antigen with calponin homology and coiled-coil domains 1-like a | specc1la | -2.613545148 | 0.000163649 |
| 561775 | GO:0005819 | spindle | CC | sperm antigen with calponin homology and coiled-coil domains 1-like a | specc1la | -2.613545148 | 0.000163649 |
| 561775 | GO:0003674 | molecular_function | MF | sperm antigen with calponin homology and coiled-coil domains 1-like a | specc1la | -2.613545148 | 0.000163649 |
| 326793 | GO:0008150 | biological_process | BP | leucine rich repeat containing 42 | lrrc42 | -2.605949608 | 0.000175636 |
| 780842 | GO:0008152 | metabolic process | BP | zgc:158321 | zgc:158321 | -2.539778032 | 0.000250121 |
| 780842 | GO:0005739 | mitochondrion | CC | zgc:158321 | zgc:158321 | -2.539778032 | 0.000250121 |
| 780842 | GO:0003824 | catalytic activity | MF | zgc:158321 | zgc:158321 | -2.539778032 | 0.000250121 |
| 492631 | GO:0008150 | biological_process | BP | zgc:193538 | zgc:193538 | -2.497636043 | 0.000316709 |
| 492631 | GO:0005575 | cellular_component | CC | zgc:193538 | zgc:193538 | -2.497636043 | 0.000316709 |
| 492631 | GO:0003674 | molecular_function | MF | zgc:193538 | zgc:193538 | -2.497636043 | 0.000316709 |
| 402815 | GO:0006355 | regulation of transcription, DNA-dependent | BP | nuclear receptor subfamily 1, group D, member 2a | nr1d2a | -2.494991106 | 0.000315317 |
| 402815 | GO:0043401 | steroid hormone mediated signaling pathway | BP | nuclear receptor subfamily 1, group D, member 2a | nr1d2a | -2.494991106 | 0.000315317 |
| 402815 | GO:0006351 | transcription, DNA-dependent | BP | nuclear receptor subfamily 1, group D, member 2a | nr1d2a | -2.494991106 | 0.000315317 |
| 402815 | GO:0005634 | nucleus | CC | nuclear receptor subfamily 1, group D, member 2a | nr1d2a | -2.494991106 | 0.000315317 |
| 402815 | GO:0003677 | DNA binding | MF | nuclear receptor subfamily 1, group D, member 2a | nr1d2a | -2.494991106 | 0.000315317 |
| 402815 | GO:0046872 | metal ion binding | MF | nuclear receptor subfamily 1, group D, member 2a | nr1d2a | -2.494991106 | 0.000315317 |
| 402815 | GO:0043565 | sequence-specific DNA binding | MF | nuclear receptor subfamily 1, group D, member 2a | nr1d2a | -2.494991106 | 0.000315317 |
| 402815 | GO:0003700 | sequence-specific DNA binding transcription factor activity | MF | nuclear receptor subfamily 1, group D, member 2a | nr1d2a | -2.494991106 | 0.000315317 |
| 402815 | GO:0003707 | steroid hormone receptor activity | MF | nuclear receptor subfamily 1, group D, member 2a | nr1d2a | -2.494991106 | 0.000315317 |
| 402815 | GO:0004887 | thyroid hormone receptor activity | MF | nuclear receptor subfamily 1, group D, member 2a | nr1d2a | -2.494991106 | 0.000315317 |
| 402815 | GO:0008270 | zinc ion binding | MF | nuclear receptor subfamily 1, group D, member 2a | nr1d2a | -2.494991106 | 0.000315317 |
| 557566 | GO:0006508 | proteolysis | BP | si:ch211-212d10.1 | si:ch211-212d10.1 | -2.465918385 | 0.00037286 |
| 557566 | GO:0003824 | catalytic activity | MF | si:ch211-212d10.1 | si:ch211-212d10.1 | -2.465918385 | 0.00037286 |
| 557566 | GO:0004252 | serine-type endopeptidase activity | MF | si:ch211-212d10.1 | si:ch211-212d10.1 | -2.465918385 | 0.00037286 |
| 559852 | GO:0016021 | integral to membrane | CC | si:ch211-106h4.6 | si:ch211-106h4.6 | -2.458416911 | 0.000390182 |
| 559852 | GO:0016020 | membrane | CC | si:ch211-106h4.6 | si:ch211-106h4.6 | -2.458416911 | 0.000390182 |
| 559852 | GO:0004872 | receptor activity | MF | si:ch211-106h4.6 | si:ch211-106h4.6 | -2.458416911 | 0.000390182 |
| 557342 | GO:0008150 | biological_process | BP | si:dkey-86e18.1 | si:dkey-86e18.1 | -2.4460348 | 0.000397915 |
| 557342 | GO:0005575 | cellular_component | CC | si:dkey-86e18.1 | si:dkey-86e18.1 | -2.4460348 | 0.000397915 |
| 557342 | GO:0003674 | molecular_function | MF | si:dkey-86e18.1 | si:dkey-86e18.1 | -2.4460348 | 0.000397915 |
| 556936 | GO:0008150 | biological_process | BP | si:dkeyp-81f3.1 | si:dkeyp-81f3.1 | -2.427720853 | 0.000437655 |
| 556936 | GO:0005575 | cellular_component | CC | si:dkeyp-81f3.1 | si:dkeyp-81f3.1 | -2.427720853 | 0.000437655 |
| 556936 | GO:0003674 | molecular_function | MF | si:dkeyp-81f3.1 | si:dkeyp-81f3.1 | -2.427720853 | 0.000437655 |
| 387258 | GO:0051090 | regulation of sequence-specific DNA binding transcription factor activity | BP | forkhead box I3b | foxi3b | -2.400911355 | 0.000505392 |
| 387258 | GO:0006357 | regulation of transcription from RNA polymerase II promoter | BP | forkhead box I3b | foxi3b | -2.400911355 | 0.000505392 |
| 387258 | GO:0006355 | regulation of transcription, DNA-dependent | BP | forkhead box I3b | foxi3b | -2.400911355 | 0.000505392 |
| 387258 | GO:0006351 | transcription, DNA-dependent | BP | forkhead box I3b | foxi3b | -2.400911355 | 0.000505392 |
| 387258 | GO:0005634 | nucleus | CC | forkhead box I3b | foxi3b | -2.400911355 | 0.000505392 |
| 387258 | GO:0005667 | transcription factor complex | CC | forkhead box I3b | foxi3b | -2.400911355 | 0.000505392 |
| 387258 | GO:0003677 | DNA binding | MF | forkhead box I3b | foxi3b | -2.400911355 | 0.000505392 |
| 387258 | GO:0008301 | DNA binding, bending | MF | forkhead box I3b | foxi3b | -2.400911355 | 0.000505392 |
| 387258 | GO:0003690 | double-stranded DNA binding | MF | forkhead box I3b | foxi3b | -2.400911355 | 0.000505392 |
| 387258 | GO:0003705 | RNA polymerase II distal enhancer sequence-specific DNA binding transcription factor activity | MF | forkhead box I3b | foxi3b | -2.400911355 | 0.000505392 |
| 387258 | GO:0043565 | sequence-specific DNA binding | MF | forkhead box I3b | foxi3b | -2.400911355 | 0.000505392 |
| 387258 | GO:0003700 | sequence-specific DNA binding transcription factor activity | MF | forkhead box I3b | foxi3b | -2.400911355 | 0.000505392 |
| 387258 | GO:0008134 | transcription factor binding | MF | forkhead box I3b | foxi3b | -2.400911355 | 0.000505392 |
| 399484 | GO:0006413 | translational initiation | BP | eukaryotic translation initiation factor 4A, isoform 1B | eif4a1b | -2.397151161 | 0.000505297 |
| 399484 | GO:0005524 | ATP binding | MF | eukaryotic translation initiation factor 4A, isoform 1B | eif4a1b | -2.397151161 | 0.000505297 |
| 399484 | GO:0008026 | ATP-dependent helicase activity | MF | eukaryotic translation initiation factor 4A, isoform 1B | eif4a1b | -2.397151161 | 0.000505297 |
| 399484 | GO:0004386 | helicase activity | MF | eukaryotic translation initiation factor 4A, isoform 1B | eif4a1b | -2.397151161 | 0.000505297 |
| 399484 | GO:0016787 | hydrolase activity | MF | eukaryotic translation initiation factor 4A, isoform 1B | eif4a1b | -2.397151161 | 0.000505297 |
| 399484 | GO:0003676 | nucleic acid binding | MF | eukaryotic translation initiation factor 4A, isoform 1B | eif4a1b | -2.397151161 | 0.000505297 |
| 399484 | GO:0000166 | nucleotide binding | MF | eukaryotic translation initiation factor 4A, isoform 1B | eif4a1b | -2.397151161 | 0.000505297 |
| 399484 | GO:0003743 | translation initiation factor activity | MF | eukaryotic translation initiation factor 4A, isoform 1B | eif4a1b | -2.397151161 | 0.000505297 |
| 378962 | GO:0009072 | aromatic amino acid family metabolic process | BP | phenylalanine hydroxylase | pah | -2.395474429 | 0.000494655 |
| 378962 | GO:0006559 | L-phenylalanine catabolic process | BP | phenylalanine hydroxylase | pah | -2.395474429 | 0.000494655 |
| 378962 | GO:0008152 | metabolic process | BP | phenylalanine hydroxylase | pah | -2.395474429 | 0.000494655 |
| 378962 | GO:0055114 | oxidation-reduction process | BP | phenylalanine hydroxylase | pah | -2.395474429 | 0.000494655 |
| 378962 | GO:0042221 | response to chemical stimulus | BP | phenylalanine hydroxylase | pah | -2.395474429 | 0.000494655 |
| 378962 | GO:0016597 | amino acid binding | MF | phenylalanine hydroxylase | pah | -2.395474429 | 0.000494655 |
| 378962 | GO:0005506 | iron ion binding | MF | phenylalanine hydroxylase | pah | -2.395474429 | 0.000494655 |
| 378962 | GO:0004497 | monooxygenase activity | MF | phenylalanine hydroxylase | pah | -2.395474429 | 0.000494655 |
| 378962 | GO:0016714 | oxidoreductase activity, acting on paired donors, with incorporation or reduction of molecular oxygen, reduced pteridine as one donor, and incorporation of one atom of oxygen | MF | phenylalanine hydroxylase | pah | -2.395474429 | 0.000494655 |
| 378962 | GO:0004505 | phenylalanine 4-monooxygenase activity | MF | phenylalanine hydroxylase | pah | -2.395474429 | 0.000494655 |
| 553163 | GO:0005576 | extracellular region | CC | isthmin 2 | ism2 | -2.379711298 | 0.000541198 |
| 554103 | GO:0005746 | mitochondrial respiratory chain | CC | cytochrome c oxidase, subunit VIIa 2 | cox7a2 | -2.35974239 | 0.000619449 |
| 554103 | GO:0004129 | cytochrome-c oxidase activity | MF | cytochrome c oxidase, subunit VIIa 2 | cox7a2 | -2.35974239 | 0.000619449 |
| 554103 | GO:0009055 | electron carrier activity | MF | cytochrome c oxidase, subunit VIIa 2 | cox7a2 | -2.35974239 | 0.000619449 |
| 567275 | GO:0006750 | glutathione biosynthetic process | BP | zgc:158387 | zgc:158387 | -2.355319693 | 0.000619986 |
| 567275 | GO:0005783 | endoplasmic reticulum | CC | zgc:158387 | zgc:158387 | -2.355319693 | 0.000619986 |
| 567275 | GO:0005635 | nuclear envelope | CC | zgc:158387 | zgc:158387 | -2.355319693 | 0.000619986 |
| 567275 | GO:0004602 | glutathione peroxidase activity | MF | zgc:158387 | zgc:158387 | -2.355319693 | 0.000619986 |
| 567275 | GO:0004364 | glutathione transferase activity | MF | zgc:158387 | zgc:158387 | -2.355319693 | 0.000619986 |
| 567275 | GO:0016740 | transferase activity | MF | zgc:158387 | zgc:158387 | -2.355319693 | 0.000619986 |
| 559099 | GO:0006508 | proteolysis | BP | ubiquitin specific peptidase 30 | usp30 | -2.340089339 | 0.000763363 |
| 559099 | GO:0006511 | ubiquitin-dependent protein catabolic process | BP | ubiquitin specific peptidase 30 | usp30 | -2.340089339 | 0.000763363 |
| 559099 | GO:0016021 | integral to membrane | CC | ubiquitin specific peptidase 30 | usp30 | -2.340089339 | 0.000763363 |
| 559099 | GO:0016020 | membrane | CC | ubiquitin specific peptidase 30 | usp30 | -2.340089339 | 0.000763363 |
| 559099 | GO:0005741 | mitochondrial outer membrane | CC | ubiquitin specific peptidase 30 | usp30 | -2.340089339 | 0.000763363 |
| 559099 | GO:0005739 | mitochondrion | CC | ubiquitin specific peptidase 30 | usp30 | -2.340089339 | 0.000763363 |
| 559099 | GO:0008234 | cysteine-type peptidase activity | MF | ubiquitin specific peptidase 30 | usp30 | -2.340089339 | 0.000763363 |
| 559099 | GO:0016787 | hydrolase activity | MF | ubiquitin specific peptidase 30 | usp30 | -2.340089339 | 0.000763363 |
| 559099 | GO:0008233 | peptidase activity | MF | ubiquitin specific peptidase 30 | usp30 | -2.340089339 | 0.000763363 |
| 559099 | GO:0004221 | ubiquitin thiolesterase activity | MF | ubiquitin specific peptidase 30 | usp30 | -2.340089339 | 0.000763363 |
| 555814 | GO:0008150 | biological_process | BP | zgc:174354 | zgc:174354 | -2.303194 | 0.000837791 |
| 555814 | GO:0005634 | nucleus | CC | zgc:174354 | zgc:174354 | -2.303194 | 0.000837791 |
| 555814 | GO:0008270 | zinc ion binding | MF | zgc:174354 | zgc:174354 | -2.303194 | 0.000837791 |
| 557073 | GO:0003674 | molecular_function | MF | zgc:165666 | zgc:165666 | -2.299516466 | 0.000801076 |
| 550373 | GO:0006457 | protein folding | BP | peptidylprolyl isomerase E (cyclophilin E) | ppie | -2.277954528 | 0.000961344 |
| 550373 | GO:0016853 | isomerase activity | MF | peptidylprolyl isomerase E (cyclophilin E) | ppie | -2.277954528 | 0.000961344 |
| 550373 | GO:0003676 | nucleic acid binding | MF | peptidylprolyl isomerase E (cyclophilin E) | ppie | -2.277954528 | 0.000961344 |
| 550373 | GO:0000166 | nucleotide binding | MF | peptidylprolyl isomerase E (cyclophilin E) | ppie | -2.277954528 | 0.000961344 |
| 550373 | GO:0003755 | peptidyl-prolyl cis-trans isomerase activity | MF | peptidylprolyl isomerase E (cyclophilin E) | ppie | -2.277954528 | 0.000961344 |
| 550373 | GO:0003723 | RNA binding | MF | peptidylprolyl isomerase E (cyclophilin E) | ppie | -2.277954528 | 0.000961344 |
| 450016 | GO:0009058 | biosynthetic process | BP | phosphate cytidylyltransferase 2, ethanolamine | pcyt2 | -2.264472301 | 0.000961276 |
| 450016 | GO:0005575 | cellular_component | CC | phosphate cytidylyltransferase 2, ethanolamine | pcyt2 | -2.264472301 | 0.000961276 |
| 450016 | GO:0003824 | catalytic activity | MF | phosphate cytidylyltransferase 2, ethanolamine | pcyt2 | -2.264472301 | 0.000961276 |
| 450016 | GO:0016779 | nucleotidyltransferase activity | MF | phosphate cytidylyltransferase 2, ethanolamine | pcyt2 | -2.264472301 | 0.000961276 |
| 450016 | GO:0016740 | transferase activity | MF | phosphate cytidylyltransferase 2, ethanolamine | pcyt2 | -2.264472301 | 0.000961276 |
| 30304 | GO:0009897 | external side of plasma membrane | CC | one-eyed pinhead | oep | -2.254511269 | 0.001008343 |
| 492514 | GO:0008152 | metabolic process | BP | DPH5 homolog (S. cerevisiae) | dph5 | -2.20168591 | 0.002250088 |
| 492514 | GO:0017183 | peptidyl-diphthamide biosynthetic process from peptidyl-histidine | BP | DPH5 homolog (S. cerevisiae) | dph5 | -2.20168591 | 0.002250088 |
| 492514 | GO:0005575 | cellular_component | CC | DPH5 homolog (S. cerevisiae) | dph5 | -2.20168591 | 0.002250088 |
| 492514 | GO:0004164 | diphthine synthase activity | MF | DPH5 homolog (S. cerevisiae) | dph5 | -2.20168591 | 0.002250088 |
| 492514 | GO:0008168 | methyltransferase activity | MF | DPH5 homolog (S. cerevisiae) | dph5 | -2.20168591 | 0.002250088 |
| 641290 | GO:0008150 | biological_process | BP | nucleotide binding protein 2 (MinD homolog, E. coli) | nubp2 | -2.175793685 | 0.00152526 |
| 641290 | GO:0005575 | cellular_component | CC | nucleotide binding protein 2 (MinD homolog, E. coli) | nubp2 | -2.175793685 | 0.00152526 |
| 641290 | GO:0051539 | 4 iron, 4 sulfur cluster binding | MF | nucleotide binding protein 2 (MinD homolog, E. coli) | nubp2 | -2.175793685 | 0.00152526 |
| 641290 | GO:0005524 | ATP binding | MF | nucleotide binding protein 2 (MinD homolog, E. coli) | nubp2 | -2.175793685 | 0.00152526 |
| 641290 | GO:0051536 | iron-sulfur cluster binding | MF | nucleotide binding protein 2 (MinD homolog, E. coli) | nubp2 | -2.175793685 | 0.00152526 |
| 641290 | GO:0046872 | metal ion binding | MF | nucleotide binding protein 2 (MinD homolog, E. coli) | nubp2 | -2.175793685 | 0.00152526 |
| 641290 | GO:0000166 | nucleotide binding | MF | nucleotide binding protein 2 (MinD homolog, E. coli) | nubp2 | -2.175793685 | 0.00152526 |
| 560060 | GO:0030036 | actin cytoskeleton organization | BP | dishevelled associated activator of morphogenesis 1b | daam1b | -2.137241796 | 0.001802089 |
| 560060 | GO:0016043 | cellular component organization | BP | dishevelled associated activator of morphogenesis 1b | daam1b | -2.137241796 | 0.001802089 |
| 560060 | GO:0003779 | actin binding | MF | dishevelled associated activator of morphogenesis 1b | daam1b | -2.137241796 | 0.001802089 |
| 560060 | GO:0017048 | Rho GTPase binding | MF | dishevelled associated activator of morphogenesis 1b | daam1b | -2.137241796 | 0.001802089 |
| 393424 | GO:0005575 | cellular_component | CC | DnaJ (Hsp40) homolog, subfamily C, member 24 | dnajc24 | -2.071917758 | 0.002309874 |
| 445080 | GO:0035556 | intracellular signal transduction | BP | splA/ryanodine receptor domain and SOCS box containing 4b | spsb4b | -2.069597281 | 0.002419374 |
| 796968 | GO:0005622 | intracellular | CC | si:ch73-299h12.4 | si:ch73-299h12.4 | -2.068366649 | 0.002420094 |
| 796968 | GO:0003676 | nucleic acid binding | MF | si:ch73-299h12.4 | si:ch73-299h12.4 | -2.068366649 | 0.002420094 |
| 796968 | GO:0008270 | zinc ion binding | MF | si:ch73-299h12.4 | si:ch73-299h12.4 | -2.068366649 | 0.002420094 |
| 393149 | GO:0006260 | DNA replication | BP | RMI1, RecQ mediated genome instability 1, homolog (S. cerevisiae) | rmi1 | -2.047265279 | 0.002656542 |
| 393149 | GO:0005634 | nucleus | CC | RMI1, RecQ mediated genome instability 1, homolog (S. cerevisiae) | rmi1 | -2.047265279 | 0.002656542 |
| 393149 | GO:0003674 | molecular_function | MF | RMI1, RecQ mediated genome instability 1, homolog (S. cerevisiae) | rmi1 | -2.047265279 | 0.002656542 |
| 492758 | GO:0005575 | cellular_component | CC | fibroblast growth factor 13a | fgf13a | -2.035472851 | 0.002787894 |
| 492758 | GO:0008083 | growth factor activity | MF | fibroblast growth factor 13a | fgf13a | -2.035472851 | 0.002787894 |
| 30698 | GO:0006355 | regulation of transcription, DNA-dependent | BP | NK2 transcription factor related 3 | nkx2.3 | -2.0245962 | 0.003002457 |
| 30698 | GO:0005634 | nucleus | CC | NK2 transcription factor related 3 | nkx2.3 | -2.0245962 | 0.003002457 |
| 30698 | GO:0003677 | DNA binding | MF | NK2 transcription factor related 3 | nkx2.3 | -2.0245962 | 0.003002457 |
| 30698 | GO:0043565 | sequence-specific DNA binding | MF | NK2 transcription factor related 3 | nkx2.3 | -2.0245962 | 0.003002457 |
| 30698 | GO:0003700 | sequence-specific DNA binding transcription factor activity | MF | NK2 transcription factor related 3 | nkx2.3 | -2.0245962 | 0.003002457 |
| 561051 | GO:0008150 | biological_process | BP | zgc:153980 | zgc:153980 | -2.00236313 | 0.003212985 |
| 561051 | GO:0042995 | cell projection | CC | zgc:153980 | zgc:153980 | -2.00236313 | 0.003212985 |
| 561051 | GO:0005737 | cytoplasm | CC | zgc:153980 | zgc:153980 | -2.00236313 | 0.003212985 |
| 561051 | GO:0016021 | integral to membrane | CC | zgc:153980 | zgc:153980 | -2.00236313 | 0.003212985 |
| 561051 | GO:0016020 | membrane | CC | zgc:153980 | zgc:153980 | -2.00236313 | 0.003212985 |
| 561051 | GO:0048471 | perinuclear region of cytoplasm | CC | zgc:153980 | zgc:153980 | -2.00236313 | 0.003212985 |
| 561051 | GO:0005886 | plasma membrane | CC | zgc:153980 | zgc:153980 | -2.00236313 | 0.003212985 |
| 561051 | GO:0003674 | molecular_function | MF | zgc:153980 | zgc:153980 | -2.00236313 | 0.003212985 |
| 327167 | GO:0006355 | regulation of transcription, DNA-dependent | BP | myoneurin | mynn | -2.000758017 | 0.003262739 |
| 327167 | GO:0006351 | transcription, DNA-dependent | BP | myoneurin | mynn | -2.000758017 | 0.003262739 |
| 327167 | GO:0005622 | intracellular | CC | myoneurin | mynn | -2.000758017 | 0.003262739 |
| 327167 | GO:0005634 | nucleus | CC | myoneurin | mynn | -2.000758017 | 0.003262739 |
| 327167 | GO:0003677 | DNA binding | MF | myoneurin | mynn | -2.000758017 | 0.003262739 |
| 327167 | GO:0046872 | metal ion binding | MF | myoneurin | mynn | -2.000758017 | 0.003262739 |
| 327167 | GO:0003676 | nucleic acid binding | MF | myoneurin | mynn | -2.000758017 | 0.003262739 |
| 327167 | GO:0008270 | zinc ion binding | MF | myoneurin | mynn | -2.000758017 | 0.003262739 |
| 553630 | GO:0005783 | endoplasmic reticulum | CC | tripartite motif-containing 13 | trim13 | -1.991153597 | 0.003352177 |
| 553630 | GO:0005789 | endoplasmic reticulum membrane | CC | tripartite motif-containing 13 | trim13 | -1.991153597 | 0.003352177 |
| 553630 | GO:0016021 | integral to membrane | CC | tripartite motif-containing 13 | trim13 | -1.991153597 | 0.003352177 |
| 553630 | GO:0005622 | intracellular | CC | tripartite motif-containing 13 | trim13 | -1.991153597 | 0.003352177 |
| 553630 | GO:0016020 | membrane | CC | tripartite motif-containing 13 | trim13 | -1.991153597 | 0.003352177 |
| 553630 | GO:0016874 | ligase activity | MF | tripartite motif-containing 13 | trim13 | -1.991153597 | 0.003352177 |
| 553630 | GO:0046872 | metal ion binding | MF | tripartite motif-containing 13 | trim13 | -1.991153597 | 0.003352177 |
| 553630 | GO:0008270 | zinc ion binding | MF | tripartite motif-containing 13 | trim13 | -1.991153597 | 0.003352177 |
| 556427 | GO:0051056 | regulation of small GTPase mediated signal transduction | BP | Ral GTPase activating protein, alpha subunit 1 (catalytic) | ralgapa1 | -1.98507494 | 0.003383901 |
| 556427 | GO:0005096 | GTPase activator activity | MF | Ral GTPase activating protein, alpha subunit 1 (catalytic) | ralgapa1 | -1.98507494 | 0.003383901 |
| 445125 | GO:0008150 | biological_process | BP | phospholipid transfer protein | pltp | -1.980697067 | 0.003442615 |
| 445125 | GO:0005575 | cellular_component | CC | phospholipid transfer protein | pltp | -1.980697067 | 0.003442615 |
| 445125 | GO:0008289 | lipid binding | MF | phospholipid transfer protein | pltp | -1.980697067 | 0.003442615 |
| 566857 | GO:0006260 | DNA replication | BP | polymerase (DNA directed) nu | poln | -1.942602809 | 0.004109309 |
| 566857 | GO:0003677 | DNA binding | MF | polymerase (DNA directed) nu | poln | -1.942602809 | 0.004109309 |
| 566857 | GO:0003887 | DNA-directed DNA polymerase activity | MF | polymerase (DNA directed) nu | poln | -1.942602809 | 0.004109309 |
| 751735 | GO:0008152 | metabolic process | BP | zgc:153724 | zgc:153724 | -1.902073114 | 0.004947666 |
| 751735 | GO:0005575 | cellular_component | CC | zgc:153724 | zgc:153724 | -1.902073114 | 0.004947666 |
| 751735 | GO:0000166 | nucleotide binding | MF | zgc:153724 | zgc:153724 | -1.902073114 | 0.004947666 |
| 751735 | GO:0016491 | oxidoreductase activity | MF | zgc:153724 | zgc:153724 | -1.902073114 | 0.004947666 |
| 30598 | GO:0006355 | regulation of transcription, DNA-dependent | BP | visual system homeobox 1 homolog, chx10-like | vsx1 | -1.899041458 | 0.005178343 |
| 30598 | GO:0006351 | transcription, DNA-dependent | BP | visual system homeobox 1 homolog, chx10-like | vsx1 | -1.899041458 | 0.005178343 |
| 30598 | GO:0005634 | nucleus | CC | visual system homeobox 1 homolog, chx10-like | vsx1 | -1.899041458 | 0.005178343 |
| 30598 | GO:0003677 | DNA binding | MF | visual system homeobox 1 homolog, chx10-like | vsx1 | -1.899041458 | 0.005178343 |
| 30598 | GO:0043565 | sequence-specific DNA binding | MF | visual system homeobox 1 homolog, chx10-like | vsx1 | -1.899041458 | 0.005178343 |
| 30598 | GO:0003700 | sequence-specific DNA binding transcription factor activity | MF | visual system homeobox 1 homolog, chx10-like | vsx1 | -1.899041458 | 0.005178343 |
| 792024 | GO:0006184 | GTP catabolic process | BP | si:ch73-199e17.1 | si:ch73-199e17.1 | -1.898451464 | 0.005125396 |
| 792024 | GO:0007017 | microtubule-based process | BP | si:ch73-199e17.1 | si:ch73-199e17.1 | -1.898451464 | 0.005125396 |
| 792024 | GO:0051258 | protein polymerization | BP | si:ch73-199e17.1 | si:ch73-199e17.1 | -1.898451464 | 0.005125396 |
| 792024 | GO:0005737 | cytoplasm | CC | si:ch73-199e17.1 | si:ch73-199e17.1 | -1.898451464 | 0.005125396 |
| 792024 | GO:0005856 | cytoskeleton | CC | si:ch73-199e17.1 | si:ch73-199e17.1 | -1.898451464 | 0.005125396 |
| 792024 | GO:0005874 | microtubule | CC | si:ch73-199e17.1 | si:ch73-199e17.1 | -1.898451464 | 0.005125396 |
| 792024 | GO:0043234 | protein complex | CC | si:ch73-199e17.1 | si:ch73-199e17.1 | -1.898451464 | 0.005125396 |
| 792024 | GO:0005525 | GTP binding | MF | si:ch73-199e17.1 | si:ch73-199e17.1 | -1.898451464 | 0.005125396 |
| 792024 | GO:0003924 | GTPase activity | MF | si:ch73-199e17.1 | si:ch73-199e17.1 | -1.898451464 | 0.005125396 |
| 792024 | GO:0000166 | nucleotide binding | MF | si:ch73-199e17.1 | si:ch73-199e17.1 | -1.898451464 | 0.005125396 |
| 792024 | GO:0005200 | structural constituent of cytoskeleton | MF | si:ch73-199e17.1 | si:ch73-199e17.1 | -1.898451464 | 0.005125396 |
| 117234 | GO:0005634 | nucleus | CC | mab-21-like 2 | mab21l2 | -1.892221216 | 0.005245019 |
| 100006671 | GO:0008150 | biological_process | BP | zgc:165653 | zgc:165653 | -1.891569472 | 0.005157297 |
| 100006671 | GO:0005575 | cellular_component | CC | zgc:165653 | zgc:165653 | -1.891569472 | 0.005157297 |
| 100006671 | GO:0003779 | actin binding | MF | zgc:165653 | zgc:165653 | -1.891569472 | 0.005157297 |
| 450076 | GO:0008150 | biological_process | BP | ADP-ribosylation factor-like 6 interacting protein 5a | arl6ip5a | -1.891072165 | 0.005190832 |
| 450076 | GO:0005575 | cellular_component | CC | ADP-ribosylation factor-like 6 interacting protein 5a | arl6ip5a | -1.891072165 | 0.005190832 |
| 450076 | GO:0003674 | molecular_function | MF | ADP-ribosylation factor-like 6 interacting protein 5a | arl6ip5a | -1.891072165 | 0.005190832 |
| 30771 | GO:0005622 | intracellular | CC | hypermethylated in cancer 1 like | hic1l | -1.867159268 | 0.005761444 |
| 30771 | GO:0005634 | nucleus | CC | hypermethylated in cancer 1 like | hic1l | -1.867159268 | 0.005761444 |
| 30771 | GO:0046872 | metal ion binding | MF | hypermethylated in cancer 1 like | hic1l | -1.867159268 | 0.005761444 |
| 30771 | GO:0003676 | nucleic acid binding | MF | hypermethylated in cancer 1 like | hic1l | -1.867159268 | 0.005761444 |
| 30771 | GO:0008270 | zinc ion binding | MF | hypermethylated in cancer 1 like | hic1l | -1.867159268 | 0.005761444 |
| 436588 | GO:0044237 | cellular metabolic process | BP | polymerase (RNA) II (DNA directed) polypeptide D | polr2d | -1.847965551 | 0.007836535 |
| 436588 | GO:0006351 | transcription, DNA-dependent | BP | polymerase (RNA) II (DNA directed) polypeptide D | polr2d | -1.847965551 | 0.007836535 |
| 436588 | GO:0003824 | catalytic activity | MF | polymerase (RNA) II (DNA directed) polypeptide D | polr2d | -1.847965551 | 0.007836535 |
| 436588 | GO:0003899 | DNA-directed RNA polymerase activity | MF | polymerase (RNA) II (DNA directed) polypeptide D | polr2d | -1.847965551 | 0.007836535 |
| 436588 | GO:0000166 | nucleotide binding | MF | polymerase (RNA) II (DNA directed) polypeptide D | polr2d | -1.847965551 | 0.007836535 |
| 405884 | GO:0006465 | signal peptide processing | BP | signal peptidase complex subunit 3 homolog (S. cerevisiae) | spcs3 | -1.836451641 | 0.007061308 |
| 405884 | GO:0016021 | integral to membrane | CC | signal peptidase complex subunit 3 homolog (S. cerevisiae) | spcs3 | -1.836451641 | 0.007061308 |
| 405884 | GO:0005787 | signal peptidase complex | CC | signal peptidase complex subunit 3 homolog (S. cerevisiae) | spcs3 | -1.836451641 | 0.007061308 |
| 405884 | GO:0008233 | peptidase activity | MF | signal peptidase complex subunit 3 homolog (S. cerevisiae) | spcs3 | -1.836451641 | 0.007061308 |
| 30714 | GO:0007186 | G-protein coupled receptor signaling pathway | BP | neuropeptide Y receptor Y8a | npy8ar | -1.799251262 | 0.008155383 |
| 30714 | GO:0007165 | signal transduction | BP | neuropeptide Y receptor Y8a | npy8ar | -1.799251262 | 0.008155383 |
| 30714 | GO:0016021 | integral to membrane | CC | neuropeptide Y receptor Y8a | npy8ar | -1.799251262 | 0.008155383 |
| 30714 | GO:0004930 | G-protein coupled receptor activity | MF | neuropeptide Y receptor Y8a | npy8ar | -1.799251262 | 0.008155383 |
| 30714 | GO:0004983 | neuropeptide Y receptor activity | MF | neuropeptide Y receptor Y8a | npy8ar | -1.799251262 | 0.008155383 |
| 30714 | GO:0004871 | signal transducer activity | MF | neuropeptide Y receptor Y8a | npy8ar | -1.799251262 | 0.008155383 |
| 100038789 | GO:0006334 | nucleosome assembly | BP | zgc:163040 | zgc:163040 | -1.782379129 | 0.008365116 |
| 100038789 | GO:0005694 | chromosome | CC | zgc:163040 | zgc:163040 | -1.782379129 | 0.008365116 |
| 100038789 | GO:0000786 | nucleosome | CC | zgc:163040 | zgc:163040 | -1.782379129 | 0.008365116 |
| 100038789 | GO:0005634 | nucleus | CC | zgc:163040 | zgc:163040 | -1.782379129 | 0.008365116 |
| 100038789 | GO:0003677 | DNA binding | MF | zgc:163040 | zgc:163040 | -1.782379129 | 0.008365116 |
| 100038789 | GO:0046982 | protein heterodimerization activity | MF | zgc:163040 | zgc:163040 | -1.782379129 | 0.008365116 |
| 394241 | GO:0043066 | negative regulation of apoptotic process | BP | Fanconi anemia, complementation group D2 | fancd2 | -1.782258861 | 0.008213356 |
| 58056 | GO:0006355 | regulation of transcription, DNA-dependent | BP | homeo box B10a | hoxb10a | -1.781172156 | 0.00826672 |
| 58056 | GO:0006351 | transcription, DNA-dependent | BP | homeo box B10a | hoxb10a | -1.781172156 | 0.00826672 |
| 58056 | GO:0005634 | nucleus | CC | homeo box B10a | hoxb10a | -1.781172156 | 0.00826672 |
| 58056 | GO:0003677 | DNA binding | MF | homeo box B10a | hoxb10a | -1.781172156 | 0.00826672 |
| 58056 | GO:0043565 | sequence-specific DNA binding | MF | homeo box B10a | hoxb10a | -1.781172156 | 0.00826672 |
| 58056 | GO:0003700 | sequence-specific DNA binding transcription factor activity | MF | homeo box B10a | hoxb10a | -1.781172156 | 0.00826672 |
| 450067 | GO:0006672 | ceramide metabolic process | BP | ORM1-like 3 (S. cerevisiae) | ormdl3 | -1.77291648 | 0.00890557 |
| 450067 | GO:0005783 | endoplasmic reticulum | CC | ORM1-like 3 (S. cerevisiae) | ormdl3 | -1.77291648 | 0.00890557 |
| 450067 | GO:0016021 | integral to membrane | CC | ORM1-like 3 (S. cerevisiae) | ormdl3 | -1.77291648 | 0.00890557 |
| 450067 | GO:0016020 | membrane | CC | ORM1-like 3 (S. cerevisiae) | ormdl3 | -1.77291648 | 0.00890557 |
| 450067 | GO:0035339 | SPOTS complex | CC | ORM1-like 3 (S. cerevisiae) | ormdl3 | -1.77291648 | 0.00890557 |
| 450067 | GO:0003674 | molecular_function | MF | ORM1-like 3 (S. cerevisiae) | ormdl3 | -1.77291648 | 0.00890557 |
| 100003752 | GO:0005882 | intermediate filament | CC | si:ch73-78i5.3 | si:ch73-78i5.3 | -1.760185989 | 0.009230611 |
| 100003752 | GO:0005198 | structural molecule activity | MF | si:ch73-78i5.3 | si:ch73-78i5.3 | -1.760185989 | 0.009230611 |
| 406652 | GO:0042493 | response to drug | BP | signal recognition particle 19 | srp19 | -1.757355714 | 0.009353192 |
| 406652 | GO:0006614 | SRP-dependent cotranslational protein targeting to membrane | BP | signal recognition particle 19 | srp19 | -1.757355714 | 0.009353192 |
| 406652 | GO:0048500 | signal recognition particle | CC | signal recognition particle 19 | srp19 | -1.757355714 | 0.009353192 |
| 406652 | GO:0008312 | 7S RNA binding | MF | signal recognition particle 19 | srp19 | -1.757355714 | 0.009353192 |
| 100526813 | GO:0042742 | defense response to bacterium | BP | si:dkey-22f5.9 | si:dkey-22f5.9 | -1.755799735 | 0.009225982 |
| 563772 | GO:0005622 | intracellular | CC | tripartite motif-containing 54 | trim54 | -1.752120156 | 0.00975005 |
| 563772 | GO:0046872 | metal ion binding | MF | tripartite motif-containing 54 | trim54 | -1.752120156 | 0.00975005 |
| 563772 | GO:0008270 | zinc ion binding | MF | tripartite motif-containing 54 | trim54 | -1.752120156 | 0.00975005 |
| 492765 | GO:0008150 | biological_process | BP | XK, Kell blood group complex subunit-related, X-linked | xkrx | -1.739814277 | 0.009645047 |
| 492765 | GO:0005575 | cellular_component | CC | XK, Kell blood group complex subunit-related, X-linked | xkrx | -1.739814277 | 0.009645047 |
| 492765 | GO:0003674 | molecular_function | MF | XK, Kell blood group complex subunit-related, X-linked | xkrx | -1.739814277 | 0.009645047 |
| 100170782 | GO:0005975 | carbohydrate metabolic process | BP | zgc:194879 | zgc:194879 | -1.725376091 | 0.010684865 |
| 100170782 | GO:0000139 | Golgi membrane | CC | zgc:194879 | zgc:194879 | -1.725376091 | 0.010684865 |
| 100170782 | GO:0008146 | sulfotransferase activity | MF | zgc:194879 | zgc:194879 | -1.725376091 | 0.010684865 |
| 553389 | GO:0051297 | centrosome organization | BP | HAUS augmin-like complex, subunit 1 | haus1 | -1.723028335 | 0.011060988 |
| 553389 | GO:0051225 | spindle assembly | BP | HAUS augmin-like complex, subunit 1 | haus1 | -1.723028335 | 0.011060988 |
| 553389 | GO:0070652 | HAUS complex | CC | HAUS augmin-like complex, subunit 1 | haus1 | -1.723028335 | 0.011060988 |
| 406513 | GO:0009966 | regulation of signal transduction | BP | sprouty-related, EVH1 domain containing 1 | spred1 | -1.718839583 | 0.010652123 |
| 406513 | GO:0016020 | membrane | CC | sprouty-related, EVH1 domain containing 1 | spred1 | -1.718839583 | 0.010652123 |
| 406513 | GO:0003674 | molecular_function | MF | sprouty-related, EVH1 domain containing 1 | spred1 | -1.718839583 | 0.010652123 |
| 30236 | GO:0007154 | cell communication | BP | connexin 43 | cx43 | -1.717128724 | 0.010690808 |
| 30236 | GO:0008283 | cell proliferation | BP | connexin 43 | cx43 | -1.717128724 | 0.010690808 |
| 30236 | GO:0007267 | cell-cell signaling | BP | connexin 43 | cx43 | -1.717128724 | 0.010690808 |
| 30236 | GO:0030054 | cell junction | CC | connexin 43 | cx43 | -1.717128724 | 0.010690808 |
| 30236 | GO:0005922 | connexon complex | CC | connexin 43 | cx43 | -1.717128724 | 0.010690808 |
| 30236 | GO:0005921 | gap junction | CC | connexin 43 | cx43 | -1.717128724 | 0.010690808 |
| 30236 | GO:0016021 | integral to membrane | CC | connexin 43 | cx43 | -1.717128724 | 0.010690808 |
| 30236 | GO:0016020 | membrane | CC | connexin 43 | cx43 | -1.717128724 | 0.010690808 |
| 30236 | GO:0005886 | plasma membrane | CC | connexin 43 | cx43 | -1.717128724 | 0.010690808 |
| 30236 | GO:0005243 | gap junction channel activity | MF | connexin 43 | cx43 | -1.717128724 | 0.010690808 |
| 30236 | GO:0055077 | gap junction hemi-channel activity | MF | connexin 43 | cx43 | -1.717128724 | 0.010690808 |
| 30236 | GO:0022857 | transmembrane transporter activity | MF | connexin 43 | cx43 | -1.717128724 | 0.010690808 |
| 30236 | GO:0022832 | voltage-gated channel activity | MF | connexin 43 | cx43 | -1.717128724 | 0.010690808 |
| 492516 | GO:0008150 | biological_process | BP | tetratricopeptide repeat domain 36 | ttc36 | -1.714652574 | 0.023362216 |
| 492516 | GO:0005575 | cellular_component | CC | tetratricopeptide repeat domain 36 | ttc36 | -1.714652574 | 0.023362216 |
| 100005158 | GO:0005507 | copper ion binding | MF | COX11 cytochrome c oxidase assembly homolog (yeast) | cox11 | -1.714132443 | 0.011208161 |
| 100170792 | GO:0008150 | biological_process | BP | zgc:194215 | zgc:194215 | -1.712808246 | 0.026299588 |
| 100170792 | GO:0005575 | cellular_component | CC | zgc:194215 | zgc:194215 | -1.712808246 | 0.026299588 |
| 100170792 | GO:0003674 | molecular_function | MF | zgc:194215 | zgc:194215 | -1.712808246 | 0.026299588 |
| 677740 | GO:0005529 | carbohydrate binding | MF | immune-related, lectin-like receptor 3 | illr3 | -1.711688942 | 0.01246533 |
| 321294 | GO:0042541 | hemoglobin biosynthetic process | BP | coproporphyrinogen oxidase | cpox | -1.699487541 | 0.011617367 |
| 321294 | GO:0055114 | oxidation-reduction process | BP | coproporphyrinogen oxidase | cpox | -1.699487541 | 0.011617367 |
| 321294 | GO:0006779 | porphyrin-containing compound biosynthetic process | BP | coproporphyrinogen oxidase | cpox | -1.699487541 | 0.011617367 |
| 321294 | GO:0004109 | coproporphyrinogen oxidase activity | MF | coproporphyrinogen oxidase | cpox | -1.699487541 | 0.011617367 |
| 393171 | GO:0006478 | peptidyl-tyrosine sulfation | BP | tyrosylprotein sulfotransferase 1, like | tpst1l | -1.689235094 | 0.011995449 |
| 393171 | GO:0008476 | protein-tyrosine sulfotransferase activity | MF | tyrosylprotein sulfotransferase 1, like | tpst1l | -1.689235094 | 0.011995449 |
| 393171 | GO:0016740 | transferase activity | MF | tyrosylprotein sulfotransferase 1, like | tpst1l | -1.689235094 | 0.011995449 |
| 555450 | GO:0006355 | regulation of transcription, DNA-dependent | BP | Scm-like with four mbt domains 2 | sfmbt2 | -1.68029987 | 0.012583946 |
| 555450 | GO:0005634 | nucleus | CC | Scm-like with four mbt domains 2 | sfmbt2 | -1.68029987 | 0.012583946 |
| 563138 | GO:0016758 | transferase activity, transferring hexosyl groups | MF | si:ch211-219a15.4 | si:ch211-219a15.4 | -1.677020894 | 0.012549187 |
| 30298 | GO:0007243 | intracellular protein kinase cascade | BP | Janus kinase 2b | jak2b | -1.676625722 | 0.012540767 |
| 30298 | GO:0016310 | phosphorylation | BP | Janus kinase 2b | jak2b | -1.676625722 | 0.012540767 |
| 30298 | GO:0006468 | protein phosphorylation | BP | Janus kinase 2b | jak2b | -1.676625722 | 0.012540767 |
| 30298 | GO:0005856 | cytoskeleton | CC | Janus kinase 2b | jak2b | -1.676625722 | 0.012540767 |
| 30298 | GO:0016020 | membrane | CC | Janus kinase 2b | jak2b | -1.676625722 | 0.012540767 |
| 30298 | GO:0005524 | ATP binding | MF | Janus kinase 2b | jak2b | -1.676625722 | 0.012540767 |
| 30298 | GO:0016301 | kinase activity | MF | Janus kinase 2b | jak2b | -1.676625722 | 0.012540767 |
| 30298 | GO:0004715 | non-membrane spanning protein tyrosine kinase activity | MF | Janus kinase 2b | jak2b | -1.676625722 | 0.012540767 |
| 30298 | GO:0000166 | nucleotide binding | MF | Janus kinase 2b | jak2b | -1.676625722 | 0.012540767 |
| 30298 | GO:0004672 | protein kinase activity | MF | Janus kinase 2b | jak2b | -1.676625722 | 0.012540767 |
| 30298 | GO:0004713 | protein tyrosine kinase activity | MF | Janus kinase 2b | jak2b | -1.676625722 | 0.012540767 |
| 30298 | GO:0016740 | transferase activity | MF | Janus kinase 2b | jak2b | -1.676625722 | 0.012540767 |
| 30298 | GO:0016772 | transferase activity, transferring phosphorus-containing groups | MF | Janus kinase 2b | jak2b | -1.676625722 | 0.012540767 |
| 378997 | GO:0005737 | cytoplasm | CC | glucocorticoid induced transcript 1 | glcci1 | -1.673833589 | 0.012782178 |
| 378997 | GO:0003674 | molecular_function | MF | glucocorticoid induced transcript 1 | glcci1 | -1.673833589 | 0.012782178 |
| 30586 | GO:0006355 | regulation of transcription, DNA-dependent | BP | distal-less homeobox gene 6a | dlx6a | -1.672333971 | 0.013228037 |
| 30586 | GO:0005634 | nucleus | CC | distal-less homeobox gene 6a | dlx6a | -1.672333971 | 0.013228037 |
| 30586 | GO:0003677 | DNA binding | MF | distal-less homeobox gene 6a | dlx6a | -1.672333971 | 0.013228037 |
| 30586 | GO:0043565 | sequence-specific DNA binding | MF | distal-less homeobox gene 6a | dlx6a | -1.672333971 | 0.013228037 |
| 30586 | GO:0003700 | sequence-specific DNA binding transcription factor activity | MF | distal-less homeobox gene 6a | dlx6a | -1.672333971 | 0.013228037 |
| 30586 | GO:0000976 | transcription regulatory region sequence-specific DNA binding | MF | distal-less homeobox gene 6a | dlx6a | -1.672333971 | 0.013228037 |
| 445310 | GO:0008150 | biological_process | BP | transmembrane protein 179 | tmem179 | -1.665927918 | 0.014078683 |
| 445310 | GO:0016021 | integral to membrane | CC | transmembrane protein 179 | tmem179 | -1.665927918 | 0.014078683 |
| 445310 | GO:0016020 | membrane | CC | transmembrane protein 179 | tmem179 | -1.665927918 | 0.014078683 |
| 445310 | GO:0003674 | molecular_function | MF | transmembrane protein 179 | tmem179 | -1.665927918 | 0.014078683 |
| 541445 | GO:0008150 | biological_process | BP | osteoglycin | ogn | -1.656658742 | 0.013821354 |
| 560495 | GO:0006355 | regulation of transcription, DNA-dependent | BP | si:ch211-195d17.2 | si:ch211-195d17.2 | -1.65561203 | 0.013636131 |
| 560495 | GO:0006351 | transcription, DNA-dependent | BP | si:ch211-195d17.2 | si:ch211-195d17.2 | -1.65561203 | 0.013636131 |
| 560495 | GO:0005634 | nucleus | CC | si:ch211-195d17.2 | si:ch211-195d17.2 | -1.65561203 | 0.013636131 |
| 560495 | GO:0005667 | transcription factor complex | CC | si:ch211-195d17.2 | si:ch211-195d17.2 | -1.65561203 | 0.013636131 |
| 560495 | GO:0003677 | DNA binding | MF | si:ch211-195d17.2 | si:ch211-195d17.2 | -1.65561203 | 0.013636131 |
| 560495 | GO:0003676 | nucleic acid binding | MF | si:ch211-195d17.2 | si:ch211-195d17.2 | -1.65561203 | 0.013636131 |
| 560495 | GO:0003700 | sequence-specific DNA binding transcription factor activity | MF | si:ch211-195d17.2 | si:ch211-195d17.2 | -1.65561203 | 0.013636131 |
| 405877 | GO:0045893 | positive regulation of transcription, DNA-dependent | BP | v-maf musculoaponeurotic fibrosarcoma oncogene homolog g (avian), 2 | mafg2 | -1.655141323 | 0.013769686 |
| 405877 | GO:0006355 | regulation of transcription, DNA-dependent | BP | v-maf musculoaponeurotic fibrosarcoma oncogene homolog g (avian), 2 | mafg2 | -1.655141323 | 0.013769686 |
| 405877 | GO:0005634 | nucleus | CC | v-maf musculoaponeurotic fibrosarcoma oncogene homolog g (avian), 2 | mafg2 | -1.655141323 | 0.013769686 |
| 405877 | GO:0005667 | transcription factor complex | CC | v-maf musculoaponeurotic fibrosarcoma oncogene homolog g (avian), 2 | mafg2 | -1.655141323 | 0.013769686 |
| 405877 | GO:0003677 | DNA binding | MF | v-maf musculoaponeurotic fibrosarcoma oncogene homolog g (avian), 2 | mafg2 | -1.655141323 | 0.013769686 |
| 405877 | GO:0046982 | protein heterodimerization activity | MF | v-maf musculoaponeurotic fibrosarcoma oncogene homolog g (avian), 2 | mafg2 | -1.655141323 | 0.013769686 |
| 405877 | GO:0043565 | sequence-specific DNA binding | MF | v-maf musculoaponeurotic fibrosarcoma oncogene homolog g (avian), 2 | mafg2 | -1.655141323 | 0.013769686 |
| 405877 | GO:0003700 | sequence-specific DNA binding transcription factor activity | MF | v-maf musculoaponeurotic fibrosarcoma oncogene homolog g (avian), 2 | mafg2 | -1.655141323 | 0.013769686 |
| 100330810 | GO:0042742 | defense response to bacterium | BP | nucleotide-binding oligomerization domain containing 1 | nod1 | -1.651531937 | 0.013736274 |
| 327276 | GO:0015711 | organic anion transport | BP | solute carrier family 16 (monocarboxylic acid transporters), member 3 | slc16a3 | -1.645752802 | 0.014245287 |
| 327276 | GO:0055085 | transmembrane transport | BP | solute carrier family 16 (monocarboxylic acid transporters), member 3 | slc16a3 | -1.645752802 | 0.014245287 |
| 327276 | GO:0016021 | integral to membrane | CC | solute carrier family 16 (monocarboxylic acid transporters), member 3 | slc16a3 | -1.645752802 | 0.014245287 |
| 327276 | GO:0015355 | secondary active monocarboxylate transmembrane transporter activity | MF | solute carrier family 16 (monocarboxylic acid transporters), member 3 | slc16a3 | -1.645752802 | 0.014245287 |
| 503864 | GO:0005576 | extracellular region | CC | relaxin 3a | rln3a | -1.636829073 | 0.017287672 |
| 503864 | GO:0005179 | hormone activity | MF | relaxin 3a | rln3a | -1.636829073 | 0.017287672 |
| 799124 | GO:0006811 | ion transport | BP | gamma-aminobutyric acid (GABA) A receptor, alpha 5 | gabra5 | -1.622965524 | 0.015630055 |
| 799124 | GO:0006810 | transport | BP | gamma-aminobutyric acid (GABA) A receptor, alpha 5 | gabra5 | -1.622965524 | 0.015630055 |
| 799124 | GO:0030054 | cell junction | CC | gamma-aminobutyric acid (GABA) A receptor, alpha 5 | gabra5 | -1.622965524 | 0.015630055 |
| 799124 | GO:0016021 | integral to membrane | CC | gamma-aminobutyric acid (GABA) A receptor, alpha 5 | gabra5 | -1.622965524 | 0.015630055 |
| 799124 | GO:0016020 | membrane | CC | gamma-aminobutyric acid (GABA) A receptor, alpha 5 | gabra5 | -1.622965524 | 0.015630055 |
| 799124 | GO:0005886 | plasma membrane | CC | gamma-aminobutyric acid (GABA) A receptor, alpha 5 | gabra5 | -1.622965524 | 0.015630055 |
| 799124 | GO:0005230 | extracellular ligand-gated ion channel activity | MF | gamma-aminobutyric acid (GABA) A receptor, alpha 5 | gabra5 | -1.622965524 | 0.015630055 |
| 799124 | GO:0004890 | GABA-A receptor activity | MF | gamma-aminobutyric acid (GABA) A receptor, alpha 5 | gabra5 | -1.622965524 | 0.015630055 |
| 799124 | GO:0005216 | ion channel activity | MF | gamma-aminobutyric acid (GABA) A receptor, alpha 5 | gabra5 | -1.622965524 | 0.015630055 |
| 30324 | GO:0005737 | cytoplasm | CC | alpha-tropomyosin | tpma | -1.609743843 | 0.016882184 |
| 30324 | GO:0005856 | cytoskeleton | CC | alpha-tropomyosin | tpma | -1.609743843 | 0.016882184 |
| 30324 | GO:0003779 | actin binding | MF | alpha-tropomyosin | tpma | -1.609743843 | 0.016882184 |
| 402952 | GO:0003676 | nucleic acid binding | MF | zgc:77262 | zgc:77262 | -1.602589465 | 0.01671265 |
| 402952 | GO:0000166 | nucleotide binding | MF | zgc:77262 | zgc:77262 | -1.602589465 | 0.01671265 |
| 30038 | GO:0051091 | positive regulation of sequence-specific DNA binding transcription factor activity | BP | SRY-box containing gene 19a | sox19a | -1.597115666 | 0.017012547 |
| 30038 | GO:0006355 | regulation of transcription, DNA-dependent | BP | SRY-box containing gene 19a | sox19a | -1.597115666 | 0.017012547 |
| 30038 | GO:0006351 | transcription, DNA-dependent | BP | SRY-box containing gene 19a | sox19a | -1.597115666 | 0.017012547 |
| 30038 | GO:0005634 | nucleus | CC | SRY-box containing gene 19a | sox19a | -1.597115666 | 0.017012547 |
| 30038 | GO:0005667 | transcription factor complex | CC | SRY-box containing gene 19a | sox19a | -1.597115666 | 0.017012547 |
| 30038 | GO:0003677 | DNA binding | MF | SRY-box containing gene 19a | sox19a | -1.597115666 | 0.017012547 |
| 30038 | GO:0003700 | sequence-specific DNA binding transcription factor activity | MF | SRY-box containing gene 19a | sox19a | -1.597115666 | 0.017012547 |
| 791199 | GO:0006909 | phagocytosis | BP | ELMO/CED-12 domain containing 1 | elmod1 | -1.587778504 | 0.018667776 |
| 791199 | GO:0005856 | cytoskeleton | CC | ELMO/CED-12 domain containing 1 | elmod1 | -1.587778504 | 0.018667776 |
| 791199 | GO:0003674 | molecular_function | MF | ELMO/CED-12 domain containing 1 | elmod1 | -1.587778504 | 0.018667776 |
| 393120 | GO:0005575 | cellular_component | CC | Josephin domain containing 2 | josd2 | -1.586239752 | 0.01790793 |
| 393120 | GO:0008242 | omega peptidase activity | MF | Josephin domain containing 2 | josd2 | -1.586239752 | 0.01790793 |
| 402850 | GO:0000290 | deadenylation-dependent decapping of nuclear-transcribed mRNA | BP | mRNA decapping enzyme | dcps | -1.585643597 | 0.018430363 |
| 402850 | GO:0003824 | catalytic activity | MF | mRNA decapping enzyme | dcps | -1.585643597 | 0.018430363 |
| 402850 | GO:0016787 | hydrolase activity | MF | mRNA decapping enzyme | dcps | -1.585643597 | 0.018430363 |
| 334271 | GO:0005622 | intracellular | CC | zgc:171476 | zgc:171476 | -1.585508559 | 0.018061322 |
| 334271 | GO:0008270 | zinc ion binding | MF | zgc:171476 | zgc:171476 | -1.585508559 | 0.018061322 |
| 58076 | GO:0045944 | positive regulation of transcription from RNA polymerase II promoter | BP | GATA-binding protein 6 | gata6 | -1.577744908 | 0.018676627 |
| 58076 | GO:0045893 | positive regulation of transcription, DNA-dependent | BP | GATA-binding protein 6 | gata6 | -1.577744908 | 0.018676627 |
| 58076 | GO:0006355 | regulation of transcription, DNA-dependent | BP | GATA-binding protein 6 | gata6 | -1.577744908 | 0.018676627 |
| 58076 | GO:0005634 | nucleus | CC | GATA-binding protein 6 | gata6 | -1.577744908 | 0.018676627 |
| 58076 | GO:0003677 | DNA binding | MF | GATA-binding protein 6 | gata6 | -1.577744908 | 0.018676627 |
| 58076 | GO:0043565 | sequence-specific DNA binding | MF | GATA-binding protein 6 | gata6 | -1.577744908 | 0.018676627 |
| 58076 | GO:0003700 | sequence-specific DNA binding transcription factor activity | MF | GATA-binding protein 6 | gata6 | -1.577744908 | 0.018676627 |
| 58076 | GO:0008270 | zinc ion binding | MF | GATA-binding protein 6 | gata6 | -1.577744908 | 0.018676627 |
| 503752 | GO:0005575 | cellular_component | CC | peptidyl-tRNA hydrolase 1 homolog (S. cerevisiae) | ptrh1 | -1.573287275 | 0.024798549 |
| 503752 | GO:0004045 | aminoacyl-tRNA hydrolase activity | MF | peptidyl-tRNA hydrolase 1 homolog (S. cerevisiae) | ptrh1 | -1.573287275 | 0.024798549 |
| 503752 | GO:0016787 | hydrolase activity | MF | peptidyl-tRNA hydrolase 1 homolog (S. cerevisiae) | ptrh1 | -1.573287275 | 0.024798549 |
| 554122 | GO:0005575 | cellular_component | CC | zgc:112374 | zgc:112374 | -1.558792725 | 0.020650229 |
| 554122 | GO:0004064 | arylesterase activity | MF | zgc:112374 | zgc:112374 | -1.558792725 | 0.020650229 |
| 449555 | GO:0008152 | metabolic process | BP | zgc:101858 | zgc:101858 | -1.550208957 | 0.021239735 |
| 449555 | GO:0005575 | cellular_component | CC | zgc:101858 | zgc:101858 | -1.550208957 | 0.021239735 |
| 449555 | GO:0000166 | nucleotide binding | MF | zgc:101858 | zgc:101858 | -1.550208957 | 0.021239735 |
| 449555 | GO:0016491 | oxidoreductase activity | MF | zgc:101858 | zgc:101858 | -1.550208957 | 0.021239735 |
| 100002261 | GO:0006367 | transcription initiation from RNA polymerase II promoter | BP | TAF7 RNA polymerase II, TATA box binding protein (TBP)-associated factor | taf7 | -1.547250676 | 0.020925668 |
| 100002261 | GO:0005669 | transcription factor TFIID complex | CC | TAF7 RNA polymerase II, TATA box binding protein (TBP)-associated factor | taf7 | -1.547250676 | 0.020925668 |
| 393225 | GO:0032259 | methylation | BP | DNA methyltransferase 1 associated protein 1 | dmap1 | -1.543401264 | 0.021573709 |
| 393225 | GO:0045892 | negative regulation of transcription, DNA-dependent | BP | DNA methyltransferase 1 associated protein 1 | dmap1 | -1.543401264 | 0.021573709 |
| 393225 | GO:0005634 | nucleus | CC | DNA methyltransferase 1 associated protein 1 | dmap1 | -1.543401264 | 0.021573709 |
| 393225 | GO:0003682 | chromatin binding | MF | DNA methyltransferase 1 associated protein 1 | dmap1 | -1.543401264 | 0.021573709 |
| 393225 | GO:0008168 | methyltransferase activity | MF | DNA methyltransferase 1 associated protein 1 | dmap1 | -1.543401264 | 0.021573709 |
| 393225 | GO:0016740 | transferase activity | MF | DNA methyltransferase 1 associated protein 1 | dmap1 | -1.543401264 | 0.021573709 |
| 65235 | GO:0006355 | regulation of transcription, DNA-dependent | BP | sine oculis homeobox homolog 4.3 | six4.3 | -1.527779956 | 0.022465337 |
| 65235 | GO:0005634 | nucleus | CC | sine oculis homeobox homolog 4.3 | six4.3 | -1.527779956 | 0.022465337 |
| 65235 | GO:0003677 | DNA binding | MF | sine oculis homeobox homolog 4.3 | six4.3 | -1.527779956 | 0.022465337 |
| 65235 | GO:0043565 | sequence-specific DNA binding | MF | sine oculis homeobox homolog 4.3 | six4.3 | -1.527779956 | 0.022465337 |
| 65235 | GO:0003700 | sequence-specific DNA binding transcription factor activity | MF | sine oculis homeobox homolog 4.3 | six4.3 | -1.527779956 | 0.022465337 |
| 393212 | GO:0008150 | biological_process | BP | zgc:56041 | zgc:56041 | -1.514413456 | 0.023445366 |
| 393212 | GO:0005575 | cellular_component | CC | zgc:56041 | zgc:56041 | -1.514413456 | 0.023445366 |
| 566871 | GO:0005525 | GTP binding | MF | si:dkey-32e23.4 | si:dkey-32e23.4 | -1.512003754 | 0.02376579 |
| 566871 | GO:0003924 | GTPase activity | MF | si:dkey-32e23.4 | si:dkey-32e23.4 | -1.512003754 | 0.02376579 |
| 566871 | GO:0000166 | nucleotide binding | MF | si:dkey-32e23.4 | si:dkey-32e23.4 | -1.512003754 | 0.02376579 |
| 567859 | GO:0009103 | lipopolysaccharide biosynthetic process | BP | glycosyltransferase 25 domain containing 1 | glt25d1 | -1.504801593 | 0.024397137 |
| 554138 | GO:0070940 | dephosphorylation of RNA polymerase II C-terminal domain | BP | RNA polymerase II associated protein 2 | rpap2 | -1.502609004 | 0.02668418 |
| 554138 | GO:0006355 | regulation of transcription, DNA-dependent | BP | RNA polymerase II associated protein 2 | rpap2 | -1.502609004 | 0.02668418 |
| 554138 | GO:0009301 | snRNA transcription | BP | RNA polymerase II associated protein 2 | rpap2 | -1.502609004 | 0.02668418 |
| 554138 | GO:0006351 | transcription, DNA-dependent | BP | RNA polymerase II associated protein 2 | rpap2 | -1.502609004 | 0.02668418 |
| 554138 | GO:0005737 | cytoplasm | CC | RNA polymerase II associated protein 2 | rpap2 | -1.502609004 | 0.02668418 |
| 554138 | GO:0016591 | DNA-directed RNA polymerase II, holoenzyme | CC | RNA polymerase II associated protein 2 | rpap2 | -1.502609004 | 0.02668418 |
| 554138 | GO:0005634 | nucleus | CC | RNA polymerase II associated protein 2 | rpap2 | -1.502609004 | 0.02668418 |
| 554138 | GO:0008420 | CTD phosphatase activity | MF | RNA polymerase II associated protein 2 | rpap2 | -1.502609004 | 0.02668418 |
| 554138 | GO:0016787 | hydrolase activity | MF | RNA polymerase II associated protein 2 | rpap2 | -1.502609004 | 0.02668418 |
| 554138 | GO:0046872 | metal ion binding | MF | RNA polymerase II associated protein 2 | rpap2 | -1.502609004 | 0.02668418 |
| 554138 | GO:0004721 | phosphoprotein phosphatase activity | MF | RNA polymerase II associated protein 2 | rpap2 | -1.502609004 | 0.02668418 |
| 394143 | GO:0006396 | RNA processing | BP | splicing factor, suppressor of white-apricot homolog (Drosophila) | sfswap | -1.499618854 | 0.024854532 |
| 394143 | GO:0005575 | cellular_component | CC | splicing factor, suppressor of white-apricot homolog (Drosophila) | sfswap | -1.499618854 | 0.024854532 |
| 394143 | GO:0003723 | RNA binding | MF | splicing factor, suppressor of white-apricot homolog (Drosophila) | sfswap | -1.499618854 | 0.024854532 |
| 100007371 | GO:0042742 | defense response to bacterium | BP | complement component 1, q subcomponent-like 4 like | c1ql4l | -1.495648156 | 0.026184031 |
| 100007371 | GO:0043066 | negative regulation of apoptotic process | BP | complement component 1, q subcomponent-like 4 like | c1ql4l | -1.495648156 | 0.026184031 |
| 100007371 | GO:0045930 | negative regulation of mitotic cell cycle | BP | complement component 1, q subcomponent-like 4 like | c1ql4l | -1.495648156 | 0.026184031 |
| 100007371 | GO:0043027 | cysteine-type endopeptidase inhibitor activity involved in apoptotic process | MF | complement component 1, q subcomponent-like 4 like | c1ql4l | -1.495648156 | 0.026184031 |
| 378848 | GO:0006950 | response to stress | BP | heat shock protein 5 | hspa5 | -1.47409136 | 0.027503805 |
| 378848 | GO:0005576 | extracellular region | CC | heat shock protein 5 | hspa5 | -1.47409136 | 0.027503805 |
| 378848 | GO:0005524 | ATP binding | MF | heat shock protein 5 | hspa5 | -1.47409136 | 0.027503805 |
| 378848 | GO:0000166 | nucleotide binding | MF | heat shock protein 5 | hspa5 | -1.47409136 | 0.027503805 |
| 791699 | GO:0008150 | biological_process | BP | zgc:154064 | zgc:154064 | -1.47034122 | 0.027811054 |
| 791699 | GO:0005575 | cellular_component | CC | zgc:154064 | zgc:154064 | -1.47034122 | 0.027811054 |
| 791699 | GO:0003674 | molecular_function | MF | zgc:154064 | zgc:154064 | -1.47034122 | 0.027811054 |
| 436745 | GO:0070588 | calcium ion transmembrane transport | BP | ATPase, Ca++ transporting, plasma membrane 3a | atp2b3a | -1.469253442 | 0.028046456 |
| 436745 | GO:0006812 | cation transport | BP | ATPase, Ca++ transporting, plasma membrane 3a | atp2b3a | -1.469253442 | 0.028046456 |
| 436745 | GO:0016021 | integral to membrane | CC | ATPase, Ca++ transporting, plasma membrane 3a | atp2b3a | -1.469253442 | 0.028046456 |
| 436745 | GO:0016020 | membrane | CC | ATPase, Ca++ transporting, plasma membrane 3a | atp2b3a | -1.469253442 | 0.028046456 |
| 436745 | GO:0005524 | ATP binding | MF | ATPase, Ca++ transporting, plasma membrane 3a | atp2b3a | -1.469253442 | 0.028046456 |
| 436745 | GO:0005388 | calcium-transporting ATPase activity | MF | ATPase, Ca++ transporting, plasma membrane 3a | atp2b3a | -1.469253442 | 0.028046456 |
| 436745 | GO:0019829 | cation-transporting ATPase activity | MF | ATPase, Ca++ transporting, plasma membrane 3a | atp2b3a | -1.469253442 | 0.028046456 |
| 436745 | GO:0016787 | hydrolase activity | MF | ATPase, Ca++ transporting, plasma membrane 3a | atp2b3a | -1.469253442 | 0.028046456 |
| 436745 | GO:0016820 | hydrolase activity, acting on acid anhydrides, catalyzing transmembrane movement of substances | MF | ATPase, Ca++ transporting, plasma membrane 3a | atp2b3a | -1.469253442 | 0.028046456 |
| 436745 | GO:0046872 | metal ion binding | MF | ATPase, Ca++ transporting, plasma membrane 3a | atp2b3a | -1.469253442 | 0.028046456 |
| 436745 | GO:0000166 | nucleotide binding | MF | ATPase, Ca++ transporting, plasma membrane 3a | atp2b3a | -1.469253442 | 0.028046456 |
| 565000 | GO:0000178 | exosome (RNase complex) | CC | exosome component 3 | exosc3 | -1.466445115 | 0.029164509 |
| 565000 | GO:0003723 | RNA binding | MF | exosome component 3 | exosc3 | -1.466445115 | 0.029164509 |
| 393518 | GO:0042176 | regulation of protein catabolic process | BP | proteasome (prosome, macropain) 26S subunit, non-ATPase, 2 | psmd2 | -1.465150433 | 0.028338646 |
| 393518 | GO:0000502 | proteasome complex | CC | proteasome (prosome, macropain) 26S subunit, non-ATPase, 2 | psmd2 | -1.465150433 | 0.028338646 |
| 393518 | GO:0030234 | enzyme regulator activity | MF | proteasome (prosome, macropain) 26S subunit, non-ATPase, 2 | psmd2 | -1.465150433 | 0.028338646 |
| 751692 | GO:0009303 | rRNA transcription | BP | ribonuclease, RNase K a | rnaseka | -1.454946745 | 0.030293799 |
| 751692 | GO:0016021 | integral to membrane | CC | ribonuclease, RNase K a | rnaseka | -1.454946745 | 0.030293799 |
| 751692 | GO:0016020 | membrane | CC | ribonuclease, RNase K a | rnaseka | -1.454946745 | 0.030293799 |
| 751692 | GO:0004519 | endonuclease activity | MF | ribonuclease, RNase K a | rnaseka | -1.454946745 | 0.030293799 |
| 751692 | GO:0004521 | endoribonuclease activity | MF | ribonuclease, RNase K a | rnaseka | -1.454946745 | 0.030293799 |
| 751692 | GO:0016787 | hydrolase activity | MF | ribonuclease, RNase K a | rnaseka | -1.454946745 | 0.030293799 |
| 751692 | GO:0004518 | nuclease activity | MF | ribonuclease, RNase K a | rnaseka | -1.454946745 | 0.030293799 |
| 64810 | GO:0006355 | regulation of transcription, DNA-dependent | BP | ventral expressed homeobox | vent | -1.441801009 | 0.03179295 |
| 64810 | GO:0005634 | nucleus | CC | ventral expressed homeobox | vent | -1.441801009 | 0.03179295 |
| 64810 | GO:0003677 | DNA binding | MF | ventral expressed homeobox | vent | -1.441801009 | 0.03179295 |
| 64810 | GO:0043565 | sequence-specific DNA binding | MF | ventral expressed homeobox | vent | -1.441801009 | 0.03179295 |
| 64810 | GO:0003700 | sequence-specific DNA binding transcription factor activity | MF | ventral expressed homeobox | vent | -1.441801009 | 0.03179295 |
| 100009631 | GO:0055085 | transmembrane transport | BP | solute carrier family 43, member 1b | slc43a1b | -1.441533123 | 0.031206126 |
| 100009631 | GO:0016021 | integral to membrane | CC | solute carrier family 43, member 1b | slc43a1b | -1.441533123 | 0.031206126 |
| 100009631 | GO:0003674 | molecular_function | MF | solute carrier family 43, member 1b | slc43a1b | -1.441533123 | 0.031206126 |
| 541449 | GO:0046474 | glycerophospholipid biosynthetic process | BP | zgc:113516 | zgc:113516 | -1.440629789 | 0.031039078 |
| 541449 | GO:0005575 | cellular_component | CC | zgc:113516 | zgc:113516 | -1.440629789 | 0.031039078 |
| 541449 | GO:0016301 | kinase activity | MF | zgc:113516 | zgc:113516 | -1.440629789 | 0.031039078 |
| 541449 | GO:0016772 | transferase activity, transferring phosphorus-containing groups | MF | zgc:113516 | zgc:113516 | -1.440629789 | 0.031039078 |
| 100093709 | GO:0007186 | G-protein coupled receptor signaling pathway | BP | odorant receptor, family F, subfamily 115, member 13 | or115-13 | -1.439527101 | 0.034158909 |
| 100093709 | GO:0007165 | signal transduction | BP | odorant receptor, family F, subfamily 115, member 13 | or115-13 | -1.439527101 | 0.034158909 |
| 100093709 | GO:0016021 | integral to membrane | CC | odorant receptor, family F, subfamily 115, member 13 | or115-13 | -1.439527101 | 0.034158909 |
| 100093709 | GO:0016020 | membrane | CC | odorant receptor, family F, subfamily 115, member 13 | or115-13 | -1.439527101 | 0.034158909 |
| 100093709 | GO:0005886 | plasma membrane | CC | odorant receptor, family F, subfamily 115, member 13 | or115-13 | -1.439527101 | 0.034158909 |
| 100093709 | GO:0004930 | G-protein coupled receptor activity | MF | odorant receptor, family F, subfamily 115, member 13 | or115-13 | -1.439527101 | 0.034158909 |
| 100093709 | GO:0004871 | signal transducer activity | MF | odorant receptor, family F, subfamily 115, member 13 | or115-13 | -1.439527101 | 0.034158909 |
| 336959 | GO:0005575 | cellular_component | CC | metallophosphoesterase domain containing 2 | mpped2 | -1.438154948 | 0.031706533 |
| 336959 | GO:0016787 | hydrolase activity | MF | metallophosphoesterase domain containing 2 | mpped2 | -1.438154948 | 0.031706533 |
| 654774 | GO:0007264 | small GTPase mediated signal transduction | BP | dedicator of cytokinesis 4b | dock4b | -1.437877514 | 0.031227421 |
| 654774 | GO:0005085 | guanyl-nucleotide exchange factor activity | MF | dedicator of cytokinesis 4b | dock4b | -1.437877514 | 0.031227421 |
| 553417 | GO:0016311 | dephosphorylation | BP | protein phosphatase 1E (PP2C domain containing) | ppm1e | -1.436441782 | 0.031452591 |
| 553417 | GO:0016310 | phosphorylation | BP | protein phosphatase 1E (PP2C domain containing) | ppm1e | -1.436441782 | 0.031452591 |
| 553417 | GO:0006470 | protein dephosphorylation | BP | protein phosphatase 1E (PP2C domain containing) | ppm1e | -1.436441782 | 0.031452591 |
| 553417 | GO:0005634 | nucleus | CC | protein phosphatase 1E (PP2C domain containing) | ppm1e | -1.436441782 | 0.031452591 |
| 553417 | GO:0003824 | catalytic activity | MF | protein phosphatase 1E (PP2C domain containing) | ppm1e | -1.436441782 | 0.031452591 |
| 553417 | GO:0016787 | hydrolase activity | MF | protein phosphatase 1E (PP2C domain containing) | ppm1e | -1.436441782 | 0.031452591 |
| 553417 | GO:0016301 | kinase activity | MF | protein phosphatase 1E (PP2C domain containing) | ppm1e | -1.436441782 | 0.031452591 |
| 553417 | GO:0046872 | metal ion binding | MF | protein phosphatase 1E (PP2C domain containing) | ppm1e | -1.436441782 | 0.031452591 |
| 553417 | GO:0004721 | phosphoprotein phosphatase activity | MF | protein phosphatase 1E (PP2C domain containing) | ppm1e | -1.436441782 | 0.031452591 |
| 553417 | GO:0004722 | protein serine/threonine phosphatase activity | MF | protein phosphatase 1E (PP2C domain containing) | ppm1e | -1.436441782 | 0.031452591 |
| 30071 | GO:0045893 | positive regulation of transcription, DNA-dependent | BP | T-box 5a | tbx5a | -1.436090191 | 0.031543989 |
| 30071 | GO:0006355 | regulation of transcription, DNA-dependent | BP | T-box 5a | tbx5a | -1.436090191 | 0.031543989 |
| 30071 | GO:0006351 | transcription, DNA-dependent | BP | T-box 5a | tbx5a | -1.436090191 | 0.031543989 |
| 30071 | GO:0015629 | actin cytoskeleton | CC | T-box 5a | tbx5a | -1.436090191 | 0.031543989 |
| 30071 | GO:0005634 | nucleus | CC | T-box 5a | tbx5a | -1.436090191 | 0.031543989 |
| 30071 | GO:0003677 | DNA binding | MF | T-box 5a | tbx5a | -1.436090191 | 0.031543989 |
| 30071 | GO:0043565 | sequence-specific DNA binding | MF | T-box 5a | tbx5a | -1.436090191 | 0.031543989 |
| 30071 | GO:0003700 | sequence-specific DNA binding transcription factor activity | MF | T-box 5a | tbx5a | -1.436090191 | 0.031543989 |
| 393755 | GO:0005509 | calcium ion binding | MF | Kv channel interacting protein 3, calsenilin | kcnip3 | -1.43123946 | 0.032441002 |
| 554057 | GO:0007155 | cell adhesion | BP | protocadherin 2 gamma 9 | pcdh2g9 | -1.427301775 | 0.035512873 |
| 554057 | GO:0007156 | homophilic cell adhesion | BP | protocadherin 2 gamma 9 | pcdh2g9 | -1.427301775 | 0.035512873 |
| 554057 | GO:0016021 | integral to membrane | CC | protocadherin 2 gamma 9 | pcdh2g9 | -1.427301775 | 0.035512873 |
| 554057 | GO:0016020 | membrane | CC | protocadherin 2 gamma 9 | pcdh2g9 | -1.427301775 | 0.035512873 |
| 554057 | GO:0005886 | plasma membrane | CC | protocadherin 2 gamma 9 | pcdh2g9 | -1.427301775 | 0.035512873 |
| 554057 | GO:0005509 | calcium ion binding | MF | protocadherin 2 gamma 9 | pcdh2g9 | -1.427301775 | 0.035512873 |
| 100005589 | GO:0055085 | transmembrane transport | BP | si:dkey-24h22.4 | si:dkey-24h22.4 | -1.418267579 | 0.034435539 |
| 100005589 | GO:0006810 | transport | BP | si:dkey-24h22.4 | si:dkey-24h22.4 | -1.418267579 | 0.034435539 |
| 100005589 | GO:0016021 | integral to membrane | CC | si:dkey-24h22.4 | si:dkey-24h22.4 | -1.418267579 | 0.034435539 |
| 100005589 | GO:0016020 | membrane | CC | si:dkey-24h22.4 | si:dkey-24h22.4 | -1.418267579 | 0.034435539 |
| 100005589 | GO:0005215 | transporter activity | MF | si:dkey-24h22.4 | si:dkey-24h22.4 | -1.418267579 | 0.034435539 |
| 64674 | GO:0007229 | integrin-mediated signaling pathway | BP | muscle-specific beta 1 integrin binding protein | mibp | -1.41401697 | 0.034504213 |
| 64674 | GO:0016310 | phosphorylation | BP | muscle-specific beta 1 integrin binding protein | mibp | -1.41401697 | 0.034504213 |
| 64674 | GO:0016301 | kinase activity | MF | muscle-specific beta 1 integrin binding protein | mibp | -1.41401697 | 0.034504213 |
| 619273 | GO:0008150 | biological_process | BP | zgc:114119 | zgc:114119 | -1.412615723 | 0.035464236 |
| 619273 | GO:0005575 | cellular_component | CC | zgc:114119 | zgc:114119 | -1.412615723 | 0.035464236 |
| 619273 | GO:0003674 | molecular_function | MF | zgc:114119 | zgc:114119 | -1.412615723 | 0.035464236 |
| 437011 | GO:0051260 | protein homooligomerization | BP | potassium channel tetramerisation domain containing 9 | kctd9 | -1.412033198 | 0.034280358 |
| 437011 | GO:0005216 | ion channel activity | MF | potassium channel tetramerisation domain containing 9 | kctd9 | -1.412033198 | 0.034280358 |
| 100000230 | GO:0005737 | cytoplasm | CC | si:ch211-166a6.5 | si:ch211-166a6.5 | -1.407931024 | 0.03483645 |
| 798997 | GO:0006355 | regulation of transcription, DNA-dependent | BP | zgc:110584 | zgc:110584 | -1.400817189 | 0.036755243 |
| 798997 | GO:0006367 | transcription initiation from RNA polymerase II promoter | BP | zgc:110584 | zgc:110584 | -1.400817189 | 0.036755243 |
| 798997 | GO:0006351 | transcription, DNA-dependent | BP | zgc:110584 | zgc:110584 | -1.400817189 | 0.036755243 |
| 798997 | GO:0005634 | nucleus | CC | zgc:110584 | zgc:110584 | -1.400817189 | 0.036755243 |
| 798997 | GO:0005672 | transcription factor TFIIA complex | CC | zgc:110584 | zgc:110584 | -1.400817189 | 0.036755243 |
| 436804 | GO:0009968 | negative regulation of signal transduction | BP | regulator of G-protein signaling 8 | rgs8 | -1.40022699 | 0.036481347 |
| 436804 | GO:0043547 | positive regulation of GTPase activity | BP | regulator of G-protein signaling 8 | rgs8 | -1.40022699 | 0.036481347 |
| 436804 | GO:0038032 | termination of G-protein coupled receptor signaling pathway | BP | regulator of G-protein signaling 8 | rgs8 | -1.40022699 | 0.036481347 |
| 436804 | GO:0005737 | cytoplasm | CC | regulator of G-protein signaling 8 | rgs8 | -1.40022699 | 0.036481347 |
| 436804 | GO:0005886 | plasma membrane | CC | regulator of G-protein signaling 8 | rgs8 | -1.40022699 | 0.036481347 |
| 436804 | GO:0005096 | GTPase activator activity | MF | regulator of G-protein signaling 8 | rgs8 | -1.40022699 | 0.036481347 |
| 492780 | GO:0005737 | cytoplasm | CC | bridging integrator 2a | bin2a | -1.397211038 | 0.037063884 |
| 393235 | GO:0005575 | cellular_component | CC | tetratricopeptide repeat domain 9C | ttc9c | -1.395439397 | 0.03648751 |
| 394133 | GO:0008152 | metabolic process | BP | aldehyde dehydrogenase 4 family, member A1 | aldh4a1 | -1.392079421 | 0.037327078 |
| 394133 | GO:0055114 | oxidation-reduction process | BP | aldehyde dehydrogenase 4 family, member A1 | aldh4a1 | -1.392079421 | 0.037327078 |
| 394133 | GO:0006561 | proline biosynthetic process | BP | aldehyde dehydrogenase 4 family, member A1 | aldh4a1 | -1.392079421 | 0.037327078 |
| 394133 | GO:0006560 | proline metabolic process | BP | aldehyde dehydrogenase 4 family, member A1 | aldh4a1 | -1.392079421 | 0.037327078 |
| 394133 | GO:0005759 | mitochondrial matrix | CC | aldehyde dehydrogenase 4 family, member A1 | aldh4a1 | -1.392079421 | 0.037327078 |
| 394133 | GO:0005739 | mitochondrion | CC | aldehyde dehydrogenase 4 family, member A1 | aldh4a1 | -1.392079421 | 0.037327078 |
| 394133 | GO:0003842 | 1-pyrroline-5-carboxylate dehydrogenase activity | MF | aldehyde dehydrogenase 4 family, member A1 | aldh4a1 | -1.392079421 | 0.037327078 |
| 394133 | GO:0016491 | oxidoreductase activity | MF | aldehyde dehydrogenase 4 family, member A1 | aldh4a1 | -1.392079421 | 0.037327078 |
| 394133 | GO:0016620 | oxidoreductase activity, acting on the aldehyde or oxo group of donors, NAD or NADP as acceptor | MF | aldehyde dehydrogenase 4 family, member A1 | aldh4a1 | -1.392079421 | 0.037327078 |
| 791878 | GO:0010466 | negative regulation of peptidase activity | BP | cystatin C (amyloid angiopathy and cerebral hemorrhage) | cst3 | -1.391591304 | 0.037561718 |
| 791878 | GO:0005575 | cellular_component | CC | cystatin C (amyloid angiopathy and cerebral hemorrhage) | cst3 | -1.391591304 | 0.037561718 |
| 791878 | GO:0004869 | cysteine-type endopeptidase inhibitor activity | MF | cystatin C (amyloid angiopathy and cerebral hemorrhage) | cst3 | -1.391591304 | 0.037561718 |
| 791878 | GO:0030414 | peptidase inhibitor activity | MF | cystatin C (amyloid angiopathy and cerebral hemorrhage) | cst3 | -1.391591304 | 0.037561718 |
| 30470 | GO:0005737 | cytoplasm | CC | erythrocyte protein band 4.1-like 4 | epb4.1l4 | -1.390587955 | 0.037395832 |
| 30470 | GO:0005856 | cytoskeleton | CC | erythrocyte protein band 4.1-like 4 | epb4.1l4 | -1.390587955 | 0.037395832 |
| 30470 | GO:0005516 | calmodulin binding | MF | erythrocyte protein band 4.1-like 4 | epb4.1l4 | -1.390587955 | 0.037395832 |
| 571967 | GO:0020037 | heme binding | MF | indoleamine 2,3-dioxygenase 1 | ido1 | -1.390159498 | 0.040133674 |
| 571967 | GO:0016702 | oxidoreductase activity, acting on single donors with incorporation of molecular oxygen, incorporation of two atoms of oxygen | MF | indoleamine 2,3-dioxygenase 1 | ido1 | -1.390159498 | 0.040133674 |
| 562050 | GO:0006810 | transport | BP | zgc:158225 | zgc:158225 | -1.388531941 | 0.03712038 |
| 562050 | GO:0016021 | integral to membrane | CC | zgc:158225 | zgc:158225 | -1.388531941 | 0.03712038 |
| 562050 | GO:0016020 | membrane | CC | zgc:158225 | zgc:158225 | -1.388531941 | 0.03712038 |
| 497073 | GO:0008150 | biological_process | BP | XK, Kell blood group complex subunit-related family, member 7 | xkr7 | -1.387386931 | 0.037122513 |
| 497073 | GO:0005575 | cellular_component | CC | XK, Kell blood group complex subunit-related family, member 7 | xkr7 | -1.387386931 | 0.037122513 |
| 497073 | GO:0003674 | molecular_function | MF | XK, Kell blood group complex subunit-related family, member 7 | xkr7 | -1.387386931 | 0.037122513 |
| 100124602 | GO:0008150 | biological_process | BP | zgc:171719 | zgc:171719 | -1.385132342 | 0.037806717 |
| 100124602 | GO:0005575 | cellular_component | CC | zgc:171719 | zgc:171719 | -1.385132342 | 0.037806717 |
| 100124602 | GO:0003674 | molecular_function | MF | zgc:171719 | zgc:171719 | -1.385132342 | 0.037806717 |
| 393533 | GO:0001525 | angiogenesis | BP | frizzled homolog 6 (Drosophila) | fzd6 | -1.384398625 | 0.037681311 |
| 393533 | GO:0060070 | canonical Wnt receptor signaling pathway | BP | frizzled homolog 6 (Drosophila) | fzd6 | -1.384398625 | 0.037681311 |
| 393533 | GO:0007166 | cell surface receptor signaling pathway | BP | frizzled homolog 6 (Drosophila) | fzd6 | -1.384398625 | 0.037681311 |
| 393533 | GO:0007186 | G-protein coupled receptor signaling pathway | BP | frizzled homolog 6 (Drosophila) | fzd6 | -1.384398625 | 0.037681311 |
| 393533 | GO:0007199 | G-protein coupled receptor signaling pathway coupled to cGMP nucleotide second messenger | BP | frizzled homolog 6 (Drosophila) | fzd6 | -1.384398625 | 0.037681311 |
| 393533 | GO:0007165 | signal transduction | BP | frizzled homolog 6 (Drosophila) | fzd6 | -1.384398625 | 0.037681311 |
| 393533 | GO:0016055 | Wnt receptor signaling pathway | BP | frizzled homolog 6 (Drosophila) | fzd6 | -1.384398625 | 0.037681311 |
| 393533 | GO:0045177 | apical part of cell | CC | frizzled homolog 6 (Drosophila) | fzd6 | -1.384398625 | 0.037681311 |
| 393533 | GO:0016327 | apicolateral plasma membrane | CC | frizzled homolog 6 (Drosophila) | fzd6 | -1.384398625 | 0.037681311 |
| 393533 | GO:0005737 | cytoplasm | CC | frizzled homolog 6 (Drosophila) | fzd6 | -1.384398625 | 0.037681311 |
| 393533 | GO:0016021 | integral to membrane | CC | frizzled homolog 6 (Drosophila) | fzd6 | -1.384398625 | 0.037681311 |
| 393533 | GO:0016020 | membrane | CC | frizzled homolog 6 (Drosophila) | fzd6 | -1.384398625 | 0.037681311 |
| 393533 | GO:0004930 | G-protein coupled receptor activity | MF | frizzled homolog 6 (Drosophila) | fzd6 | -1.384398625 | 0.037681311 |
| 393533 | GO:0030165 | PDZ domain binding | MF | frizzled homolog 6 (Drosophila) | fzd6 | -1.384398625 | 0.037681311 |
| 393533 | GO:0004871 | signal transducer activity | MF | frizzled homolog 6 (Drosophila) | fzd6 | -1.384398625 | 0.037681311 |
| 393533 | GO:0004888 | transmembrane signaling receptor activity | MF | frizzled homolog 6 (Drosophila) | fzd6 | -1.384398625 | 0.037681311 |
| 393533 | GO:0042813 | Wnt-activated receptor activity | MF | frizzled homolog 6 (Drosophila) | fzd6 | -1.384398625 | 0.037681311 |
| 393533 | GO:0017147 | Wnt-protein binding | MF | frizzled homolog 6 (Drosophila) | fzd6 | -1.384398625 | 0.037681311 |
| 565267 | GO:0016459 | myosin complex | CC | myosin VAb | myo5ab | -1.38406464 | 0.037563223 |
| 565267 | GO:0005524 | ATP binding | MF | myosin VAb | myo5ab | -1.38406464 | 0.037563223 |
| 565267 | GO:0003774 | motor activity | MF | myosin VAb | myo5ab | -1.38406464 | 0.037563223 |
| 565267 | GO:0000166 | nucleotide binding | MF | myosin VAb | myo5ab | -1.38406464 | 0.037563223 |
| 378478 | GO:0016310 | phosphorylation | BP | epidermal growth factor receptor a (erythroblastic leukemia viral (v-erb-b) oncogene homolog, avian) | egfra | -1.381026512 | 0.038054692 |
| 378478 | GO:0006468 | protein phosphorylation | BP | epidermal growth factor receptor a (erythroblastic leukemia viral (v-erb-b) oncogene homolog, avian) | egfra | -1.381026512 | 0.038054692 |
| 378478 | GO:0007169 | transmembrane receptor protein tyrosine kinase signaling pathway | BP | epidermal growth factor receptor a (erythroblastic leukemia viral (v-erb-b) oncogene homolog, avian) | egfra | -1.381026512 | 0.038054692 |
| 378478 | GO:0016021 | integral to membrane | CC | epidermal growth factor receptor a (erythroblastic leukemia viral (v-erb-b) oncogene homolog, avian) | egfra | -1.381026512 | 0.038054692 |
| 378478 | GO:0016020 | membrane | CC | epidermal growth factor receptor a (erythroblastic leukemia viral (v-erb-b) oncogene homolog, avian) | egfra | -1.381026512 | 0.038054692 |
| 378478 | GO:0005524 | ATP binding | MF | epidermal growth factor receptor a (erythroblastic leukemia viral (v-erb-b) oncogene homolog, avian) | egfra | -1.381026512 | 0.038054692 |
| 378478 | GO:0016301 | kinase activity | MF | epidermal growth factor receptor a (erythroblastic leukemia viral (v-erb-b) oncogene homolog, avian) | egfra | -1.381026512 | 0.038054692 |
| 378478 | GO:0000166 | nucleotide binding | MF | epidermal growth factor receptor a (erythroblastic leukemia viral (v-erb-b) oncogene homolog, avian) | egfra | -1.381026512 | 0.038054692 |
| 378478 | GO:0004672 | protein kinase activity | MF | epidermal growth factor receptor a (erythroblastic leukemia viral (v-erb-b) oncogene homolog, avian) | egfra | -1.381026512 | 0.038054692 |
| 378478 | GO:0004713 | protein tyrosine kinase activity | MF | epidermal growth factor receptor a (erythroblastic leukemia viral (v-erb-b) oncogene homolog, avian) | egfra | -1.381026512 | 0.038054692 |
| 378478 | GO:0004716 | receptor signaling protein tyrosine kinase activity | MF | epidermal growth factor receptor a (erythroblastic leukemia viral (v-erb-b) oncogene homolog, avian) | egfra | -1.381026512 | 0.038054692 |
| 378478 | GO:0016740 | transferase activity | MF | epidermal growth factor receptor a (erythroblastic leukemia viral (v-erb-b) oncogene homolog, avian) | egfra | -1.381026512 | 0.038054692 |
| 378478 | GO:0016772 | transferase activity, transferring phosphorus-containing groups | MF | epidermal growth factor receptor a (erythroblastic leukemia viral (v-erb-b) oncogene homolog, avian) | egfra | -1.381026512 | 0.038054692 |
| 378478 | GO:0004714 | transmembrane receptor protein tyrosine kinase activity | MF | epidermal growth factor receptor a (erythroblastic leukemia viral (v-erb-b) oncogene homolog, avian) | egfra | -1.381026512 | 0.038054692 |
| 571937 | GO:0007155 | cell adhesion | BP | EGF-like repeats and discoidin I-like domains 3b | edil3b | -1.380735527 | 0.039416391 |
| 571937 | GO:0005509 | calcium ion binding | MF | EGF-like repeats and discoidin I-like domains 3b | edil3b | -1.380735527 | 0.039416391 |
| 431761 | GO:0008150 | biological_process | BP | transcription factor CP2-like 1 | tfcp2l1 | -1.37886305 | 0.039305837 |
| 431761 | GO:0005575 | cellular_component | CC | transcription factor CP2-like 1 | tfcp2l1 | -1.37886305 | 0.039305837 |
| 431761 | GO:0003674 | molecular_function | MF | transcription factor CP2-like 1 | tfcp2l1 | -1.37886305 | 0.039305837 |
| 445087 | GO:0008150 | biological_process | BP | zgc:91811 | zgc:91811 | -1.377318884 | 0.039581622 |
| 445087 | GO:0005575 | cellular_component | CC | zgc:91811 | zgc:91811 | -1.377318884 | 0.039581622 |
| 445087 | GO:0003674 | molecular_function | MF | zgc:91811 | zgc:91811 | -1.377318884 | 0.039581622 |
| 368847 | GO:0016310 | phosphorylation | BP | triple functional domain (PTPRF interacting) | trio | -1.368197392 | 0.039841852 |
| 368847 | GO:0006468 | protein phosphorylation | BP | triple functional domain (PTPRF interacting) | trio | -1.368197392 | 0.039841852 |
| 368847 | GO:0035023 | regulation of Rho protein signal transduction | BP | triple functional domain (PTPRF interacting) | trio | -1.368197392 | 0.039841852 |
| 368847 | GO:0005622 | intracellular | CC | triple functional domain (PTPRF interacting) | trio | -1.368197392 | 0.039841852 |
| 368847 | GO:0005524 | ATP binding | MF | triple functional domain (PTPRF interacting) | trio | -1.368197392 | 0.039841852 |
| 368847 | GO:0016301 | kinase activity | MF | triple functional domain (PTPRF interacting) | trio | -1.368197392 | 0.039841852 |
| 368847 | GO:0000166 | nucleotide binding | MF | triple functional domain (PTPRF interacting) | trio | -1.368197392 | 0.039841852 |
| 368847 | GO:0005543 | phospholipid binding | MF | triple functional domain (PTPRF interacting) | trio | -1.368197392 | 0.039841852 |
| 368847 | GO:0004672 | protein kinase activity | MF | triple functional domain (PTPRF interacting) | trio | -1.368197392 | 0.039841852 |
| 368847 | GO:0004674 | protein serine/threonine kinase activity | MF | triple functional domain (PTPRF interacting) | trio | -1.368197392 | 0.039841852 |
| 368847 | GO:0005089 | Rho guanyl-nucleotide exchange factor activity | MF | triple functional domain (PTPRF interacting) | trio | -1.368197392 | 0.039841852 |
| 368847 | GO:0016740 | transferase activity | MF | triple functional domain (PTPRF interacting) | trio | -1.368197392 | 0.039841852 |
| 368847 | GO:0016772 | transferase activity, transferring phosphorus-containing groups | MF | triple functional domain (PTPRF interacting) | trio | -1.368197392 | 0.039841852 |
| 337596 | GO:0072488 | ammonium transmembrane transport | BP | Rhesus blood group, B glycoprotein | rhbg | -1.364598939 | 0.040591667 |
| 337596 | GO:0015696 | ammonium transport | BP | Rhesus blood group, B glycoprotein | rhbg | -1.364598939 | 0.040591667 |
| 337596 | GO:0015695 | organic cation transport | BP | Rhesus blood group, B glycoprotein | rhbg | -1.364598939 | 0.040591667 |
| 337596 | GO:0006810 | transport | BP | Rhesus blood group, B glycoprotein | rhbg | -1.364598939 | 0.040591667 |
| 337596 | GO:0016323 | basolateral plasma membrane | CC | Rhesus blood group, B glycoprotein | rhbg | -1.364598939 | 0.040591667 |
| 337596 | GO:0031410 | cytoplasmic vesicle | CC | Rhesus blood group, B glycoprotein | rhbg | -1.364598939 | 0.040591667 |
| 337596 | GO:0030659 | cytoplasmic vesicle membrane | CC | Rhesus blood group, B glycoprotein | rhbg | -1.364598939 | 0.040591667 |
| 337596 | GO:0016021 | integral to membrane | CC | Rhesus blood group, B glycoprotein | rhbg | -1.364598939 | 0.040591667 |
| 337596 | GO:0016020 | membrane | CC | Rhesus blood group, B glycoprotein | rhbg | -1.364598939 | 0.040591667 |
| 337596 | GO:0005886 | plasma membrane | CC | Rhesus blood group, B glycoprotein | rhbg | -1.364598939 | 0.040591667 |
| 337596 | GO:0008519 | ammonium transmembrane transporter activity | MF | Rhesus blood group, B glycoprotein | rhbg | -1.364598939 | 0.040591667 |
| 405784 | GO:0006355 | regulation of transcription, DNA-dependent | BP | runt-related transcription factor 2a | runx2a | -1.362501271 | 0.041376428 |
| 405784 | GO:0005634 | nucleus | CC | runt-related transcription factor 2a | runx2a | -1.362501271 | 0.041376428 |
| 405784 | GO:0005524 | ATP binding | MF | runt-related transcription factor 2a | runx2a | -1.362501271 | 0.041376428 |
| 405784 | GO:0003677 | DNA binding | MF | runt-related transcription factor 2a | runx2a | -1.362501271 | 0.041376428 |
| 405784 | GO:0003700 | sequence-specific DNA binding transcription factor activity | MF | runt-related transcription factor 2a | runx2a | -1.362501271 | 0.041376428 |
| 327288 | GO:0008152 | metabolic process | BP | CNDP dipeptidase 2 (metallopeptidase M20 family) | cndp2 | -1.362334846 | 0.042149665 |
| 327288 | GO:0006508 | proteolysis | BP | CNDP dipeptidase 2 (metallopeptidase M20 family) | cndp2 | -1.362334846 | 0.042149665 |
| 327288 | GO:0005575 | cellular_component | CC | CNDP dipeptidase 2 (metallopeptidase M20 family) | cndp2 | -1.362334846 | 0.042149665 |
| 327288 | GO:0016805 | dipeptidase activity | MF | CNDP dipeptidase 2 (metallopeptidase M20 family) | cndp2 | -1.362334846 | 0.042149665 |
| 327288 | GO:0016787 | hydrolase activity | MF | CNDP dipeptidase 2 (metallopeptidase M20 family) | cndp2 | -1.362334846 | 0.042149665 |
| 327288 | GO:0008237 | metallopeptidase activity | MF | CNDP dipeptidase 2 (metallopeptidase M20 family) | cndp2 | -1.362334846 | 0.042149665 |
| 327288 | GO:0034701 | tripeptidase activity | MF | CNDP dipeptidase 2 (metallopeptidase M20 family) | cndp2 | -1.362334846 | 0.042149665 |
| 393195 | GO:0016070 | RNA metabolic process | BP | ribonuclease H2, subunit A | rnaseh2a | -1.360054771 | 0.042059781 |
| 393195 | GO:0004519 | endonuclease activity | MF | ribonuclease H2, subunit A | rnaseh2a | -1.360054771 | 0.042059781 |
| 393195 | GO:0016787 | hydrolase activity | MF | ribonuclease H2, subunit A | rnaseh2a | -1.360054771 | 0.042059781 |
| 393195 | GO:0004518 | nuclease activity | MF | ribonuclease H2, subunit A | rnaseh2a | -1.360054771 | 0.042059781 |
| 393195 | GO:0003676 | nucleic acid binding | MF | ribonuclease H2, subunit A | rnaseh2a | -1.360054771 | 0.042059781 |
| 393195 | GO:0004523 | ribonuclease H activity | MF | ribonuclease H2, subunit A | rnaseh2a | -1.360054771 | 0.042059781 |
| 393195 | GO:0003723 | RNA binding | MF | ribonuclease H2, subunit A | rnaseh2a | -1.360054771 | 0.042059781 |
| 794932 | GO:0005509 | calcium ion binding | MF | dystrobrevin, alpha | dtna | -1.359765956 | 0.042310814 |
| 794932 | GO:0008270 | zinc ion binding | MF | dystrobrevin, alpha | dtna | -1.359765956 | 0.042310814 |
| 415203 | GO:0045454 | cell redox homeostasis | BP | thioredoxin-related transmembrane protein 2b | tmx2b | -1.358573929 | 0.041599131 |
| 415203 | GO:0016021 | integral to membrane | CC | thioredoxin-related transmembrane protein 2b | tmx2b | -1.358573929 | 0.041599131 |
| 415203 | GO:0016020 | membrane | CC | thioredoxin-related transmembrane protein 2b | tmx2b | -1.358573929 | 0.041599131 |
| 393810 | GO:0005575 | cellular_component | CC | zgc:73359 | zgc:73359 | -1.354944637 | 0.042816879 |
| 393810 | GO:0004114 | 3',5'-cyclic-nucleotide phosphodiesterase activity | MF | zgc:73359 | zgc:73359 | -1.354944637 | 0.042816879 |
| 393810 | GO:0030553 | cGMP binding | MF | zgc:73359 | zgc:73359 | -1.354944637 | 0.042816879 |
| 445126 | GO:0018208 | peptidyl-proline modification | BP | FK506 binding protein 9 | fkbp9 | -1.354842467 | 0.041954341 |
| 445126 | GO:0006457 | protein folding | BP | FK506 binding protein 9 | fkbp9 | -1.354842467 | 0.041954341 |
| 445126 | GO:0000413 | protein peptidyl-prolyl isomerization | BP | FK506 binding protein 9 | fkbp9 | -1.354842467 | 0.041954341 |
| 445126 | GO:0016020 | membrane | CC | FK506 binding protein 9 | fkbp9 | -1.354842467 | 0.041954341 |
| 445126 | GO:0005509 | calcium ion binding | MF | FK506 binding protein 9 | fkbp9 | -1.354842467 | 0.041954341 |
| 445126 | GO:0005528 | FK506 binding | MF | FK506 binding protein 9 | fkbp9 | -1.354842467 | 0.041954341 |
| 445126 | GO:0003755 | peptidyl-prolyl cis-trans isomerase activity | MF | FK506 binding protein 9 | fkbp9 | -1.354842467 | 0.041954341 |
| 336335 | GO:0008150 | biological_process | BP | si:ch211-51e12.7 | si:ch211-51e12.7 | -1.35382557 | 0.042298194 |
| 336335 | GO:0005575 | cellular_component | CC | si:ch211-51e12.7 | si:ch211-51e12.7 | -1.35382557 | 0.042298194 |
| 336335 | GO:0003674 | molecular_function | MF | si:ch211-51e12.7 | si:ch211-51e12.7 | -1.35382557 | 0.042298194 |
| 415175 | GO:0005575 | cellular_component | CC | troponin C type 1b (slow) | tnnc1b | -1.348973459 | 0.047420371 |
| 415175 | GO:0005509 | calcium ion binding | MF | troponin C type 1b (slow) | tnnc1b | -1.348973459 | 0.047420371 |
| 767718 | GO:0005575 | cellular_component | CC | zgc:153723 | zgc:153723 | -1.348121825 | 0.043245744 |
| 767718 | GO:0008146 | sulfotransferase activity | MF | zgc:153723 | zgc:153723 | -1.348121825 | 0.043245744 |
| 30523 | GO:0060070 | canonical Wnt receptor signaling pathway | BP | transcription factor 7-like 1a (T-cell specific, HMG-box) | tcf7l1a | -1.347801819 | 0.043049595 |
| 30523 | GO:0030178 | negative regulation of Wnt receptor signaling pathway | BP | transcription factor 7-like 1a (T-cell specific, HMG-box) | tcf7l1a | -1.347801819 | 0.043049595 |
| 30523 | GO:0008284 | positive regulation of cell proliferation | BP | transcription factor 7-like 1a (T-cell specific, HMG-box) | tcf7l1a | -1.347801819 | 0.043049595 |
| 30523 | GO:0006357 | regulation of transcription from RNA polymerase II promoter | BP | transcription factor 7-like 1a (T-cell specific, HMG-box) | tcf7l1a | -1.347801819 | 0.043049595 |
| 30523 | GO:0006355 | regulation of transcription, DNA-dependent | BP | transcription factor 7-like 1a (T-cell specific, HMG-box) | tcf7l1a | -1.347801819 | 0.043049595 |
| 30523 | GO:0006351 | transcription, DNA-dependent | BP | transcription factor 7-like 1a (T-cell specific, HMG-box) | tcf7l1a | -1.347801819 | 0.043049595 |
| 30523 | GO:0016055 | Wnt receptor signaling pathway | BP | transcription factor 7-like 1a (T-cell specific, HMG-box) | tcf7l1a | -1.347801819 | 0.043049595 |
| 30523 | GO:0005634 | nucleus | CC | transcription factor 7-like 1a (T-cell specific, HMG-box) | tcf7l1a | -1.347801819 | 0.043049595 |
| 30523 | GO:0005667 | transcription factor complex | CC | transcription factor 7-like 1a (T-cell specific, HMG-box) | tcf7l1a | -1.347801819 | 0.043049595 |
| 30523 | GO:0008013 | beta-catenin binding | MF | transcription factor 7-like 1a (T-cell specific, HMG-box) | tcf7l1a | -1.347801819 | 0.043049595 |
| 30523 | GO:0003682 | chromatin binding | MF | transcription factor 7-like 1a (T-cell specific, HMG-box) | tcf7l1a | -1.347801819 | 0.043049595 |
| 30523 | GO:0003677 | DNA binding | MF | transcription factor 7-like 1a (T-cell specific, HMG-box) | tcf7l1a | -1.347801819 | 0.043049595 |
| 30523 | GO:0043565 | sequence-specific DNA binding | MF | transcription factor 7-like 1a (T-cell specific, HMG-box) | tcf7l1a | -1.347801819 | 0.043049595 |
| 30523 | GO:0003700 | sequence-specific DNA binding transcription factor activity | MF | transcription factor 7-like 1a (T-cell specific, HMG-box) | tcf7l1a | -1.347801819 | 0.043049595 |
| 30523 | GO:0044212 | transcription regulatory region DNA binding | MF | transcription factor 7-like 1a (T-cell specific, HMG-box) | tcf7l1a | -1.347801819 | 0.043049595 |
| 794647 | GO:0007186 | G-protein coupled receptor signaling pathway | BP | trace amine-associated receptor 11 | taar11 | -1.345365007 | 0.043964472 |
| 794647 | GO:0007165 | signal transduction | BP | trace amine-associated receptor 11 | taar11 | -1.345365007 | 0.043964472 |
| 794647 | GO:0016021 | integral to membrane | CC | trace amine-associated receptor 11 | taar11 | -1.345365007 | 0.043964472 |
| 794647 | GO:0004930 | G-protein coupled receptor activity | MF | trace amine-associated receptor 11 | taar11 | -1.345365007 | 0.043964472 |
| 794647 | GO:0004871 | signal transducer activity | MF | trace amine-associated receptor 11 | taar11 | -1.345365007 | 0.043964472 |
| 794647 | GO:0001594 | trace-amine receptor activity | MF | trace amine-associated receptor 11 | taar11 | -1.345365007 | 0.043964472 |
| 100038802 | GO:0006810 | transport | BP | solute carrier organic anion transporter family, member 2A1 | slco2a1 | -1.34462902 | 0.044283292 |
| 100038802 | GO:0016020 | membrane | CC | solute carrier organic anion transporter family, member 2A1 | slco2a1 | -1.34462902 | 0.044283292 |
| 100038802 | GO:0005215 | transporter activity | MF | solute carrier organic anion transporter family, member 2A1 | slco2a1 | -1.34462902 | 0.044283292 |
| 566832 | GO:0006508 | proteolysis | BP | dipeptidyl-peptidase 6b | dpp6b | -1.342091727 | 0.043929217 |
| 566832 | GO:0016020 | membrane | CC | dipeptidyl-peptidase 6b | dpp6b | -1.342091727 | 0.043929217 |
| 566832 | GO:0008236 | serine-type peptidase activity | MF | dipeptidyl-peptidase 6b | dpp6b | -1.342091727 | 0.043929217 |
| 100170776 | GO:0008150 | biological_process | BP | zgc:194443 | zgc:194443 | -1.34031507 | 0.04465091 |
| 100170776 | GO:0005575 | cellular_component | CC | zgc:194443 | zgc:194443 | -1.34031507 | 0.04465091 |
| 100170776 | GO:0005525 | GTP binding | MF | zgc:194443 | zgc:194443 | -1.34031507 | 0.04465091 |
| 322451 | GO:0006508 | proteolysis | BP | chymotrypsinogen B1 | ctrb1 | -1.335327306 | 0.04701256 |
| 322451 | GO:0003824 | catalytic activity | MF | chymotrypsinogen B1 | ctrb1 | -1.335327306 | 0.04701256 |
| 322451 | GO:0016787 | hydrolase activity | MF | chymotrypsinogen B1 | ctrb1 | -1.335327306 | 0.04701256 |
| 322451 | GO:0008233 | peptidase activity | MF | chymotrypsinogen B1 | ctrb1 | -1.335327306 | 0.04701256 |
| 322451 | GO:0004252 | serine-type endopeptidase activity | MF | chymotrypsinogen B1 | ctrb1 | -1.335327306 | 0.04701256 |
| 322451 | GO:0008236 | serine-type peptidase activity | MF | chymotrypsinogen B1 | ctrb1 | -1.335327306 | 0.04701256 |
| 30270 | GO:0005576 | extracellular region | CC | neurotrophin 7 | ntf7 | -1.335233352 | 0.045107946 |
| 30270 | GO:0008083 | growth factor activity | MF | neurotrophin 7 | ntf7 | -1.335233352 | 0.045107946 |
| 30270 | GO:0005102 | receptor binding | MF | neurotrophin 7 | ntf7 | -1.335233352 | 0.045107946 |
| 402944 | GO:0008150 | biological_process | BP | zgc:77929 | zgc:77929 | -1.334332149 | 0.045185721 |
| 402944 | GO:0005576 | extracellular region | CC | zgc:77929 | zgc:77929 | -1.334332149 | 0.045185721 |
| 402944 | GO:0003674 | molecular_function | MF | zgc:77929 | zgc:77929 | -1.334332149 | 0.045185721 |
| 30647 | GO:0008537 | proteasome activator complex | CC | proteasome activator subunit 2 | psme2 | -1.328212392 | 0.04592894 |
| 30647 | GO:0000502 | proteasome complex | CC | proteasome activator subunit 2 | psme2 | -1.328212392 | 0.04592894 |
| 327079 | GO:0006355 | regulation of transcription, DNA-dependent | BP | sap30-like | sap30l | -1.312892327 | 0.049893273 |
| 327079 | GO:0006351 | transcription, DNA-dependent | BP | sap30-like | sap30l | -1.312892327 | 0.049893273 |
| 327079 | GO:0005634 | nucleus | CC | sap30-like | sap30l | -1.312892327 | 0.049893273 |
| 327079 | GO:0003677 | DNA binding | MF | sap30-like | sap30l | -1.312892327 | 0.049893273 |
| 327079 | GO:0046872 | metal ion binding | MF | sap30-like | sap30l | -1.312892327 | 0.049893273 |
| 563147 | GO:0016310 | phosphorylation | BP | protein kinase, cAMP-dependent, catalytic, beta b | prkacbb | -1.310965205 | 0.048879854 |
| 563147 | GO:0006468 | protein phosphorylation | BP | protein kinase, cAMP-dependent, catalytic, beta b | prkacbb | -1.310965205 | 0.048879854 |
| 563147 | GO:0005575 | cellular_component | CC | protein kinase, cAMP-dependent, catalytic, beta b | prkacbb | -1.310965205 | 0.048879854 |
| 563147 | GO:0005524 | ATP binding | MF | protein kinase, cAMP-dependent, catalytic, beta b | prkacbb | -1.310965205 | 0.048879854 |
| 563147 | GO:0016301 | kinase activity | MF | protein kinase, cAMP-dependent, catalytic, beta b | prkacbb | -1.310965205 | 0.048879854 |
| 563147 | GO:0000166 | nucleotide binding | MF | protein kinase, cAMP-dependent, catalytic, beta b | prkacbb | -1.310965205 | 0.048879854 |
| 563147 | GO:0004672 | protein kinase activity | MF | protein kinase, cAMP-dependent, catalytic, beta b | prkacbb | -1.310965205 | 0.048879854 |
| 563147 | GO:0004674 | protein serine/threonine kinase activity | MF | protein kinase, cAMP-dependent, catalytic, beta b | prkacbb | -1.310965205 | 0.048879854 |
| 563147 | GO:0016740 | transferase activity | MF | protein kinase, cAMP-dependent, catalytic, beta b | prkacbb | -1.310965205 | 0.048879854 |
| 563147 | GO:0016772 | transferase activity, transferring phosphorus-containing groups | MF | protein kinase, cAMP-dependent, catalytic, beta b | prkacbb | -1.310965205 | 0.048879854 |
| 571664 | GO:0005509 | calcium ion binding | MF | follistatin-like 4 | fstl4 | -1.309002103 | 0.04901425 |
