# Supplementary material for: Functional Categorization of Transcriptome in the Species Symphysodon aequifasciatus Pellegrin 1904 (Perciformes: Cichlidae) Exposed to Benzo[a]pyrene and Phenanthrene
Source: PLoS One. 2013 Dec 3;8(12):e81083. doi: 10.1371/journal.pone.0081083 (PMC3849039; doi:10.1371/journal.pone.0081083)
Supplement: Table S2 — Up-regulated genes in Symphysodon aequifasciatus exposed to benzo[a]pyrene and phenanthrene for 48 h. Only genes for which information regarding gene function is currently available in the Gene Ontology (AmiGO v. 1.8) are reported here. Where multiple terms have been identified for a gene, only those most relevant in the context of this study are reported. Genes are ranked according to Fold Change compared to control group (Log2FC). gene_id, gene_name and symbol are according to GenBank (National Center for Biotechnology Information). (BP = Biological Process; CC = Cellular Component; MF = Molecular Function). (DOC) [file pone.0081083.s007.doc]

Table S2. Up-regulated genes in *Symphysodon aequifasciatus* exposed to benzo[a]pyrene and phenanthrene for 48h. Only genes for which information regarding gene function is currently available in the Gene Ontology (AmiGO v. 1.8) are reported here. Where multiple terms have been identified for a gene, only those most relevant in the context of this study are reported. Genes are ranked according to Fold Change compared to control group (Log_2_FC). gene_id, gene_name and symbol are according to GenBank (National Center for Biotechnology Information). (BP=Biological Process; CC=Cellular Component; MF=Molecular Function).

| gene_id | go_id | Gene Ontology term | Ontology | gene_name | symbol | log_2_FC | PValue |
| --- | --- | --- | --- | --- | --- | --- | --- |
| 569530 | GO:0007049 | cell cycle | BP | endosulfine alpha a | ensaa | 5.67491056 | 2.61773E-12 |
| 569530 | GO:0051301 | cell division | BP | endosulfine alpha a | ensaa | 5.67491056 | 2.61773E-12 |
| 569530 | GO:0000086 | G2/M transition of mitotic cell cycle | BP | endosulfine alpha a | ensaa | 5.67491056 | 2.61773E-12 |
| 569530 | GO:0007067 | mitosis | BP | endosulfine alpha a | ensaa | 5.67491056 | 2.61773E-12 |
| 569530 | GO:0005737 | cytoplasm | CC | endosulfine alpha a | ensaa | 5.67491056 | 2.61773E-12 |
| 569530 | GO:0019212 | phosphatase inhibitor activity | MF | endosulfine alpha a | ensaa | 5.67491056 | 2.61773E-12 |
| 569530 | GO:0051721 | protein phosphatase 2A binding | MF | endosulfine alpha a | ensaa | 5.67491056 | 2.61773E-12 |
| 569530 | GO:0004864 | protein phosphatase inhibitor activity | MF | endosulfine alpha a | ensaa | 5.67491056 | 2.61773E-12 |
| 569530 | GO:0008601 | protein phosphatase type 2A regulator activity | MF | endosulfine alpha a | ensaa | 5.67491056 | 2.61773E-12 |
| 100150682 | GO:0008150 | biological_process | BP | zgc:173615 | zgc:173615 | 4.988680112 | 9.37908E-09 |
| 100150682 | GO:0005622 | intracellular | CC | zgc:173615 | zgc:173615 | 4.988680112 | 9.37908E-09 |
| 100150682 | GO:0003676 | nucleic acid binding | MF | zgc:173615 | zgc:173615 | 4.988680112 | 9.37908E-09 |
| 100150682 | GO:0008270 | zinc ion binding | MF | zgc:173615 | zgc:173615 | 4.988680112 | 9.37908E-09 |
| 100150158 | GO:0007186 | G-protein coupled receptor signaling pathway | BP | odorant receptor, family E, subfamily 124, member 1 | or124-1 | 4.597089777 | 2.79704E-08 |
| 100150158 | GO:0042574 | retinal metabolic process | BP | odorant receptor, family E, subfamily 124, member 1 | or124-1 | 4.597089777 | 2.79704E-08 |
| 100150158 | GO:0007165 | signal transduction | BP | odorant receptor, family E, subfamily 124, member 1 | or124-1 | 4.597089777 | 2.79704E-08 |
| 100150158 | GO:0016021 | integral to membrane | CC | odorant receptor, family E, subfamily 124, member 1 | or124-1 | 4.597089777 | 2.79704E-08 |
| 100150158 | GO:0016020 | membrane | CC | odorant receptor, family E, subfamily 124, member 1 | or124-1 | 4.597089777 | 2.79704E-08 |
| 100150158 | GO:0005886 | plasma membrane | CC | odorant receptor, family E, subfamily 124, member 1 | or124-1 | 4.597089777 | 2.79704E-08 |
| 100150158 | GO:0004930 | G-protein coupled receptor activity | MF | odorant receptor, family E, subfamily 124, member 1 | or124-1 | 4.597089777 | 2.79704E-08 |
| 100150158 | GO:0004871 | signal transducer activity | MF | odorant receptor, family E, subfamily 124, member 1 | or124-1 | 4.597089777 | 2.79704E-08 |
| 192331 | GO:0030529 | ribonucleoprotein complex | CC | small nuclear ribonucleoprotein D1 polypeptide | snrpd1 | 4.28377065 | 1.05604E-08 |
| 192331 | GO:0003676 | nucleic acid binding | MF | small nuclear ribonucleoprotein D1 polypeptide | snrpd1 | 4.28377065 | 1.05604E-08 |
| 378872 | GO:0008150 | biological_process | BP | zinc finger protein 395 | znf395 | 3.443446351 | 4.2888E-06 |
| 378872 | GO:0003674 | molecular_function | MF | zinc finger protein 395 | znf395 | 3.443446351 | 4.2888E-06 |
| 797137 | GO:0005634 | nucleus | CC | immediate early response 2 | ier2 | 3.410386771 | 2.17003E-06 |
| 797137 | GO:0005515 | protein binding | MF | immediate early response 2 | ier2 | 3.410386771 | 2.17003E-06 |
| 798203 | GO:0008150 | biological_process | BP | zgc:162962 | zgc:162962 | 3.095318977 | 2.30814E-05 |
| 798203 | GO:0005622 | intracellular | CC | zgc:162962 | zgc:162962 | 3.095318977 | 2.30814E-05 |
| 798203 | GO:0003676 | nucleic acid binding | MF | zgc:162962 | zgc:162962 | 3.095318977 | 2.30814E-05 |
| 798203 | GO:0008270 | zinc ion binding | MF | zgc:162962 | zgc:162962 | 3.095318977 | 2.30814E-05 |
| 553537 | GO:0055114 | oxidation-reduction process | BP | cytochrome P450, family 2, subfamily AA, polypeptide 11 | cyp2aa11 | 3.016759522 | 2.53588E-05 |
| 553537 | GO:0009055 | electron carrier activity | MF | cytochrome P450, family 2, subfamily AA, polypeptide 11 | cyp2aa11 | 3.016759522 | 2.53588E-05 |
| 553537 | GO:0020037 | heme binding | MF | cytochrome P450, family 2, subfamily AA, polypeptide 11 | cyp2aa11 | 3.016759522 | 2.53588E-05 |
| 553537 | GO:0005506 | iron ion binding | MF | cytochrome P450, family 2, subfamily AA, polypeptide 11 | cyp2aa11 | 3.016759522 | 2.53588E-05 |
| 553537 | GO:0046872 | metal ion binding | MF | cytochrome P450, family 2, subfamily AA, polypeptide 11 | cyp2aa11 | 3.016759522 | 2.53588E-05 |
| 553537 | GO:0004497 | monooxygenase activity | MF | cytochrome P450, family 2, subfamily AA, polypeptide 11 | cyp2aa11 | 3.016759522 | 2.53588E-05 |
| 553537 | GO:0016491 | oxidoreductase activity | MF | cytochrome P450, family 2, subfamily AA, polypeptide 11 | cyp2aa11 | 3.016759522 | 2.53588E-05 |
| 553537 | GO:0016705 | oxidoreductase activity, acting on paired donors, with incorporation or reduction of molecular oxygen | MF | cytochrome P450, family 2, subfamily AA, polypeptide 11 | cyp2aa11 | 3.016759522 | 2.53588E-05 |
| 322415 | GO:0008146 | sulfotransferase activity | MF | sulfotransferase family 3, cytosolic sulfotransferase 2 | sult3st2 | 2.706559989 | 0.000122868 |
| 322415 | GO:0016740 | transferase activity | MF | sulfotransferase family 3, cytosolic sulfotransferase 2 | sult3st2 | 2.706559989 | 0.000122868 |
| 100037361 | GO:0008150 | biological_process | BP | zgc:158463 | zgc:158463 | 2.646795804 | 0.000139226 |
| 100037361 | GO:0005575 | cellular_component | CC | zgc:158463 | zgc:158463 | 2.646795804 | 0.000139226 |
| 100037361 | GO:0003674 | molecular_function | MF | zgc:158463 | zgc:158463 | 2.646795804 | 0.000139226 |
| 30219 | GO:0001525 | angiogenesis | BP | ephrin B2a | efnb2a | 2.600165752 | 0.000174487 |
| 30219 | GO:0007155 | cell adhesion | BP | ephrin B2a | efnb2a | 2.600165752 | 0.000174487 |
| 30219 | GO:0030154 | cell differentiation | BP | ephrin B2a | efnb2a | 2.600165752 | 0.000174487 |
| 30219 | GO:0048013 | ephrin receptor signaling pathway | BP | ephrin B2a | efnb2a | 2.600165752 | 0.000174487 |
| 30219 | GO:0050920 | regulation of chemotaxis | BP | ephrin B2a | efnb2a | 2.600165752 | 0.000174487 |
| 30219 | GO:0016021 | integral to membrane | CC | ephrin B2a | efnb2a | 2.600165752 | 0.000174487 |
| 30219 | GO:0016020 | membrane | CC | ephrin B2a | efnb2a | 2.600165752 | 0.000174487 |
| 100137104 | GO:0005622 | intracellular | CC | zgc:173581 | zgc:173581 | 2.475114038 | 0.000338041 |
| 100137104 | GO:0046872 | metal ion binding | MF | zgc:173581 | zgc:173581 | 2.475114038 | 0.000338041 |
| 100137104 | GO:0008270 | zinc ion binding | MF | zgc:173581 | zgc:173581 | 2.475114038 | 0.000338041 |
| 81595 | GO:0008150 | biological_process | BP | B-cell CLL/lymphoma 7B, b | bcl7bb | 2.40455361 | 0.000474163 |
| 81595 | GO:0005575 | cellular_component | CC | B-cell CLL/lymphoma 7B, b | bcl7bb | 2.40455361 | 0.000474163 |
| 81595 | GO:0003674 | molecular_function | MF | B-cell CLL/lymphoma 7B, b | bcl7bb | 2.40455361 | 0.000474163 |
| 445056 | GO:0006508 | proteolysis | BP | zgc:92511 | zgc:92511 | 2.353544799 | 0.000628666 |
| 445056 | GO:0005575 | cellular_component | CC | zgc:92511 | zgc:92511 | 2.353544799 | 0.000628666 |
| 445056 | GO:0003824 | catalytic activity | MF | zgc:92511 | zgc:92511 | 2.353544799 | 0.000628666 |
| 445056 | GO:0004252 | serine-type endopeptidase activity | MF | zgc:92511 | zgc:92511 | 2.353544799 | 0.000628666 |
| 406327 | GO:0030168 | platelet activation | BP | fibrinogen, gamma polypeptide | fgg | 2.333170517 | 0.00066722 |
| 406327 | GO:0051258 | protein polymerization | BP | fibrinogen, gamma polypeptide | fgg | 2.333170517 | 0.00066722 |
| 406327 | GO:0007165 | signal transduction | BP | fibrinogen, gamma polypeptide | fgg | 2.333170517 | 0.00066722 |
| 406327 | GO:0005615 | extracellular space | CC | fibrinogen, gamma polypeptide | fgg | 2.333170517 | 0.00066722 |
| 406327 | GO:0005577 | fibrinogen complex | CC | fibrinogen, gamma polypeptide | fgg | 2.333170517 | 0.00066722 |
| 406327 | GO:0030674 | protein binding, bridging | MF | fibrinogen, gamma polypeptide | fgg | 2.333170517 | 0.00066722 |
| 406327 | GO:0005102 | receptor binding | MF | fibrinogen, gamma polypeptide | fgg | 2.333170517 | 0.00066722 |
| 449545 | GO:0005575 | cellular_component | CC | carnitine O-acetyltransferase a | crata | 2.29117123 | 0.000841642 |
| 449545 | GO:0016740 | transferase activity | MF | carnitine O-acetyltransferase a | crata | 2.29117123 | 0.000841642 |
| 449545 | GO:0016746 | transferase activity, transferring acyl groups | MF | carnitine O-acetyltransferase a | crata | 2.29117123 | 0.000841642 |
| 30486 | GO:0045892 | negative regulation of transcription, DNA-dependent | BP | retinoid x receptor, beta b | rxrbb | 2.18890425 | 0.001362907 |
| 30486 | GO:0006355 | regulation of transcription, DNA-dependent | BP | retinoid x receptor, beta b | rxrbb | 2.18890425 | 0.001362907 |
| 30486 | GO:0043401 | steroid hormone mediated signaling pathway | BP | retinoid x receptor, beta b | rxrbb | 2.18890425 | 0.001362907 |
| 30486 | GO:0006351 | transcription, DNA-dependent | BP | retinoid x receptor, beta b | rxrbb | 2.18890425 | 0.001362907 |
| 30486 | GO:0005634 | nucleus | CC | retinoid x receptor, beta b | rxrbb | 2.18890425 | 0.001362907 |
| 30486 | GO:0003677 | DNA binding | MF | retinoid x receptor, beta b | rxrbb | 2.18890425 | 0.001362907 |
| 30486 | GO:0046872 | metal ion binding | MF | retinoid x receptor, beta b | rxrbb | 2.18890425 | 0.001362907 |
| 30486 | GO:0001071 | nucleic acid binding transcription factor activity | MF | retinoid x receptor, beta b | rxrbb | 2.18890425 | 0.001362907 |
| 30486 | GO:0043565 | sequence-specific DNA binding | MF | retinoid x receptor, beta b | rxrbb | 2.18890425 | 0.001362907 |
| 30486 | GO:0003700 | sequence-specific DNA binding transcription factor activity | MF | retinoid x receptor, beta b | rxrbb | 2.18890425 | 0.001362907 |
| 30486 | GO:0003707 | steroid hormone receptor activity | MF | retinoid x receptor, beta b | rxrbb | 2.18890425 | 0.001362907 |
| 30486 | GO:0008270 | zinc ion binding | MF | retinoid x receptor, beta b | rxrbb | 2.18890425 | 0.001362907 |
| 30639 | GO:0035556 | intracellular signal transduction | BP | MAD homolog 2 (Drosophila) | smad2 | 2.171419086 | 0.001477359 |
| 30639 | GO:0045892 | negative regulation of transcription, DNA-dependent | BP | MAD homolog 2 (Drosophila) | smad2 | 2.171419086 | 0.001477359 |
| 30639 | GO:0006355 | regulation of transcription, DNA-dependent | BP | MAD homolog 2 (Drosophila) | smad2 | 2.171419086 | 0.001477359 |
| 30639 | GO:0006351 | transcription, DNA-dependent | BP | MAD homolog 2 (Drosophila) | smad2 | 2.171419086 | 0.001477359 |
| 30639 | GO:0007179 | transforming growth factor beta receptor signaling pathway | BP | MAD homolog 2 (Drosophila) | smad2 | 2.171419086 | 0.001477359 |
| 30639 | GO:0005737 | cytoplasm | CC | MAD homolog 2 (Drosophila) | smad2 | 2.171419086 | 0.001477359 |
| 30639 | GO:0005622 | intracellular | CC | MAD homolog 2 (Drosophila) | smad2 | 2.171419086 | 0.001477359 |
| 30639 | GO:0005634 | nucleus | CC | MAD homolog 2 (Drosophila) | smad2 | 2.171419086 | 0.001477359 |
| 30639 | GO:0005667 | transcription factor complex | CC | MAD homolog 2 (Drosophila) | smad2 | 2.171419086 | 0.001477359 |
| 30639 | GO:0003700 | sequence-specific DNA binding transcription factor activity | MF | MAD homolog 2 (Drosophila) | smad2 | 2.171419086 | 0.001477359 |
| 322104 | GO:0007165 | signal transduction | BP | guanine nucleotide binding protein (G protein), beta polypeptide 1a | gnb1a | 2.136396993 | 0.001728734 |
| 322104 | GO:0004871 | signal transducer activity | MF | guanine nucleotide binding protein (G protein), beta polypeptide 1a | gnb1a | 2.136396993 | 0.001728734 |
| 554119 | GO:0046983 | protein dimerization activity | MF | MAX-like protein X | mlx | 2.114220577 | 0.002033443 |
| 334956 | GO:0006396 | RNA processing | BP | La ribonucleoprotein domain family, member 7 | larp7 | 2.110121458 | 0.002051897 |
| 334956 | GO:0005634 | nucleus | CC | La ribonucleoprotein domain family, member 7 | larp7 | 2.110121458 | 0.002051897 |
| 334956 | GO:0030529 | ribonucleoprotein complex | CC | La ribonucleoprotein domain family, member 7 | larp7 | 2.110121458 | 0.002051897 |
| 334956 | GO:0003676 | nucleic acid binding | MF | La ribonucleoprotein domain family, member 7 | larp7 | 2.110121458 | 0.002051897 |
| 334956 | GO:0000166 | nucleotide binding | MF | La ribonucleoprotein domain family, member 7 | larp7 | 2.110121458 | 0.002051897 |
| 334956 | GO:0003723 | RNA binding | MF | La ribonucleoprotein domain family, member 7 | larp7 | 2.110121458 | 0.002051897 |
| 566692 | GO:0005529 | carbohydrate binding | MF | si:ch211-154a22.8 | si:ch211-154a22.8 | 2.100106532 | 0.002336494 |
| 553415 | GO:0007264 | small GTPase mediated signal transduction | BP | Rho-related BTB domain containing 2a | rhobtb2a | 2.080737093 | 0.002308826 |
| 553415 | GO:0005622 | intracellular | CC | Rho-related BTB domain containing 2a | rhobtb2a | 2.080737093 | 0.002308826 |
| 553415 | GO:0016020 | membrane | CC | Rho-related BTB domain containing 2a | rhobtb2a | 2.080737093 | 0.002308826 |
| 553415 | GO:0005525 | GTP binding | MF | Rho-related BTB domain containing 2a | rhobtb2a | 2.080737093 | 0.002308826 |
| 553415 | GO:0000166 | nucleotide binding | MF | Rho-related BTB domain containing 2a | rhobtb2a | 2.080737093 | 0.002308826 |
| 100003563 | GO:0031110 | regulation of microtubule polymerization or depolymerization | BP | stathmin-like 2b | stmn2b | 2.076875411 | 0.002259912 |
| 100003563 | GO:0003674 | molecular_function | MF | stathmin-like 2b | stmn2b | 2.076875411 | 0.002259912 |
| 559502 | GO:0005529 | carbohydrate binding | MF | mannose receptor, C type 1b | mrc1b | 2.05416039 | 0.002516006 |
| 559245 | GO:0006355 | regulation of transcription, DNA-dependent | BP | RAR-related orphan receptor C a | rorca | 2.053840309 | 0.002732834 |
| 559245 | GO:0043401 | steroid hormone mediated signaling pathway | BP | RAR-related orphan receptor C a | rorca | 2.053840309 | 0.002732834 |
| 559245 | GO:0006351 | transcription, DNA-dependent | BP | RAR-related orphan receptor C a | rorca | 2.053840309 | 0.002732834 |
| 559245 | GO:0005634 | nucleus | CC | RAR-related orphan receptor C a | rorca | 2.053840309 | 0.002732834 |
| 559245 | GO:0003677 | DNA binding | MF | RAR-related orphan receptor C a | rorca | 2.053840309 | 0.002732834 |
| 559245 | GO:0004879 | ligand-activated sequence-specific DNA binding RNA polymerase II transcription factor activity | MF | RAR-related orphan receptor C a | rorca | 2.053840309 | 0.002732834 |
| 559245 | GO:0046872 | metal ion binding | MF | RAR-related orphan receptor C a | rorca | 2.053840309 | 0.002732834 |
| 559245 | GO:0043565 | sequence-specific DNA binding | MF | RAR-related orphan receptor C a | rorca | 2.053840309 | 0.002732834 |
| 559245 | GO:0003700 | sequence-specific DNA binding transcription factor activity | MF | RAR-related orphan receptor C a | rorca | 2.053840309 | 0.002732834 |
| 559245 | GO:0003707 | steroid hormone receptor activity | MF | RAR-related orphan receptor C a | rorca | 2.053840309 | 0.002732834 |
| 559245 | GO:0008270 | zinc ion binding | MF | RAR-related orphan receptor C a | rorca | 2.053840309 | 0.002732834 |
| 558387 | GO:0005622 | intracellular | CC | si:dkey-226l10.6 | si:dkey-226l10.6 | 2.038901317 | 0.002697673 |
| 558387 | GO:0003676 | nucleic acid binding | MF | si:dkey-226l10.6 | si:dkey-226l10.6 | 2.038901317 | 0.002697673 |
| 558387 | GO:0008270 | zinc ion binding | MF | si:dkey-226l10.6 | si:dkey-226l10.6 | 2.038901317 | 0.002697673 |
| 352915 | GO:0045454 | cell redox homeostasis | BP | selenoprotein W, 1 | sepw1 | 2.030175074 | 0.002944115 |
| 352915 | GO:0005737 | cytoplasm | CC | selenoprotein W, 1 | sepw1 | 2.030175074 | 0.002944115 |
| 352915 | GO:0016209 | antioxidant activity | MF | selenoprotein W, 1 | sepw1 | 2.030175074 | 0.002944115 |
| 352915 | GO:0008430 | selenium binding | MF | selenoprotein W, 1 | sepw1 | 2.030175074 | 0.002944115 |
| 751093 | GO:0008283 | cell proliferation | BP | macrophage migration inhibitory factor | mif | 1.978866461 | 0.004170843 |
| 751093 | GO:0043066 | negative regulation of apoptotic process | BP | macrophage migration inhibitory factor | mif | 1.978866461 | 0.004170843 |
| 555510 | GO:0055114 | oxidation-reduction process | BP | cytochrome P450, family 2, subfamily AA, polypeptide 12 | cyp2aa12 | 1.949345129 | 0.003985796 |
| 555510 | GO:0005575 | cellular_component | CC | cytochrome P450, family 2, subfamily AA, polypeptide 12 | cyp2aa12 | 1.949345129 | 0.003985796 |
| 555510 | GO:0009055 | electron carrier activity | MF | cytochrome P450, family 2, subfamily AA, polypeptide 12 | cyp2aa12 | 1.949345129 | 0.003985796 |
| 555510 | GO:0020037 | heme binding | MF | cytochrome P450, family 2, subfamily AA, polypeptide 12 | cyp2aa12 | 1.949345129 | 0.003985796 |
| 555510 | GO:0005506 | iron ion binding | MF | cytochrome P450, family 2, subfamily AA, polypeptide 12 | cyp2aa12 | 1.949345129 | 0.003985796 |
| 555510 | GO:0046872 | metal ion binding | MF | cytochrome P450, family 2, subfamily AA, polypeptide 12 | cyp2aa12 | 1.949345129 | 0.003985796 |
| 555510 | GO:0004497 | monooxygenase activity | MF | cytochrome P450, family 2, subfamily AA, polypeptide 12 | cyp2aa12 | 1.949345129 | 0.003985796 |
| 555510 | GO:0016491 | oxidoreductase activity | MF | cytochrome P450, family 2, subfamily AA, polypeptide 12 | cyp2aa12 | 1.949345129 | 0.003985796 |
| 555510 | GO:0016705 | oxidoreductase activity, acting on paired donors, with incorporation or reduction of molecular oxygen | MF | cytochrome P450, family 2, subfamily AA, polypeptide 12 | cyp2aa12 | 1.949345129 | 0.003985796 |
| 555510 | GO:0016712 | oxidoreductase activity, acting on paired donors, with incorporation or reduction of molecular oxygen, reduced flavin or flavoprotein as one donor, and incorporation of one atom of oxygen | MF | cytochrome P450, family 2, subfamily AA, polypeptide 12 | cyp2aa12 | 1.949345129 | 0.003985796 |
| 445178 | GO:0006412 | translation | BP | alanyl-tRNA synthetase domain containing 1 | aarsd1 | 1.910317637 | 0.00515392 |
| 445178 | GO:0043039 | tRNA aminoacylation | BP | alanyl-tRNA synthetase domain containing 1 | aarsd1 | 1.910317637 | 0.00515392 |
| 445178 | GO:0005737 | cytoplasm | CC | alanyl-tRNA synthetase domain containing 1 | aarsd1 | 1.910317637 | 0.00515392 |
| 445178 | GO:0005524 | ATP binding | MF | alanyl-tRNA synthetase domain containing 1 | aarsd1 | 1.910317637 | 0.00515392 |
| 445178 | GO:0016876 | ligase activity, forming aminoacyl-tRNA and related compounds | MF | alanyl-tRNA synthetase domain containing 1 | aarsd1 | 1.910317637 | 0.00515392 |
| 445178 | GO:0046872 | metal ion binding | MF | alanyl-tRNA synthetase domain containing 1 | aarsd1 | 1.910317637 | 0.00515392 |
| 445178 | GO:0000166 | nucleotide binding | MF | alanyl-tRNA synthetase domain containing 1 | aarsd1 | 1.910317637 | 0.00515392 |
| 562261 | GO:0016021 | integral to membrane | CC | androgen-induced 1 (H. sapiens) | aig1 | 1.89650797 | 0.005109694 |
| 555979 | GO:0055085 | transmembrane transport | BP | zgc:171831 | zgc:171831 | 1.88895238 | 0.00517715 |
| 555979 | GO:0016021 | integral to membrane | CC | zgc:171831 | zgc:171831 | 1.88895238 | 0.00517715 |
| 555979 | GO:0022857 | transmembrane transporter activity | MF | zgc:171831 | zgc:171831 | 1.88895238 | 0.00517715 |
| 567331 | GO:0007165 | signal transduction | BP | SPARC-like 1 | sparcl1 | 1.855855963 | 0.00597583 |
| 567331 | GO:0005615 | extracellular space | CC | SPARC-like 1 | sparcl1 | 1.855855963 | 0.00597583 |
| 567331 | GO:0005578 | proteinaceous extracellular matrix | CC | SPARC-like 1 | sparcl1 | 1.855855963 | 0.00597583 |
| 567331 | GO:0005509 | calcium ion binding | MF | SPARC-like 1 | sparcl1 | 1.855855963 | 0.00597583 |
| 114446 | GO:0007015 | actin filament organization | BP | discs, large (Drosophila) homolog 1 | dlg1 | 1.853146089 | 0.006241411 |
| 114446 | GO:0016337 | cell-cell adhesion | BP | discs, large (Drosophila) homolog 1 | dlg1 | 1.853146089 | 0.006241411 |
| 114446 | GO:0030866 | cortical actin cytoskeleton organization | BP | discs, large (Drosophila) homolog 1 | dlg1 | 1.853146089 | 0.006241411 |
| 114446 | GO:0001935 | endothelial cell proliferation | BP | discs, large (Drosophila) homolog 1 | dlg1 | 1.853146089 | 0.006241411 |
| 114446 | GO:0045930 | negative regulation of mitotic cell cycle | BP | discs, large (Drosophila) homolog 1 | dlg1 | 1.853146089 | 0.006241411 |
| 114446 | GO:0016323 | basolateral plasma membrane | CC | discs, large (Drosophila) homolog 1 | dlg1 | 1.853146089 | 0.006241411 |
| 114446 | GO:0030054 | cell junction | CC | discs, large (Drosophila) homolog 1 | dlg1 | 1.853146089 | 0.006241411 |
| 114446 | GO:0005783 | endoplasmic reticulum | CC | discs, large (Drosophila) homolog 1 | dlg1 | 1.853146089 | 0.006241411 |
| 114446 | GO:0005789 | endoplasmic reticulum membrane | CC | discs, large (Drosophila) homolog 1 | dlg1 | 1.853146089 | 0.006241411 |
| 114446 | GO:0016020 | membrane | CC | discs, large (Drosophila) homolog 1 | dlg1 | 1.853146089 | 0.006241411 |
| 114446 | GO:0019902 | phosphatase binding | MF | discs, large (Drosophila) homolog 1 | dlg1 | 1.853146089 | 0.006241411 |
| 114446 | GO:0019901 | protein kinase binding | MF | discs, large (Drosophila) homolog 1 | dlg1 | 1.853146089 | 0.006241411 |
| 571468 | GO:0005575 | cellular_component | CC | collagen, type XIX, alpha 1 | col19a1 | 1.852434225 | 0.006132592 |
| 192302 | GO:0016568 | chromatin modification | BP | histone deacetylase 1 | hdac1 | 1.832667935 | 0.006596089 |
| 192302 | GO:0016575 | histone deacetylation | BP | histone deacetylase 1 | hdac1 | 1.832667935 | 0.006596089 |
| 192302 | GO:0070932 | histone H3 deacetylation | BP | histone deacetylase 1 | hdac1 | 1.832667935 | 0.006596089 |
| 192302 | GO:0070933 | histone H4 deacetylation | BP | histone deacetylase 1 | hdac1 | 1.832667935 | 0.006596089 |
| 192302 | GO:0008285 | negative regulation of cell proliferation | BP | histone deacetylase 1 | hdac1 | 1.832667935 | 0.006596089 |
| 192302 | GO:0006355 | regulation of transcription, DNA-dependent | BP | histone deacetylase 1 | hdac1 | 1.832667935 | 0.006596089 |
| 192302 | GO:0006351 | transcription, DNA-dependent | BP | histone deacetylase 1 | hdac1 | 1.832667935 | 0.006596089 |
| 192302 | GO:0016055 | Wnt receptor signaling pathway | BP | histone deacetylase 1 | hdac1 | 1.832667935 | 0.006596089 |
| 192302 | GO:0005634 | nucleus | CC | histone deacetylase 1 | hdac1 | 1.832667935 | 0.006596089 |
| 192302 | GO:0004407 | histone deacetylase activity | MF | histone deacetylase 1 | hdac1 | 1.832667935 | 0.006596089 |
| 192302 | GO:0031078 | histone deacetylase activity (H3-K14 specific) | MF | histone deacetylase 1 | hdac1 | 1.832667935 | 0.006596089 |
| 192302 | GO:0032129 | histone deacetylase activity (H3-K9 specific) | MF | histone deacetylase 1 | hdac1 | 1.832667935 | 0.006596089 |
| 192302 | GO:0034739 | histone deacetylase activity (H4-K16 specific) | MF | histone deacetylase 1 | hdac1 | 1.832667935 | 0.006596089 |
| 192302 | GO:0016787 | hydrolase activity | MF | histone deacetylase 1 | hdac1 | 1.832667935 | 0.006596089 |
| 192302 | GO:0032041 | NAD-dependent histone deacetylase activity (H3-K14 specific) | MF | histone deacetylase 1 | hdac1 | 1.832667935 | 0.006596089 |
| 192302 | GO:0046969 | NAD-dependent histone deacetylase activity (H3-K9 specific) | MF | histone deacetylase 1 | hdac1 | 1.832667935 | 0.006596089 |
| 192302 | GO:0046970 | NAD-dependent histone deacetylase activity (H4-K16 specific) | MF | histone deacetylase 1 | hdac1 | 1.832667935 | 0.006596089 |
| 100170801 | GO:0038032 | termination of G-protein coupled receptor signaling pathway | BP | regulator of G-protein signaling 19 | rgs19 | 1.83222517 | 0.007793365 |
| 100170801 | GO:0005575 | cellular_component | CC | regulator of G-protein signaling 19 | rgs19 | 1.83222517 | 0.007793365 |
| 558307 | GO:0006839 | mitochondrial transport | BP | solute carrier family 25, member 36b | slc25a36b | 1.82402968 | 0.007275171 |
| 558307 | GO:0006810 | transport | BP | solute carrier family 25, member 36b | slc25a36b | 1.82402968 | 0.007275171 |
| 558307 | GO:0016021 | integral to membrane | CC | solute carrier family 25, member 36b | slc25a36b | 1.82402968 | 0.007275171 |
| 558307 | GO:0016020 | membrane | CC | solute carrier family 25, member 36b | slc25a36b | 1.82402968 | 0.007275171 |
| 558307 | GO:0031966 | mitochondrial membrane | CC | solute carrier family 25, member 36b | slc25a36b | 1.82402968 | 0.007275171 |
| 572258 | GO:0006351 | transcription, DNA-dependent | BP | im:7139382 | im:7139382 | 1.820492895 | 0.006950315 |
| 572258 | GO:0005634 | nucleus | CC | im:7139382 | im:7139382 | 1.820492895 | 0.006950315 |
| 572258 | GO:0003677 | DNA binding | MF | im:7139382 | im:7139382 | 1.820492895 | 0.006950315 |
| 572258 | GO:0003899 | DNA-directed RNA polymerase activity | MF | im:7139382 | im:7139382 | 1.820492895 | 0.006950315 |
| 572258 | GO:0016779 | nucleotidyltransferase activity | MF | im:7139382 | im:7139382 | 1.820492895 | 0.006950315 |
| 572258 | GO:0032549 | ribonucleoside binding | MF | im:7139382 | im:7139382 | 1.820492895 | 0.006950315 |
| 572258 | GO:0016740 | transferase activity | MF | im:7139382 | im:7139382 | 1.820492895 | 0.006950315 |
| 572258 | GO:0008270 | zinc ion binding | MF | im:7139382 | im:7139382 | 1.820492895 | 0.006950315 |
| 170452 | GO:0006811 | ion transport | BP | glutamate receptor, ionotropic, AMPA 3a | gria3a | 1.813421267 | 0.007103253 |
| 170452 | GO:0006810 | transport | BP | glutamate receptor, ionotropic, AMPA 3a | gria3a | 1.813421267 | 0.007103253 |
| 170452 | GO:0030054 | cell junction | CC | glutamate receptor, ionotropic, AMPA 3a | gria3a | 1.813421267 | 0.007103253 |
| 170452 | GO:0016021 | integral to membrane | CC | glutamate receptor, ionotropic, AMPA 3a | gria3a | 1.813421267 | 0.007103253 |
| 170452 | GO:0016020 | membrane | CC | glutamate receptor, ionotropic, AMPA 3a | gria3a | 1.813421267 | 0.007103253 |
| 170452 | GO:0030288 | outer membrane-bounded periplasmic space | CC | glutamate receptor, ionotropic, AMPA 3a | gria3a | 1.813421267 | 0.007103253 |
| 170452 | GO:0005886 | plasma membrane | CC | glutamate receptor, ionotropic, AMPA 3a | gria3a | 1.813421267 | 0.007103253 |
| 170452 | GO:0005234 | extracellular-glutamate-gated ion channel activity | MF | glutamate receptor, ionotropic, AMPA 3a | gria3a | 1.813421267 | 0.007103253 |
| 170452 | GO:0005216 | ion channel activity | MF | glutamate receptor, ionotropic, AMPA 3a | gria3a | 1.813421267 | 0.007103253 |
| 170452 | GO:0004970 | ionotropic glutamate receptor activity | MF | glutamate receptor, ionotropic, AMPA 3a | gria3a | 1.813421267 | 0.007103253 |
| 170452 | GO:0004872 | receptor activity | MF | glutamate receptor, ionotropic, AMPA 3a | gria3a | 1.813421267 | 0.007103253 |
| 170452 | GO:0005215 | transporter activity | MF | glutamate receptor, ionotropic, AMPA 3a | gria3a | 1.813421267 | 0.007103253 |
| 560402 | GO:0008150 | biological_process | BP | zgc:165500 | zgc:165500 | 1.811301067 | 0.007449089 |
| 560402 | GO:0005575 | cellular_component | CC | zgc:165500 | zgc:165500 | 1.811301067 | 0.007449089 |
| 560402 | GO:0003674 | molecular_function | MF | zgc:165500 | zgc:165500 | 1.811301067 | 0.007449089 |
| 100003895 | GO:0019904 | protein domain specific binding | MF | ADP-ribosylation factor interacting protein 1 (arfaptin 1) | arfip1 | 1.797643577 | 0.007578258 |
| 768129 | GO:0008150 | biological_process | BP | zgc:153119 | zgc:153119 | 1.790345663 | 0.008449434 |
| 768129 | GO:0005575 | cellular_component | CC | zgc:153119 | zgc:153119 | 1.790345663 | 0.008449434 |
| 768129 | GO:0003674 | molecular_function | MF | zgc:153119 | zgc:153119 | 1.790345663 | 0.008449434 |
| 100033576 | GO:0035195 | gene silencing by miRNA | BP | microRNA 19a | mir19a | 1.78978952 | 0.008209121 |
| 100033576 | GO:0005575 | cellular_component | CC | microRNA 19a | mir19a | 1.78978952 | 0.008209121 |
| 100033576 | GO:0003674 | molecular_function | MF | microRNA 19a | mir19a | 1.78978952 | 0.008209121 |
| 378440 | GO:0008152 | metabolic process | BP | retinol dehydrogenase 1 | rdh1 | 1.789140911 | 0.007976253 |
| 378440 | GO:0055114 | oxidation-reduction process | BP | retinol dehydrogenase 1 | rdh1 | 1.789140911 | 0.007976253 |
| 378440 | GO:0042574 | retinal metabolic process | BP | retinol dehydrogenase 1 | rdh1 | 1.789140911 | 0.007976253 |
| 378440 | GO:0042572 | retinol metabolic process | BP | retinol dehydrogenase 1 | rdh1 | 1.789140911 | 0.007976253 |
| 378440 | GO:0005575 | cellular_component | CC | retinol dehydrogenase 1 | rdh1 | 1.789140911 | 0.007976253 |
| 378440 | GO:0000166 | nucleotide binding | MF | retinol dehydrogenase 1 | rdh1 | 1.789140911 | 0.007976253 |
| 378440 | GO:0016491 | oxidoreductase activity | MF | retinol dehydrogenase 1 | rdh1 | 1.789140911 | 0.007976253 |
| 378440 | GO:0004745 | retinol dehydrogenase activity | MF | retinol dehydrogenase 1 | rdh1 | 1.789140911 | 0.007976253 |
| 768139 | GO:0044237 | cellular metabolic process | BP | zgc:153776 | zgc:153776 | 1.787685132 | 0.00844095 |
| 768139 | GO:0005575 | cellular_component | CC | zgc:153776 | zgc:153776 | 1.787685132 | 0.00844095 |
| 768139 | GO:0003824 | catalytic activity | MF | zgc:153776 | zgc:153776 | 1.787685132 | 0.00844095 |
| 768139 | GO:0050662 | coenzyme binding | MF | zgc:153776 | zgc:153776 | 1.787685132 | 0.00844095 |
| 768139 | GO:0000166 | nucleotide binding | MF | zgc:153776 | zgc:153776 | 1.787685132 | 0.00844095 |
| 447889 | GO:0006139 | nucleobase-containing compound metabolic process | BP | purine nucleoside phosphorylase 5b | pnp5b | 1.782648276 | 0.008492616 |
| 447889 | GO:0009116 | nucleoside metabolic process | BP | purine nucleoside phosphorylase 5b | pnp5b | 1.782648276 | 0.008492616 |
| 447889 | GO:0005575 | cellular_component | CC | purine nucleoside phosphorylase 5b | pnp5b | 1.782648276 | 0.008492616 |
| 447889 | GO:0003824 | catalytic activity | MF | purine nucleoside phosphorylase 5b | pnp5b | 1.782648276 | 0.008492616 |
| 447889 | GO:0004731 | purine-nucleoside phosphorylase activity | MF | purine nucleoside phosphorylase 5b | pnp5b | 1.782648276 | 0.008492616 |
| 447889 | GO:0016740 | transferase activity | MF | purine nucleoside phosphorylase 5b | pnp5b | 1.782648276 | 0.008492616 |
| 447889 | GO:0016757 | transferase activity, transferring glycosyl groups | MF | purine nucleoside phosphorylase 5b | pnp5b | 1.782648276 | 0.008492616 |
| 447889 | GO:0016763 | transferase activity, transferring pentosyl groups | MF | purine nucleoside phosphorylase 5b | pnp5b | 1.782648276 | 0.008492616 |
| 450038 | GO:0008150 | biological_process | BP | family with sequence similarity 86, member A | fam86a | 1.782212211 | 0.008113028 |
| 450038 | GO:0005575 | cellular_component | CC | family with sequence similarity 86, member A | fam86a | 1.782212211 | 0.008113028 |
| 450038 | GO:0003674 | molecular_function | MF | family with sequence similarity 86, member A | fam86a | 1.782212211 | 0.008113028 |
| 436920 | GO:0005622 | intracellular | CC | zgc:92020 | zgc:92020 | 1.775123567 | 0.008386156 |
| 436920 | GO:0003676 | nucleic acid binding | MF | zgc:92020 | zgc:92020 | 1.775123567 | 0.008386156 |
| 436920 | GO:0008270 | zinc ion binding | MF | zgc:92020 | zgc:92020 | 1.775123567 | 0.008386156 |
| 494039 | GO:0005575 | cellular_component | CC | zgc:103710 | zgc:103710 | 1.757249974 | 0.008978098 |
| 494039 | GO:0004866 | endopeptidase inhibitor activity | MF | zgc:103710 | zgc:103710 | 1.757249974 | 0.008978098 |
| 791173 | GO:0006629 | lipid metabolic process | BP | patatin-like phospholipase domain containing 7a | pnpla7a | 1.749830217 | 0.009404348 |
| 791173 | GO:0008152 | metabolic process | BP | patatin-like phospholipase domain containing 7a | pnpla7a | 1.749830217 | 0.009404348 |
| 791173 | GO:0005575 | cellular_component | CC | patatin-like phospholipase domain containing 7a | pnpla7a | 1.749830217 | 0.009404348 |
| 791173 | GO:0016787 | hydrolase activity | MF | patatin-like phospholipase domain containing 7a | pnpla7a | 1.749830217 | 0.009404348 |
| 798572 | GO:0016021 | integral to membrane | CC | membrane-spanning 4-domains, subfamily A, member 17A.14 | ms4a17a.14 | 1.746881885 | 0.009678867 |
| 449802 | GO:0006955 | immune response | BP | zgc:103599 | zgc:103599 | 1.726576622 | 0.011390579 |
| 449802 | GO:0016021 | integral to membrane | CC | zgc:103599 | zgc:103599 | 1.726576622 | 0.011390579 |
| 449802 | GO:0016020 | membrane | CC | zgc:103599 | zgc:103599 | 1.726576622 | 0.011390579 |
| 436872 | GO:0005575 | cellular_component | CC | sulfotransferase family 1, cytosolic sulfotransferase 6 | sult1st6 | 1.725955673 | 0.010438255 |
| 436872 | GO:0004304 | estrone sulfotransferase activity | MF | sulfotransferase family 1, cytosolic sulfotransferase 6 | sult1st6 | 1.725955673 | 0.010438255 |
| 436872 | GO:0008146 | sulfotransferase activity | MF | sulfotransferase family 1, cytosolic sulfotransferase 6 | sult1st6 | 1.725955673 | 0.010438255 |
| 436872 | GO:0016740 | transferase activity | MF | sulfotransferase family 1, cytosolic sulfotransferase 6 | sult1st6 | 1.725955673 | 0.010438255 |
| 558037 | GO:0007049 | cell cycle | BP | zgc:162239 | zgc:162239 | 1.719966959 | 0.010499835 |
| 558037 | GO:0031105 | septin complex | CC | zgc:162239 | zgc:162239 | 1.719966959 | 0.010499835 |
| 558037 | GO:0005525 | GTP binding | MF | zgc:162239 | zgc:162239 | 1.719966959 | 0.010499835 |
| 558037 | GO:0000166 | nucleotide binding | MF | zgc:162239 | zgc:162239 | 1.719966959 | 0.010499835 |
| 352929 | GO:0055114 | oxidation-reduction process | BP | glutathione peroxidase 4b | gpx4b | 1.713929891 | 0.011544954 |
| 352929 | GO:0006979 | response to oxidative stress | BP | glutathione peroxidase 4b | gpx4b | 1.713929891 | 0.011544954 |
| 352929 | GO:0004602 | glutathione peroxidase activity | MF | glutathione peroxidase 4b | gpx4b | 1.713929891 | 0.011544954 |
| 352929 | GO:0016491 | oxidoreductase activity | MF | glutathione peroxidase 4b | gpx4b | 1.713929891 | 0.011544954 |
| 352929 | GO:0004601 | peroxidase activity | MF | glutathione peroxidase 4b | gpx4b | 1.713929891 | 0.011544954 |
| 791641 | GO:0046836 | glycolipid transport | BP | glycolipid transfer protein domain containing 1 | gltpd1 | 1.709903198 | 0.01119443 |
| 791641 | GO:0005737 | cytoplasm | CC | glycolipid transfer protein domain containing 1 | gltpd1 | 1.709903198 | 0.01119443 |
| 791641 | GO:0051861 | glycolipid binding | MF | glycolipid transfer protein domain containing 1 | gltpd1 | 1.709903198 | 0.01119443 |
| 791641 | GO:0017089 | glycolipid transporter activity | MF | glycolipid transfer protein domain containing 1 | gltpd1 | 1.709903198 | 0.01119443 |
| 445290 | GO:0008152 | metabolic process | BP | acetyl-Coenzyme A acetyltransferase 1 (acetoacetyl Coenzyme A thiolase) | acat1 | 1.694040677 | 0.011844553 |
| 445290 | GO:0005739 | mitochondrion | CC | acetyl-Coenzyme A acetyltransferase 1 (acetoacetyl Coenzyme A thiolase) | acat1 | 1.694040677 | 0.011844553 |
| 445290 | GO:0003985 | acetyl-CoA C-acetyltransferase activity | MF | acetyl-Coenzyme A acetyltransferase 1 (acetoacetyl Coenzyme A thiolase) | acat1 | 1.694040677 | 0.011844553 |
| 445290 | GO:0003824 | catalytic activity | MF | acetyl-Coenzyme A acetyltransferase 1 (acetoacetyl Coenzyme A thiolase) | acat1 | 1.694040677 | 0.011844553 |
| 445290 | GO:0046872 | metal ion binding | MF | acetyl-Coenzyme A acetyltransferase 1 (acetoacetyl Coenzyme A thiolase) | acat1 | 1.694040677 | 0.011844553 |
| 445290 | GO:0016740 | transferase activity | MF | acetyl-Coenzyme A acetyltransferase 1 (acetoacetyl Coenzyme A thiolase) | acat1 | 1.694040677 | 0.011844553 |
| 445290 | GO:0016746 | transferase activity, transferring acyl groups | MF | acetyl-Coenzyme A acetyltransferase 1 (acetoacetyl Coenzyme A thiolase) | acat1 | 1.694040677 | 0.011844553 |
| 445290 | GO:0016747 | transferase activity, transferring acyl groups other than amino-acyl groups | MF | acetyl-Coenzyme A acetyltransferase 1 (acetoacetyl Coenzyme A thiolase) | acat1 | 1.694040677 | 0.011844553 |
| 445122 | GO:0008150 | biological_process | BP | zgc:100908 | zgc:100908 | 1.692591915 | 0.012188733 |
| 445122 | GO:0005743 | mitochondrial inner membrane | CC | zgc:100908 | zgc:100908 | 1.692591915 | 0.012188733 |
| 445122 | GO:0005747 | mitochondrial respiratory chain complex I | CC | zgc:100908 | zgc:100908 | 1.692591915 | 0.012188733 |
| 445122 | GO:0003674 | molecular_function | MF | zgc:100908 | zgc:100908 | 1.692591915 | 0.012188733 |
| 793882 | GO:0016310 | phosphorylation | BP | G protein-coupled receptor kinase interactor 2a | git2a | 1.69197016 | 0.011799951 |
| 793882 | GO:0032312 | regulation of ARF GTPase activity | BP | G protein-coupled receptor kinase interactor 2a | git2a | 1.69197016 | 0.011799951 |
| 793882 | GO:0022604 | regulation of cell morphogenesis | BP | G protein-coupled receptor kinase interactor 2a | git2a | 1.69197016 | 0.011799951 |
| 793882 | GO:0008060 | ARF GTPase activator activity | MF | G protein-coupled receptor kinase interactor 2a | git2a | 1.69197016 | 0.011799951 |
| 793882 | GO:0016301 | kinase activity | MF | G protein-coupled receptor kinase interactor 2a | git2a | 1.69197016 | 0.011799951 |
| 793882 | GO:0008270 | zinc ion binding | MF | G protein-coupled receptor kinase interactor 2a | git2a | 1.69197016 | 0.011799951 |
| 405853 | GO:0006810 | transport | BP | solute carrier organic anion transporter family, member 1F1 | slco1f1 | 1.690249868 | 0.01197543 |
| 405853 | GO:0016020 | membrane | CC | solute carrier organic anion transporter family, member 1F1 | slco1f1 | 1.690249868 | 0.01197543 |
| 405853 | GO:0005215 | transporter activity | MF | solute carrier organic anion transporter family, member 1F1 | slco1f1 | 1.690249868 | 0.01197543 |
| 792062 | GO:0005882 | intermediate filament | CC | type I cytokeratin, enveloping layer, like | cyt1l | 1.688623489 | 0.019834365 |
| 792062 | GO:0005198 | structural molecule activity | MF | type I cytokeratin, enveloping layer, like | cyt1l | 1.688623489 | 0.019834365 |
| 100126134 | GO:0008150 | biological_process | BP | crystallin, gamma M2d10 | crygm2d10 | 1.678800511 | 0.015764109 |
| 100126134 | GO:0005575 | cellular_component | CC | crystallin, gamma M2d10 | crygm2d10 | 1.678800511 | 0.015764109 |
| 100126134 | GO:0003674 | molecular_function | MF | crystallin, gamma M2d10 | crygm2d10 | 1.678800511 | 0.015764109 |
| 335709 | GO:0015986 | ATP synthesis coupled proton transport | BP | ATP synthase, H+ transporting, mitochondrial F1 complex, delta subunit | atp5d | 1.672720373 | 0.012733367 |
| 335709 | GO:0045261 | proton-transporting ATP synthase complex, catalytic core F(1) | CC | ATP synthase, H+ transporting, mitochondrial F1 complex, delta subunit | atp5d | 1.672720373 | 0.012733367 |
| 335709 | GO:0046933 | hydrogen ion transporting ATP synthase activity, rotational mechanism | MF | ATP synthase, H+ transporting, mitochondrial F1 complex, delta subunit | atp5d | 1.672720373 | 0.012733367 |
| 335709 | GO:0046961 | proton-transporting ATPase activity, rotational mechanism | MF | ATP synthase, H+ transporting, mitochondrial F1 complex, delta subunit | atp5d | 1.672720373 | 0.012733367 |
| 100141491 | GO:0042802 | identical protein binding | MF | sc:d0220 | sc:d0220 | 1.67212624 | 0.012894441 |
| 140615 | GO:0022900 | electron transport chain | BP | fatty acid desaturase 2 | fads2 | 1.666166711 | 0.01295452 |
| 140615 | GO:0006633 | fatty acid biosynthetic process | BP | fatty acid desaturase 2 | fads2 | 1.666166711 | 0.01295452 |
| 140615 | GO:0006631 | fatty acid metabolic process | BP | fatty acid desaturase 2 | fads2 | 1.666166711 | 0.01295452 |
| 140615 | GO:0006629 | lipid metabolic process | BP | fatty acid desaturase 2 | fads2 | 1.666166711 | 0.01295452 |
| 140615 | GO:0055114 | oxidation-reduction process | BP | fatty acid desaturase 2 | fads2 | 1.666166711 | 0.01295452 |
| 140615 | GO:0006810 | transport | BP | fatty acid desaturase 2 | fads2 | 1.666166711 | 0.01295452 |
| 140615 | GO:0005783 | endoplasmic reticulum | CC | fatty acid desaturase 2 | fads2 | 1.666166711 | 0.01295452 |
| 140615 | GO:0005789 | endoplasmic reticulum membrane | CC | fatty acid desaturase 2 | fads2 | 1.666166711 | 0.01295452 |
| 140615 | GO:0016021 | integral to membrane | CC | fatty acid desaturase 2 | fads2 | 1.666166711 | 0.01295452 |
| 140615 | GO:0016020 | membrane | CC | fatty acid desaturase 2 | fads2 | 1.666166711 | 0.01295452 |
| 140615 | GO:0020037 | heme binding | MF | fatty acid desaturase 2 | fads2 | 1.666166711 | 0.01295452 |
| 140615 | GO:0005506 | iron ion binding | MF | fatty acid desaturase 2 | fads2 | 1.666166711 | 0.01295452 |
| 140615 | GO:0016491 | oxidoreductase activity | MF | fatty acid desaturase 2 | fads2 | 1.666166711 | 0.01295452 |
| 140615 | GO:0016717 | oxidoreductase activity, acting on paired donors, with oxidation of a pair of donors resulting in the reduction of molecular oxygen to two molecules of water | MF | fatty acid desaturase 2 | fads2 | 1.666166711 | 0.01295452 |
| 571904 | GO:0031225 | anchored to membrane | CC | zgc:171629 | zgc:171629 | 1.66293496 | 0.013293505 |
| 571904 | GO:0016020 | membrane | CC | zgc:171629 | zgc:171629 | 1.66293496 | 0.013293505 |
| 571904 | GO:0005886 | plasma membrane | CC | zgc:171629 | zgc:171629 | 1.66293496 | 0.013293505 |
| 571904 | GO:0005578 | proteinaceous extracellular matrix | CC | zgc:171629 | zgc:171629 | 1.66293496 | 0.013293505 |
| 571904 | GO:0043395 | heparan sulfate proteoglycan binding | MF | zgc:171629 | zgc:171629 | 1.66293496 | 0.013293505 |
| 368316 | GO:0007049 | cell cycle | BP | cyclin B2 | ccnb2 | 1.661004659 | 0.014571968 |
| 368316 | GO:0051301 | cell division | BP | cyclin B2 | ccnb2 | 1.661004659 | 0.014571968 |
| 368316 | GO:0051726 | regulation of cell cycle | BP | cyclin B2 | ccnb2 | 1.661004659 | 0.014571968 |
| 368316 | GO:0000079 | regulation of cyclin-dependent protein kinase activity | BP | cyclin B2 | ccnb2 | 1.661004659 | 0.014571968 |
| 368316 | GO:0005634 | nucleus | CC | cyclin B2 | ccnb2 | 1.661004659 | 0.014571968 |
| 368316 | GO:0019901 | protein kinase binding | MF | cyclin B2 | ccnb2 | 1.661004659 | 0.014571968 |
| 100033775 | GO:0035195 | gene silencing by miRNA | BP | microRNA 216a-1 | mir216a-1 | 1.658583568 | 0.017060121 |
| 100033775 | GO:0005575 | cellular_component | CC | microRNA 216a-1 | mir216a-1 | 1.658583568 | 0.017060121 |
| 100033775 | GO:0003674 | molecular_function | MF | microRNA 216a-1 | mir216a-1 | 1.658583568 | 0.017060121 |
| 559087 | GO:0008152 | metabolic process | BP | phospholipase A2, group IVA (cytosolic, calcium-dependent) | pla2g4a | 1.647006236 | 0.014105498 |
| 559087 | GO:0009395 | phospholipid catabolic process | BP | phospholipase A2, group IVA (cytosolic, calcium-dependent) | pla2g4a | 1.647006236 | 0.014105498 |
| 559087 | GO:0004620 | phospholipase activity | MF | phospholipase A2, group IVA (cytosolic, calcium-dependent) | pla2g4a | 1.647006236 | 0.014105498 |
| 100036767 | GO:0055114 | oxidation-reduction process | BP | procollagen-lysine, 2-oxoglutarate 5-dioxygenase 2 | plod2 | 1.620767422 | 0.015523165 |
| 100036767 | GO:0005581 | collagen | CC | procollagen-lysine, 2-oxoglutarate 5-dioxygenase 2 | plod2 | 1.620767422 | 0.015523165 |
| 100036767 | GO:0005783 | endoplasmic reticulum | CC | procollagen-lysine, 2-oxoglutarate 5-dioxygenase 2 | plod2 | 1.620767422 | 0.015523165 |
| 100036767 | GO:0005506 | iron ion binding | MF | procollagen-lysine, 2-oxoglutarate 5-dioxygenase 2 | plod2 | 1.620767422 | 0.015523165 |
| 100036767 | GO:0031418 | L-ascorbic acid binding | MF | procollagen-lysine, 2-oxoglutarate 5-dioxygenase 2 | plod2 | 1.620767422 | 0.015523165 |
| 100036767 | GO:0016491 | oxidoreductase activity | MF | procollagen-lysine, 2-oxoglutarate 5-dioxygenase 2 | plod2 | 1.620767422 | 0.015523165 |
| 100036767 | GO:0016705 | oxidoreductase activity, acting on paired donors, with incorporation or reduction of molecular oxygen | MF | procollagen-lysine, 2-oxoglutarate 5-dioxygenase 2 | plod2 | 1.620767422 | 0.015523165 |
| 100036767 | GO:0016706 | oxidoreductase activity, acting on paired donors, with incorporation or reduction of molecular oxygen, 2-oxoglutarate as one donor, and incorporation of one atom each of oxygen into both donors | MF | procollagen-lysine, 2-oxoglutarate 5-dioxygenase 2 | plod2 | 1.620767422 | 0.015523165 |
| 100036767 | GO:0016702 | oxidoreductase activity, acting on single donors with incorporation of molecular oxygen, incorporation of two atoms of oxygen | MF | procollagen-lysine, 2-oxoglutarate 5-dioxygenase 2 | plod2 | 1.620767422 | 0.015523165 |
| 100036767 | GO:0008475 | procollagen-lysine 5-dioxygenase activity | MF | procollagen-lysine, 2-oxoglutarate 5-dioxygenase 2 | plod2 | 1.620767422 | 0.015523165 |
| 796385 | GO:0003950 | NAD+ ADP-ribosyltransferase activity | MF | si:dkey-71l1.7 | si:dkey-71l1.7 | 1.617158384 | 0.015979914 |
| 394111 | GO:0007049 | cell cycle | BP | pelota homolog (Drosophila) | pelo | 1.613841536 | 0.016641202 |
| 394111 | GO:0051301 | cell division | BP | pelota homolog (Drosophila) | pelo | 1.613841536 | 0.016641202 |
| 394111 | GO:0005737 | cytoplasm | CC | pelota homolog (Drosophila) | pelo | 1.613841536 | 0.016641202 |
| 394111 | GO:0005634 | nucleus | CC | pelota homolog (Drosophila) | pelo | 1.613841536 | 0.016641202 |
| 394111 | GO:0004519 | endonuclease activity | MF | pelota homolog (Drosophila) | pelo | 1.613841536 | 0.016641202 |
| 394111 | GO:0016787 | hydrolase activity | MF | pelota homolog (Drosophila) | pelo | 1.613841536 | 0.016641202 |
| 394111 | GO:0046872 | metal ion binding | MF | pelota homolog (Drosophila) | pelo | 1.613841536 | 0.016641202 |
| 394111 | GO:0004518 | nuclease activity | MF | pelota homolog (Drosophila) | pelo | 1.613841536 | 0.016641202 |
| 64615 | GO:0006754 | ATP biosynthetic process | BP | ATPase, Na+/K+ transporting, alpha 1a.4 polypeptide | atp1a1a.4 | 1.606478727 | 0.016750776 |
| 64615 | GO:0006812 | cation transport | BP | ATPase, Na+/K+ transporting, alpha 1a.4 polypeptide | atp1a1a.4 | 1.606478727 | 0.016750776 |
| 64615 | GO:0006811 | ion transport | BP | ATPase, Na+/K+ transporting, alpha 1a.4 polypeptide | atp1a1a.4 | 1.606478727 | 0.016750776 |
| 64615 | GO:0015672 | monovalent inorganic cation transport | BP | ATPase, Na+/K+ transporting, alpha 1a.4 polypeptide | atp1a1a.4 | 1.606478727 | 0.016750776 |
| 64615 | GO:0006810 | transport | BP | ATPase, Na+/K+ transporting, alpha 1a.4 polypeptide | atp1a1a.4 | 1.606478727 | 0.016750776 |
| 64615 | GO:0016021 | integral to membrane | CC | ATPase, Na+/K+ transporting, alpha 1a.4 polypeptide | atp1a1a.4 | 1.606478727 | 0.016750776 |
| 64615 | GO:0016020 | membrane | CC | ATPase, Na+/K+ transporting, alpha 1a.4 polypeptide | atp1a1a.4 | 1.606478727 | 0.016750776 |
| 64615 | GO:0005524 | ATP binding | MF | ATPase, Na+/K+ transporting, alpha 1a.4 polypeptide | atp1a1a.4 | 1.606478727 | 0.016750776 |
| 64615 | GO:0019829 | cation-transporting ATPase activity | MF | ATPase, Na+/K+ transporting, alpha 1a.4 polypeptide | atp1a1a.4 | 1.606478727 | 0.016750776 |
| 64615 | GO:0016787 | hydrolase activity | MF | ATPase, Na+/K+ transporting, alpha 1a.4 polypeptide | atp1a1a.4 | 1.606478727 | 0.016750776 |
| 64615 | GO:0016820 | hydrolase activity, acting on acid anhydrides, catalyzing transmembrane movement of substances | MF | ATPase, Na+/K+ transporting, alpha 1a.4 polypeptide | atp1a1a.4 | 1.606478727 | 0.016750776 |
| 64615 | GO:0046872 | metal ion binding | MF | ATPase, Na+/K+ transporting, alpha 1a.4 polypeptide | atp1a1a.4 | 1.606478727 | 0.016750776 |
| 64615 | GO:0015077 | monovalent inorganic cation transmembrane transporter activity | MF | ATPase, Na+/K+ transporting, alpha 1a.4 polypeptide | atp1a1a.4 | 1.606478727 | 0.016750776 |
| 64615 | GO:0000166 | nucleotide binding | MF | ATPase, Na+/K+ transporting, alpha 1a.4 polypeptide | atp1a1a.4 | 1.606478727 | 0.016750776 |
| 450060 | GO:0055114 | oxidation-reduction process | BP | cytochrome P450, family 2, subfamily AA, polypeptide 8 | cyp2aa8 | 1.601673797 | 0.018121067 |
| 450060 | GO:0009055 | electron carrier activity | MF | cytochrome P450, family 2, subfamily AA, polypeptide 8 | cyp2aa8 | 1.601673797 | 0.018121067 |
| 450060 | GO:0020037 | heme binding | MF | cytochrome P450, family 2, subfamily AA, polypeptide 8 | cyp2aa8 | 1.601673797 | 0.018121067 |
| 450060 | GO:0005506 | iron ion binding | MF | cytochrome P450, family 2, subfamily AA, polypeptide 8 | cyp2aa8 | 1.601673797 | 0.018121067 |
| 450060 | GO:0046872 | metal ion binding | MF | cytochrome P450, family 2, subfamily AA, polypeptide 8 | cyp2aa8 | 1.601673797 | 0.018121067 |
| 450060 | GO:0004497 | monooxygenase activity | MF | cytochrome P450, family 2, subfamily AA, polypeptide 8 | cyp2aa8 | 1.601673797 | 0.018121067 |
| 450060 | GO:0016491 | oxidoreductase activity | MF | cytochrome P450, family 2, subfamily AA, polypeptide 8 | cyp2aa8 | 1.601673797 | 0.018121067 |
| 450060 | GO:0016705 | oxidoreductase activity, acting on paired donors, with incorporation or reduction of molecular oxygen | MF | cytochrome P450, family 2, subfamily AA, polypeptide 8 | cyp2aa8 | 1.601673797 | 0.018121067 |
| 450060 | GO:0016712 | oxidoreductase activity, acting on paired donors, with incorporation or reduction of molecular oxygen, reduced flavin or flavoprotein as one donor, and incorporation of one atom of oxygen | MF | cytochrome P450, family 2, subfamily AA, polypeptide 8 | cyp2aa8 | 1.601673797 | 0.018121067 |
| 450085 | GO:0003674 | molecular_function | MF | golgi phosphoprotein 3 | golph3 | 1.586674971 | 0.018428852 |
| 565235 | GO:0006681 | galactosylceramide metabolic process | BP | aldehyde dehydrogenase 5 family, member A1 (succinate-semialdehyde dehydrogenase) | aldh5a1 | 1.582523493 | 0.017989417 |
| 565235 | GO:0009450 | gamma-aminobutyric acid catabolic process | BP | aldehyde dehydrogenase 5 family, member A1 (succinate-semialdehyde dehydrogenase) | aldh5a1 | 1.582523493 | 0.017989417 |
| 565235 | GO:0008152 | metabolic process | BP | aldehyde dehydrogenase 5 family, member A1 (succinate-semialdehyde dehydrogenase) | aldh5a1 | 1.582523493 | 0.017989417 |
| 565235 | GO:0055114 | oxidation-reduction process | BP | aldehyde dehydrogenase 5 family, member A1 (succinate-semialdehyde dehydrogenase) | aldh5a1 | 1.582523493 | 0.017989417 |
| 565235 | GO:0046459 | short-chain fatty acid metabolic process | BP | aldehyde dehydrogenase 5 family, member A1 (succinate-semialdehyde dehydrogenase) | aldh5a1 | 1.582523493 | 0.017989417 |
| 565235 | GO:0005739 | mitochondrion | CC | aldehyde dehydrogenase 5 family, member A1 (succinate-semialdehyde dehydrogenase) | aldh5a1 | 1.582523493 | 0.017989417 |
| 565235 | GO:0051287 | NAD binding | MF | aldehyde dehydrogenase 5 family, member A1 (succinate-semialdehyde dehydrogenase) | aldh5a1 | 1.582523493 | 0.017989417 |
| 565235 | GO:0016491 | oxidoreductase activity | MF | aldehyde dehydrogenase 5 family, member A1 (succinate-semialdehyde dehydrogenase) | aldh5a1 | 1.582523493 | 0.017989417 |
| 565235 | GO:0016620 | oxidoreductase activity, acting on the aldehyde or oxo group of donors, NAD or NADP as acceptor | MF | aldehyde dehydrogenase 5 family, member A1 (succinate-semialdehyde dehydrogenase) | aldh5a1 | 1.582523493 | 0.017989417 |
| 565235 | GO:0004777 | succinate-semialdehyde dehydrogenase (NAD+) activity | MF | aldehyde dehydrogenase 5 family, member A1 (succinate-semialdehyde dehydrogenase) | aldh5a1 | 1.582523493 | 0.017989417 |
| 565235 | GO:0009013 | succinate-semialdehyde dehydrogenase [NAD(P)+] activity | MF | aldehyde dehydrogenase 5 family, member A1 (succinate-semialdehyde dehydrogenase) | aldh5a1 | 1.582523493 | 0.017989417 |
| 566274 | GO:0007165 | signal transduction | BP | unc-5 homolog Da (C. elegans) | unc5da | 1.572391694 | 0.019222054 |
| 558733 | GO:0006508 | proteolysis | BP | pyroglutamyl-peptidase I-like | pgpep1l | 1.558188617 | 0.019738754 |
| 550496 | GO:0007154 | cell communication | BP | sorting nexin family member 30 | snx30 | 1.557925571 | 0.020393555 |
| 550496 | GO:0015031 | protein transport | BP | sorting nexin family member 30 | snx30 | 1.557925571 | 0.020393555 |
| 550496 | GO:0006810 | transport | BP | sorting nexin family member 30 | snx30 | 1.557925571 | 0.020393555 |
| 550496 | GO:0005575 | cellular_component | CC | sorting nexin family member 30 | snx30 | 1.557925571 | 0.020393555 |
| 550496 | GO:0035091 | phosphatidylinositol binding | MF | sorting nexin family member 30 | snx30 | 1.557925571 | 0.020393555 |
| 406307 | GO:0006407 | rRNA export from nucleus | BP | ribosomal protein S28 | rps28 | 1.556637426 | 0.020154833 |
| 406307 | GO:0006412 | translation | BP | ribosomal protein S28 | rps28 | 1.556637426 | 0.020154833 |
| 406307 | GO:0022627 | cytosolic small ribosomal subunit | CC | ribosomal protein S28 | rps28 | 1.556637426 | 0.020154833 |
| 406307 | GO:0005622 | intracellular | CC | ribosomal protein S28 | rps28 | 1.556637426 | 0.020154833 |
| 406307 | GO:0030529 | ribonucleoprotein complex | CC | ribosomal protein S28 | rps28 | 1.556637426 | 0.020154833 |
| 406307 | GO:0005840 | ribosome | CC | ribosomal protein S28 | rps28 | 1.556637426 | 0.020154833 |
| 406307 | GO:0003735 | structural constituent of ribosome | MF | ribosomal protein S28 | rps28 | 1.556637426 | 0.020154833 |
| 795901 | GO:0005991 | trehalose metabolic process | BP | trehalase (brush-border membrane glycoprotein) | treh | 1.554841149 | 0.02035651 |
| 795901 | GO:0004555 | alpha,alpha-trehalase activity | MF | trehalase (brush-border membrane glycoprotein) | treh | 1.554841149 | 0.02035651 |
| 795901 | GO:0003824 | catalytic activity | MF | trehalase (brush-border membrane glycoprotein) | treh | 1.554841149 | 0.02035651 |
| 406732 | GO:0006355 | regulation of transcription, DNA-dependent | BP | mediator complex subunit 21 | med21 | 1.554033786 | 0.02316721 |
| 406732 | GO:0006351 | transcription, DNA-dependent | BP | mediator complex subunit 21 | med21 | 1.554033786 | 0.02316721 |
| 406732 | GO:0005634 | nucleus | CC | mediator complex subunit 21 | med21 | 1.554033786 | 0.02316721 |
| 556600 | GO:0005576 | extracellular region | CC | WAP four-disulfide core domain 2 | wfdc2 | 1.547475851 | 0.022269848 |
| 556600 | GO:0030414 | peptidase inhibitor activity | MF | WAP four-disulfide core domain 2 | wfdc2 | 1.547475851 | 0.022269848 |
| 81534 | GO:0016020 | membrane | CC | stomatin | stom | 1.542673087 | 0.021339188 |
| 81534 | GO:0003674 | molecular_function | MF | stomatin | stom | 1.542673087 | 0.021339188 |
| 445165 | GO:0005975 | carbohydrate metabolic process | BP | zgc:101116 | zgc:101116 | 1.53887011 | 0.022014126 |
| 445165 | GO:0006004 | fucose metabolic process | BP | zgc:101116 | zgc:101116 | 1.53887011 | 0.022014126 |
| 445165 | GO:0005575 | cellular_component | CC | zgc:101116 | zgc:101116 | 1.53887011 | 0.022014126 |
| 445165 | GO:0004560 | alpha-L-fucosidase activity | MF | zgc:101116 | zgc:101116 | 1.53887011 | 0.022014126 |
| 445165 | GO:0003824 | catalytic activity | MF | zgc:101116 | zgc:101116 | 1.53887011 | 0.022014126 |
| 445165 | GO:0043169 | cation binding | MF | zgc:101116 | zgc:101116 | 1.53887011 | 0.022014126 |
| 100033674 | GO:0035195 | gene silencing by miRNA | BP | microRNA 150 | mir150 | 1.526666931 | 0.029353082 |
| 767758 | GO:0006412 | translation | BP | mitochondrial ribosomal protein L35 | mrpl35 | 1.509513934 | 0.024169425 |
| 767758 | GO:0005622 | intracellular | CC | mitochondrial ribosomal protein L35 | mrpl35 | 1.509513934 | 0.024169425 |
| 767758 | GO:0005840 | ribosome | CC | mitochondrial ribosomal protein L35 | mrpl35 | 1.509513934 | 0.024169425 |
| 767758 | GO:0003735 | structural constituent of ribosome | MF | mitochondrial ribosomal protein L35 | mrpl35 | 1.509513934 | 0.024169425 |
| 326746 | GO:0005634 | nucleus | CC | chromobox homolog 1a (HP1 beta homolog Drosophila) | cbx1a | 1.505727146 | 0.024250048 |
| 561606 | GO:0046983 | protein dimerization activity | MF | atonal homolog 8 | atoh8 | 1.497506792 | 0.025070909 |
| 556292 | GO:0008150 | biological_process | BP | zgc:171901 | zgc:171901 | 1.496707195 | 0.026621312 |
| 556292 | GO:0005622 | intracellular | CC | zgc:171901 | zgc:171901 | 1.496707195 | 0.026621312 |
| 556292 | GO:0003676 | nucleic acid binding | MF | zgc:171901 | zgc:171901 | 1.496707195 | 0.026621312 |
| 556292 | GO:0008270 | zinc ion binding | MF | zgc:171901 | zgc:171901 | 1.496707195 | 0.026621312 |
| 799071 | GO:0016310 | phosphorylation | BP | aarF domain containing kinase 3 | adck3 | 1.494510235 | 0.025094471 |
| 799071 | GO:0005739 | mitochondrion | CC | aarF domain containing kinase 3 | adck3 | 1.494510235 | 0.025094471 |
| 799071 | GO:0005524 | ATP binding | MF | aarF domain containing kinase 3 | adck3 | 1.494510235 | 0.025094471 |
| 799071 | GO:0016301 | kinase activity | MF | aarF domain containing kinase 3 | adck3 | 1.494510235 | 0.025094471 |
| 799071 | GO:0000166 | nucleotide binding | MF | aarF domain containing kinase 3 | adck3 | 1.494510235 | 0.025094471 |
| 799071 | GO:0004674 | protein serine/threonine kinase activity | MF | aarF domain containing kinase 3 | adck3 | 1.494510235 | 0.025094471 |
| 799071 | GO:0016740 | transferase activity | MF | aarF domain containing kinase 3 | adck3 | 1.494510235 | 0.025094471 |
| 799071 | GO:0016772 | transferase activity, transferring phosphorus-containing groups | MF | aarF domain containing kinase 3 | adck3 | 1.494510235 | 0.025094471 |
| 406404 | GO:0006139 | nucleobase-containing compound metabolic process | BP | adenylate kinase 3 | ak3 | 1.489929162 | 0.025990254 |
| 406404 | GO:0009165 | nucleotide biosynthetic process | BP | adenylate kinase 3 | ak3 | 1.489929162 | 0.025990254 |
| 406404 | GO:0046939 | nucleotide phosphorylation | BP | adenylate kinase 3 | ak3 | 1.489929162 | 0.025990254 |
| 406404 | GO:0016310 | phosphorylation | BP | adenylate kinase 3 | ak3 | 1.489929162 | 0.025990254 |
| 406404 | GO:0005737 | cytoplasm | CC | adenylate kinase 3 | ak3 | 1.489929162 | 0.025990254 |
| 406404 | GO:0004017 | adenylate kinase activity | MF | adenylate kinase 3 | ak3 | 1.489929162 | 0.025990254 |
| 406404 | GO:0005524 | ATP binding | MF | adenylate kinase 3 | ak3 | 1.489929162 | 0.025990254 |
| 406404 | GO:0016301 | kinase activity | MF | adenylate kinase 3 | ak3 | 1.489929162 | 0.025990254 |
| 406404 | GO:0019205 | nucleobase-containing compound kinase activity | MF | adenylate kinase 3 | ak3 | 1.489929162 | 0.025990254 |
| 406404 | GO:0000166 | nucleotide binding | MF | adenylate kinase 3 | ak3 | 1.489929162 | 0.025990254 |
| 406404 | GO:0019201 | nucleotide kinase activity | MF | adenylate kinase 3 | ak3 | 1.489929162 | 0.025990254 |
| 406404 | GO:0016776 | phosphotransferase activity, phosphate group as acceptor | MF | adenylate kinase 3 | ak3 | 1.489929162 | 0.025990254 |
| 406404 | GO:0016740 | transferase activity | MF | adenylate kinase 3 | ak3 | 1.489929162 | 0.025990254 |
| 393354 | GO:0006914 | autophagy | BP | CDGSH iron sulfur domain 2 | cisd2 | 1.489848564 | 0.026484859 |
| 393354 | GO:0000422 | mitochondrion degradation | BP | CDGSH iron sulfur domain 2 | cisd2 | 1.489848564 | 0.026484859 |
| 393354 | GO:0010259 | multicellular organismal aging | BP | CDGSH iron sulfur domain 2 | cisd2 | 1.489848564 | 0.026484859 |
| 393354 | GO:0010506 | regulation of autophagy | BP | CDGSH iron sulfur domain 2 | cisd2 | 1.489848564 | 0.026484859 |
| 393354 | GO:0005783 | endoplasmic reticulum | CC | CDGSH iron sulfur domain 2 | cisd2 | 1.489848564 | 0.026484859 |
| 393354 | GO:0005789 | endoplasmic reticulum membrane | CC | CDGSH iron sulfur domain 2 | cisd2 | 1.489848564 | 0.026484859 |
| 393354 | GO:0016021 | integral to membrane | CC | CDGSH iron sulfur domain 2 | cisd2 | 1.489848564 | 0.026484859 |
| 393354 | GO:0043231 | intracellular membrane-bounded organelle | CC | CDGSH iron sulfur domain 2 | cisd2 | 1.489848564 | 0.026484859 |
| 393354 | GO:0016020 | membrane | CC | CDGSH iron sulfur domain 2 | cisd2 | 1.489848564 | 0.026484859 |
| 393354 | GO:0005741 | mitochondrial outer membrane | CC | CDGSH iron sulfur domain 2 | cisd2 | 1.489848564 | 0.026484859 |
| 393354 | GO:0005739 | mitochondrion | CC | CDGSH iron sulfur domain 2 | cisd2 | 1.489848564 | 0.026484859 |
| 393354 | GO:0051537 | 2 iron, 2 sulfur cluster binding | MF | CDGSH iron sulfur domain 2 | cisd2 | 1.489848564 | 0.026484859 |
| 393354 | GO:0051536 | iron-sulfur cluster binding | MF | CDGSH iron sulfur domain 2 | cisd2 | 1.489848564 | 0.026484859 |
| 393354 | GO:0046872 | metal ion binding | MF | CDGSH iron sulfur domain 2 | cisd2 | 1.489848564 | 0.026484859 |
| 393354 | GO:0042803 | protein homodimerization activity | MF | CDGSH iron sulfur domain 2 | cisd2 | 1.489848564 | 0.026484859 |
| 100038768 | GO:0008150 | biological_process | BP | zgc:162623 | zgc:162623 | 1.483368758 | 0.026233391 |
| 100038768 | GO:0005622 | intracellular | CC | zgc:162623 | zgc:162623 | 1.483368758 | 0.026233391 |
| 100038768 | GO:0003676 | nucleic acid binding | MF | zgc:162623 | zgc:162623 | 1.483368758 | 0.026233391 |
| 100038768 | GO:0008270 | zinc ion binding | MF | zgc:162623 | zgc:162623 | 1.483368758 | 0.026233391 |
| 567738 | GO:0003677 | DNA binding | MF | F-box protein 21 | fbxo21 | 1.474311655 | 0.028115374 |
| 553544 | GO:0006281 | DNA repair | BP | non-SMC element 4 homolog A (S. cerevisiae) | nsmce4a | 1.472135388 | 0.027644356 |
| 553544 | GO:0005634 | nucleus | CC | non-SMC element 4 homolog A (S. cerevisiae) | nsmce4a | 1.472135388 | 0.027644356 |
| 553544 | GO:0030915 | Smc5-Smc6 complex | CC | non-SMC element 4 homolog A (S. cerevisiae) | nsmce4a | 1.472135388 | 0.027644356 |
| 100170819 | GO:0005622 | intracellular | CC | si:dkeyp-2e4.6 | si:dkeyp-2e4.6 | 1.469008783 | 0.032397774 |
| 100170819 | GO:0003676 | nucleic acid binding | MF | si:dkeyp-2e4.6 | si:dkeyp-2e4.6 | 1.469008783 | 0.032397774 |
| 100170819 | GO:0008270 | zinc ion binding | MF | si:dkeyp-2e4.6 | si:dkeyp-2e4.6 | 1.469008783 | 0.032397774 |
| 100007978 | GO:0030133 | transport vesicle | CC | si:dkey-261i16.5 | si:dkey-261i16.5 | 1.462534262 | 0.029219235 |
| 613165 | GO:0007166 | cell surface receptor signaling pathway | BP | si:dkey-30j22.4 | si:dkey-30j22.4 | 1.460178721 | 0.029359317 |
| 613165 | GO:0007186 | G-protein coupled receptor signaling pathway | BP | si:dkey-30j22.4 | si:dkey-30j22.4 | 1.460178721 | 0.029359317 |
| 613165 | GO:0016021 | integral to membrane | CC | si:dkey-30j22.4 | si:dkey-30j22.4 | 1.460178721 | 0.029359317 |
| 613165 | GO:0016020 | membrane | CC | si:dkey-30j22.4 | si:dkey-30j22.4 | 1.460178721 | 0.029359317 |
| 613165 | GO:0005886 | plasma membrane | CC | si:dkey-30j22.4 | si:dkey-30j22.4 | 1.460178721 | 0.029359317 |
| 613165 | GO:0004930 | G-protein coupled receptor activity | MF | si:dkey-30j22.4 | si:dkey-30j22.4 | 1.460178721 | 0.029359317 |
| 613165 | GO:0004888 | transmembrane signaling receptor activity | MF | si:dkey-30j22.4 | si:dkey-30j22.4 | 1.460178721 | 0.029359317 |
| 554148 | GO:0005575 | cellular_component | CC | PDZ and LIM domain 3a | pdlim3a | 1.459136556 | 0.02978512 |
| 554148 | GO:0046872 | metal ion binding | MF | PDZ and LIM domain 3a | pdlim3a | 1.459136556 | 0.02978512 |
| 554148 | GO:0008270 | zinc ion binding | MF | PDZ and LIM domain 3a | pdlim3a | 1.459136556 | 0.02978512 |
| 406291 | GO:0007049 | cell cycle | BP | katanin p80 (WD repeat containing) subunit B 1 | katnb1 | 1.45300642 | 0.029906828 |
| 406291 | GO:0051301 | cell division | BP | katanin p80 (WD repeat containing) subunit B 1 | katnb1 | 1.45300642 | 0.029906828 |
| 406291 | GO:0051013 | microtubule severing | BP | katanin p80 (WD repeat containing) subunit B 1 | katnb1 | 1.45300642 | 0.029906828 |
| 406291 | GO:0007067 | mitosis | BP | katanin p80 (WD repeat containing) subunit B 1 | katnb1 | 1.45300642 | 0.029906828 |
| 406291 | GO:0005737 | cytoplasm | CC | katanin p80 (WD repeat containing) subunit B 1 | katnb1 | 1.45300642 | 0.029906828 |
| 406291 | GO:0005856 | cytoskeleton | CC | katanin p80 (WD repeat containing) subunit B 1 | katnb1 | 1.45300642 | 0.029906828 |
| 406291 | GO:0008352 | katanin complex | CC | katanin p80 (WD repeat containing) subunit B 1 | katnb1 | 1.45300642 | 0.029906828 |
| 406291 | GO:0005874 | microtubule | CC | katanin p80 (WD repeat containing) subunit B 1 | katnb1 | 1.45300642 | 0.029906828 |
| 406291 | GO:0000922 | spindle pole | CC | katanin p80 (WD repeat containing) subunit B 1 | katnb1 | 1.45300642 | 0.029906828 |
| 406291 | GO:0008017 | microtubule binding | MF | katanin p80 (WD repeat containing) subunit B 1 | katnb1 | 1.45300642 | 0.029906828 |
| 402985 | GO:0008152 | metabolic process | BP | glyoxylate reductase/hydroxypyruvate reductase b | grhprb | 1.44793734 | 0.029932607 |
| 402985 | GO:0055114 | oxidation-reduction process | BP | glyoxylate reductase/hydroxypyruvate reductase b | grhprb | 1.44793734 | 0.029932607 |
| 402985 | GO:0005575 | cellular_component | CC | glyoxylate reductase/hydroxypyruvate reductase b | grhprb | 1.44793734 | 0.029932607 |
| 402985 | GO:0048037 | cofactor binding | MF | glyoxylate reductase/hydroxypyruvate reductase b | grhprb | 1.44793734 | 0.029932607 |
| 402985 | GO:0051287 | NAD binding | MF | glyoxylate reductase/hydroxypyruvate reductase b | grhprb | 1.44793734 | 0.029932607 |
| 402985 | GO:0000166 | nucleotide binding | MF | glyoxylate reductase/hydroxypyruvate reductase b | grhprb | 1.44793734 | 0.029932607 |
| 402985 | GO:0016616 | oxidoreductase activity, acting on the CH-OH group of donors, NAD or NADP as acceptor | MF | glyoxylate reductase/hydroxypyruvate reductase b | grhprb | 1.44793734 | 0.029932607 |
| 100415794 | GO:0008152 | metabolic process | BP | UDP glucuronosyltransferase 5 family, polypeptide D1 | ugt5d1 | 1.447098529 | 0.030936834 |
| 100415794 | GO:0016740 | transferase activity | MF | UDP glucuronosyltransferase 5 family, polypeptide D1 | ugt5d1 | 1.447098529 | 0.030936834 |
| 100415794 | GO:0016757 | transferase activity, transferring glycosyl groups | MF | UDP glucuronosyltransferase 5 family, polypeptide D1 | ugt5d1 | 1.447098529 | 0.030936834 |
| 100415794 | GO:0016758 | transferase activity, transferring hexosyl groups | MF | UDP glucuronosyltransferase 5 family, polypeptide D1 | ugt5d1 | 1.447098529 | 0.030936834 |
| 641487 | GO:0008150 | biological_process | BP | zgc:123060 | zgc:123060 | 1.44703776 | 0.03023245 |
| 641487 | GO:0005575 | cellular_component | CC | zgc:123060 | zgc:123060 | 1.44703776 | 0.03023245 |
| 641487 | GO:0003676 | nucleic acid binding | MF | zgc:123060 | zgc:123060 | 1.44703776 | 0.03023245 |
| 568180 | GO:0006950 | response to stress | BP | heat shock protein, alpha-crystallin-related, b3 | hspb3 | 1.434990503 | 0.037332032 |
| 557541 | GO:0042742 | defense response to bacterium | BP | interleukin 1, beta | il1b | 1.432171028 | 0.0326817 |
| 557541 | GO:0006955 | immune response | BP | interleukin 1, beta | il1b | 1.432171028 | 0.0326817 |
| 557541 | GO:0006954 | inflammatory response | BP | interleukin 1, beta | il1b | 1.432171028 | 0.0326817 |
| 557541 | GO:1901222 | regulation of NIK/NF-kappaB cascade | BP | interleukin 1, beta | il1b | 1.432171028 | 0.0326817 |
| 557541 | GO:0009617 | response to bacterium | BP | interleukin 1, beta | il1b | 1.432171028 | 0.0326817 |
| 557541 | GO:0043330 | response to exogenous dsRNA | BP | interleukin 1, beta | il1b | 1.432171028 | 0.0326817 |
| 557541 | GO:0032496 | response to lipopolysaccharide | BP | interleukin 1, beta | il1b | 1.432171028 | 0.0326817 |
| 557541 | GO:0002237 | response to molecule of bacterial origin | BP | interleukin 1, beta | il1b | 1.432171028 | 0.0326817 |
| 557541 | GO:0005576 | extracellular region | CC | interleukin 1, beta | il1b | 1.432171028 | 0.0326817 |
| 557541 | GO:0005615 | extracellular space | CC | interleukin 1, beta | il1b | 1.432171028 | 0.0326817 |
| 557541 | GO:0005125 | cytokine activity | MF | interleukin 1, beta | il1b | 1.432171028 | 0.0326817 |
| 557541 | GO:0005149 | interleukin-1 receptor binding | MF | interleukin 1, beta | il1b | 1.432171028 | 0.0326817 |
| 368253 | GO:0005730 | nucleolus | CC | pin2/trf1-interacting protein 1 | pinx1 | 1.419571161 | 0.034807265 |
| 368253 | GO:0003676 | nucleic acid binding | MF | pin2/trf1-interacting protein 1 | pinx1 | 1.419571161 | 0.034807265 |
| 368253 | GO:0005515 | protein binding | MF | pin2/trf1-interacting protein 1 | pinx1 | 1.419571161 | 0.034807265 |
| 368253 | GO:0010521 | telomerase inhibitor activity | MF | pin2/trf1-interacting protein 1 | pinx1 | 1.419571161 | 0.034807265 |
| 100126013 | GO:0008150 | biological_process | BP | zgc:171682 | zgc:171682 | 1.416156873 | 0.035169233 |
| 100126013 | GO:0005575 | cellular_component | CC | zgc:171682 | zgc:171682 | 1.416156873 | 0.035169233 |
| 100126013 | GO:0003674 | molecular_function | MF | zgc:171682 | zgc:171682 | 1.416156873 | 0.035169233 |
| 568595 | GO:0008150 | biological_process | BP | zgc:171573 | zgc:171573 | 1.413294188 | 0.03521638 |
| 568595 | GO:0005575 | cellular_component | CC | zgc:171573 | zgc:171573 | 1.413294188 | 0.03521638 |
| 568595 | GO:0003674 | molecular_function | MF | zgc:171573 | zgc:171573 | 1.413294188 | 0.03521638 |
| 559769 | GO:0005575 | cellular_component | CC | zgc:153225 | zgc:153225 | 1.409861862 | 0.035207875 |
| 559769 | GO:0003674 | molecular_function | MF | zgc:153225 | zgc:153225 | 1.409861862 | 0.035207875 |
| 100034618 | GO:0006508 | proteolysis | BP | si:dkey-21e2.3 | si:dkey-21e2.3 | 1.404971091 | 0.0377474 |
| 100034618 | GO:0003824 | catalytic activity | MF | si:dkey-21e2.3 | si:dkey-21e2.3 | 1.404971091 | 0.0377474 |
| 100034618 | GO:0016787 | hydrolase activity | MF | si:dkey-21e2.3 | si:dkey-21e2.3 | 1.404971091 | 0.0377474 |
| 100034618 | GO:0008233 | peptidase activity | MF | si:dkey-21e2.3 | si:dkey-21e2.3 | 1.404971091 | 0.0377474 |
| 100034618 | GO:0004252 | serine-type endopeptidase activity | MF | si:dkey-21e2.3 | si:dkey-21e2.3 | 1.404971091 | 0.0377474 |
| 100034618 | GO:0008236 | serine-type peptidase activity | MF | si:dkey-21e2.3 | si:dkey-21e2.3 | 1.404971091 | 0.0377474 |
| 606646 | GO:0008150 | biological_process | BP | im:7160159 | im:7160159 | 1.40276384 | 0.036272563 |
| 606646 | GO:0005575 | cellular_component | CC | im:7160159 | im:7160159 | 1.40276384 | 0.036272563 |
| 606646 | GO:0003674 | molecular_function | MF | im:7160159 | im:7160159 | 1.40276384 | 0.036272563 |
| 325292 | GO:0016428 | tRNA (cytosine-5-)-methyltransferase activity | MF | NOL1/NOP2/Sun domain family, member 2 | nsun2 | 1.398579838 | 0.035840543 |
| 334588 | GO:0005840 | ribosome | CC | mitochondrial ribosomal protein L39 | mrpl39 | 1.395362113 | 0.037556259 |
| 334588 | GO:0000166 | nucleotide binding | MF | mitochondrial ribosomal protein L39 | mrpl39 | 1.395362113 | 0.037556259 |
| 100126105 | GO:0008150 | biological_process | BP | zgc:171915 | zgc:171915 | 1.394556427 | 0.036482282 |
| 100126105 | GO:0005575 | cellular_component | CC | zgc:171915 | zgc:171915 | 1.394556427 | 0.036482282 |
| 563892 | GO:0001525 | angiogenesis | BP | caldesmon 1 | cald1 | 1.39071467 | 0.03717353 |
| 563892 | GO:0001570 | vasculogenesis | BP | caldesmon 1 | cald1 | 1.39071467 | 0.03717353 |
| 563892 | GO:0003779 | actin binding | MF | caldesmon 1 | cald1 | 1.39071467 | 0.03717353 |
| 563892 | GO:0005516 | calmodulin binding | MF | caldesmon 1 | cald1 | 1.39071467 | 0.03717353 |
| 563892 | GO:0017022 | myosin binding | MF | caldesmon 1 | cald1 | 1.39071467 | 0.03717353 |
| 563892 | GO:0032036 | myosin heavy chain binding | MF | caldesmon 1 | cald1 | 1.39071467 | 0.03717353 |
| 30315 | GO:0045893 | positive regulation of transcription, DNA-dependent | BP | forkhead box N4 | foxn4 | 1.3903511 | 0.037143704 |
| 30315 | GO:0051090 | regulation of sequence-specific DNA binding transcription factor activity | BP | forkhead box N4 | foxn4 | 1.3903511 | 0.037143704 |
| 30315 | GO:0006357 | regulation of transcription from RNA polymerase II promoter | BP | forkhead box N4 | foxn4 | 1.3903511 | 0.037143704 |
| 30315 | GO:0006355 | regulation of transcription, DNA-dependent | BP | forkhead box N4 | foxn4 | 1.3903511 | 0.037143704 |
| 30315 | GO:0006351 | transcription, DNA-dependent | BP | forkhead box N4 | foxn4 | 1.3903511 | 0.037143704 |
| 30315 | GO:0005634 | nucleus | CC | forkhead box N4 | foxn4 | 1.3903511 | 0.037143704 |
| 30315 | GO:0005667 | transcription factor complex | CC | forkhead box N4 | foxn4 | 1.3903511 | 0.037143704 |
| 30315 | GO:0003677 | DNA binding | MF | forkhead box N4 | foxn4 | 1.3903511 | 0.037143704 |
| 30315 | GO:0008301 | DNA binding, bending | MF | forkhead box N4 | foxn4 | 1.3903511 | 0.037143704 |
| 30315 | GO:0003690 | double-stranded DNA binding | MF | forkhead box N4 | foxn4 | 1.3903511 | 0.037143704 |
| 30315 | GO:0003705 | RNA polymerase II distal enhancer sequence-specific DNA binding transcription factor activity | MF | forkhead box N4 | foxn4 | 1.3903511 | 0.037143704 |
| 30315 | GO:0043565 | sequence-specific DNA binding | MF | forkhead box N4 | foxn4 | 1.3903511 | 0.037143704 |
| 30315 | GO:0003700 | sequence-specific DNA binding transcription factor activity | MF | forkhead box N4 | foxn4 | 1.3903511 | 0.037143704 |
| 30315 | GO:0008134 | transcription factor binding | MF | forkhead box N4 | foxn4 | 1.3903511 | 0.037143704 |
| 334529 | GO:0006810 | transport | BP | aquaporin 7 | aqp7 | 1.390173229 | 0.038429189 |
| 334529 | GO:0016021 | integral to membrane | CC | aquaporin 7 | aqp7 | 1.390173229 | 0.038429189 |
| 334529 | GO:0016020 | membrane | CC | aquaporin 7 | aqp7 | 1.390173229 | 0.038429189 |
| 334529 | GO:0015168 | glycerol transmembrane transporter activity | MF | aquaporin 7 | aqp7 | 1.390173229 | 0.038429189 |
| 334529 | GO:0005215 | transporter activity | MF | aquaporin 7 | aqp7 | 1.390173229 | 0.038429189 |
| 334529 | GO:0015204 | urea transmembrane transporter activity | MF | aquaporin 7 | aqp7 | 1.390173229 | 0.038429189 |
| 334529 | GO:0005372 | water transmembrane transporter activity | MF | aquaporin 7 | aqp7 | 1.390173229 | 0.038429189 |
| 548346 | GO:0046854 | phosphatidylinositol phosphorylation | BP | phosphoinositide-3-kinase, class 3 | pik3c3 | 1.388526243 | 0.037486216 |
| 548346 | GO:0048015 | phosphatidylinositol-mediated signaling | BP | phosphoinositide-3-kinase, class 3 | pik3c3 | 1.388526243 | 0.037486216 |
| 548346 | GO:0016310 | phosphorylation | BP | phosphoinositide-3-kinase, class 3 | pik3c3 | 1.388526243 | 0.037486216 |
| 548346 | GO:0016303 | 1-phosphatidylinositol-3-kinase activity | MF | phosphoinositide-3-kinase, class 3 | pik3c3 | 1.388526243 | 0.037486216 |
| 548346 | GO:0005524 | ATP binding | MF | phosphoinositide-3-kinase, class 3 | pik3c3 | 1.388526243 | 0.037486216 |
| 548346 | GO:0016301 | kinase activity | MF | phosphoinositide-3-kinase, class 3 | pik3c3 | 1.388526243 | 0.037486216 |
| 548346 | GO:0000166 | nucleotide binding | MF | phosphoinositide-3-kinase, class 3 | pik3c3 | 1.388526243 | 0.037486216 |
| 548346 | GO:0016773 | phosphotransferase activity, alcohol group as acceptor | MF | phosphoinositide-3-kinase, class 3 | pik3c3 | 1.388526243 | 0.037486216 |
| 548346 | GO:0016740 | transferase activity | MF | phosphoinositide-3-kinase, class 3 | pik3c3 | 1.388526243 | 0.037486216 |
| 548346 | GO:0016772 | transferase activity, transferring phosphorus-containing groups | MF | phosphoinositide-3-kinase, class 3 | pik3c3 | 1.388526243 | 0.037486216 |
| 562772 | GO:0006810 | transport | BP | solute carrier organic anion transporter family, member 1C1 | slco1c1 | 1.383915333 | 0.038247057 |
| 562772 | GO:0016020 | membrane | CC | solute carrier organic anion transporter family, member 1C1 | slco1c1 | 1.383915333 | 0.038247057 |
| 562772 | GO:0005215 | transporter activity | MF | solute carrier organic anion transporter family, member 1C1 | slco1c1 | 1.383915333 | 0.038247057 |
| 30503 | GO:0007186 | G-protein coupled receptor signaling pathway | BP | opsin 1 (cone pigments), medium-wave-sensitive, 1 | opn1mw1 | 1.380736466 | 0.039052789 |
| 30503 | GO:0007165 | signal transduction | BP | opsin 1 (cone pigments), medium-wave-sensitive, 1 | opn1mw1 | 1.380736466 | 0.039052789 |
| 30503 | GO:0016021 | integral to membrane | CC | opsin 1 (cone pigments), medium-wave-sensitive, 1 | opn1mw1 | 1.380736466 | 0.039052789 |
| 30503 | GO:0016020 | membrane | CC | opsin 1 (cone pigments), medium-wave-sensitive, 1 | opn1mw1 | 1.380736466 | 0.039052789 |
| 30503 | GO:0004930 | G-protein coupled receptor activity | MF | opsin 1 (cone pigments), medium-wave-sensitive, 1 | opn1mw1 | 1.380736466 | 0.039052789 |
| 30503 | GO:0004871 | signal transducer activity | MF | opsin 1 (cone pigments), medium-wave-sensitive, 1 | opn1mw1 | 1.380736466 | 0.039052789 |
| 406484 | GO:0005576 | extracellular region | CC | zgc:77778 | zgc:77778 | 1.378801016 | 0.041993834 |
| 406484 | GO:0005615 | extracellular space | CC | zgc:77778 | zgc:77778 | 1.378801016 | 0.041993834 |
| 406484 | GO:0003674 | molecular_function | MF | zgc:77778 | zgc:77778 | 1.378801016 | 0.041993834 |
| 445256 | GO:0071569 | protein ufmylation | BP | ubiquitin-fold modifier conjugating enzyme 1 | ufc1 | 1.378722496 | 0.047257366 |
| 445256 | GO:0005575 | cellular_component | CC | ubiquitin-fold modifier conjugating enzyme 1 | ufc1 | 1.378722496 | 0.047257366 |
| 445256 | GO:0071568 | UFM1 conjugating enzyme activity | MF | ubiquitin-fold modifier conjugating enzyme 1 | ufc1 | 1.378722496 | 0.047257366 |
| 494108 | GO:0055114 | oxidation-reduction process | BP | prostaglandin reductase 1 | ptgr1 | 1.373214563 | 0.042633356 |
| 494108 | GO:0005575 | cellular_component | CC | prostaglandin reductase 1 | ptgr1 | 1.373214563 | 0.042633356 |
| 494108 | GO:0000166 | nucleotide binding | MF | prostaglandin reductase 1 | ptgr1 | 1.373214563 | 0.042633356 |
| 494108 | GO:0016491 | oxidoreductase activity | MF | prostaglandin reductase 1 | ptgr1 | 1.373214563 | 0.042633356 |
| 494108 | GO:0008270 | zinc ion binding | MF | prostaglandin reductase 1 | ptgr1 | 1.373214563 | 0.042633356 |
| 449558 | GO:0008152 | metabolic process | BP | zgc:101723 | zgc:101723 | 1.373090724 | 0.03935632 |
| 449558 | GO:0055114 | oxidation-reduction process | BP | zgc:101723 | zgc:101723 | 1.373090724 | 0.03935632 |
| 449558 | GO:0005575 | cellular_component | CC | zgc:101723 | zgc:101723 | 1.373090724 | 0.03935632 |
| 449558 | GO:0047837 | D-xylose 1-dehydrogenase (NADP+) activity | MF | zgc:101723 | zgc:101723 | 1.373090724 | 0.03935632 |
| 449558 | GO:0000166 | nucleotide binding | MF | zgc:101723 | zgc:101723 | 1.373090724 | 0.03935632 |
| 449558 | GO:0016491 | oxidoreductase activity | MF | zgc:101723 | zgc:101723 | 1.373090724 | 0.03935632 |
| 449679 | GO:0005901 | caveola | CC | caveolin 3 | cav3 | 1.371338163 | 0.039276065 |
| 449679 | GO:0005794 | Golgi apparatus | CC | caveolin 3 | cav3 | 1.371338163 | 0.039276065 |
| 449679 | GO:0000139 | Golgi membrane | CC | caveolin 3 | cav3 | 1.371338163 | 0.039276065 |
| 449679 | GO:0016020 | membrane | CC | caveolin 3 | cav3 | 1.371338163 | 0.039276065 |
| 449679 | GO:0005886 | plasma membrane | CC | caveolin 3 | cav3 | 1.371338163 | 0.039276065 |
| 449679 | GO:0003674 | molecular_function | MF | caveolin 3 | cav3 | 1.371338163 | 0.039276065 |
| 407736 | GO:0006810 | transport | BP | fatty acid binding protein 7, brain, b | fabp7b | 1.370564097 | 0.041916914 |
| 407736 | GO:0005504 | fatty acid binding | MF | fatty acid binding protein 7, brain, b | fabp7b | 1.370564097 | 0.041916914 |
| 407736 | GO:0008289 | lipid binding | MF | fatty acid binding protein 7, brain, b | fabp7b | 1.370564097 | 0.041916914 |
| 407736 | GO:0005215 | transporter activity | MF | fatty acid binding protein 7, brain, b | fabp7b | 1.370564097 | 0.041916914 |
| 494047 | GO:0008150 | biological_process | BP | solute carrier family 35, member C1 | slc35c1 | 1.369669611 | 0.040779529 |
| 494047 | GO:0016021 | integral to membrane | CC | solute carrier family 35, member C1 | slc35c1 | 1.369669611 | 0.040779529 |
| 494047 | GO:0016020 | membrane | CC | solute carrier family 35, member C1 | slc35c1 | 1.369669611 | 0.040779529 |
| 494047 | GO:0003674 | molecular_function | MF | solute carrier family 35, member C1 | slc35c1 | 1.369669611 | 0.040779529 |
| 445404 | GO:0006396 | RNA processing | BP | small nuclear ribonucleoprotein polypeptide F | snrpf | 1.366538913 | 0.046367594 |
| 445404 | GO:0005634 | nucleus | CC | small nuclear ribonucleoprotein polypeptide F | snrpf | 1.366538913 | 0.046367594 |
| 445404 | GO:0030529 | ribonucleoprotein complex | CC | small nuclear ribonucleoprotein polypeptide F | snrpf | 1.366538913 | 0.046367594 |
| 445404 | GO:0003676 | nucleic acid binding | MF | small nuclear ribonucleoprotein polypeptide F | snrpf | 1.366538913 | 0.046367594 |
| 368771 | GO:0006200 | ATP catabolic process | BP | ATP-binding cassette, sub-family B (MDR/TAP), member 3 like 1 | abcb3l1 | 1.364206565 | 0.041133652 |
| 368771 | GO:0035672 | oligopeptide transmembrane transport | BP | ATP-binding cassette, sub-family B (MDR/TAP), member 3 like 1 | abcb3l1 | 1.364206565 | 0.041133652 |
| 368771 | GO:0015833 | peptide transport | BP | ATP-binding cassette, sub-family B (MDR/TAP), member 3 like 1 | abcb3l1 | 1.364206565 | 0.041133652 |
| 368771 | GO:0046686 | response to cadmium ion | BP | ATP-binding cassette, sub-family B (MDR/TAP), member 3 like 1 | abcb3l1 | 1.364206565 | 0.041133652 |
| 368771 | GO:0055085 | transmembrane transport | BP | ATP-binding cassette, sub-family B (MDR/TAP), member 3 like 1 | abcb3l1 | 1.364206565 | 0.041133652 |
| 368771 | GO:0006810 | transport | BP | ATP-binding cassette, sub-family B (MDR/TAP), member 3 like 1 | abcb3l1 | 1.364206565 | 0.041133652 |
| 368771 | GO:0016021 | integral to membrane | CC | ATP-binding cassette, sub-family B (MDR/TAP), member 3 like 1 | abcb3l1 | 1.364206565 | 0.041133652 |
| 368771 | GO:0005743 | mitochondrial inner membrane | CC | ATP-binding cassette, sub-family B (MDR/TAP), member 3 like 1 | abcb3l1 | 1.364206565 | 0.041133652 |
| 368771 | GO:0005886 | plasma membrane | CC | ATP-binding cassette, sub-family B (MDR/TAP), member 3 like 1 | abcb3l1 | 1.364206565 | 0.041133652 |
| 368771 | GO:0042825 | TAP complex | CC | ATP-binding cassette, sub-family B (MDR/TAP), member 3 like 1 | abcb3l1 | 1.364206565 | 0.041133652 |
| 368771 | GO:0005524 | ATP binding | MF | ATP-binding cassette, sub-family B (MDR/TAP), member 3 like 1 | abcb3l1 | 1.364206565 | 0.041133652 |
| 368771 | GO:0016887 | ATPase activity | MF | ATP-binding cassette, sub-family B (MDR/TAP), member 3 like 1 | abcb3l1 | 1.364206565 | 0.041133652 |
| 368771 | GO:0042626 | ATPase activity, coupled to transmembrane movement of substances | MF | ATP-binding cassette, sub-family B (MDR/TAP), member 3 like 1 | abcb3l1 | 1.364206565 | 0.041133652 |
| 368771 | GO:0042288 | MHC class I protein binding | MF | ATP-binding cassette, sub-family B (MDR/TAP), member 3 like 1 | abcb3l1 | 1.364206565 | 0.041133652 |
| 368771 | GO:0017111 | nucleoside-triphosphatase activity | MF | ATP-binding cassette, sub-family B (MDR/TAP), member 3 like 1 | abcb3l1 | 1.364206565 | 0.041133652 |
| 368771 | GO:0000166 | nucleotide binding | MF | ATP-binding cassette, sub-family B (MDR/TAP), member 3 like 1 | abcb3l1 | 1.364206565 | 0.041133652 |
| 368771 | GO:0015421 | oligopeptide-transporting ATPase activity | MF | ATP-binding cassette, sub-family B (MDR/TAP), member 3 like 1 | abcb3l1 | 1.364206565 | 0.041133652 |
| 368771 | GO:0042605 | peptide antigen binding | MF | ATP-binding cassette, sub-family B (MDR/TAP), member 3 like 1 | abcb3l1 | 1.364206565 | 0.041133652 |
| 368771 | GO:0046978 | TAP1 binding | MF | ATP-binding cassette, sub-family B (MDR/TAP), member 3 like 1 | abcb3l1 | 1.364206565 | 0.041133652 |
| 368771 | GO:0046979 | TAP2 binding | MF | ATP-binding cassette, sub-family B (MDR/TAP), member 3 like 1 | abcb3l1 | 1.364206565 | 0.041133652 |
| 368771 | GO:0046980 | tapasin binding | MF | ATP-binding cassette, sub-family B (MDR/TAP), member 3 like 1 | abcb3l1 | 1.364206565 | 0.041133652 |
| 557652 | GO:0016791 | phosphatase activity | MF | si:dkeyp-27c8.2 | si:dkeyp-27c8.2 | 1.358920222 | 0.044133535 |
| 437025 | GO:0006355 | regulation of transcription, DNA-dependent | BP | YEATS domain containing 4 | yeats4 | 1.358800652 | 0.044026574 |
| 437025 | GO:0005634 | nucleus | CC | YEATS domain containing 4 | yeats4 | 1.358800652 | 0.044026574 |
| 58120 | GO:0006241 | CTP biosynthetic process | BP | non-metastatic cells 6, protein expressed in (nucleoside-diphosphate kinase) | nme6 | 1.353255744 | 0.04383862 |
| 58120 | GO:0006183 | GTP biosynthetic process | BP | non-metastatic cells 6, protein expressed in (nucleoside-diphosphate kinase) | nme6 | 1.353255744 | 0.04383862 |
| 58120 | GO:0006165 | nucleoside diphosphate phosphorylation | BP | non-metastatic cells 6, protein expressed in (nucleoside-diphosphate kinase) | nme6 | 1.353255744 | 0.04383862 |
| 58120 | GO:0009117 | nucleotide metabolic process | BP | non-metastatic cells 6, protein expressed in (nucleoside-diphosphate kinase) | nme6 | 1.353255744 | 0.04383862 |
| 58120 | GO:0016310 | phosphorylation | BP | non-metastatic cells 6, protein expressed in (nucleoside-diphosphate kinase) | nme6 | 1.353255744 | 0.04383862 |
| 58120 | GO:0006228 | UTP biosynthetic process | BP | non-metastatic cells 6, protein expressed in (nucleoside-diphosphate kinase) | nme6 | 1.353255744 | 0.04383862 |
| 58120 | GO:0005739 | mitochondrion | CC | non-metastatic cells 6, protein expressed in (nucleoside-diphosphate kinase) | nme6 | 1.353255744 | 0.04383862 |
| 58120 | GO:0005524 | ATP binding | MF | non-metastatic cells 6, protein expressed in (nucleoside-diphosphate kinase) | nme6 | 1.353255744 | 0.04383862 |
| 58120 | GO:0016301 | kinase activity | MF | non-metastatic cells 6, protein expressed in (nucleoside-diphosphate kinase) | nme6 | 1.353255744 | 0.04383862 |
| 58120 | GO:0046872 | metal ion binding | MF | non-metastatic cells 6, protein expressed in (nucleoside-diphosphate kinase) | nme6 | 1.353255744 | 0.04383862 |
| 58120 | GO:0004550 | nucleoside diphosphate kinase activity | MF | non-metastatic cells 6, protein expressed in (nucleoside-diphosphate kinase) | nme6 | 1.353255744 | 0.04383862 |
| 58120 | GO:0000166 | nucleotide binding | MF | non-metastatic cells 6, protein expressed in (nucleoside-diphosphate kinase) | nme6 | 1.353255744 | 0.04383862 |
| 58120 | GO:0016740 | transferase activity | MF | non-metastatic cells 6, protein expressed in (nucleoside-diphosphate kinase) | nme6 | 1.353255744 | 0.04383862 |
| 30526 | GO:0006355 | regulation of transcription, DNA-dependent | BP | muscle segment homeobox C | msxc | 1.350721539 | 0.044582656 |
| 30526 | GO:0005634 | nucleus | CC | muscle segment homeobox C | msxc | 1.350721539 | 0.044582656 |
| 30526 | GO:0003677 | DNA binding | MF | muscle segment homeobox C | msxc | 1.350721539 | 0.044582656 |
| 30526 | GO:0043565 | sequence-specific DNA binding | MF | muscle segment homeobox C | msxc | 1.350721539 | 0.044582656 |
| 30526 | GO:0003700 | sequence-specific DNA binding transcription factor activity | MF | muscle segment homeobox C | msxc | 1.350721539 | 0.044582656 |
| 678641 | GO:0008150 | biological_process | BP | disabled homolog 1b (Drosophila) | dab1b | 1.350092812 | 0.042809788 |
| 678641 | GO:0005575 | cellular_component | CC | disabled homolog 1b (Drosophila) | dab1b | 1.350092812 | 0.042809788 |
| 678641 | GO:0003674 | molecular_function | MF | disabled homolog 1b (Drosophila) | dab1b | 1.350092812 | 0.042809788 |
| 327572 | GO:0051056 | regulation of small GTPase mediated signal transduction | BP | RasGEF domain family, member 1Ba | rasgef1ba | 1.345031417 | 0.043561388 |
| 327572 | GO:0007264 | small GTPase mediated signal transduction | BP | RasGEF domain family, member 1Ba | rasgef1ba | 1.345031417 | 0.043561388 |
| 327572 | GO:0005622 | intracellular | CC | RasGEF domain family, member 1Ba | rasgef1ba | 1.345031417 | 0.043561388 |
| 327572 | GO:0005085 | guanyl-nucleotide exchange factor activity | MF | RasGEF domain family, member 1Ba | rasgef1ba | 1.345031417 | 0.043561388 |
| 327572 | GO:0005088 | Ras guanyl-nucleotide exchange factor activity | MF | RasGEF domain family, member 1Ba | rasgef1ba | 1.345031417 | 0.043561388 |
| 561172 | GO:0006486 | protein glycosylation | BP | zgc:171538 | zgc:171538 | 1.34324087 | 0.04457102 |
| 561172 | GO:0005794 | Golgi apparatus | CC | zgc:171538 | zgc:171538 | 1.34324087 | 0.04457102 |
| 561172 | GO:0016021 | integral to membrane | CC | zgc:171538 | zgc:171538 | 1.34324087 | 0.04457102 |
| 561172 | GO:0016020 | membrane | CC | zgc:171538 | zgc:171538 | 1.34324087 | 0.04457102 |
| 561172 | GO:0008378 | galactosyltransferase activity | MF | zgc:171538 | zgc:171538 | 1.34324087 | 0.04457102 |
| 561172 | GO:0016740 | transferase activity | MF | zgc:171538 | zgc:171538 | 1.34324087 | 0.04457102 |
| 561172 | GO:0016757 | transferase activity, transferring glycosyl groups | MF | zgc:171538 | zgc:171538 | 1.34324087 | 0.04457102 |
| 403068 | GO:0009058 | biosynthetic process | BP | asparagine-linked glycosylation 2 homolog (S. cerevisiae, alpha-1,3-mannosyltransferase) | alg2 | 1.336492058 | 0.044647733 |
| 403068 | GO:0005634 | nucleus | CC | asparagine-linked glycosylation 2 homolog (S. cerevisiae, alpha-1,3-mannosyltransferase) | alg2 | 1.336492058 | 0.044647733 |
| 403068 | GO:0004376 | glycolipid mannosyltransferase activity | MF | asparagine-linked glycosylation 2 homolog (S. cerevisiae, alpha-1,3-mannosyltransferase) | alg2 | 1.336492058 | 0.044647733 |
| 30641 | GO:0030509 | BMP signaling pathway | BP | MAD homolog 5 (Drosophila) | smad5 | 1.33394468 | 0.045971098 |
| 30641 | GO:0006355 | regulation of transcription, DNA-dependent | BP | MAD homolog 5 (Drosophila) | smad5 | 1.33394468 | 0.045971098 |
| 30641 | GO:0006351 | transcription, DNA-dependent | BP | MAD homolog 5 (Drosophila) | smad5 | 1.33394468 | 0.045971098 |
| 30641 | GO:0007179 | transforming growth factor beta receptor signaling pathway | BP | MAD homolog 5 (Drosophila) | smad5 | 1.33394468 | 0.045971098 |
| 30641 | GO:0005737 | cytoplasm | CC | MAD homolog 5 (Drosophila) | smad5 | 1.33394468 | 0.045971098 |
| 30641 | GO:0005622 | intracellular | CC | MAD homolog 5 (Drosophila) | smad5 | 1.33394468 | 0.045971098 |
| 30641 | GO:0005634 | nucleus | CC | MAD homolog 5 (Drosophila) | smad5 | 1.33394468 | 0.045971098 |
| 30641 | GO:0005667 | transcription factor complex | CC | MAD homolog 5 (Drosophila) | smad5 | 1.33394468 | 0.045971098 |
| 30641 | GO:0003700 | sequence-specific DNA binding transcription factor activity | MF | MAD homolog 5 (Drosophila) | smad5 | 1.33394468 | 0.045971098 |
| 368511 | GO:0016021 | integral to membrane | CC | transmembrane and ubiquitin-like domain containing 2 | tmub2 | 1.333685433 | 0.04602124 |
| 550288 | GO:0051726 | regulation of cell cycle | BP | diencephalon/mesencephalon homeobox 1b | dmbx1b | 1.330382699 | 0.046621441 |
| 550288 | GO:0006355 | regulation of transcription, DNA-dependent | BP | diencephalon/mesencephalon homeobox 1b | dmbx1b | 1.330382699 | 0.046621441 |
| 550288 | GO:0005634 | nucleus | CC | diencephalon/mesencephalon homeobox 1b | dmbx1b | 1.330382699 | 0.046621441 |
| 550288 | GO:0003677 | DNA binding | MF | diencephalon/mesencephalon homeobox 1b | dmbx1b | 1.330382699 | 0.046621441 |
| 550288 | GO:0043565 | sequence-specific DNA binding | MF | diencephalon/mesencephalon homeobox 1b | dmbx1b | 1.330382699 | 0.046621441 |
| 550288 | GO:0003700 | sequence-specific DNA binding transcription factor activity | MF | diencephalon/mesencephalon homeobox 1b | dmbx1b | 1.330382699 | 0.046621441 |
| 386808 | GO:0007219 | Notch signaling pathway | BP | anterior pharynx defective 1B | aph1b | 1.325528465 | 0.047928103 |
| 386808 | GO:0016485 | protein processing | BP | anterior pharynx defective 1B | aph1b | 1.325528465 | 0.047928103 |
| 386808 | GO:0016021 | integral to membrane | CC | anterior pharynx defective 1B | aph1b | 1.325528465 | 0.047928103 |
| 386808 | GO:0016020 | membrane | CC | anterior pharynx defective 1B | aph1b | 1.325528465 | 0.047928103 |
| 567117 | GO:0015837 | amine transport | BP | solute carrier family 7 (cationic amino acid transporter, y+ system), member 9 | slc7a9 | 1.322844263 | 0.048491063 |
| 567117 | GO:0003333 | amino acid transmembrane transport | BP | solute carrier family 7 (cationic amino acid transporter, y+ system), member 9 | slc7a9 | 1.322844263 | 0.048491063 |
| 567117 | GO:0016021 | integral to membrane | CC | solute carrier family 7 (cationic amino acid transporter, y+ system), member 9 | slc7a9 | 1.322844263 | 0.048491063 |
| 567117 | GO:0016020 | membrane | CC | solute carrier family 7 (cationic amino acid transporter, y+ system), member 9 | slc7a9 | 1.322844263 | 0.048491063 |
| 567117 | GO:0015171 | amino acid transmembrane transporter activity | MF | solute carrier family 7 (cationic amino acid transporter, y+ system), member 9 | slc7a9 | 1.322844263 | 0.048491063 |
| 393314 | GO:0003676 | nucleic acid binding | MF | protein inhibitor of activated STAT, 4b | pias4b | 1.318297704 | 0.04802586 |
| 393314 | GO:0008270 | zinc ion binding | MF | protein inhibitor of activated STAT, 4b | pias4b | 1.318297704 | 0.04802586 |
| 768205 | GO:0008150 | biological_process | BP | zgc:153987 | zgc:153987 | 1.315200792 | 0.048572995 |
| 768205 | GO:0005575 | cellular_component | CC | zgc:153987 | zgc:153987 | 1.315200792 | 0.048572995 |
| 768205 | GO:0003674 | molecular_function | MF | zgc:153987 | zgc:153987 | 1.315200792 | 0.048572995 |
| 114412 | GO:0005575 | cellular_component | CC | LIM domain only 4a | lmo4a | 1.310831144 | 0.049898233 |
| 114412 | GO:0046872 | metal ion binding | MF | LIM domain only 4a | lmo4a | 1.310831144 | 0.049898233 |
| 114412 | GO:0008270 | zinc ion binding | MF | LIM domain only 4a | lmo4a | 1.310831144 | 0.049898233 |
| 550584 | GO:0006811 | ion transport | BP | cholinergic receptor, nicotinic, alpha 5 | chrna5 | 1.309342555 | 0.049180057 |
| 550584 | GO:0006810 | transport | BP | cholinergic receptor, nicotinic, alpha 5 | chrna5 | 1.309342555 | 0.049180057 |
| 550584 | GO:0030054 | cell junction | CC | cholinergic receptor, nicotinic, alpha 5 | chrna5 | 1.309342555 | 0.049180057 |
| 550584 | GO:0016021 | integral to membrane | CC | cholinergic receptor, nicotinic, alpha 5 | chrna5 | 1.309342555 | 0.049180057 |
| 550584 | GO:0016020 | membrane | CC | cholinergic receptor, nicotinic, alpha 5 | chrna5 | 1.309342555 | 0.049180057 |
| 550584 | GO:0005886 | plasma membrane | CC | cholinergic receptor, nicotinic, alpha 5 | chrna5 | 1.309342555 | 0.049180057 |
| 550584 | GO:0004889 | acetylcholine-activated cation-selective channel activity | MF | cholinergic receptor, nicotinic, alpha 5 | chrna5 | 1.309342555 | 0.049180057 |
| 550584 | GO:0005230 | extracellular ligand-gated ion channel activity | MF | cholinergic receptor, nicotinic, alpha 5 | chrna5 | 1.309342555 | 0.049180057 |
| 550584 | GO:0005216 | ion channel activity | MF | cholinergic receptor, nicotinic, alpha 5 | chrna5 | 1.309342555 | 0.049180057 |
| 571143 | GO:0008152 | metabolic process | BP | matrix metallopeptidase 24 | mmp24 | 1.308477407 | 0.049618959 |
| 571143 | GO:0006508 | proteolysis | BP | matrix metallopeptidase 24 | mmp24 | 1.308477407 | 0.049618959 |
| 571143 | GO:0031012 | extracellular matrix | CC | matrix metallopeptidase 24 | mmp24 | 1.308477407 | 0.049618959 |
| 571143 | GO:0005509 | calcium ion binding | MF | matrix metallopeptidase 24 | mmp24 | 1.308477407 | 0.049618959 |
| 571143 | GO:0016787 | hydrolase activity | MF | matrix metallopeptidase 24 | mmp24 | 1.308477407 | 0.049618959 |
| 571143 | GO:0046872 | metal ion binding | MF | matrix metallopeptidase 24 | mmp24 | 1.308477407 | 0.049618959 |
| 571143 | GO:0004222 | metalloendopeptidase activity | MF | matrix metallopeptidase 24 | mmp24 | 1.308477407 | 0.049618959 |
| 571143 | GO:0008237 | metallopeptidase activity | MF | matrix metallopeptidase 24 | mmp24 | 1.308477407 | 0.049618959 |
| 571143 | GO:0008233 | peptidase activity | MF | matrix metallopeptidase 24 | mmp24 | 1.308477407 | 0.049618959 |
| 571143 | GO:0008270 | zinc ion binding | MF | matrix metallopeptidase 24 | mmp24 | 1.308477407 | 0.049618959 |
| 561440 | GO:0005576 | extracellular region | CC | netrin 4 | ntn4 | 1.304379644 | 0.049763953 |
