# Supplementary material for: Functional Categorization of Transcriptome in the Species Symphysodon aequifasciatus Pellegrin 1904 (Perciformes: Cichlidae) Exposed to Benzo[a]pyrene and Phenanthrene
Source: PLoS One. 2013 Dec 3;8(12):e81083. doi: 10.1371/journal.pone.0081083 (PMC3849039; doi:10.1371/journal.pone.0081083)
Supplement: Table S3 — The list of genes in Symphysodon aequifasciatus exposed to benzo[a]pyrene and phenanthrene for 48 h submitted to STRING software (v.9.1). (DOC) [file pone.0081083.s008.doc]

Table S3. The list of genes in *Symphysodon aequifasciatus* exposed to benzo[a]pyrene and phenanthrene for 48h submitted to STRING software (v.9.1).

| gene symbol submitted | STRING protein name | STRING id | Gene name description |
| --- | --- | --- | --- |
| aarsd1 | aarsd1 | 7955.ENSDARP00000011878 | Alanyl-tRNA synthetase domain-containing protein 1 |
| abcb3l1 | abcb3l1 | 7955.ENSDARP00000028924 | ATP-binding cassette, sub-family B (MDR/TAP), member 3 like 1 |
| acat1 | acat1 | 7955.ENSDARP00000067447 | Acetyl-CoA acetyltransferase, mitochondrial Precursor (EC 2.3.1.9)(Acetoacetyl-CoA thiolase); Plays a major role in ketone body metabolism (By similarity) |
| aig1 | aig1 | 7955.ENSDARP00000018086 | Novel protein similar to human and mouse androgen-induced 1 (AIG1) Fragment |
| ak3 | ak3 | 7955.ENSDARP00000075506 | adenylate kinase 3 |
| aldh4a1 | aldh4a1 | 7955.ENSDARP00000055709 | Delta-1-pyrroline-5-carboxylate dehydrogenase, mitochondrial Precursor (P5C dehydrogenase)(EC 1.5.1.12)(Aldehyde dehydrogenase family 4 member A1); Irreversible conversion of delta-1-pyrroline-5- carboxylate (P5C), derived either from proline or ornithine, to glutamate. This is a necessary step in the pathway interconnecting the urea and tricarboxylic acid cycles (By similarity) |
| aldh5a1 | aldh5a1 | 7955.ENSDARP00000012423 | aldehyde dehydrogenase 5 family, member A1 (succinate-semialdehyde dehydrogenase) |
| alg2 | alg2 | 7955.ENSDARP00000094034 | alpha-1,3-mannosyltransferase ALG2 |
| aph1b | aph1b | 7955.ENSDARP00000011743 | Gamma-secretase subunit Aph-1b (Anterior-pharynx-defective protein 1b); Essential subunit of the gamma-secretase complex, an endoprotease complex that catalyzes the intramembrane cleavage of integral proteins such as Notch receptors. It may represent a stabilizing cofactor for the presenilin homodimer that promotes the formation of a stable complex (By similarity) |
| aqp7 | zgc:63700 | 7955.ENSDARP00000037835 | aquaporin 7 |
| arfip1 | arfip1 | 7955.ENSDARP00000093278 | ADP-ribosylation factor interacting protein 1 (arfaptin 1) |
| arl6ip5a | cb937 | 7955.ENSDARP00000098330 | ADP-ribosylation factor-like 6 interacting protein 5 |
| atoh8 | atoh8 | 7955.ENSDARP00000054688 | atonal homolog 8 |
| atp1a1a.4 | atp1a1a.4 | 7955.ENSDARP00000048827 | ATPase, Na+/K+ transporting, alpha 1a.4 polypeptide |
| atp2b3a | atp2b3a | 7955.ENSDARP00000084685 | plasma membrane calcium ATPase 3a |
| atp5d | atp5d | 7955.ENSDARP00000022528 | ATP synthase, H+ transporting, mitochondrial F1 complex, delta subunit |
| bin2a | bin2a | 7955.ENSDARP00000019400 | bridging integrator 2a |
| cav3 | cav3 | 7955.ENSDARP00000034317 | caveolin 3 ; May act as a scaffolding protein within caveolar membranes. Interacts directly with G-protein alpha subunits and can functionally regulate their activity (By similarity) |
| cbx1a | cbx1a | 7955.ENSDARP00000027355 | chromobox homolog 1 |
| ccnb2 | ccnb2 | 7955.ENSDARP00000052540 | cyclin B2 |
| chrna5 | chrna5 | 7955.ENSDARP00000002331 | cholinergic receptor, nicotinic, alpha 5 |
| cisd2 | zgc:64148 | 7955.ENSDARP00000069092 | CDGSH iron sulfur domain 2 |
| cndp2 | cndp2 | 7955.ENSDARP00000033632 | CNDP dipeptidase 2 |
| cox11 | im:6904550 | 7955.ENSDARP00000097556 | COX11 homolog, cytochrome c oxidase assembly protein |
| cox7a2 | cox7a2 | 7955.ENSDARP00000069708 | cytochrome c oxidase, subunit VIIa 2 |
| cpox | cpox | 7955.ENSDARP00000083605 | coproporphyrinogen oxidase |
| crata | zgc:92317 | 7955.ENSDARP00000047376 | carnitine acetyltransferase |
| cst3 | cst3 | 7955.ENSDARP00000103585 | cystatin C |
| ctrb1 | ctrb1 | 7955.ENSDARP00000036127 | chymotrypsin B1 |
| cx43 | cx43 | 7955.ENSDARP00000061260 | Gap junction alpha-1 protein (Connexin-43)(Cx43)(Short fin protein); One gap junction consists of a cluster of closely packed pairs of transmembrane channels, the connexons, through which materials of low MW diffuse from one cell to a neighboring cell |
| cyt1 | cyt1 | 7955.ENSDARP00000095806 | type I cytokeratin, enveloping layer |
| daam1b | daam1l | 7955.ENSDARP00000025716 | si:ch211-87i20.1 |
| dcps | dcps | 7955.ENSDARP00000022189 | mRNA decapping enzyme |
| dlg1 | dlg1 | 7955.ENSDARP00000061428 | Disks large homolog 1 (Synapse-associated protein 97A)(SAP-97A); Essential multidomain scaffolding protein required for normal development. Recruits channels, receptors and signaling molecules to discrete plasma membrane domains in polarized cells. May play a role in adherens junction assembly, signal transduction and cell proliferation (By similarity). May play a role in synapse assembly and function |
| dlx6a | dlx6a | 7955.ENSDARP00000093553 | Homeobox protein Dlx6a (Distal-less homeobox protein 6a)(DLX-6) |
| dmap1 | dmap1 | 7955.ENSDARP00000061678 | DNA methyltransferase 1 associated protein 1 |
| dmbx1b | dmbx1b | 7955.ENSDARP00000027542 | Diencephalon/mesencephalon homeobox protein 1-B |
| dnajc24 | dnajc24 | 7955.ENSDARP00000037632 | DPH4 protein |
| dock4b | dock4b | 7955.ENSDARP00000036828 | Novel protein similar to vertebrate dedicator of cytokinesis 4 (DOCK4) Fragment |
| dph5 | dph5 | 7955.ENSDARP00000022829 | dph5-like ; Required for the methylation step in diphthamide biosynthesis (By similarity) |
| dpp6b | dpp6 | 7955.ENSDARP00000028871 | dipeptidyl-peptidase 6 |
| dtna | dtna | 7955.ENSDARP00000096457 | dystrobrevin, alpha |
| edil3b | edil3b | 7955.ENSDARP00000080662 | EGF-like repeats and discoidin I-like domains 3 |
| efnb2a | efnb2a | 7955.ENSDARP00000010432 | Ephrin-B2a Precursor |
| egfra | egfr | 7955.ENSDARP00000012063 | epidermal growth factor receptor |
| eif4a1b | eif4a1b | 7955.ENSDARP00000018923 | eukaryotic translation initiation factor 4A, isoform 1B |
| emp1 | emp1 | 7955.ENSDARP00000092251 | Novel protein with PMP-22/EMP/MP20/Claudin family domain |
| epb4.1l4 | epb4.1l4 | 7955.ENSDARP00000058626 | Band 4.1-like protein 4 (Protein Nbl4); Not known, binds calmodulin |
| fabp7b | fabp7b | 7955.ENSDARP00000041228 | fatty acid binding protein 7, brain, b |
| fads2 | fads2 | 7955.ENSDARP00000022396 | Fatty acid desaturase 2 (EC 1.14.19.-)(Delta(5)/Delta(6) fatty acid desaturase)(Delta-5/Delta-6 fatty acid desaturase)(D5D/D6D fatty acid desaturase); Fatty acid desaturase with both Delta(5) and Delta(6) activities. Component of a lipid metabolic pathway that catalyzes biosynthesis of highly unsaturated fatty acids (HUFA) from precursor essential polyunsaturated fatty acids (PUFA) linoleic acid (LA) (18:2n-6) and alpha-linolenic acid (ALA) (18:3n-3) (By similarity) |
| fam86a | fam86a | 7955.ENSDARP00000071691 | hypothetical protein LOC450038 |
| fancd2 | fancd2 | 7955.ENSDARP00000070083 | Fanconi anemia, complementation group D2 |
| fgg | fgg | 7955.ENSDARP00000054229 | fibrinogen gamma polypeptide |
| fkbp9 | fkbp9 | 7955.ENSDARP00000022147 | FK506 binding protein 9 |
| foxi3b | foxi3b | 7955.ENSDARP00000002195 | forkhead box I3b |
| foxn4 | foxn4 | 7955.ENSDARP00000020367 | forkhead box N4 |
| fstl4 | fstl4 | 7955.ENSDARP00000065082 | follistatin-like |
| FZD6 | FZD6 | 7955.ENSDARP00000062702 | frizzled homolog 6 (Drosophila) |
| gata6 | gata6 | 7955.ENSDARP00000051997 | GATA-binding protein 6 |
| git2a | git2a | 7955.ENSDARP00000062785 | similar to p95-APP2 |
| glcci1 | glcci1 | 7955.ENSDARP00000035581 | glucocorticoid induced transcript 1 |
| glt25d1 | glt25d1 | 7955.ENSDARP00000102260 | glycosyltransferase 25 domain containing 1 |
| gltpd1 | zgc:92000 | 7955.ENSDARP00000068896 | Glycolipid transfer protein domain-containing protein 1 |
| golph3 | golph3 | 7955.ENSDARP00000051069 | golgi phosphoprotein 3 |
| gpx4b | gpx4b | 7955.ENSDARP00000103087 | glutathione peroxidase 4b |
| gria3a | gria3a | 7955.ENSDARP00000049290 | glutamate receptor, ionotropic, AMPA 3a |
| hdac1 | hdac1 | 7955.ENSDARP00000051798 | histone deacetylase 1 |
| hic1l | hic1l | 7955.ENSDARP00000067137 | hypermethylated in cancer 1 like |
| hoxb10a | hoxb10a | 7955.ENSDARP00000016856 | Homeobox protein Hox-B10a Fragment (Hox-B10); Sequence-specific transcription factor which is part of a developmental regulatory system that provides cells with specific positional identities on the anterior-posterior axis (By similarity) |
| hspa5 | hspa5 | 7955.ENSDARP00000017456 | heat shock 70kDa protein 5 |
| hspb3 | hspb3 | 7955.ENSDARP00000088297 | heat shock protein, alpha-crystallin-related, b3 |
| id1 | id1 | 7955.ENSDARP00000059731 | inhibitor of DNA binding 1 |
| ido1 | ido1 | 7955.ENSDARP00000089488 | indoleamine 2,3-dioxygenase 1 |
| ier2 | wu:fb01b03 | 7955.ENSDARP00000098882 | immediate early response 2 |
| il1b | il1b | 7955.ENSDARP00000002293 | interleukin 1, beta |
| illr3 | illr3 | 7955.ENSDARP00000095925 | immune-related, lectin-like receptor 3 isoform b |
| ism2 | ism2 | 7955.ENSDARP00000070453 | Isthmin-2 Precursor |
| jak2b | jak2b | 7955.ENSDARP00000028132 | Janus kinase 2 |
| josd2 | josd2 | 7955.ENSDARP00000074792 | Josephin domain containing 2 |
| katnb1 | katnb1 | 7955.ENSDARP00000024623 | Katanin p80 WD40-containing subunit B1 (Katanin p80 subunit B1)(p80 katanin); Participates in a complex which severs microtubules in an ATP-dependent manner. May act to target the enzymatic subunit of this complex to sites of action such as the centrosome. Microtubule severing may promote rapid reorganization of cellular microtubule arrays and the release of microtubules from the centrosome following nucleation (By similarity) |
| kcnip3 | kcnip3 | 7955.ENSDARP00000064580 | Kv channel interacting protein 3, calsenilin |
| kctd9 | kctd9 | 7955.ENSDARP00000027995 | potassium channel tetramerisation domain containing 9 |
| larp7 | zgc:56476 | 7955.ENSDARP00000013520 | La-related protein 7 (La ribonucleoprotein domain family member 7) |
| lmo4 | lmo4 | 7955.ENSDARP00000079654 | LIM domain only 4 |
| lrrc42 | lrrc42 | 7955.ENSDARP00000045055 | Leucine-rich repeat-containing protein 42 |
| mab21l2 | mab21l2 | 7955.ENSDARP00000019528 | Protein mab-21-like 2 ; Required for eye morphogenesis. May promote the survival of proliferating retinal progenitor cells |
| mafg2 | mafg2 | 7955.ENSDARP00000046178 | v-maf musculoaponeurotic fibrosarcoma oncogene homolog g (avian), 2 |
| med21 | med21 | 7955.ENSDARP00000036773 | Mediator of RNA polymerase II transcription subunit 21 (Mediator complex subunit 21)(RNA polymerase II holoenzyme component SRB7)(RNAPII complex component SRB7); Component of the Mediator complex, a coactivator involved in the regulated transcription of nearly all RNA polymerase II-dependent genes. Mediator functions as a bridge to convey information from gene-specific regulatory proteins to the basal RNA polymerase II transcription machinery. Mediator is recruited to promoters by direct interactions with regulatory proteins and serves as a scaffold for the assembly of a functional pre [...] |
| mibp | mibp | 7955.ENSDARP00000099308 | muscle-specific beta 1 integrin binding protein |
| mif | mif | 7955.ENSDARP00000096200 | macrophage migration inhibitory factor |
| mlx | mlx | 7955.ENSDARP00000055226 | transcription factor-like 4 |
| mmp24 | mmp24 | 7955.ENSDARP00000047398 | Novel protein similar to vertebrate membrane-inserted matrix metalloproteinase 24 (MMP24) Fragment |
| mpped2 | mpped2 | 7955.ENSDARP00000050477 | metallophosphoesterase domain containing 2 |
| mrc1b | mrc1b | 7955.ENSDARP00000056685 | Novel protein similar to human and mouse mannose receptor, C type 1 (MRC1) Fragment |
| mrpl35 | mrpl35 | 7955.ENSDARP00000094603 | mitochondrial ribosomal protein L35 |
| mrpl39 | mrpl39 | 7955.ENSDARP00000082504 | mitochondrial ribosomal protein L39 |
| ms4a17a.14 | ms4a17a.14 | 7955.ENSDARP00000090682 | Novel protein |
| msxc | msxc | 7955.ENSDARP00000024644 | Homeobox protein MSH-C ; Involved in the development of the inner ear |
| mynn | mynn | 7955.ENSDARP00000016471 | Myoneurin |
| myo5b | myo5b | 7955.ENSDARP00000083463 | myosin VB |
| nkx2.3 | nkx2.3 | 7955.ENSDARP00000057093 | NK2 transcription factor related 3 |
| npy8ar | npy8ar | 7955.ENSDARP00000098548 | neuropeptide Y receptor Y8a |
| nr1d2a | nr1d2a | 7955.ENSDARP00000089813 | nuclear receptor subfamily 1, group D, member 2a |
| nsun2 | nsun2 | 7955.ENSDARP00000094668 | NOL1/NOP2/Sun domain family, member 2 |
| ntf7 | ntf7 | 7955.ENSDARP00000096012 | Neurotrophin-7 Precursor (NT-7)(zNT-7); Recombinant ZNT-7 was able to bind to the human p75 neurotrophin receptor and to induce tyrosine phosphorylation of the rat TRKA receptor tyrosine kinase, albeit less efficiently than rat NGF. ZNT-7 did not interact with rat TRKB or TRKC, indicating a similar receptor specificity as NGF |
| ntn4 | NTN4 | 7955.ENSDARP00000101085 | netrin 4 |
| nubp2 | nubp2 | 7955.ENSDARP00000072510 | nucleotide binding protein 2 (MinD homolog, E. coli) ; Component of the cytosolic iron-sulfur (Fe/S) protein assembly machinery. Required for maturation of extramitochondrial Fe/S proteins. May bind and transfer a labile 4Fe-4S cluster to target apoproteins (By similarity) |
| oep | oep | 7955.ENSDARP00000091797 | one-eyed pinhead |
| ogn | ogn | 7955.ENSDARP00000064800 | osteoglycin |
| opn1mw1 | opn1mw1 | 7955.ENSDARP00000001158 | Green-sensitive opsin-1 (Green cone photoreceptor pigment 1)(Opsin-1, medium-wave-sensitive 1)(Opsin RH2-1); Visual pigments are the light-absorbing molecules that mediate vision. They consist of an apoprotein, opsin, covalently linked to cis-retinal |
| or115-13 | or115-13 | 7955.ENSDARP00000023558 | odorant receptor, family F, subfamily 115, member 13 |
| or124-1 | or124-1 | 7955.ENSDARP00000092469 | odorant receptor, family E, subfamily 124, member 1 |
| ormdl3 | zgc:101654 | 7955.ENSDARP00000032009 | ORM1-like protein 3 |
| pah | pah | 7955.ENSDARP00000012808 | phenylalanine hydroxylase |
| pcdh2g9 | pcdh2g9 | 7955.ENSDARP00000099581 | protocadherin 2 gamma 9 |
| pcyt2 | zgc:103434 | 7955.ENSDARP00000063134 | phosphate cytidylyltransferase 2, ethanolamine |
| pdlim3a | pdlim3a | 7955.ENSDARP00000006482 | PDZ and LIM domain protein 3 |
| pelo | pelo | 7955.ENSDARP00000072295 | Protein pelota homolog (EC 3.1.-.-); Required for normal chromosome segregation during cell division and genomic stability (By similarity). May function in recognizing stalled ribosomes and triggering endonucleolytic cleavage of the mRNA, a mechanism to release non-functional ribosomes and degrade damaged mRNAs. May have ribonuclease activity (Potential) |
| pik3c3 | pik3c3 | 7955.ENSDARP00000092039 | catalytic phosphatidylinositol 3-kinase 3 |
| pinx1 | pinx1 | 7955.ENSDARP00000031264 | pin2/trf1-interacting protein 1 |
| pla2g4a | pla2g4a | 7955.ENSDARP00000024335 | Novel protein similar to cytosolic phospholipase a2 (Cpla2) Fragment |
| plcd3b | plcd3b | 7955.ENSDARP00000074246 | Novel protein similar to H.sapiens phospholipase C, delta Fragment |
| plod2 | plod2 | 7955.ENSDARP00000012147 | procollagen-lysine, 2-oxoglutarate 5-dioxygenase 2 isoform 1 |
| pltp | zgc:100903 | 7955.ENSDARP00000051900 | phospholipid transfer protein |
| poln | poln | 7955.ENSDARP00000099211 | hypothetical protein LOC566923 |
| polr2d | polr2d | 7955.ENSDARP00000099022 | polymerase (RNA) II (DNA directed) polypeptide D |
| ppie | ppie | 7955.ENSDARP00000076776 | peptidylprolyl isomerase E |
| ppm1e | ppm1e | 7955.ENSDARP00000063550 | Ca/calmodulin-dependent protein kinase phosphatase-N |
| prkacbb | prkacb | 7955.ENSDARP00000076580 | protein kinase, cAMP-dependent, catalytic, beta |
| psme2 | psme2 | 7955.ENSDARP00000049166 | proteasome activator subunit 2 |
| ptgr1 | ptgr1 | 7955.ENSDARP00000038835 | prostaglandin reductase 1 |
| ptrh1 | ptrh1 | 7955.ENSDARP00000050195 | hypothetical protein LOC559192 |
| RALGAPA1 | RALGAPA1 | 7955.ENSDARP00000101097 | Ral GTPase activating protein, alpha subunit 1 (catalytic) |
| rasgef1ba | rasgef1ba | 7955.ENSDARP00000088652 | Ras-GEF domain-containing family member 1B-A ; Guanine nucleotide exchange factor (GEF) for Ras family proteins (in vitro) (By similarity) |
| rdh1 | rdh1 | 7955.ENSDARP00000003828 | retinol dehydrogenase 1 |
| rgs8 | zgc:92913 | 7955.ENSDARP00000093238 | Regulator of G-protein signaling 8 (RGS8); May inhibit signal transduction by increasing the GTPase activity of G protein alpha subunits thereby driving them into their inactive GDP-bound form (By similarity) |
| rhbg | rhbg | 7955.ENSDARP00000058869 | Ammonium transporter Rh type B (Rhesus blood group family type B glycoprotein)(Rh family type B glycoprotein)(Rh type B glycoprotein); Functions as an ammonia transporter. May play a role in the elimination of ammonia in the gill (By similarity) |
| rhobtb2a | rhobtb2a | 7955.ENSDARP00000051113 | Rho-related BTB domain containing 2a |
| rln3a | rln3 | 7955.ENSDARP00000082236 | relaxin 3 |
| rmi1 | rmi1 | 7955.ENSDARP00000060656 | RecQ-mediated genome instability protein 1 ; Essential component of the RMI complex, a complex that plays an important role in the processing of homologous recombination intermediates to limit DNA crossover formation in cells. Promotes TOP3A binding to double Holliday junctions (DHJ) and hence stimulates TOP3A-mediated dissolution. Required for BLM phosphorylation during mitosis. Within the BLM complex, required for BLM and TOP3A stability (By similarity) |
| rnaseh2a | rnaseh2a | 7955.ENSDARP00000045893 | ribonuclease H2, large subunit ; Endonuclease that specifically degrades the RNA of RNA- DNA hybrids (By similarity) |
| rnaseka | rnaseka | 7955.ENSDARP00000091898 | Ribonuclease kappa-A (RNase kappa-A)(RNase K-A)(EC 3.1.-.-); Endoribonuclease which preferentially cleaves ApU and ApG phosphodiester bonds (By similarity) |
| rorca | rorca | 7955.ENSDARP00000091257 | RAR-related orphan receptor C a |
| rpap2 | rpap2 | 7955.ENSDARP00000056033 | Novel protein |
| rps28 | rps28 | 7955.ENSDARP00000052062 | 40S ribosomal protein S28 |
| runx2a | runx2a | 7955.ENSDARP00000096167 | runt-related transcription factor 2a |
| rxrbb | rxrbb | 7955.ENSDARP00000022973 | Retinoic acid receptor RXR-beta-B (Retinoid X receptor beta-B)(Retinoic acid receptor RXR-delta)(Retinoid X receptor delta)(Nuclear receptor subfamily 2 group B member 2-B); Nuclear hormone receptor. Involved in the retinoic acid response pathway. Does not bind 9-cis retinoic acid (9C-RA). Transcriptionally inactive on RXR response elements. May function to negatively modulate thyroid hormone-dependent transcription |
| sap30l | sap30l | 7955.ENSDARP00000028875 | Histone deacetylase complex subunit SAP30L (Sin3A-associated protein p30-like protein)(Sin3 corepressor complex subunit SAP30L); Required for the function of the class 1 Sin3-histone deacetylase complex (HDAC) (By similarity) |
| sepw1 | sepw1 | 7955.ENSDARP00000050883 | Selenoprotein W (SelW); May be involved in a redox-related process. May play a role in the myopathies of selenium deficiency (By similarity) |
| sfmbt2 | sfmbt2 | 7955.ENSDARP00000044376 | Novel protein similar to vertebrate Scm-like with four mbt domains 1 (SFMBT1) Fragment |
| si:ch211-212d10.1 | ENSDARG00000014623 | 7955.ENSDARP00000096188 | Si:ch211-212d10.1 protein Fragment |
| six4.3 | six4.3 | 7955.ENSDARP00000089692 | sine oculis homeobox homolog 4.3 |
| slc16a3 | slc16a3 | 7955.ENSDARP00000066232 | monocarboxylate transporter 4 |
| slc25a36b | slc25a36b | 7955.ENSDARP00000045613 | solute carrier family 25, member 36b |
| slc43a1b | slc43a1b | 7955.ENSDARP00000076274 | solute carrier family 43, member 1b |
| slc7a9 | slc7a9 | 7955.ENSDARP00000091252 | solute carrier family 7 (cationic amino acid transporter, y+ system), member 9 |
| slco1c1 | slco1c1 | 7955.ENSDARP00000039385 | solute carrier organic anion transporter family, member 1C1 |
| slco2a1 | slco2a1 | 7955.ENSDARP00000083168 | solute carrier organic anion transporter family, member 2A1 |
| smad2 | smad2 | 7955.ENSDARP00000044755 | Mothers against decapentaplegic homolog 2 (Mothers against DPP homolog 2)(SMAD 2)(Smad2); Promotes differentiation of dorsal tissues. May be involved in the mediation of Ndr2 signaling during mesoderm and axis formation during embryogenesis |
| smad5 | smad5 | 7955.ENSDARP00000054174 | Mothers against decapentaplegic homolog 5 (Mothers against DPP homolog 5)(SMAD 5)(Smad5)(Protein somitabun); Involved in ventralization. May mediate Bmp2b signaling during early phases of embryonic dorsal-ventral pattern formation. Required for initation of Smad1 expression during gastrulation |
| snrpd1 | snrpd1 | 7955.ENSDARP00000027770 | small nuclear ribonucleoprotein D1 polypeptide |
| snx30 | snx30 | 7955.ENSDARP00000050176 | Sorting nexin-30 ; May be involved in several stages of intracellular trafficking (By similarity) |
| sox19a | sox19a | 7955.ENSDARP00000019070 | Transcription factor Sox-19a ; Transcriptional activator |
| sparcl1 | sparcl | 7955.ENSDARP00000078028 | secreted acidic cysteine rich glycoprotein-like |
| spred1 | spred1 | 7955.ENSDARP00000060763 | sprouty-related protein 1 with EVH-1 domain |
| spsb4b | spsb4b | 7955.ENSDARP00000059326 | splA/ryanodine receptor domain and SOCS box containing 4b |
| srp19 | srp19 | 7955.ENSDARP00000051775 | signal recognition particle 19 |
| stmn2b | stmn2b | 7955.ENSDARP00000072761 | stathmin-like 2b |
| stom | stom | 7955.ENSDARP00000074954 | stomatin |
| sult1st6 | sult1st6 | 7955.ENSDARP00000023637 | sulfotransferase family, cytosolic, 1C, member 3 |
| sult3st2 | sult3st2 | 7955.ENSDARP00000096517 | sulfotransferase family 3, cytosolic sulfotransferase 2 |
| taf7 | taf7 | 7955.ENSDARP00000068986 | TAF7 RNA polymerase II, TATA box binding protein (TBP)-associated factor |
| tbx5a | tbx5 | 7955.ENSDARP00000033053 | T-box transcription factor TBX5 (T-box protein 5) |
| tceb1b | zgc:92635 | 7955.ENSDARP00000066703 | transcription elongation factor B (SIII), polypeptide 1 |
| tcf7l1a | tcf7l1a | 7955.ENSDARP00000090035 | Transcription factor 7-like 1-A (HMG box transcription factor 3-A)(TCF-3-A)(TCF-3)(zTcf-3)(Protein headless); Participates in the Wnt signaling pathway. Probably binds to DNA and acts as a repressor in the absence of ctnnb1, and possibly as an activator in its presence. Required early in development for dorsal specific gene activation and during gastrulation for repression of dorsal-specific genes in the marginal zone. Required for head formation and forebrain specification. Regulates anterior-posterior patterning in the neuroectoderm by repressing posterior neural fates |
| tfcp2l1 | tfcp2l1 | 7955.ENSDARP00000040479 | transcription factor CP2-like 1 |
| tmem179 | zgc:101058 | 7955.ENSDARP00000022822 | Transmembrane protein 179 |
| tmub2 | tmub2 | 7955.ENSDARP00000073431 | transmembrane and ubiquitin-like domain containing 2 |
| tpma | tpma | 7955.ENSDARP00000039656 | Tropomyosin alpha-1 chain (Tropomyosin-1)(Alpha-tropomyosin); Binds to actin filaments in muscle and non-muscle cells. Plays a central role, in association with the troponin complex, in the calcium dependent regulation of vertebrate striated muscle contraction. Smooth muscle contraction is regulated by interaction with caldesmon. In non-muscle cells is implicated in stabilizing cytoskeleton actin filaments |
| tpst1l | tpst1l | 7955.ENSDARP00000065364 | tyrosylprotein sulfotransferase 1, like |
| trim13 | trim13 | 7955.ENSDARP00000013495 | tripartite motif-containing 13 |
| trim54 | trim54 | 7955.ENSDARP00000049429 | ring finger protein 30 |
| trio | ENSDARG00000019426 | 7955.ENSDARP00000081489 | Triple functional domain protein (EC 2.7.11.1); Promotes the exchange of GDP by GTP. Together with leukocyte antigen-related (LAR) protein, it could play a role in coordinating cell-matrix and cytoskeletal rearrangements necessary for cell migration and cell growth (By similarity) |
| ttc36 | zgc:103600 | 7955.ENSDARP00000017354 | Tetratricopeptide repeat protein 36 (TPR repeat protein 36) |
| ttc9c | zgc:56497 | 7955.ENSDARP00000016595 | tetratricopeptide repeat domain 9C |
| ufc1 | ufc1 | 7955.ENSDARP00000036899 | ubiquitin-fold modifier conjugating enzyme 1 |
| ugt5d1 | LOC799305 | 7955.ENSDARP00000091175 | UDP glucuronosyltransferase 5 family, polypeptide D1 |
| unc5da | unc5da | 7955.ENSDARP00000085424 | Unc5Da Fragment |
| usp30 | usp30 | 7955.ENSDARP00000073858 | Ubiquitin carboxyl-terminal hydrolase 30 (EC 3.1.2.15)(Ubiquitin thioesterase 30)(Ubiquitin-specific protease 30)(Ub-specific protease 30)(Deubiquitinating enzyme 30); May participate in the maintenance of mitochondrial morphology (By similarity) |
| vent | vent | 7955.ENSDARP00000011087 | ventral expressed homeobox |
| vsx1 | vsx1 | 7955.ENSDARP00000073224 | Visual system homeobox 1 (Transcription factor VSX1); May be involved in maintenance as well as cellular differentiation of retinal interneurons, such as bipolar cells. May play a role in establishing interneuronal cell classes in nonsensory as well as sensory systems |
| xkrx | xkrx | 7955.ENSDARP00000064613 | XK, Kell blood group complex subunit-related, X-linked |
| yeats4 | yeats4 | 7955.ENSDARP00000067426 | YEATS domain containing 4 |
| zgc:123060 | LOC555422 | 7955.ENSDARP00000096332 | zgc:123060 (zgc:123060), mRNA |
| zgc:158225 | zgc:158225 | 7955.ENSDARP00000091715 | zgc:158225 |
| zgc:171573 | zgc:171573 | 7955.ENSDARP00000103213 | Zgc:171573 protein |
| zgc:174354 | ENSDARG00000044984 | 7955.ENSDARP00000066126 | Zgc:174354 protein |
| zgc:174690 | ENSDARG00000074557 | 7955.ENSDARP00000098694 | zgc:174690 (zgc:174690), mRNA |
| znf395 | znf395 | 7955.ENSDARP00000007418 | zinc finger protein 395 |
