# Supplementary material for: Functional Categorization of Transcriptome in the Species Symphysodon aequifasciatus Pellegrin 1904 (Perciformes: Cichlidae) Exposed to Benzo[a]pyrene and Phenanthrene
Source: PLoS One. 2013 Dec 3;8(12):e81083. doi: 10.1371/journal.pone.0081083 (PMC3849039; doi:10.1371/journal.pone.0081083)
Supplement: Table S4 — Proteins interactions in Symphysodon aequifasciatus exposed to benzo[a]pyrene and phenanthrene for 48 h using the STRING software (v.9.1). (DOC) [file pone.0081083.s009.doc]

Table S4. Proteins interactions in *Symphysodon aequifasciatus* exposed to benzo[a]pyrene and phenanthrene for 48h using the STRING software (v.9.1).

| node1 | node2 | node1_string_id | node2_string_id | node1_external_id | node2_external_id | neighborhood | fusion | cooccurence | homology | coexpression | experimental | knowledge | textmining | combined score |
| --- | --- | --- | --- | --- | --- | --- | --- | --- | --- | --- | --- | --- | --- | --- |
| oep | smad2 | 818929 | 808131 | ENSDARP00000091797 | ENSDARP00000044755 | 0 | 0 | 0 | 0 | 0 | 0 | 0,9 | 0,759 | 0,974 |
| cox7a2 | atp5d | 813235 | 805466 | ENSDARP00000069708 | ENSDARP00000022528 | 0 | 0 | 0 | 0 | 0,962 | 0 | 0 | 0,08 | 0,963 |
| polr2d | snrpd1 | 822340 | 806216 | ENSDARP00000099022 | ENSDARP00000027770 | 0 | 0 | 0 | 0 | 0,318 | 0 | 0,9 | 0 | 0,927 |
| oep | gata6 | 818929 | 809096 | ENSDARP00000091797 | ENSDARP00000051997 | 0 | 0 | 0 | 0 | 0 | 0 | 0 | 0,914 | 0,914 |
| hdac1 | smad2 | 809066 | 808131 | ENSDARP00000051798 | ENSDARP00000044755 | 0 | 0 | 0 | 0 | 0 | 0,077 | 0,9 | 0,125 | 0,908 |
| psme2 | abcb3l1 | 808678 | 806339 | ENSDARP00000049166 | ENSDARP00000028924 | 0 | 0 | 0 | 0 | 0,134 | 0 | 0,9 | 0 | 0,907 |
| nr1d2a | rxrbb | 818180 | 805542 | ENSDARP00000089813 | ENSDARP00000022973 | 0 | 0 | 0 | 0,729 | 0,116 | 0 | 0,9 | 0 | 0,905 |
| polr2d | ak3 | 822340 | 814910 | ENSDARP00000099022 | ENSDARP00000075506 | 0 | 0 | 0 | 0 | 0 | 0 | 0,9 | 0 | 0,899 |
| pla2g4a | fads2 | 805741 | 805447 | ENSDARP00000024335 | ENSDARP00000022396 | 0 | 0 | 0 | 0 | 0 | 0 | 0,9 | 0 | 0,899 |
| gata6 | smad2 | 809096 | 808131 | ENSDARP00000051997 | ENSDARP00000044755 | 0 | 0 | 0 | 0 | 0 | 0 | 0 | 0,878 | 0,878 |
| poln | med21 | 822494 | 807146 | ENSDARP00000099211 | ENSDARP00000036773 | 0 | 0 | 0 | 0 | 0 | 0 | 0 | 0,878 | 0,877 |
| yeats4 | dmap1 | 812589 | 811199 | ENSDARP00000067426 | ENSDARP00000061678 | 0 | 0 | 0 | 0 | 0,134 | 0,646 | 0 | 0,576 | 0,852 |
| prkacb | cx43 | 815283 | 811110 | ENSDARP00000076580 | ENSDARP00000061260 | 0 | 0 | 0 | 0 | 0 | 0 | 0,8 | 0 | 0,8 |
| gata6 | tbx5 | 809096 | 806747 | ENSDARP00000051997 | ENSDARP00000033053 | 0 | 0 | 0 | 0 | 0 | 0 | 0 | 0,71 | 0,71 |
| ufc1 | hspa5 | 807157 | 804785 | ENSDARP00000036899 | ENSDARP00000017456 | 0 | 0 | 0 | 0 | 0,169 | 0 | 0 | 0,6 | 0,645 |
| vsx1 | foxn4 | 814216 | 805192 | ENSDARP00000073224 | ENSDARP00000020367 | 0 | 0 | 0 | 0 | 0,143 | 0 | 0 | 0,6 | 0,634 |
| tmub2 | ufc1 | 814279 | 807157 | ENSDARP00000073431 | ENSDARP00000036899 | 0 | 0 | 0 | 0 | 0 | 0 | 0 | 0,601 | 0,601 |
| tmub2 | fancd2 | 814279 | 813338 | ENSDARP00000073431 | ENSDARP00000070083 | 0 | 0 | 0 | 0 | 0 | 0 | 0 | 0,596 | 0,595 |
| fgg | pah | 809584 | 804139 | ENSDARP00000054229 | ENSDARP00000012808 | 0 | 0 | 0 | 0 | 0,52 | 0 | 0 | 0,079 | 0,528 |
| cst3 | ak3 | 825993 | 814910 | ENSDARP00000103585 | ENSDARP00000075506 | 0 | 0 | 0 | 0 | 0,495 | 0 | 0 | 0 | 0,495 |
| snrpd1 | dph5 | 806216 | 805519 | ENSDARP00000027770 | ENSDARP00000022829 | 0 | 0 | 0 | 0 | 0,478 | 0 | 0 | 0 | 0,478 |
| ak3 | gata6 | 814910 | 809096 | ENSDARP00000075506 | ENSDARP00000051997 | 0 | 0 | 0 | 0 | 0,478 | 0 | 0 | 0 | 0,477 |
| pelo | rps28 | 813898 | 809108 | ENSDARP00000072295 | ENSDARP00000052062 | 0 | 0 | 0 | 0 | 0 | 0,47 | 0 | 0 | 0,47 |
| hdac1 | sap30l | 809066 | 806334 | ENSDARP00000051798 | ENSDARP00000028875 | 0 | 0 | 0 | 0 | 0 | 0,399 | 0,066 | 0,157 | 0,461 |
| tbx5 | efnb2a | 806747 | 803776 | ENSDARP00000033053 | ENSDARP00000010432 | 0 | 0 | 0 | 0 | 0 | 0 | 0 | 0,433 | 0,433 |
| rnaseh2a | zgc:63700 | 808279 | 807261 | ENSDARP00000045893 | ENSDARP00000037835 | 0 | 0 | 0 | 0 | 0 | 0 | 0 | 0,433 | 0,433 |
| sap30l | rxrbb | 806334 | 805542 | ENSDARP00000028875 | ENSDARP00000022973 | 0 | 0 | 0 | 0 | 0 | 0 | 0 | 0,431 | 0,43 |
| smad5 | vent | 809568 | 803869 | ENSDARP00000054174 | ENSDARP00000011087 | 0 | 0 | 0 | 0 | 0 | 0 | 0 | 0,43 | 0,43 |
| tbx5 | foxn4 | 806747 | 805192 | ENSDARP00000033053 | ENSDARP00000020367 | 0 | 0 | 0 | 0 | 0 | 0 | 0 | 0,43 | 0,43 |
| tbx5 | msxc | 806747 | 805788 | ENSDARP00000033053 | ENSDARP00000024644 | 0 | 0 | 0 | 0 | 0 | 0 | 0 | 0,43 | 0,43 |
| tcf7l1a | smad2 | 818257 | 808131 | ENSDARP00000090035 | ENSDARP00000044755 | 0 | 0 | 0 | 0 | 0 | 0 | 0,36 | 0,165 | 0,429 |
| rnaseh2a | snrpd1 | 808279 | 806216 | ENSDARP00000045893 | ENSDARP00000027770 | 0 | 0 | 0 | 0 | 0,403 | 0 | 0 | 0 | 0,403 |
| fads2 | rdh1 | 805447 | 802867 | ENSDARP00000022396 | ENSDARP00000003828 | 0 | 0 | 0 | 0 | 0,399 | 0 | 0 | 0 | 0,399 |
| nkx2.3 | gata6 | 810193 | 809096 | ENSDARP00000057093 | ENSDARP00000051997 | 0 | 0 | 0 | 0 | 0 | 0 | 0 | 0,398 | 0,398 |
| oep | smad5 | 818929 | 809568 | ENSDARP00000091797 | ENSDARP00000054174 | 0 | 0 | 0 | 0 | 0 | 0 | 0 | 0,397 | 0,396 |
| id1 | smad2 | 810779 | 808131 | ENSDARP00000059731 | ENSDARP00000044755 | 0 | 0 | 0 | 0 | 0 | 0 | 0 | 0,397 | 0,396 |
| id1 | smad5 | 810779 | 809568 | ENSDARP00000059731 | ENSDARP00000054174 | 0 | 0 | 0 | 0 | 0 | 0 | 0 | 0,397 | 0,396 |
| smad5 | smad2 | 809568 | 808131 | ENSDARP00000054174 | ENSDARP00000044755 | 0 | 0 | 0 | 0,944 | 0 | 0 | 0,36 | 0,819 | 0,388 |
| yeats4 | snrpd1 | 812589 | 806216 | ENSDARP00000067426 | ENSDARP00000027770 | 0 | 0 | 0 | 0 | 0,388 | 0 | 0 | 0 | 0,388 |
| ctrb1 | rdh1 | 807080 | 802867 | ENSDARP00000036127 | ENSDARP00000003828 | 0 | 0 | 0 | 0 | 0,371 | 0 | 0 | 0 | 0,371 |
| rnaseh2a | dcps | 808279 | 805415 | ENSDARP00000045893 | ENSDARP00000022189 | 0 | 0 | 0 | 0 | 0,364 | 0 | 0 | 0,071 | 0,37 |
| tbx5 | rxrbb | 806747 | 805542 | ENSDARP00000033053 | ENSDARP00000022973 | 0 | 0 | 0 | 0 | 0 | 0 | 0 | 0,364 | 0,363 |
| tcf7l1a | smad5 | 818257 | 809568 | ENSDARP00000090035 | ENSDARP00000054174 | 0 | 0 | 0 | 0 | 0 | 0 | 0,36 | 0 | 0,36 |
| epb4.1l4 | psme2 | 810525 | 808678 | ENSDARP00000058626 | ENSDARP00000049166 | 0 | 0 | 0 | 0 | 0 | 0 | 0 | 0,357 | 0,357 |
| oep | mab21l2 | 818929 | 805084 | ENSDARP00000091797 | ENSDARP00000019528 | 0 | 0 | 0 | 0 | 0,339 | 0 | 0 | 0,086 | 0,355 |
| rps28 | snrpd1 | 809108 | 806216 | ENSDARP00000052062 | ENSDARP00000027770 | 0 | 0 | 0 | 0 | 0,355 | 0 | 0 | 0 | 0,355 |
| srp19 | ufc1 | 809061 | 807157 | ENSDARP00000051775 | ENSDARP00000036899 | 0 | 0 | 0 | 0 | 0,352 | 0 | 0 | 0 | 0,352 |
| ido1 | id1 | 818079 | 810779 | ENSDARP00000089488 | ENSDARP00000059731 | 0 | 0 | 0 | 0 | 0 | 0 | 0 | 0,35 | 0,35 |
| zgc:64148 | zgc:56476 | 813075 | 804242 | ENSDARP00000069092 | ENSDARP00000013520 | 0 | 0 | 0 | 0 | 0,35 | 0 | 0 | 0 | 0,35 |
| zgc:103600 | rdh1 | 804773 | 802867 | ENSDARP00000017354 | ENSDARP00000003828 | 0 | 0 | 0 | 0 | 0,349 | 0 | 0 | 0 | 0,349 |
| mab21l2 | zgc:103600 | 805084 | 804773 | ENSDARP00000019528 | ENSDARP00000017354 | 0 | 0 | 0 | 0 | 0,346 | 0 | 0 | 0 | 0,346 |
| sult1st6 | rdh1 | 805633 | 802867 | ENSDARP00000023637 | ENSDARP00000003828 | 0 | 0 | 0 | 0 | 0,346 | 0 | 0 | 0 | 0,346 |
| zgc:103600 | pah | 804773 | 804139 | ENSDARP00000017354 | ENSDARP00000012808 | 0 | 0 | 0 | 0 | 0,346 | 0 | 0 | 0 | 0,346 |
| srp19 | snrpd1 | 809061 | 806216 | ENSDARP00000051775 | ENSDARP00000027770 | 0 | 0 | 0 | 0 | 0,345 | 0 | 0 | 0 | 0,345 |
| sult3st2 | zgc:103600 | 820753 | 804773 | ENSDARP00000096517 | ENSDARP00000017354 | 0 | 0 | 0 | 0 | 0,345 | 0 | 0 | 0 | 0,345 |
| sult1st6 | bin2a | 805633 | 805066 | ENSDARP00000023637 | ENSDARP00000019400 | 0 | 0 | 0 | 0 | 0,343 | 0 | 0 | 0 | 0,343 |
| vsx1 | mab21l2 | 814216 | 805084 | ENSDARP00000073224 | ENSDARP00000019528 | 0 | 0 | 0 | 0 | 0,342 | 0 | 0 | 0 | 0,341 |
| fgg | zgc:103600 | 809584 | 804773 | ENSDARP00000054229 | ENSDARP00000017354 | 0 | 0 | 0 | 0 | 0,341 | 0 | 0 | 0 | 0,34 |
| stmn2b | trim13 | 814062 | 804237 | ENSDARP00000072761 | ENSDARP00000013495 | 0 | 0 | 0 | 0 | 0 | 0 | 0 | 0,34 | 0,34 |
| slc16a3 | ccnb2 | 812274 | 809228 | ENSDARP00000066232 | ENSDARP00000052540 | 0 | 0 | 0 | 0 | 0,34 | 0 | 0 | 0 | 0,34 |
| sult1st6 | zgc:103600 | 805633 | 804773 | ENSDARP00000023637 | ENSDARP00000017354 | 0 | 0 | 0 | 0 | 0,339 | 0 | 0 | 0 | 0,339 |
| atp1a1a.4 | bin2a | 808633 | 805066 | ENSDARP00000048827 | ENSDARP00000019400 | 0 | 0 | 0 | 0 | 0,339 | 0 | 0 | 0 | 0,339 |
| cyt1 | cav3 | 820493 | 806896 | ENSDARP00000095806 | ENSDARP00000034317 | 0 | 0 | 0 | 0 | 0,332 | 0 | 0 | 0 | 0,332 |
| id1 | sox19a | 810779 | 805023 | ENSDARP00000059731 | ENSDARP00000019070 | 0 | 0 | 0 | 0 | 0,332 | 0 | 0 | 0 | 0,332 |
| poln | fancd2 | 822494 | 813338 | ENSDARP00000099211 | ENSDARP00000070083 | 0 | 0 | 0 | 0 | 0 | 0,25 | 0 | 0,161 | 0,329 |
| ccnb2 | mynn | 809228 | 804651 | ENSDARP00000052540 | ENSDARP00000016471 | 0 | 0 | 0 | 0 | 0,327 | 0 | 0 | 0 | 0,327 |
| smad2 | vent | 808131 | 803869 | ENSDARP00000044755 | ENSDARP00000011087 | 0 | 0 | 0 | 0 | 0 | 0 | 0 | 0,323 | 0,323 |
| psme2 | il1b | 808678 | 802642 | ENSDARP00000049166 | ENSDARP00000002293 | 0 | 0 | 0 | 0 | 0 | 0 | 0 | 0,323 | 0,323 |
| cx43 | tbx5 | 811110 | 806747 | ENSDARP00000061260 | ENSDARP00000033053 | 0 | 0 | 0 | 0 | 0 | 0 | 0 | 0,321 | 0,321 |
| usp30 | foxi3b | 814423 | 802625 | ENSDARP00000073858 | ENSDARP00000002195 | 0 | 0 | 0 | 0 | 0 | 0 | 0 | 0,321 | 0,321 |
| ogn | fads2 | 811956 | 805447 | ENSDARP00000064800 | ENSDARP00000022396 | 0 | 0 | 0 | 0 | 0 | 0 | 0 | 0,321 | 0,321 |
| cav3 | abcb3l1 | 806896 | 806339 | ENSDARP00000034317 | ENSDARP00000028924 | 0 | 0 | 0 | 0 | 0 | 0 | 0 | 0,321 | 0,321 |
| dmap1 | hdac1 | 811199 | 809066 | ENSDARP00000061678 | ENSDARP00000051798 | 0 | 0 | 0 | 0 | 0,131 | 0,206 | 0 | 0,136 | 0,321 |
| ENSDARG00000019426 | vent | 816246 | 803869 | ENSDARP00000081489 | ENSDARP00000011087 | 0 | 0 | 0 | 0 | 0 | 0 | 0 | 0,32 | 0,319 |
| mlx | snrpd1 | 809780 | 806216 | ENSDARP00000055226 | ENSDARP00000027770 | 0 | 0 | 0 | 0 | 0 | 0 | 0 | 0,32 | 0,319 |
| med21 | snrpd1 | 807146 | 806216 | ENSDARP00000036773 | ENSDARP00000027770 | 0 | 0 | 0 | 0 | 0,313 | 0 | 0 | 0 | 0,313 |
| oep | sox19a | 818929 | 805023 | ENSDARP00000091797 | ENSDARP00000019070 | 0 | 0 | 0 | 0 | 0,253 | 0 | 0 | 0,138 | 0,312 |
| polr2d | rpap2 | 822340 | 809968 | ENSDARP00000099022 | ENSDARP00000056033 | 0 | 0 | 0 | 0 | 0 | 0,249 | 0 | 0,139 | 0,311 |
| ccnb2 | lrrc42 | 809228 | 808161 | ENSDARP00000052540 | ENSDARP00000045055 | 0 | 0 | 0 | 0 | 0,311 | 0 | 0 | 0 | 0,311 |
| tcf7l1a | vent | 818257 | 803869 | ENSDARP00000090035 | ENSDARP00000011087 | 0 | 0 | 0 | 0 | 0 | 0 | 0 | 0,307 | 0,307 |
| nkx2.3 | tbx5 | 810193 | 806747 | ENSDARP00000057093 | ENSDARP00000033053 | 0 | 0 | 0 | 0 | 0 | 0 | 0 | 0,306 | 0,306 |
| gpx4b | atp2b3a | 825605 | 816845 | ENSDARP00000103087 | ENSDARP00000084685 | 0 | 0 | 0 | 0 | 0 | 0 | 0 | 0,305 | 0,305 |
| ak3 | atp5d | 814910 | 805466 | ENSDARP00000075506 | ENSDARP00000022528 | 0 | 0 | 0 | 0 | 0 | 0 | 0,195 | 0,192 | 0,305 |
| sult3st2 | sult1st6 | 820753 | 805633 | ENSDARP00000096517 | ENSDARP00000023637 | 0 | 0 | 0,506 | 0,861 | 0,256 | 0 | 0 | 0 | 0,304 |
| gata6 | rdh1 | 809096 | 802867 | ENSDARP00000051997 | ENSDARP00000003828 | 0 | 0 | 0 | 0 | 0,181 | 0 | 0 | 0,203 | 0,303 |
| pah | rdh1 | 804139 | 802867 | ENSDARP00000012808 | ENSDARP00000003828 | 0 | 0 | 0 | 0 | 0,302 | 0 | 0 | 0 | 0,302 |
| fgg | rdh1 | 809584 | 802867 | ENSDARP00000054229 | ENSDARP00000003828 | 0 | 0 | 0 | 0 | 0,3 | 0 | 0 | 0 | 0,299 |
| rps28 | srp19 | 809108 | 809061 | ENSDARP00000052062 | ENSDARP00000051775 | 0 | 0 | 0 | 0 | 0,08 | 0 | 0,275 | 0 | 0,288 |
| rxrbb | efnb2a | 805542 | 803776 | ENSDARP00000022973 | ENSDARP00000010432 | 0 | 0 | 0 | 0 | 0,286 | 0 | 0 | 0 | 0,286 |
| nsun2 | rps28 | 820047 | 809108 | ENSDARP00000094668 | ENSDARP00000052062 | 0 | 0 | 0 | 0 | 0,168 | 0,193 | 0 | 0 | 0,284 |
| mrpl35 | mrpl39 | 820012 | 816434 | ENSDARP00000094603 | ENSDARP00000082504 | 0 | 0 | 0 | 0 | 0,283 | 0 | 0 | 0 | 0,283 |
| polr2d | zgc:92635 | 822340 | 812373 | ENSDARP00000099022 | ENSDARP00000066703 | 0 | 0 | 0 | 0 | 0,127 | 0 | 0,23 | 0 | 0,283 |
| cx43 | fkbp9 | 811110 | 805411 | ENSDARP00000061260 | ENSDARP00000022147 | 0 | 0 | 0 | 0 | 0 | 0 | 0 | 0,279 | 0,279 |
| fgg | il1b | 809584 | 802642 | ENSDARP00000054229 | ENSDARP00000002293 | 0 | 0 | 0 | 0 | 0 | 0 | 0 | 0,279 | 0,279 |
| runx2a | msxc | 820599 | 805788 | ENSDARP00000096167 | ENSDARP00000024644 | 0 | 0 | 0 | 0 | 0 | 0 | 0 | 0,276 | 0,276 |
| lrrc42 | mynn | 808161 | 804651 | ENSDARP00000045055 | ENSDARP00000016471 | 0 | 0 | 0 | 0 | 0,27 | 0 | 0 | 0 | 0,27 |
| gpx4b | atp5d | 825605 | 805466 | ENSDARP00000103087 | ENSDARP00000022528 | 0 | 0 | 0 | 0 | 0,266 | 0 | 0 | 0 | 0,266 |
| mab21l2 | pah | 805084 | 804139 | ENSDARP00000019528 | ENSDARP00000012808 | 0 | 0 | 0 | 0 | 0,265 | 0 | 0 | 0 | 0,265 |
| cav3 | sox19a | 806896 | 805023 | ENSDARP00000034317 | ENSDARP00000019070 | 0 | 0 | 0 | 0 | 0,265 | 0 | 0 | 0 | 0,265 |
| nsun2 | dcps | 820047 | 805415 | ENSDARP00000094668 | ENSDARP00000022189 | 0 | 0 | 0 | 0 | 0,264 | 0 | 0 | 0 | 0,265 |
| rln3 | pah | 816387 | 804139 | ENSDARP00000082236 | ENSDARP00000012808 | 0 | 0 | 0 | 0 | 0,265 | 0 | 0 | 0 | 0,265 |
| foxn4 | sox19a | 805192 | 805023 | ENSDARP00000020367 | ENSDARP00000019070 | 0 | 0 | 0 | 0 | 0,14 | 0 | 0 | 0,199 | 0,264 |
| rln3 | zgc:103600 | 816387 | 804773 | ENSDARP00000082236 | ENSDARP00000017354 | 0 | 0 | 0 | 0 | 0,263 | 0 | 0 | 0 | 0,263 |
| rln3 | mab21l2 | 816387 | 805084 | ENSDARP00000082236 | ENSDARP00000019528 | 0 | 0 | 0 | 0 | 0,261 | 0 | 0 | 0 | 0,26 |
| epb4.1l4 | mab21l2 | 810525 | 805084 | ENSDARP00000058626 | ENSDARP00000019528 | 0 | 0 | 0 | 0 | 0,26 | 0 | 0 | 0 | 0,26 |
| taf7 | hdac1 | 813037 | 809066 | ENSDARP00000068986 | ENSDARP00000051798 | 0 | 0 | 0 | 0 | 0,254 | 0 | 0 | 0,07 | 0,259 |
| cst3 | srp19 | 825993 | 809061 | ENSDARP00000103585 | ENSDARP00000051775 | 0 | 0 | 0 | 0 | 0,259 | 0 | 0 | 0 | 0,259 |
| smad2 | mynn | 808131 | 804651 | ENSDARP00000044755 | ENSDARP00000016471 | 0 | 0 | 0 | 0 | 0,257 | 0 | 0 | 0 | 0,257 |
| rln3 | cx43 | 816387 | 811110 | ENSDARP00000082236 | ENSDARP00000061260 | 0 | 0 | 0 | 0 | 0,257 | 0 | 0 | 0 | 0,257 |
| ccnb2 | rnaseh2a | 809228 | 808279 | ENSDARP00000052540 | ENSDARP00000045893 | 0 | 0 | 0 | 0 | 0,257 | 0 | 0 | 0 | 0,257 |
| cox7a2 | zgc:92635 | 813235 | 812373 | ENSDARP00000069708 | ENSDARP00000066703 | 0 | 0 | 0 | 0 | 0,257 | 0 | 0 | 0 | 0,257 |
| cx43 | mab21l2 | 811110 | 805084 | ENSDARP00000061260 | ENSDARP00000019528 | 0 | 0 | 0 | 0 | 0,256 | 0 | 0 | 0 | 0,256 |
| mrpl39 | srp19 | 816434 | 809061 | ENSDARP00000082504 | ENSDARP00000051775 | 0 | 0 | 0 | 0 | 0,256 | 0 | 0 | 0 | 0,256 |
| ccnb2 | zgc:56497 | 809228 | 804660 | ENSDARP00000052540 | ENSDARP00000016595 | 0 | 0 | 0 | 0 | 0,255 | 0 | 0 | 0 | 0,255 |
| bin2a | pah | 805066 | 804139 | ENSDARP00000019400 | ENSDARP00000012808 | 0 | 0 | 0 | 0 | 0,252 | 0 | 0 | 0 | 0,252 |
| nsun2 | rnaseh2a | 820047 | 808279 | ENSDARP00000094668 | ENSDARP00000045893 | 0 | 0 | 0 | 0 | 0,252 | 0 | 0 | 0 | 0,252 |
| runx2a | hdac1 | 820599 | 809066 | ENSDARP00000096167 | ENSDARP00000051798 | 0 | 0 | 0 | 0 | 0 | 0,238 | 0 | 0,08 | 0,252 |
| vsx1 | zgc:103600 | 814216 | 804773 | ENSDARP00000073224 | ENSDARP00000017354 | 0 | 0 | 0 | 0 | 0,253 | 0 | 0 | 0 | 0,252 |
| polr2d | rnaseh2a | 822340 | 808279 | ENSDARP00000099022 | ENSDARP00000045893 | 0 | 0 | 0 | 0 | 0,251 | 0 | 0 | 0 | 0,251 |
| rps28 | dph5 | 809108 | 805519 | ENSDARP00000052062 | ENSDARP00000022829 | 0 | 0 | 0 | 0 | 0,222 | 0 | 0 | 0,096 | 0,25 |
| oep | tcf7l1a | 818929 | 818257 | ENSDARP00000091797 | ENSDARP00000090035 | 0 | 0 | 0 | 0 | 0 | 0 | 0 | 0,25 | 0,249 |
| rln3 | jak2b | 816387 | 806262 | ENSDARP00000082236 | ENSDARP00000028132 | 0 | 0 | 0 | 0 | 0 | 0 | 0 | 0,25 | 0,249 |
| dlg1 | smad2 | 811146 | 808131 | ENSDARP00000061428 | ENSDARP00000044755 | 0 | 0 | 0 | 0 | 0,25 | 0 | 0 | 0 | 0,249 |
| rln3 | bin2a | 816387 | 805066 | ENSDARP00000082236 | ENSDARP00000019400 | 0 | 0 | 0 | 0 | 0,25 | 0 | 0 | 0 | 0,249 |
| oep | tbx5 | 818929 | 806747 | ENSDARP00000091797 | ENSDARP00000033053 | 0 | 0 | 0 | 0 | 0 | 0 | 0 | 0,249 | 0,249 |
| mrpl35 | srp19 | 820012 | 809061 | ENSDARP00000094603 | ENSDARP00000051775 | 0 | 0 | 0 | 0 | 0,248 | 0 | 0 | 0 | 0,248 |
| cx43 | rxrbb | 811110 | 805542 | ENSDARP00000061260 | ENSDARP00000022973 | 0 | 0 | 0 | 0 | 0 | 0 | 0 | 0,248 | 0,247 |
| polr2d | ufc1 | 822340 | 807157 | ENSDARP00000099022 | ENSDARP00000036899 | 0 | 0 | 0 | 0 | 0,248 | 0 | 0 | 0 | 0,247 |
| cx43 | gata6 | 811110 | 809096 | ENSDARP00000061260 | ENSDARP00000051997 | 0 | 0 | 0 | 0 | 0 | 0 | 0 | 0,246 | 0,246 |
| zgc:63700 | rxrbb | 807261 | 805542 | ENSDARP00000037835 | ENSDARP00000022973 | 0 | 0 | 0 | 0 | 0 | 0 | 0 | 0,246 | 0,246 |
| ccnb2 | zgc:56476 | 809228 | 804242 | ENSDARP00000052540 | ENSDARP00000013520 | 0 | 0 | 0 | 0 | 0,245 | 0 | 0 | 0 | 0,245 |
| pelo | dph5 | 813898 | 805519 | ENSDARP00000072295 | ENSDARP00000022829 | 0 | 0 | 0 | 0 | 0,245 | 0 | 0 | 0 | 0,245 |
| runx2a | arfip1 | 820599 | 819484 | ENSDARP00000096167 | ENSDARP00000093278 | 0 | 0 | 0 | 0 | 0 | 0 | 0 | 0,245 | 0,245 |
| polr2d | srp19 | 822340 | 809061 | ENSDARP00000099022 | ENSDARP00000051775 | 0 | 0 | 0 | 0 | 0,242 | 0 | 0 | 0 | 0,243 |
| mab21l2 | sox19a | 805084 | 805023 | ENSDARP00000019528 | ENSDARP00000019070 | 0 | 0 | 0 | 0 | 0,243 | 0 | 0 | 0 | 0,243 |
| pelo | snrpd1 | 813898 | 806216 | ENSDARP00000072295 | ENSDARP00000027770 | 0 | 0 | 0 | 0 | 0,103 | 0 | 0 | 0,209 | 0,243 |
| atp2b3a | foxi3b | 816845 | 802625 | ENSDARP00000084685 | ENSDARP00000002195 | 0 | 0 | 0 | 0 | 0 | 0 | 0 | 0,243 | 0,243 |
| atoh8 | cav3 | 809670 | 806896 | ENSDARP00000054688 | ENSDARP00000034317 | 0 | 0 | 0 | 0 | 0,241 | 0 | 0 | 0 | 0,241 |
| gpx4b | cox7a2 | 825605 | 813235 | ENSDARP00000103087 | ENSDARP00000069708 | 0 | 0 | 0 | 0 | 0,241 | 0 | 0 | 0 | 0,241 |
| polr2d | med21 | 822340 | 807146 | ENSDARP00000099022 | ENSDARP00000036773 | 0 | 0 | 0 | 0 | 0,241 | 0 | 0 | 0 | 0,241 |
| rln3 | sult1st6 | 816387 | 805633 | ENSDARP00000082236 | ENSDARP00000023637 | 0 | 0 | 0 | 0 | 0,241 | 0 | 0 | 0 | 0,241 |
| oep | vent | 818929 | 803869 | ENSDARP00000091797 | ENSDARP00000011087 | 0 | 0 | 0 | 0 | 0 | 0 | 0 | 0,242 | 0,241 |
| mab21l2 | bin2a | 805084 | 805066 | ENSDARP00000019528 | ENSDARP00000019400 | 0 | 0 | 0 | 0 | 0,238 | 0 | 0 | 0 | 0,238 |
| cyt1 | sepw1 | 820493 | 808886 | ENSDARP00000095806 | ENSDARP00000050883 | 0 | 0 | 0 | 0 | 0,238 | 0 | 0 | 0 | 0,238 |
| cav3 | mab21l2 | 806896 | 805084 | ENSDARP00000034317 | ENSDARP00000019528 | 0 | 0 | 0 | 0 | 0,238 | 0 | 0 | 0 | 0,238 |
| nsun2 | dph5 | 820047 | 805519 | ENSDARP00000094668 | ENSDARP00000022829 | 0 | 0 | 0 | 0 | 0,236 | 0 | 0 | 0 | 0,235 |
| ufc1 | atp5d | 807157 | 805466 | ENSDARP00000036899 | ENSDARP00000022528 | 0 | 0 | 0 | 0 | 0,233 | 0 | 0 | 0 | 0,233 |
| fgg | mab21l2 | 809584 | 805084 | ENSDARP00000054229 | ENSDARP00000019528 | 0 | 0 | 0 | 0 | 0,229 | 0 | 0 | 0 | 0,229 |
| mrpl35 | atp5d | 820012 | 805466 | ENSDARP00000094603 | ENSDARP00000022528 | 0 | 0 | 0 | 0 | 0,228 | 0 | 0 | 0 | 0,228 |
| zgc:56497 | zgc:56476 | 804660 | 804242 | ENSDARP00000016595 | ENSDARP00000013520 | 0 | 0 | 0 | 0 | 0,224 | 0 | 0 | 0 | 0,224 |
| lrrc42 | zgc:56497 | 808161 | 804660 | ENSDARP00000045055 | ENSDARP00000016595 | 0 | 0 | 0 | 0 | 0,225 | 0 | 0 | 0 | 0,224 |
| atp1a1a.4 | tpma | 808633 | 807449 | ENSDARP00000048827 | ENSDARP00000039656 | 0 | 0 | 0 | 0 | 0,178 | 0 | 0 | 0,112 | 0,22 |
| polr2d | yeats4 | 822340 | 812589 | ENSDARP00000099022 | ENSDARP00000067426 | 0 | 0 | 0 | 0 | 0,217 | 0 | 0 | 0 | 0,217 |
| hdac1 | dcps | 809066 | 805415 | ENSDARP00000051798 | ENSDARP00000022189 | 0 | 0 | 0 | 0 | 0,216 | 0 | 0 | 0 | 0,216 |
| atoh8 | msxc | 809670 | 805788 | ENSDARP00000054688 | ENSDARP00000024644 | 0 | 0 | 0 | 0 | 0,216 | 0 | 0 | 0 | 0,216 |
| acat1 | zgc:92317 | 812597 | 808453 | ENSDARP00000067447 | ENSDARP00000047376 | 0 | 0 | 0 | 0 | 0,215 | 0 | 0 | 0 | 0,215 |
| zgc:92000 | ufc1 | 813013 | 807157 | ENSDARP00000068896 | ENSDARP00000036899 | 0 | 0 | 0 | 0 | 0,213 | 0 | 0 | 0 | 0,213 |
| fgg | bin2a | 809584 | 805066 | ENSDARP00000054229 | ENSDARP00000019400 | 0 | 0 | 0 | 0 | 0,213 | 0 | 0 | 0 | 0,213 |
| rln3 | fgg | 816387 | 809584 | ENSDARP00000082236 | ENSDARP00000054229 | 0 | 0 | 0 | 0 | 0,212 | 0 | 0 | 0 | 0,212 |
| zgc:100903 | mab21l2 | 809081 | 805084 | ENSDARP00000051900 | ENSDARP00000019528 | 0 | 0 | 0 | 0 | 0,212 | 0 | 0 | 0 | 0,212 |
| bin2a | rdh1 | 805066 | 802867 | ENSDARP00000019400 | ENSDARP00000003828 | 0 | 0 | 0 | 0 | 0,21 | 0 | 0 | 0 | 0,21 |
| mif | hspa5 | 820608 | 804785 | ENSDARP00000096200 | ENSDARP00000017456 | 0 | 0 | 0 | 0 | 0 | 0 | 0 | 0,21 | 0,21 |
| id1 | tfcp2l1 | 810779 | 807569 | ENSDARP00000059731 | ENSDARP00000040479 | 0 | 0 | 0 | 0 | 0 | 0 | 0 | 0,211 | 0,21 |
| abcb3l1 | hspa5 | 806339 | 804785 | ENSDARP00000028924 | ENSDARP00000017456 | 0 | 0 | 0 | 0 | 0 | 0 | 0 | 0,208 | 0,208 |
| ak3 | stom | 814910 | 814738 | ENSDARP00000075506 | ENSDARP00000074954 | 0 | 0 | 0 | 0 | 0,209 | 0 | 0 | 0 | 0,208 |
| zgc:92317 | rdh1 | 808453 | 802867 | ENSDARP00000047376 | ENSDARP00000003828 | 0 | 0 | 0 | 0 | 0,208 | 0 | 0 | 0 | 0,208 |
| mrpl39 | snrpd1 | 816434 | 806216 | ENSDARP00000082504 | ENSDARP00000027770 | 0 | 0 | 0 | 0 | 0,207 | 0 | 0 | 0 | 0,207 |
| zgc:162286 | ptrh1 | 816146 | 808807 | ENSDARP00000080966 | ENSDARP00000050195 | 0 | 0 | 0 | 0 | 0 | 0 | 0 | 0,206 | 0,206 |
| abcb3l1 | rxrbb | 806339 | 805542 | ENSDARP00000028924 | ENSDARP00000022973 | 0 | 0 | 0 | 0 | 0 | 0 | 0 | 0,206 | 0,205 |
| ak3 | vent | 814910 | 803869 | ENSDARP00000075506 | ENSDARP00000011087 | 0 | 0 | 0 | 0 | 0,205 | 0 | 0 | 0 | 0,205 |
| ak3 | cndp2 | 814910 | 806824 | ENSDARP00000075506 | ENSDARP00000033632 | 0 | 0 | 0 | 0 | 0,205 | 0 | 0 | 0 | 0,205 |
| nsun2 | zgc:56476 | 820047 | 804242 | ENSDARP00000094668 | ENSDARP00000013520 | 0 | 0 | 0 | 0 | 0,206 | 0 | 0 | 0 | 0,205 |
| yeats4 | hdac1 | 812589 | 809066 | ENSDARP00000067426 | ENSDARP00000051798 | 0 | 0 | 0 | 0 | 0,132 | 0,124 | 0 | 0,083 | 0,205 |
| fads2 | foxn4 | 805447 | 805192 | ENSDARP00000022396 | ENSDARP00000020367 | 0 | 0 | 0 | 0 | 0 | 0 | 0 | 0,206 | 0,205 |
| dlx6a | trim13 | 819605 | 804237 | ENSDARP00000093553 | ENSDARP00000013495 | 0 | 0 | 0 | 0 | 0 | 0 | 0 | 0,205 | 0,205 |
| mrpl35 | ufc1 | 820012 | 807157 | ENSDARP00000094603 | ENSDARP00000036899 | 0 | 0 | 0 | 0 | 0,201 | 0 | 0 | 0 | 0,202 |
| tmub2 | rmi1 | 814279 | 810979 | ENSDARP00000073431 | ENSDARP00000060656 | 0 | 0 | 0 | 0 | 0 | 0 | 0 | 0,203 | 0,202 |
| rhbg | zgc:103600 | 810580 | 804773 | ENSDARP00000058869 | ENSDARP00000017354 | 0 | 0 | 0 | 0 | 0,201 | 0 | 0 | 0 | 0,201 |
| stom | ccnb2 | 814738 | 809228 | ENSDARP00000074954 | ENSDARP00000052540 | 0 | 0 | 0 | 0 | 0 | 0 | 0 | 0,202 | 0,201 |
| sult1st6 | pah | 805633 | 804139 | ENSDARP00000023637 | ENSDARP00000012808 | 0 | 0 | 0 | 0 | 0,202 | 0 | 0 | 0 | 0,201 |
| gata6 | rxrbb | 809096 | 805542 | ENSDARP00000051997 | ENSDARP00000022973 | 0 | 0 | 0 | 0 | 0 | 0 | 0 | 0,202 | 0,201 |
| myo5b | fads2 | 816617 | 805447 | ENSDARP00000083463 | ENSDARP00000022396 | 0 | 0 | 0 | 0 | 0 | 0 | 0 | 0,202 | 0,201 |
| ccnb2 | fabp7b | 809228 | 807655 | ENSDARP00000052540 | ENSDARP00000041228 | 0 | 0 | 0 | 0 | 0 | 0 | 0 | 0,201 | 0,201 |
| cx43 | bin2a | 811110 | 805066 | ENSDARP00000061260 | ENSDARP00000019400 | 0 | 0 | 0 | 0 | 0,202 | 0 | 0 | 0 | 0,201 |
| oep | fgg | 818929 | 809584 | ENSDARP00000091797 | ENSDARP00000054229 | 0 | 0 | 0 | 0 | 0,199 | 0 | 0 | 0 | 0,199 |
| ak3 | sox19a | 814910 | 805023 | ENSDARP00000075506 | ENSDARP00000019070 | 0 | 0 | 0 | 0 | 0,198 | 0 | 0 | 0 | 0,198 |
| fads2 | pah | 805447 | 804139 | ENSDARP00000022396 | ENSDARP00000012808 | 0 | 0 | 0 | 0 | 0,198 | 0 | 0 | 0 | 0,198 |
| zgc:92635 | snrpd1 | 812373 | 806216 | ENSDARP00000066703 | ENSDARP00000027770 | 0 | 0 | 0 | 0 | 0,196 | 0 | 0 | 0 | 0,197 |
| bin2a | zgc:103600 | 805066 | 804773 | ENSDARP00000019400 | ENSDARP00000017354 | 0 | 0 | 0 | 0 | 0,197 | 0 | 0 | 0 | 0,196 |
| dmap1 | rnaseh2a | 811199 | 808279 | ENSDARP00000061678 | ENSDARP00000045893 | 0 | 0 | 0 | 0 | 0,196 | 0 | 0 | 0 | 0,196 |
| mrpl35 | cox7a2 | 820012 | 813235 | ENSDARP00000094603 | ENSDARP00000069708 | 0 | 0 | 0 | 0 | 0,197 | 0 | 0 | 0 | 0,196 |
| tcf7l1a | msxc | 818257 | 805788 | ENSDARP00000090035 | ENSDARP00000024644 | 0 | 0 | 0 | 0 | 0 | 0 | 0 | 0,193 | 0,193 |
| zgc:100903 | abcb3l1 | 809081 | 806339 | ENSDARP00000051900 | ENSDARP00000028924 | 0 | 0 | 0 | 0 | 0 | 0 | 0 | 0,194 | 0,193 |
| rln3 | rdh1 | 816387 | 802867 | ENSDARP00000082236 | ENSDARP00000003828 | 0 | 0 | 0 | 0 | 0,193 | 0 | 0 | 0 | 0,193 |
| msxc | efnb2a | 805788 | 803776 | ENSDARP00000024644 | ENSDARP00000010432 | 0 | 0 | 0 | 0 | 0 | 0 | 0 | 0,192 | 0,191 |
| hdac1 | rxrbb | 809066 | 805542 | ENSDARP00000051798 | ENSDARP00000022973 | 0 | 0 | 0 | 0 | 0 | 0 | 0 | 0,191 | 0,191 |
| rln3 | zgc:100903 | 816387 | 809081 | ENSDARP00000082236 | ENSDARP00000051900 | 0 | 0 | 0 | 0 | 0,191 | 0 | 0 | 0 | 0,191 |
| cx43 | smad5 | 811110 | 809568 | ENSDARP00000061260 | ENSDARP00000054174 | 0 | 0 | 0 | 0 | 0 | 0 | 0 | 0,19 | 0,19 |
| mif | atp5d | 820608 | 805466 | ENSDARP00000096200 | ENSDARP00000022528 | 0 | 0 | 0 | 0 | 0,189 | 0 | 0 | 0 | 0,19 |
| ccnb2 | sox19a | 809228 | 805023 | ENSDARP00000052540 | ENSDARP00000019070 | 0 | 0 | 0 | 0 | 0 | 0 | 0 | 0,189 | 0,188 |
| atoh8 | fads2 | 809670 | 805447 | ENSDARP00000054688 | ENSDARP00000022396 | 0 | 0 | 0 | 0 | 0,188 | 0 | 0 | 0 | 0,188 |
| mab21l2 | hoxb10a | 805084 | 804701 | ENSDARP00000019528 | ENSDARP00000016856 | 0 | 0 | 0 | 0 | 0,188 | 0 | 0 | 0 | 0,188 |
| oep | rln3 | 818929 | 816387 | ENSDARP00000091797 | ENSDARP00000082236 | 0 | 0 | 0 | 0 | 0,188 | 0 | 0 | 0 | 0,188 |
| foxn4 | mab21l2 | 805192 | 805084 | ENSDARP00000020367 | ENSDARP00000019528 | 0 | 0 | 0 | 0 | 0,188 | 0 | 0 | 0 | 0,188 |
| ptgr1 | aldh5a1 | 807364 | 804078 | ENSDARP00000038835 | ENSDARP00000012423 | 0 | 0 | 0 | 0 | 0 | 0 | 0,186 | 0 | 0,186 |
| hdac1 | snrpd1 | 809066 | 806216 | ENSDARP00000051798 | ENSDARP00000027770 | 0 | 0 | 0 | 0 | 0,184 | 0 | 0 | 0 | 0,185 |
| taf7 | yeats4 | 813037 | 812589 | ENSDARP00000068986 | ENSDARP00000067426 | 0 | 0 | 0 | 0 | 0 | 0 | 0 | 0,186 | 0,185 |
| cox7a2 | ufc1 | 813235 | 807157 | ENSDARP00000069708 | ENSDARP00000036899 | 0 | 0 | 0 | 0 | 0,185 | 0 | 0 | 0 | 0,185 |
| fads2 | eif4a1b | 805447 | 805003 | ENSDARP00000022396 | ENSDARP00000018923 | 0 | 0 | 0 | 0 | 0,184 | 0 | 0 | 0 | 0,184 |
| gpx4b | snrpd1 | 825605 | 806216 | ENSDARP00000103087 | ENSDARP00000027770 | 0 | 0 | 0 | 0 | 0 | 0 | 0 | 0,183 | 0,183 |
| fkbp9 | sox19a | 805411 | 805023 | ENSDARP00000022147 | ENSDARP00000019070 | 0 | 0 | 0 | 0 | 0 | 0 | 0 | 0,183 | 0,183 |
| tpma | cav3 | 807449 | 806896 | ENSDARP00000039656 | ENSDARP00000034317 | 0 | 0 | 0 | 0 | 0,183 | 0 | 0 | 0 | 0,183 |
| mif | il1b | 820608 | 802642 | ENSDARP00000096200 | ENSDARP00000002293 | 0 | 0 | 0 | 0 | 0 | 0 | 0 | 0,182 | 0,182 |
| sepw1 | cav3 | 808886 | 806896 | ENSDARP00000050883 | ENSDARP00000034317 | 0 | 0 | 0 | 0 | 0,18 | 0 | 0 | 0 | 0,18 |
| dlx6a | oep | 819605 | 818929 | ENSDARP00000093553 | ENSDARP00000091797 | 0 | 0 | 0 | 0 | 0,119 | 0 | 0 | 0,127 | 0,179 |
| spsb4b | ccnb2 | 810695 | 809228 | ENSDARP00000059326 | ENSDARP00000052540 | 0 | 0 | 0 | 0 | 0,179 | 0 | 0 | 0 | 0,179 |
| atp2b3a | atp1a1a.4 | 816845 | 808633 | ENSDARP00000084685 | ENSDARP00000048827 | 0 | 0 | 0,502 | 0,738 | 0 | 0 | 0 | 0 | 0,178 |
| gata6 | vent | 809096 | 803869 | ENSDARP00000051997 | ENSDARP00000011087 | 0 | 0 | 0 | 0 | 0,124 | 0 | 0 | 0,12 | 0,177 |
| rnaseh2a | dph5 | 808279 | 805519 | ENSDARP00000045893 | ENSDARP00000022829 | 0 | 0 | 0 | 0 | 0,177 | 0 | 0 | 0 | 0,177 |
| mrpl39 | dph5 | 816434 | 805519 | ENSDARP00000082504 | ENSDARP00000022829 | 0 | 0 | 0 | 0 | 0,175 | 0 | 0 | 0 | 0,175 |
| srp19 | med21 | 809061 | 807146 | ENSDARP00000051775 | ENSDARP00000036773 | 0 | 0 | 0 | 0 | 0,175 | 0 | 0 | 0 | 0,175 |
| hdac1 | cbx1a | 809066 | 806158 | ENSDARP00000051798 | ENSDARP00000027355 | 0 | 0 | 0 | 0 | 0,165 | 0 | 0 | 0,073 | 0,174 |
| ak3 | bin2a | 814910 | 805066 | ENSDARP00000075506 | ENSDARP00000019400 | 0 | 0 | 0 | 0 | 0,175 | 0 | 0 | 0 | 0,174 |
| vsx1 | epb4.1l4 | 814216 | 810525 | ENSDARP00000073224 | ENSDARP00000058626 | 0 | 0 | 0 | 0 | 0,172 | 0 | 0 | 0 | 0,172 |
| ctrb1 | fads2 | 807080 | 805447 | ENSDARP00000036127 | ENSDARP00000022396 | 0 | 0 | 0 | 0 | 0,171 | 0 | 0 | 0 | 0,171 |
| stom | fkbp9 | 814738 | 805411 | ENSDARP00000074954 | ENSDARP00000022147 | 0 | 0 | 0 | 0 | 0,168 | 0 | 0 | 0 | 0,168 |
| zgc:92635 | ufc1 | 812373 | 807157 | ENSDARP00000066703 | ENSDARP00000036899 | 0 | 0 | 0 | 0 | 0,168 | 0 | 0 | 0 | 0,168 |
| acat1 | zgc:103600 | 812597 | 804773 | ENSDARP00000067447 | ENSDARP00000017354 | 0 | 0 | 0 | 0 | 0,169 | 0 | 0 | 0 | 0,168 |
| yeats4 | dph5 | 812589 | 805519 | ENSDARP00000067426 | ENSDARP00000022829 | 0 | 0 | 0 | 0 | 0,168 | 0 | 0 | 0 | 0,168 |
| mrpl39 | yeats4 | 816434 | 812589 | ENSDARP00000082504 | ENSDARP00000067426 | 0 | 0 | 0 | 0 | 0,168 | 0 | 0 | 0 | 0,168 |
| mibp | abcb3l1 | 822573 | 806339 | ENSDARP00000099308 | ENSDARP00000028924 | 0 | 0 | 0 | 0 | 0 | 0 | 0 | 0,167 | 0,167 |
| zgc:92635 | spsb4b | 812373 | 810695 | ENSDARP00000066703 | ENSDARP00000059326 | 0 | 0 | 0 | 0 | 0 | 0,167 | 0 | 0 | 0,167 |
| mif | cox7a2 | 820608 | 813235 | ENSDARP00000096200 | ENSDARP00000069708 | 0 | 0 | 0 | 0 | 0,167 | 0 | 0 | 0 | 0,167 |
| runx2a | cx43 | 820599 | 811110 | ENSDARP00000096167 | ENSDARP00000061260 | 0 | 0 | 0 | 0 | 0 | 0 | 0 | 0,165 | 0,165 |
| ak3 | srp19 | 814910 | 809061 | ENSDARP00000075506 | ENSDARP00000051775 | 0 | 0 | 0 | 0 | 0,166 | 0 | 0 | 0 | 0,165 |
| smad5 | ccnb2 | 809568 | 809228 | ENSDARP00000054174 | ENSDARP00000052540 | 0 | 0 | 0 | 0 | 0,166 | 0 | 0 | 0 | 0,165 |
| atoh8 | foxn4 | 809670 | 805192 | ENSDARP00000054688 | ENSDARP00000020367 | 0 | 0 | 0 | 0 | 0,166 | 0 | 0 | 0 | 0,165 |
| fabp7b | opn1mw1 | 807655 | 802568 | ENSDARP00000041228 | ENSDARP00000001158 | 0 | 0 | 0 | 0 | 0 | 0 | 0 | 0,165 | 0,165 |
| msxc | hoxb10a | 805788 | 804701 | ENSDARP00000024644 | ENSDARP00000016856 | 0 | 0 | 0 | 0,702 | 0,165 | 0 | 0 | 0 | 0,165 |
| yeats4 | dcps | 812589 | 805415 | ENSDARP00000067426 | ENSDARP00000022189 | 0 | 0 | 0 | 0 | 0,164 | 0 | 0 | 0 | 0,164 |
| yeats4 | ufc1 | 812589 | 807157 | ENSDARP00000067426 | ENSDARP00000036899 | 0 | 0 | 0 | 0 | 0,164 | 0 | 0 | 0 | 0,164 |
| mrpl35 | dph5 | 820012 | 805519 | ENSDARP00000094603 | ENSDARP00000022829 | 0 | 0 | 0 | 0 | 0,164 | 0 | 0 | 0 | 0,163 |
| epb4.1l4 | sox19a | 810525 | 805023 | ENSDARP00000058626 | ENSDARP00000019070 | 0 | 0 | 0 | 0 | 0,164 | 0 | 0 | 0 | 0,163 |
| mrpl39 | dmap1 | 816434 | 811199 | ENSDARP00000082504 | ENSDARP00000061678 | 0 | 0 | 0 | 0 | 0,163 | 0 | 0 | 0 | 0,163 |
| ntf7 | jak2b | 820551 | 806262 | ENSDARP00000096012 | ENSDARP00000028132 | 0 | 0 | 0 | 0 | 0 | 0 | 0 | 0,164 | 0,163 |
| cav3 | jak2b | 806896 | 806262 | ENSDARP00000034317 | ENSDARP00000028132 | 0 | 0 | 0 | 0 | 0 | 0 | 0 | 0,164 | 0,163 |
| trim54 | hspa5 | 808714 | 804785 | ENSDARP00000049429 | ENSDARP00000017456 | 0 | 0 | 0 | 0 | 0 | 0 | 0 | 0,164 | 0,163 |
| acat1 | pah | 812597 | 804139 | ENSDARP00000067447 | ENSDARP00000012808 | 0 | 0 | 0 | 0 | 0 | 0 | 0 | 0,164 | 0,163 |
| yeats4 | rnaseh2a | 812589 | 808279 | ENSDARP00000067426 | ENSDARP00000045893 | 0 | 0 | 0 | 0 | 0,163 | 0 | 0 | 0 | 0,163 |
| fgg | sult1st6 | 809584 | 805633 | ENSDARP00000054229 | ENSDARP00000023637 | 0 | 0 | 0 | 0 | 0,162 | 0 | 0 | 0 | 0,162 |
| wu:fb01b03 | ak3 | 822221 | 814910 | ENSDARP00000098882 | ENSDARP00000075506 | 0 | 0 | 0 | 0 | 0,162 | 0 | 0 | 0 | 0,162 |
| zgc:64148 | cbx1a | 813075 | 806158 | ENSDARP00000069092 | ENSDARP00000027355 | 0 | 0 | 0 | 0 | 0,163 | 0 | 0 | 0 | 0,162 |
| smad2 | rxrbb | 808131 | 805542 | ENSDARP00000044755 | ENSDARP00000022973 | 0 | 0 | 0 | 0 | 0 | 0 | 0 | 0,16 | 0,16 |
| zgc:92317 | fads2 | 808453 | 805447 | ENSDARP00000047376 | ENSDARP00000022396 | 0 | 0 | 0 | 0 | 0,161 | 0 | 0 | 0 | 0,16 |
| nsun2 | tpst1l | 820047 | 812090 | ENSDARP00000094668 | ENSDARP00000065364 | 0 | 0 | 0 | 0 | 0,159 | 0 | 0 | 0 | 0,159 |
| cpox | atp5d | 816647 | 805466 | ENSDARP00000083605 | ENSDARP00000022528 | 0 | 0 | 0 | 0 | 0 | 0 | 0 | 0,159 | 0,159 |
| oep | bin2a | 818929 | 805066 | ENSDARP00000091797 | ENSDARP00000019400 | 0 | 0 | 0 | 0 | 0,159 | 0 | 0 | 0 | 0,159 |
| polr2d | mrpl39 | 822340 | 816434 | ENSDARP00000099022 | ENSDARP00000082504 | 0 | 0 | 0 | 0 | 0,159 | 0 | 0 | 0 | 0,159 |
| yeats4 | srp19 | 812589 | 809061 | ENSDARP00000067426 | ENSDARP00000051775 | 0 | 0 | 0 | 0 | 0,157 | 0 | 0 | 0 | 0,157 |
| zgc:92317 | zgc:103600 | 808453 | 804773 | ENSDARP00000047376 | ENSDARP00000017354 | 0 | 0 | 0 | 0 | 0,158 | 0 | 0 | 0 | 0,157 |
| gpx4b | rnaseh2a | 825605 | 808279 | ENSDARP00000103087 | ENSDARP00000045893 | 0 | 0 | 0 | 0 | 0 | 0 | 0 | 0,156 | 0,156 |
| msxc | foxn4 | 805788 | 805192 | ENSDARP00000024644 | ENSDARP00000020367 | 0 | 0 | 0 | 0 | 0,156 | 0 | 0 | 0 | 0,156 |
| atp2b3a | kcnip3 | 816845 | 811910 | ENSDARP00000084685 | ENSDARP00000064580 | 0 | 0 | 0 | 0 | 0,156 | 0 | 0 | 0 | 0,156 |
| dmap1 | dcps | 811199 | 805415 | ENSDARP00000061678 | ENSDARP00000022189 | 0 | 0 | 0 | 0 | 0,155 | 0 | 0 | 0 | 0,155 |
| mrpl35 | snrpd1 | 820012 | 806216 | ENSDARP00000094603 | ENSDARP00000027770 | 0 | 0 | 0 | 0 | 0,155 | 0 | 0 | 0 | 0,155 |
| oep | cav3 | 818929 | 806896 | ENSDARP00000091797 | ENSDARP00000034317 | 0 | 0 | 0 | 0 | 0,155 | 0 | 0 | 0 | 0,154 |
| sult3st2 | bin2a | 820753 | 805066 | ENSDARP00000096517 | ENSDARP00000019400 | 0 | 0 | 0 | 0 | 0,155 | 0 | 0 | 0 | 0,154 |
| zgc:103434 | zgc:92317 | 811552 | 808453 | ENSDARP00000063134 | ENSDARP00000047376 | 0 | 0 | 0 | 0 | 0,155 | 0 | 0 | 0 | 0,154 |
| josd2 | zgc:56497 | 814688 | 804660 | ENSDARP00000074792 | ENSDARP00000016595 | 0 | 0 | 0 | 0 | 0,155 | 0 | 0 | 0 | 0,154 |
| nsun2 | mrpl39 | 820047 | 816434 | ENSDARP00000094668 | ENSDARP00000082504 | 0 | 0 | 0 | 0 | 0,152 | 0 | 0 | 0 | 0,152 |
| pelo | daam1l | 813898 | 805939 | ENSDARP00000072295 | ENSDARP00000025716 | 0 | 0 | 0 | 0 | 0 | 0 | 0 | 0,152 | 0,152 |
| zgc:64148 | hspa5 | 813075 | 804785 | ENSDARP00000069092 | ENSDARP00000017456 | 0 | 0 | 0 | 0 | 0,152 | 0 | 0 | 0 | 0,152 |
| zgc:103434 | zgc:101654 | 811552 | 806636 | ENSDARP00000063134 | ENSDARP00000032009 | 0 | 0 | 0 | 0 | 0,152 | 0 | 0 | 0 | 0,152 |
| dlg1 | efnb2a | 811146 | 803776 | ENSDARP00000061428 | ENSDARP00000010432 | 0 | 0 | 0 | 0 | 0,151 | 0 | 0 | 0 | 0,151 |
