# Supplementary material for: Functional Categorization of Transcriptome in the Species Symphysodon aequifasciatus Pellegrin 1904 (Perciformes: Cichlidae) Exposed to Benzo[a]pyrene and Phenanthrene
Source: PLoS One. 2013 Dec 3;8(12):e81083. doi: 10.1371/journal.pone.0081083 (PMC3849039; doi:10.1371/journal.pone.0081083)
Supplement: Table S5 — The list of genes, in Symphysodon aequifasciatus exposed to benzo[a]pyrene and phenanthrene for 48 h, grouped in each of the five clusters using STRING software (v.9.1). (DOC) [file pone.0081083.s010.doc]

Table S5. The list of genes, in *Symphysodon aequifasciatus* exposed to benzo[a]pyrene and phenanthrene for 48h, grouped in each of the five clusters using STRING software (v.9.1).

| STRING protein name | gene symbol submitted | gene_name |
| --- | --- | --- |
|  |  |  |
| **Cluster 1** |  |  |
| abcb3l1 | abcb3l1 | ATP-binding cassette, sub-family B (MDR/TAP), member 3 like 1 |
| acat1 | acat1 | acetyl-Coenzyme A acetyltransferase 1 (acetoacetyl Coenzyme A thiolase) |
| bin2a | bin2a | bridging integrator 2a |
| epb4.1l4 | epb4.1l4 | erythrocyte protein band 4.1-like 4 |
| fgg | fgg | fibrinogen, gamma polypeptide |
| il1b | il1b | interleukin 1, beta |
| jak2b | jak2b | Janus kinase 2b |
| mab21l2 | mab21l2 | mab-21-like 2 |
| mibp | mibp | muscle-specific beta 1 integrin binding protein |
| ntf7 | ntf7 | neurotrophin 7 |
| pah | pah | phenylalanine hydroxylase |
| psme2 | psme2 | proteasome activator subunit 2 |
| rdh1 | rdh1 | retinol dehydrogenase 1 |
| rhbg | rhbg | Rhesus blood group, B glycoprotein |
| rln3 | rln3a | relaxin 3a |
| sult1st6 | sult1st6 | sulfotransferase family 1, cytosolic sulfotransferase 6 |
| sult3st2 | sult3st2 | sulfotransferase family 3, cytosolic sulfotransferase 2 |
| zgc:100903 | pltp | phospholipid transfer protein |
| zgc:92317 | crata | carnitine O-acetyltransferase a |
| zgc:103600 | ttc36 | tetratricopeptide repeat domain 36 |
|  |  |  |
| **Cluster2** |  |  |
| dlg1 | dlg1 | discs, large (Drosophila) homolog 1 |
| efnb2a | efnb2a | ephrin B2a |
| gata6 | gata6 | GATA-binding protein 6 |
| hoxb10a | hoxb10a | homeo box B10a |
| msxc | msxc | muscle segment homeobox C |
| nkx2.3 | nkx2.3 | NK2 transcription factor related 3 |
| oep | oep | one-eyed pinhead |
| smad2 | smad2 | MAD homolog 2 (Drosophila) |
| smad5 | smad5 | MAD homolog 5 (Drosophila) |
| sox19a | sox19a | SRY-box containing gene 19a |
| tbx5 | tbx5a | T-box 5a |
| tcf7l1a | tcf7l1a | transcription factor 7-like 1a (T-cell specific, HMG-box) |
| ENSDARG00000019426 | trio | triple functional domain (PTPRF interacting) |
| vent | vent | ventral expressed homeobox |
|  |  |  |
| **Cluster 3** |  |  |
| cbx1a | cbx1a | chromobox homolog 1a (HP1 beta homolog Drosophila) |
| ccnb2 | ccnb2 | cyclin B2 |
| zgc:64148 | cisd2 | CDGSH iron sulfur domain 2 |
| fabp7b | fabp7b | fatty acid binding protein 7, brain, b |
| josd2 | josd2 | Josephin domain containing 2 |
| zgc:56476 | larp7 | La ribonucleoprotein domain family, member 7 |
| lrrc42 | lrrc42 | leucine rich repeat containing 42 |
| mynn | mynn | myoneurin |
| opn1mw1 | opn1mw1 | opsin 1 (cone pigments), medium-wave-sensitive, 1 |
| slc16a3 | slc16a3 | solute carrier family 16 (monocarboxylic acid transporters), member 3 |
| spsb4b | spsb4b | splA/ryanodine receptor domain and SOCS box containing 4b |
| zgc:56497 | ttc9c | tetratricopeptide repeat domain 9C |
|  |  |  |
| **Cluster 4** |  |  |
| daam1l | daam1b | dishevelled associated activator of morphogenesis 1b |
| dph5 | dph5 | DPH5 homolog (S. cerevisiae) |
| mlx | mlx | MAX-like protein X |
| mrpl39 | mrpl39 | mitochondrial ribosomal protein L39 |
| nsun2 | nsun2 | NOL1/NOP2/Sun domain family, member 2 |
| pelo | pelo | pelota homolog (Drosophila) |
| polr2d | polr2d | polymerase (RNA) II (DNA directed) polypeptide D |
| rnaseh2a | rnaseh2a | ribonuclease H2, subunit A |
| rpap2 | rpap2 | RNA polymerase II associated protein 2 |
| rps28 | rps28 | ribosomal protein S28 |
| snrpd1 | snrpd1 | small nuclear ribonucleoprotein D1 polypeptide |
| srp19 | srp19 | signal recognition particle 19 |
| tpst1l | tpst1l | tyrosylprotein sulfotransferase 1, like |
| zgc:63700 | aqp7 | aquaporin 7 |
| zgc:92635 | tceb1b | transcription elongation factor B (SIII), polypeptide 1b |
|  |  |  |
| **Cluster 5** |  |  |
| atp5d | atp5d | ATP synthase, H+ transporting, mitochondrial F1 complex, delta subunit |
| cox7a2 | cox7a2 | cytochrome c oxidase, subunit VIIa 2 |
| cpox | cpox | coproporphyrinogen oxidase |
| fancd2 | fancd2 | Fanconi anemia, complementation group D2 |
| zgc:92000 | gltpd1 | glycolipid transfer protein domain containing 1 |
| gpx4b | gpx4b | glutathione peroxidase 4b |
| hspa5 | hspa5 | heat shock protein 5 |
| mif | mif | macrophage migration inhibitory factor |
| mrpl35 | mrpl35 | mitochondrial ribosomal protein L35 |
| rmi1 | rmi1 | RMI1, RecQ mediated genome instability 1, homolog (S. cerevisiae) |
| tmub2 | tmub2 | transmembrane and ubiquitin-like domain containing 2 |
| trim54 | trim54 | tripartite motif-containing 54 |
| ufc1 | ufc1 | ubiquitin-fold modifier conjugating enzyme 1 |
